# Using a Chemical Genetic Screen to Enhance Our Understanding of the Antibacterial Properties of Silver

**DOI:** 10.3390/genes9070344

**Published:** 2018-07-06

**Authors:** Natalie Gugala, Joe Lemire, Kate Chatfield-Reed, Ying Yan, Gordon Chua, Raymond J. Turner

**Affiliations:** 1 Department of Biological Sciences, University of Calgary, 2500 University Dr. NW, Calgary, AB T2N 1N4, Canada; ngugala@ucalgary.ca (N.G.); jalemire@ucalgary.ca (J.L.); kchatfieldreed@gmail.com (K.C.-R.); gchua@ucalgary.ca (G.C.); 2 Department of Mathematics and Statistics, University of Calgary, 2500 University Dr. NW, Calgary, AB T2N 1N4, Canada; ying.yan@ucalgary.ca

**Keywords:** silver, silver toxicity, silver resistance, Keio collection, *Escherichia coli*, antimicrobials

## Abstract

It is essential to understand the mechanisms by which a toxicant is capable of poisoning the bacterial cell. The mechanism of action of many biocides and toxins, including numerous ubiquitous compounds, is not fully understood. For example, despite the widespread clinical and commercial use of silver (Ag), the mechanisms describing how this metal poisons bacterial cells remains incomplete. To advance our understanding surrounding the antimicrobial action of Ag, we performed a chemical genetic screen of a mutant library of *Escherichia coli*—the Keio collection, in order to identify Ag sensitive or resistant deletion strains. Indeed, our findings corroborate many previously established mechanisms that describe the antibacterial effects of Ag, such as the disruption of iron-sulfur clusters containing proteins and certain cellular redox enzymes. However, the data presented here demonstrates that the activity of Ag within the bacterial cell is more extensive, encompassing genes involved in cell wall maintenance, quinone metabolism and sulfur assimilation. Altogether, this study provides further insight into the antimicrobial mechanism of Ag and the physiological adaption of *E*. *coli* to this metal.

## 1. Introduction

For centuries, metal compounds have been deployed as effective antimicrobial agents [1]. The use of silver (Ag) for antimicrobial purposes is a practice that dates back thousands of years [2] and is still implemented for medical purposes in an effort to curtail the rise of antimicrobial resistant pathogens [3,4,5,6], a threat that has once again surfaced as a clinical challenge [7,8,9,10].

Applications of Ag-based antimicrobials include: wound dressings [11] and other textiles [12], antiseptic formulations [13], nanoparticles [14], coatings [15], nanocomposites [16], polymers [17], and part of antibiotic combination therapies [18]. Many of these approaches have proven to be effective in controlling and eradicating pathogenic microorganisms. 

Presently, research in this field focuses on finding new formulations and utilities for Ag-based antimicrobials. Despite this, the identity of the cellular targets that are involved in Ag antimicrobial activities are known to a far lesser degree [19]. This current knowledge gap hinders the potential utility of Ag-based antimicrobials, and in turn the expansion of this metal as a therapeutic agent.

Previous studies examining the mechanisms of Ag resistance and toxicity have not provided a complete understanding of the global cellular effects of Ag exposure on the bacterial cell. Further, several studies fail to build upon preceding work and the literature is replete with contradicting reports, in part due to non-standardized conditions of study. Furthermore, it has been demonstrated that the speciation/oxidation state of Ag has substantial influence on toxicity, a factor that is dependent on the source of Ag ions [4], growth conditions, and is further complicated by the organism (species and strain) of interest [6].

Proposed mechanisms of metal toxicity include the production and propagation of reactive oxygen species through Fenton chemistry and antioxidant depletion, the disruption of iron-sulfur clusters, thiol coordination and the exchange of a catalytic/structural metal that leads to protein dysfunction, interference with nutrient uptake, and genotoxicity [19]. Microorganisms are able to withstand metal toxicity through several mechanisms such as reduced uptake, efflux, extracellular and intracellular sequestration, repair, metabolic by-pass and chemical modification [20]. Whether these mechanisms are solely responsible for cell death or resistance has yet to be determined. Still, what is understood is that metals demonstrate broad-spectrum activity and decreased target specificity [19] when compared to conventional antimicrobials.

In this work, we hypothesized that Ag exerts its effects on multiple targets both directly and indirectly, and thus various cellular systems may be altered by Ag exposure. To test this, we performed a genotypic screening workflow of a mutant library composed of 3985 strains, each containing a different inactivated non-essential gene in *Escherichia coli*. Using a comparable genome-wide workflow [21] and by use of transcriptomic profiling [22,23], similar approaches have been implemented in order to study the mechanisms of action caused by Ag. Despite this, genes conferring resistance to Ag when absent have been studied and compared to a far lesser degree than those that result in sensitivity when absent. Further, many previous approaches aimed at studying Ag toxicity and resistance have primarily examined the effects of Ag shock or rapid pulses of exposure, followed by the evaluation of gene expression. Hence, as a means of complementing existing work, we have identified a number of genes that are implicated in prolonged Ag resistance and/or toxicity, and mapped their metabolic function to their respective cellular system. 

## 2. Materials and Methods 

### 2.1. Escherichia coli Strains and Storage

The Keio collection [24]—a mutant library of 3985 single-gene *E. coli* BW25113 mutants (*lacI*^q^, *rrnB*_T14_, Δ*lacZ*_WJ19_, *hsdR*514, Δ*ara*BAD_AH33_, Δ*rha*BAD_LD78_)—was obtained from the National BioResource Project *E. coli*, (National Institute of Genetics, Shizuoka, Japan). All strains were initially stored at −80 °C in vials containing Lysogeny Broth (LB) media (VWR International, Mississauga, ON, Canada) with 30% glycerol (VWR International). For chemical genetic screening, the Keio collection was transferred, and subsequently arrayed into 96-well microtiter plates containing LB medium with 30% glycerol. Construction of the arrayed Keio collection and pre-culturing of the *E. coli* strains was carried out on LB agar (1.0%). For chemical genetic screening, M9 minimal media (6.8 g/L Na_2_HPO_4_, 3 g/L KH_2_PO_4_, 1 g/L NH_4_Cl, 0.5 g/L NaCl, 4 mg/L glucose, 0.5 mg/L MgSO_4_ and 0.1 mg/L CaCl_2_) containing Noble agar (1.0%) with and without silver nitrate (AgNO_3_) was used for treatment and control testing, respectively (all obtained from VWR International). 

Although the Keio collection strains are engineered with a kanamycin resistance cassette in place of the gene of interest [24], for our experiments here, we did not include this antibiotic in the growth media. Synergistic antimicrobial effects were found when bacterial cells were grown in the presence of Ag and kanamycin (data not included).

### 2.2. Stock Ag Solution

Silver nitrate (AgNO_3_) was obtained from Sigma-Aldrich (St. Louis, MO, USA). Stock solutions of Ag were made at equivalent molarities of Ag in distilled and deionized (dd)H_2_O and stored in glass vials for no longer than two weeks. 

### 2.3. Determination of the Minimal Inhibitory Concentration and Controls

The minimal inhibitory concentration was determined using a known Ag sensitive strain (*cusB*) and negative control strains (*lacA* and *lacY*). CusB is a part of the CusCFBA copper/silver efflux system [25]; therefore, it was anticipated that the absence of this gene would confer toxicity, denoted as a Ag sensitive hit. Further, LacA and LacY, are not expected to be involved in Ag resistance or toxicity. The aforementioned strains, along with the parent strain (wild type (WT)) were grown at 37 °C on M9 minimal media and Noble agar (1%) in the presence or absence of Ag at varying concentrations. The Ag concentration found to visibly decrease colony size in the *cusB* mutant and demonstrate no changes in colony size in the *lacA* and *lacY* mutants was selected. Furthermore, the latter mutants and the WT were grown in the presence of 100 μM ionic nitrate to ensure growth was not impeded by the accompanying counter ion. The full chemical genetic screen was challenged at time zero of inoculation in the presence of 100 μM AgNO_3_. 

### 2.4. Screening 

M9 minimal media Noble agar (1%) plates were prepared two days prior to use. Colony arrays in 96-format were produced and processed using a BM3 robot (S&P Robotics Inc., Toronto, ON, Canada). The strains were spotted using a 96-pin replicator, allowing for uniform application. Cells were transferred from the arrayed microtiter plates using the replicator onto LB agar plates. These plates were then grown overnight at 37 °C. Once grown, the colonies were spotted using the replicator onto two sets—with and without 100 μM AgNO_3_—of M9 minimal media Noble agar plates, and subsequently grown overnight at 37 °C. Images of both sets of plates were acquired using the spImager (S&P Robotics Inc.) and colony size, which is a measure of fitness, was determined using integrated image processing software. For each 96-colony array, four technical trials per strain were combined onto a single plate in 384-colony array format and three biological trials were performed. Therefore, each strain was tested a total of 12 times.

### 2.5. Normalization

Experimental factors such as incubation time and temperature, local nutrient availability, colony location, gradients in the growth medium and neighboring mutant fitness were all considered as independent variables that could contribute to systematic variation, and subsequently affect colony size. As a result, the colonies were normalized and scored using Synthetic Genetic Array Tools 1.0 (SGATools) [26]. Firstly, all of the plates were normalized to establish identical median colony size working on the assumption that most colonies exhibited WT fitness. Next, to ensure the colonies were directly comparable, colonies were rescaled, a factor that is primarily important for colonies close to the edge of the plate. Further, spatial smoothing accounted for partialities in each plate owing to inconsistencies, such as the thickness of the agar. Very large colonies, likely an indication of contamination among other factors, and those that were different from the corresponding technical replicates were removed. Lastly, colonies that were larger than anticipated and located next to colonies that were found to be smaller than anticipated were marked as potential false-positive hits. 

Following this normalization, the colonies were scored. Here, paired evaluation was completed by comparing the colony size (in the presence of Ag) to a matched control (in the absence of Ag). Fitness values were established, and the subsequent scores represented deviation from the fitness of the WT strain. Once normalized and scored, colonies displaying a reduction in size were indicative of a Ag sensitive hit and those displaying an increase in colony size qualified as a Ag resistant hit. Finally, the *p*-value was calculated as a two-tailed *t*-test and significance was determined using the Benjamini-Hochberg procedure, as a means of lowering the false discovery rate, which was selected to be 0.1. 

### 2.6. Data Mining and Analyses

Subsequent analyses were conducted using Pathway Tools Omics Dashboard, which surveys against the EcoCyc database [27]. This allowed for clustering of the hits into systems, subsystems, component subsystems, and lastly, into individual objects. It is important to note that genes can be found in multiple systems, since many are involved in a number of cellular processes.

Further, in order to identify biological processes most prominent under Ag challenge, enrichment analyses were conducted for the Ag resistant and Ag sensitive hits. To analyze the gene list, the Database for Annotation, Visualization and Integrated Discovery (DAVID) bioinformatics resource was utilized [28,29]. Lastly, as a means of exposing the direct (physical) and indirect (functional) protein-protein connectivity between the gene hits, the Search Tool for the Retrieval of Interacting Genes/Proteins (STRING) database was used [30]. Interactive node maps based on experimental, co-expression and gene fusion studies were generated based on genes defined in our chemical genetics screen.

## 3. Results and Discussion 

### 3.1. Genome-Wide Screen of Ag-Resistant and Ag-Sensitive Hits

**Figure 1:**
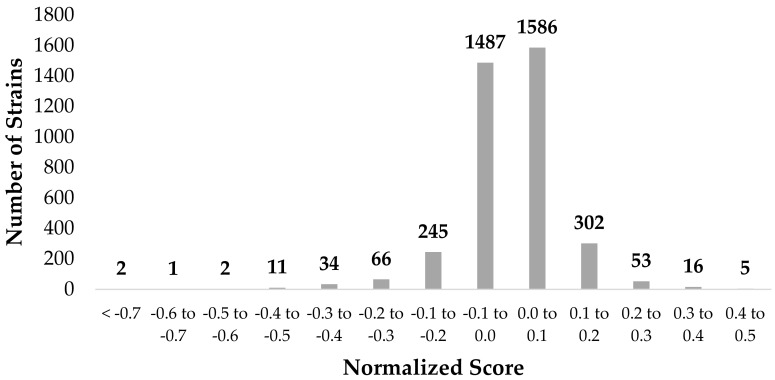
Synthetic Array Tools (version 1.0) was used to normalize and score the silver (Ag)-resistant and -sensitive gene hits as a means of representing the growth differences in *Escherichia coli* K12 BW25113 in the presence of 100 μM silver nitrate (AgNO_3_). Only those with a score greater or less than ±0.15, respectively, were selected for further analysis. Hits between ±0.15 were regarded as having neutral or non-specific interactions with Ag. The *p*-value was a two-tailed *t*-test and significance was determined using the Benjamini-Hochberg procedure; false discovery rate was selected to be 0.1. Each individual score represents the mean of 12 trials.

The chemical genetic screen completed in this work provided a method for genome-wide probing of non-essential genes involved in Ag-sensitivity or -resistance in *E. coli*. A total of 3810 non-essential genes were screened for growth in the presence of 100 μM AgNO_3_ (Supplementary Materials, Table S1). 3073 mutants displayed little change in colony size in the presence of Ag with a normalized fitness score between ±0.1 (Figure 1). The statistical colony size cutoff that indicated a significant difference in fitness was selected to be ±0.15, or two standard deviations from the mean. This resulted in 225 gene hits, which represents approximately 5% of the open reading frames in the *E*. *coli* genome. The remaining gene hits were not regarded as significant hits in this work based solely on the cut offs selected. In general, the normalization was performed on the assumption that Ag does not specifically interact with the deleted gene but rather impedes growth due to environmental stress. In short, those displaying hits between the cut off values were assumed to have non-specific or neutral interactions with Ag.

**Figure 2:**
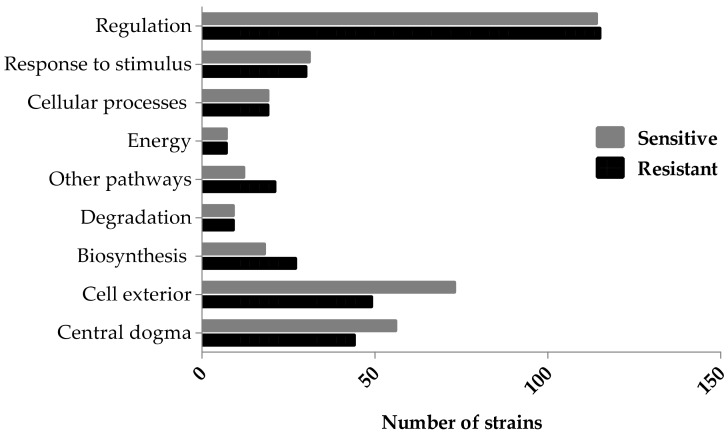
Ag-resistant and -sensitive gene hits mapped to component cellular processes. The cutoff fitness score implemented was −0.15 and 0.15 (two standard deviations from the mean) and the gene hits with a score less or greater than, respectively, were chosen for further analyses. The hits were mined using the Omics Dashboard (Pathway Tools), which surveys against the EcoCyc Database. Several gene hits are mapped to more than one subsystem. The *p*-value was calculated as a two-tailed *t*-test and significance was determined using the Benjamini-Hochberg procedure; false discovery rate was selected to be 0.1. Each individual score represents the mean of 12 trials.

It is important to note that when reflecting on the data generated from our chemical genetic screen, it is the absence of the gene that imparts the Ag-resistant or -sensitive phenotype. Upon Ag exposure, an increase in colony size (>0.15) is suggestive of an Ag-resistant hit, and therefore the presence of this gene is proposed to confer toxicity. On the contrary, a decrease in colony size (<−0.15) was designated to be an Ag-sensitive hit, and therefore the presence of this gene is proposed to confer resistance. In total, the deletion of 106 and 119 genes resulted in Ag-resistant and -sensitive hits, respectively (Table 1 and Table 2). These gene hits were mapped to their corresponding cellular systems using EcoCyc (Figure 2 and Table A1). In short, genes were found in multiple cellular systems, validating our hypothesis that Ag cytotoxicity and the corresponding physiological responses of *E. coli* involve a number of cellular mechanisms.

Comparable numbers of Ag-resistant and -sensitive hits were mapped in the systems ‘Response to stimulus’—starvation, heat, cold, DNA damage, pH, detoxification, osmotic stress, and other, ‘Cellular processes’—cell cycle and division, cell death, genetic transfer, biofilm formation, quorum sensing, adhesion, locomotion, viral response, response to bacterium, host interactions with host, other pathogenesis proteins, and ‘Degradation’—amino acids, nucleotide, amine, carbohydrate/carboxylate, secondary metabolite, alcohol, polymer and aromatic, the cell exterior, and regulation. A greater number of Ag-resistant than -sensitive hits were mapped to the processes ‘Biosynthesis’—amino acids, nucleotides, fatty acid/lipid amines, carbohydrate/carboxylates, cofactors, secondary metabolites, and other pathways and ‘Other pathways’—detoxification, inorganic nutrient metabolism, macromolecule modification, activation/inactivation/interconversion and other enzymes. 

In total, 49 and 73 resistant and sensitive hits, respectively, were found to be a part of the ‘Cell exterior’—transport, cell wall biogenesis and organization, lipopolysaccharide metabolism, pilus, flagellar, outer and inner membrane, periplasm, and cell wall components. Compared to the latter cellular processes, non-essential genes comprising ‘Energy’ processes—including glycolysis, the pentose phosphate pathway, the tricarboxylic acid (TCA) cycle, fermentation, and aerobic and anaerobic respiration were found to be involved in Ag toxicity or resistance the least, by more than seven-fold when compared to genes mapped to the ‘Cell exterior’. 

**Figure 3:**
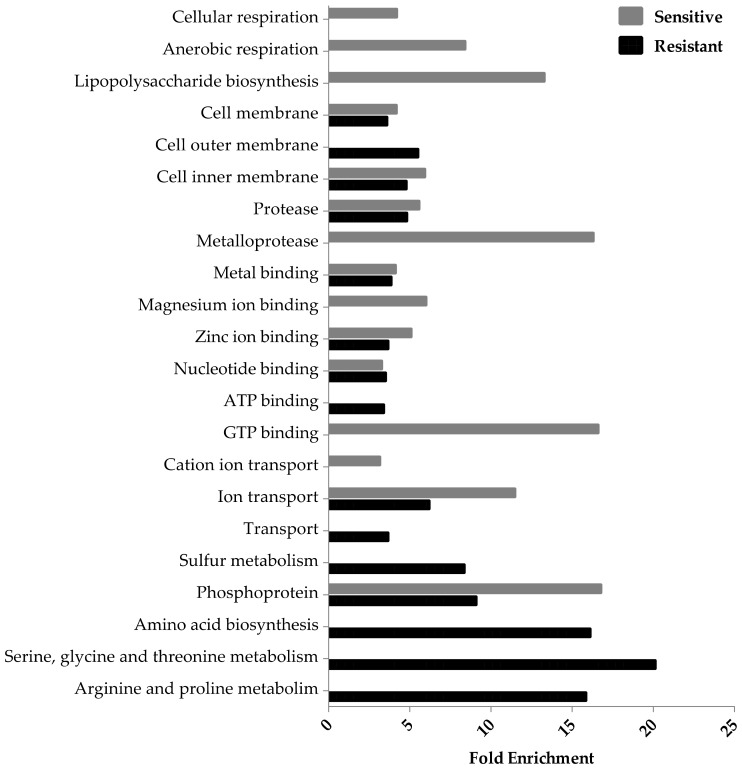
Functional enrichment among the Ag-resistant and -sensitive gene hits. The DAVID gene functional classification (version 6.8) database, a false discovery rate of 0.1 and a score cutoff of −0.15 and 0.15 (two standard deviations from the mean) were used to measure the magnitude of enrichment against the genome of *E. coli*. Processes with a *p*-value < 0.05, fold enrichment value ≥3 and gene hits >3 are included only. Each individual score represents the mean of 12 trials.

Based on the fold enrichment, metal binding proteins were affected to the same degree in both Ag-resistant and -sensitive groups, displaying an enrichment score <5 (Figure 3). However, when examining proteins involved with specific metals in more detail, such as zinc and magnesium, fold enrichment values were >5, but only for the Ag-sensitive hits (Figure 3). Cellular and anaerobic respiration were represented by the Ag-sensitive hits only, while processes involved in amino acid biosynthesis were heavily enriched for by the Ag-resistant hits.

A number of hits were found to be involved with the cell membrane using EcoCyc’s system of classification, but this was not detected in the fold enrichment analysis. Here, cell membrane proteins were affected three-fold less than the most highly represented clusters, which were amino acid biosynthesis and phosphoproteins for the Ag-resistant and -sensitive hits, respectively (Figure 3).

### 3.2. Ag-Resistant Gene Hits

#### 3.2.1. Regulators of Gene Expression 

**Figure 4:**
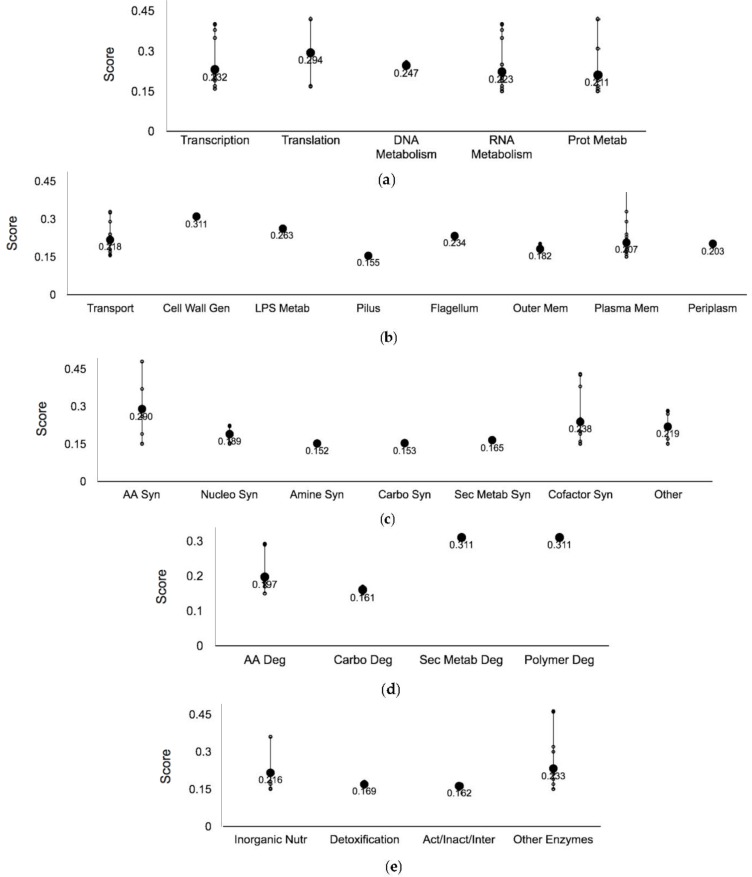

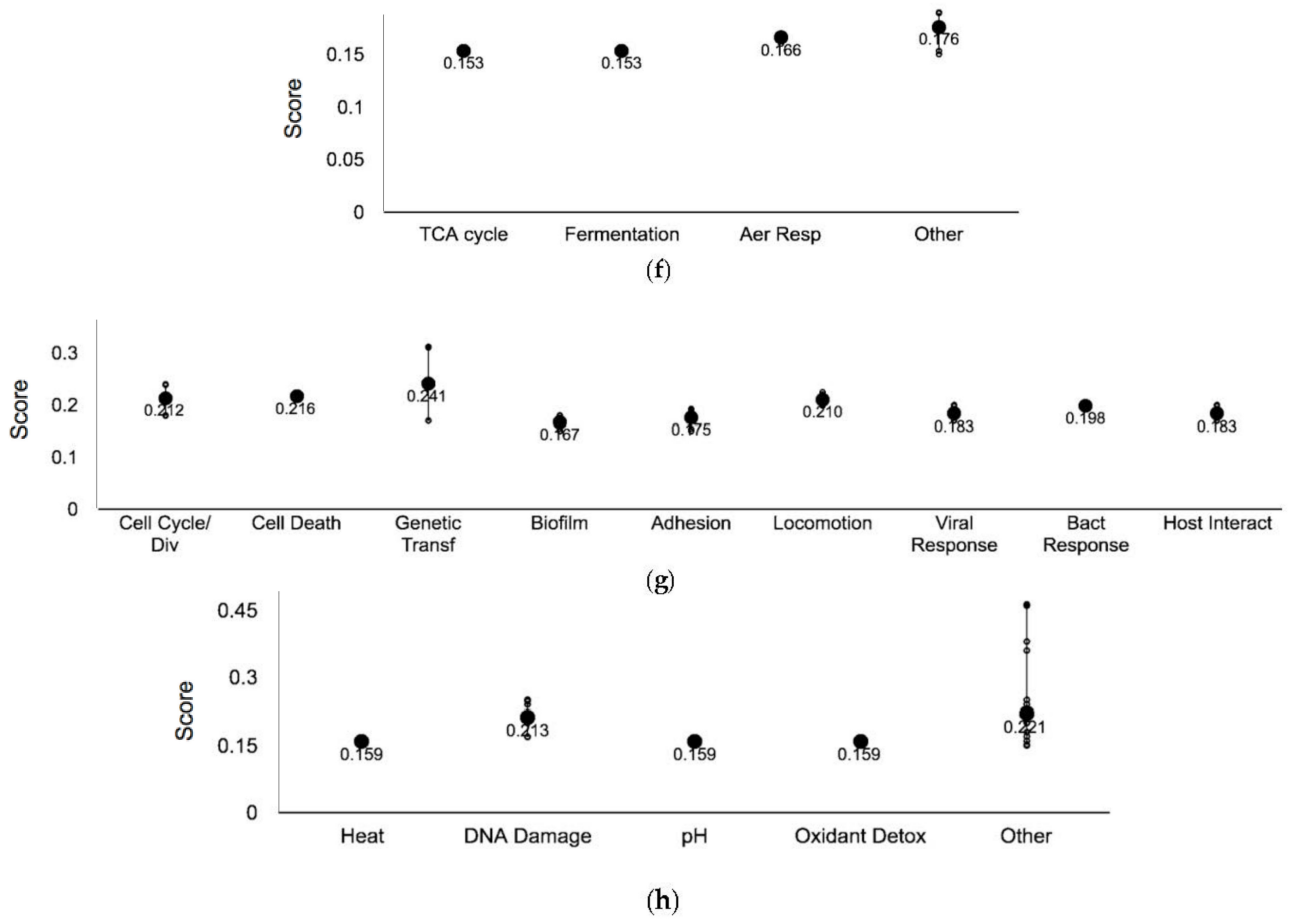
Ag-resistant gene hits plotted against respective cellular processes. Y-axis representative of the normalized score, smaller circles represent the individual hits and the larger circles represent the mean of each subsystem. The *p*-value was calculated as a two-tailed *t*-test and significance was determined using the Benjamini-Hochberg procedure; false discovery rate was selected to be 0.1. Each individual score represents the mean of 12 trials. (**a**) Central Dogma; (**b**) Cell exterior; (**c**) Biosynthesis; (**d**) Degradation; (**e**) Other pathways; (**f**) Energy; (**g**) Cellular processes; and (**h**) Response to stimulus. Plots constructed using Pathway Tools, Omics Dashboard.

When examining processes of the ‘Central dogma’—systems involved in replication, and transcription to translation—in more detail, each subsystem had a mean score between 0.211 and 0.294 (Figure 4a). Despite this consistency, transcription and RNA metabolism contained the greatest number of Ag-resistant hits, 12 and 16, respectively. The protein EttA—energy-dependent translational throttle protein [31], can be found within the subsystems translation and protein metabolism. EttA is sensitive to the energy state of the cell. This protein represses translational elongation in response to high ADP/ATP, stimulating dipeptide bond synthesis in the presence of ATP (cell high energy state) and vice versa. As a result, EttA may inhibit translation in Ag-treated cells due to the occurrence of high ADP/ATP ratios. The absence of EttA might allow for increased translation of proteins, such as RecA [32] or CusB [25], which may result in Ag resistance. Furthermore, six proteins involved in proteolysis were found to confer resistance when absent, such as Prc. This enzyme is a periplasmic protease that processes and degrades specific proteins, has been found to provide resistance against a number of small hydrophilic antibiotics and causes the leakage of periplasmic proteins when absent [33]. Antibiotic resistant mechanisms have been compared to those of metal ions, drawing on similarities such as substrate modification or sequestration. The leakage of the periplasmic proteins in *prc* mutants may result in Ag sequestration, thereby causing metal resistance.

#### 3.2.2. Cell Membrane Proteins 

It has been demonstrated that Ag may exert toxicity and potentially impede growth by acting on the cell membrane [34,35]. In this study, 49 coding genes that resulted in Ag resistance when absent were determined to be a part of the ‘Cell exterior’, which includes proteins of the cell membrane, periplasm and extracellular structures (Figure 2 and Figure 4b). Of these, 25 genes coded for plasma membrane proteins, and while Ag was observed to enter bacterial cells [23], the exact mechanism of import has yet to be determined. Loss of the porin genes *ompC* and *ompF* was observed to confer resistance to Ag [36]. While these two genes were not detected within our cut offs, we did recover two additional porin genes (*ompA* and *ompG*) as conferring Ag resistance when deleted. Relative to this, it has been demonstrated that a mechanism of entry for zinc into the cell is co-transport with low molecular weight metabolites via transport proteins found within the membrane [37]. Further, ExbB, a Ag-resistant hit with a score of 0.241, is part of the energy transducing Ton system that transports iron-siderophore complexes and vitamin B12 across the outer membrane [38]. Collectively, these findings provide insight into possible mechanisms of Ag import, such as entry through porins, co-transport with metabolites or the replacement of Ag with other ions predetermined for import. The enrichment analysis offered further evidence for this hypothesis, as a number of ion transport proteins and proteins pertaining to the cell membrane were involved in Ag resistance when absent (Figure 3). Furthermore, MngA, a permease that simultaneously phosphorylates 2-*O*-α-mannosyl-d-glycerate in a process called group translocation, contains two putative phosphorylation sites His^87^ and Cys^192^ [39]. Thiols are regarded as soft bases, and according to the hard-soft acid base theory, which is key to the reactivity and coordination of metals [40], cysteine, and to a lesser degree methionine and imidazole chemically interact with Ag(I) with high affinity. Therefore, proteins with key structural or catalytic thiols/imidazols are possible Ag interacting sites.

#### 3.2.3. Biosynthetic Enzymes

Eight hits were found to be involved in the biosynthesis of amino acids and 10 hits were found to be involved in cofactor/prosthetic group/electron carries catabolism (Figure 4c). When examining the functional enrichment analysis, serine, glycine, threonine, arginine and proline biosynthetic processes were highly enriched, on average three-fold more than the remaining cellular processes (Figure 3). The third step in the synthesis of NAD^+^ from l-aspartate occurs via the enzyme NadC—quinolinate phosphoribosyltransferase [41] and based on our data the absence of this protein confers resistance in *E. coli.* In fact, the genes coding for the first and second steps of de novo NAD^+^ synthesis, NadB—l-aspartate oxidase and NadA—quinolinate synthase, respectively, were also found to be Ag resistant hits. NadA contains a [4Fe-4S] cluster that is required for activity [42]. Soft metals have the capacity to inactivate dehydratases in vitro via iron-sulfur cluster degradation, possibly leading to the bridging of the sulfur atoms [43]. As a result, proteins with iron-sulfur centers are of possible interest when examining the interactions of Ag with cellular biomolecules. Furthermore, it has been demonstrated that H_2_O_2_ formation is diminished via the addition of precursors involved in the synthesis of NAD^+^ [44]. The absence of one gene involved in NAD^+^ biosynthesis may result in metabolite accumulation since there is no evidence of negative precursor feedback inhibition. Therefore, there is a possibility that deletion of the *nadA*, *nadB* or *nadC* may confer resistance if H_2_O_2_ is generated in the presence of Ag. 

Using the STRING database, several points of interaction were revealed. Among the Ag-resistant hits, the latter genes involved in de novo NAD^+^ production were connected to proteins a part of amino acid biosynthesis, including *trpB*, *aroC*, and *metL* (Supplementary Materials, Figure S2). 

#### 3.2.4. Catabolic Enzymes

Genes encoding enzymes functioning in the catabolism of metabolites, such as amino acids, fatty acids, carbohydrates and polymers, were underrepresented compared to anabolism (Figure 2 and Figure 4d). In fact, in the functional enrichment analysis, degradation processes were not represented within the cutoffs selected (Figure 3). The gene *idcA*—l,d-carboxypeptidase, a component of secondary metabolite and polymer degradation, had an elevated score of 0.311. IdcA is essential for murein turnover [45]. Murein processing is an important energy-conserving activity that transports cell wall components from the exterior of the cell to the cytoplasm [46]. Evidence has demonstrated that during logarithmic growth, the *idcA* mutant strain displays a decrease in the overall cross-linkage of murein, causing a reduction in turnover and the abundance of murein being transported into the cell. In turn, this may result in the transport of fewer Ag ions, which may have bound to the cell wall, into the cell thereby prompting increased resistance in the *idcA* mutant strain. Metal nanoparticles have been proposed to target the outer membrane regions of bacteria due to strong electrostatic interactions and co-coordination of the metal with the lipopolysaccharide or similar cell wall structures [47]. The particles are proposed to release ionic Ag, likely triggering toxicity through membrane damage and facilitating the entry of excess Ag ions.

#### 3.2.5. Sulfur Metabolism Proteins

Within the subsystem inorganic nutrient metabolism, a part of ‘Other pathways’, which also includes processes such as macromolecule modification and activation/inactivation/interconversion (Figure 4e), one pathway was found to be affected by Ag exposure—sulfur metabolism. CysH—phosphor-adenylsulfate reductase is involved in assimilatory sulfate reduction by catalyzing the reduction of 3′-phospho-adenylylsulfate to sulfite and adenosine 3′,5-biphospahte (PAP). This protein contains highly conserved cysteine residues that become oxidized to form a disulfide bond [48]—possible targets based on the affinity of Ag for sulfur. Moreover, the *cysC*, *cysD* and *cysI* genes, also involved in the pathway sulfate reduction I (assimilatory) via phosphorylation, adenylation and reduction, respectively, were also Ag-resistant hits. These sulfate assimilatory proteins are linked to the Ag-resistant hit CysQ, which is involved in the recycling of PAP and has been experimentally determined to be the main target of lithium toxicity [49] (Supplementary Materials, Figure S2). The protein CysI, contains a siroheme and one [4Fe-4S] cluster per polypeptide chain [50]. Comparably, it has been demonstrated that the exposure of Ag nanoparticles upregulates the expression of several genes involved in iron and sulfate homeostasis [22], including those aforementioned. A decrease in the activity of this pathway reduces the amount of hydrogen sulfide required for processes such as l-cysteine biosynthesis, and since Ag interacts with sulfur compounds well, such as hydrogen sulfide—the final product of sulfate reduction I—fewer Ag targets may be available when genes of this pathway are deleted. CysH had the highest score of 0.360 out of all four sulfur assimilatory genes, and since this protein interacts with thioredoxin, the absence of CysH may free reduced thioredoxin, thus providing elevated resistance in presence of reactive oxygen species that may arise under Ag stress. 

#### 3.2.6. Biofilm Formation

In total 19 genes in the ‘Cellular processes’ system, which includes subsystems such as genetic transfer, quorum sensing, adhesion and locomotion, were found to confer resistance when absent (Figure 2). Three hits were involved in cell cycle and division, and two were found to be involved in biofilm formation (Figure 4g), such as CsgF, which is an outer membrane protein that initiates curli subunit polymerization, and therefore involved in the colonization of surfaces and biofilm formation [49]. In the absence of CsgF, less biofilm is formed, and according to our results, Ag resistance is generated. Biofilms commonly provide resistance in the face of fluctuating or threatening environments [51]; however, studies have shown that bacterial residence within a biofilm does not always provide enhanced resistance against metals [6,52,53], an observation supported by this work. An explanation for this may reside in the ability of biofilms to sequester Ag ions by attracting them to varying components of the extracellular polymeric matrix. While this may provide resistance, it may also concentrate ions within a localized area, thereby causing greater sensitivity. Similarly, Ag nanoparticles have been shown to inhibit *E. coli* biofilm formation by potentially targeting curli fibers [54], therefore the absence of curli fibers may promote Ag resistance. Previous studies, which have found that biofilm formation are a source of Ag resistance [32], were completed under differing culture conditions, therefore direct comparisons are challenging. 

#### 3.2.7. DNA Damage and Repair

The effect of Ag exposure on DNA damage and repair in *E. coli* has been inconsistent from several studies involving gene deletion strains. Radzig et al. showed that several deletion strains lacking in the ability to excise DNA bases were sensitive to Ag exposure, but not the *ΔrecA* strain, which is involved in SOS repair [36]. In contrast, the *ΔrecA* deletion strain showed Ag sensitivity in a previous study [32]. From our list of Ag resistant hits, six mutants were identified within the DNA damage subsystem (Figure 4h) including the *Δdam* strain. Dam is methyltransferase that functions in mismatch DNA repair in *E. coli* and may also play a role in controlling oxidative damage. Based on this protein’s function, we expect that the deletion strain of the gene would exhibit Ag sensitivity, potentially due to a deficiency in DNA repair of oxidative damage. However, the *dam1Δ* strain exhibits an upregulation of RecA and constitutive SOS activity which may be the nature of the Ag resistance exhibited in this mutant [36]. Moreover, we also identified several other Ag-resistant strains from our screens (*purF*, *damX*, *dcd*, *ruvC* and *ompA*) that are also known to possess RecA-mediated constitutive SOS activity [32].

### 3.3. Ag-Sensitive Hits

#### 3.3.1. Central Dogma and Cell Exterior Proteins

**Figure 5:**
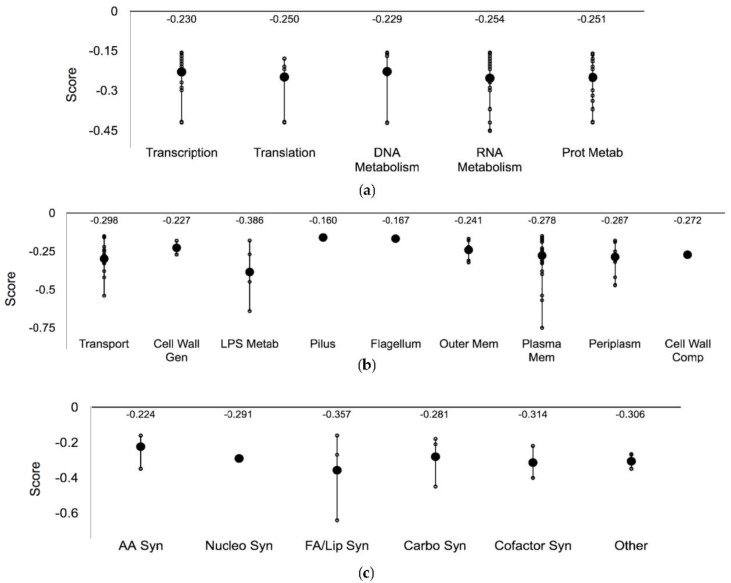

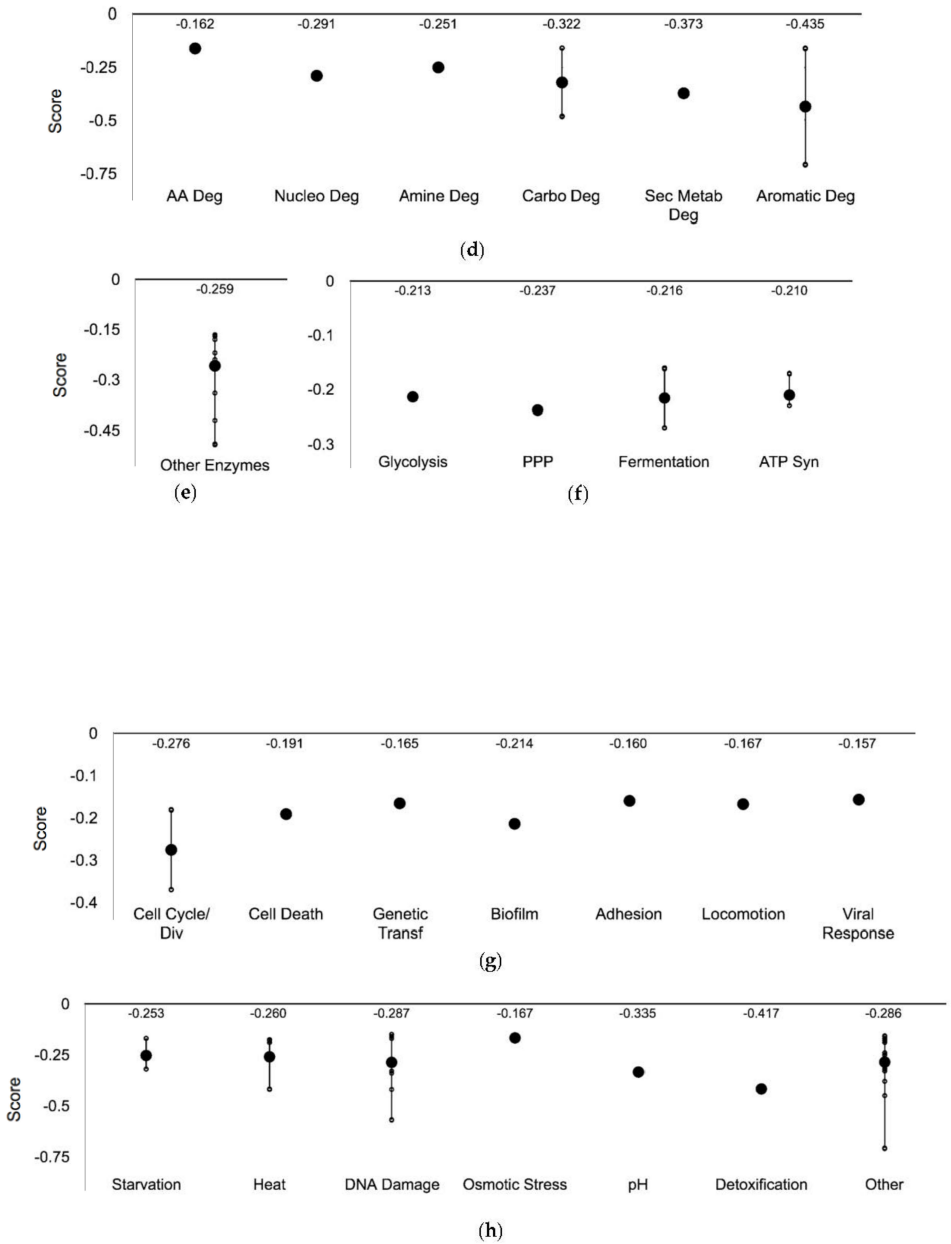
Ag-sensitive gene hits plotted against respective cellular processes. Y-axis representative of the normalized score, smaller circles represent the individual hits and the larger circles represent the mean of each subsystem. The *p*-value was a two-tailed *t*-test and significance was determined using the Benjamini-Hochberg procedure; false discovery rate was selected to be 0.1. Each individual score represents the mean of 12 trials. (**a**) Central Dogma; (**b**) Cell exterior; (**c**) Biosynthesis; (**d**) Degradation; (**e**) Other pathways; (**f**) Energy; (**g**) Cellular processes; and (**h**) Response to stimulus. Plots constructed using Pathway Tools, Omics Dashboard.

Within the ‘Central dogma’, 56 mutants resulted in Ag sensitivity (Figure 5a). For example, *ruvA*, a gene found to be involved in DNA repair, had a normalized score of −0.430 [55]. Direct DNA damage has not been attributed to Ag exposure; however, in the presence of reactive oxygen species potentially triggered by Ag exposure, the propagation of Fenton active iron may cause DNA damage [19,56].

In total of 73 genes were mapped to the system ‘Cell exterior’ (Figure 5b). The gene *ygiZ*, which codes for a putative inner membrane protein, had a score of −0.751, the lowest value of any protein in this screen. A common resistance mechanism employed by microbes is the export of the challenge from the periplasm or interior of the cell to the extracellular space [20]. The fold enrichment analysis supported this finding—cell membrane proteins and those involved in the ion transport were highly enriched (Figure 3). In total, 13 transport proteins conferred Ag sensitivity when absent, such as *cusB*, which encodes for a component of the copper/silver export system CusCFBA in *E. coli*, and contains several methionine residues important for function [57]. In the absence of this protein, sensitivity is anticipated, since the cell is unable to expel Ag ions. Another Ag sensitive hit was Lpp, considered to be the most abundant protein in *E*. *coli* [56]. Cells lacking Lpp have been found to be hypersensitive to toxic compounds [56], potentially because there is less protein available to sequester the incoming threat. In addition, the protein TolC was an Ag resistant hit. This protein is required for the function of a number of efflux systems including the AcrAB multidrug efflux system, which is involved in the export of a number of toxic exogenous compounds [58]. In contrast to efflux proteins, we identified the *cysA* and *cysP* genes—thiosulfate and sulfate permeases—to be sensitive hits when absent. CysA and CysP function in the first step of cysteine biosynthesis, which may be important in Ag resistance since this metal may target cysteine residues via thiol side chains [32].

#### 3.3.2. Lipopolysaccharide Biosynthetic Genes

In total, 18 Ag-sensitive hits were mapped to ‘Biosynthesis processes’ (Figure 5c). Processes associated with lipopolysaccharide biosynthesis were highly represented in the enrichment analysis (Figure 3). FabF, a key protein involved in fatty acid biosynthesis, and *clsB*—cardiolipin synthase B were found to be Ag-sensitive hits. If Ag targets the cellular membrane, lipid biosynthesis/regeneration could serve as a mechanism of Ag resistance and consequently, Ag toxicity would be increased if either of these processes were compromised *via* the deletion of these candidate genes. 

Processes of biomolecule degradation were affected to a lesser degree than biosynthesis (Figure 2). Only nine hits were mapped to this system (Figure 5d). The mutant *hcaD* had the second lowest score of −0.707 in this screen. This protein is a predicted ferredoxin reductase subunit that is involved in the degradation of aromatic acids as carbon sources. 

#### 3.3.3. Three Ag-Sensitive Hits Comprise the ATP Synthase F_o_ Complex

Seven hits were mapped to ‘Energy processes’ (Figure 5f). Of these, three are components of the ATP synthase F_o_ complex—AtpB, AtpE and AtpF. Ag has been suggested to damage the respiratory chain of *E*. *coli* [59], thereby preventing the efficient pumping of protons across the membrane. Small disruptions to the F_o_ complex may amplify this consequence and render this biological process hypersensitive. If this mechanism is correct and the cytoplasmic membrane becomes more permeable to protons, than the cell will attempt to compensate for this increase in acidity via several mechanisms, one being the reversal of ATP synthase in order to pump protons outward (if ATP is not limiting) and decrease cytoplasmic proton concentrations [60]. If the ATP synthase complex exhibits decreased activity due to disruptions in any of the subunits, this resistant mechanism may be unable to function properly, resulting in greater Ag sensitivity.

Several nodes of interaction based on the STRING connectivity maps were made evident within this cluster of proteins such as the association of *atpF* and *atpB* to *gshB* and several putative membrane proteins, *tufB*—elongation factor Tu and *ppgL*—a putative zinc peptidase (Supplementary Materials, Figure S3). 

#### 3.3.4. Oxidative Stress Response Genes

Out of the 31 proteins mapped to ‘Response to Stimulus’, 24 were involved in mediating DNA damage and other processes (Figure 5h). The gene coding for glutathione synthetase—*gshB* was found to be an Ag-sensitive hit. Strains overexpressing either GshA or GshB are more resistant to oxidative damage, and this system has been shown to mediate metal resistance [61]. As a result, the deletion of either gene is anticipated to cause Ag sensitivity. Furthermore, the putative Fe^+2^ trafficking protein, YggX was found to have a score of −0.450. This protein is proposed to play a role in preventing the oxidation of iron-sulfur clusters [62], a proposed mechanism of Ag toxicity. The absence of this protective protein may result in sensitivity since it can be found at elevated concentrations in vivo and it is involved in mediating oxidative damage [63]. Further, the protein Hmp, a flavohemoglobin with nitric oxide dioxygenase activity [64] had a score of −0.254. This protein has been shown to protect respiratory cytochromes in *E*. *coli* [37], which is a possible mechanism of Ag toxicity [59,65]. 

## 4. Conclusions

In this work, a chemical genetic screen of a mutant library was performed as a means of drawing insight into the mechanisms of Ag toxicity and resistance in bacteria. In total, 3810 mutant strains containing single deletions of non-essential genes in *E. coli* were screened, and subsequent hits were bioinformatically evaluated in order to highlight processes and pathways that are affected by Ag exposure. This systematic mutant screen involved a low but prolonged concentration of Ag exposure on solid minimal media to avoid indirect secondary and acute responses, while also attempting to directly target relevant genes. Here, resistant hits represented genes involved in enhancing the cytotoxicity of Ag, while in contrast sensitive hits represented genes functioning in tolerance to Ag including physiological responses that mitigate toxicity.

In short, processes involved with the cell exterior and the central dogma were found to be affected by Ag exposure to a greater extent than other processes analyzed. However, when further examining the fold enrichment, the cell membrane and transport were involved in Ag exposure to a lesser degree. In fact, proteins involved in amino acid biosynthesis (Ag sensitivity), phosphoproteins and metalloproteins (Ag resistance) were most densely represented as hits in this work—trends that were supported by the protein-protein interaction networks.

Our work supports many previously proposed mechanisms of Ag toxicity—disruption of iron-sulfur cluster containing proteins and certain cellular redox enzymes, and DNA damage, and Ag resistance—toxin export and sequestration. However, the data presented here also demonstrates that the activity of Ag within the bacterial cell is more extensive than previously suggested, involving genes a part of the cell wall structure, quinone metabolism, ATP synthesis and sulfur reduction.

The use of Ag as an antimicrobial is a practice garnering considerable popularity, as the introduction of Ag-based compounds, such as combination treatments, nanomaterials, and formulations make way. In order to continue the development of this metal as a therapeutic agent, it is imperative that we gather more understanding into the accompanying mechanisms of Ag toxicity and resistance. This study provides a vast number of biomolecular mechanistic hypotheses to the community investigating the mechanisms of action of Ag and other metals. 

## Figures and Tables

**Table 1:** Ag-resistant hits organized according to system and subsystem mined using the Omics Dashboard (Pathway Tools), which surveys against the EcoCyc Database; genes represent resistant hits, each with a score >0.15 and a false discovery rate of 0.1 ^1,2^.

System	Subsystem	Gene ^3^
Central Dogma	Transcription	*alaS crp dicC gadE* *gcvR lysR putA* *yciT yhjB yiif yjiR*
	Translation	*alaS ettA*
	DNA Metabolism	*cffC dam recT*
	RNA Metabolism	*rluF alaS gluQ trmL* *crp dicC gadE gcvR* *lysR ogrK putA yciT* *yhjB yiif yjiR yjtD*
	Protein Metabolism	*argE envZ lipB sdhE* *ldcA pepB prc* *rhsB rzpD*
Cell Exterior	Transport	*malE nhaB exbB btuB* *dppF glcA ompG lptB* *mngA yejF*
	Cell wall biogenesis/organization	*idcA*
	Lipopolysaccharide Metabolism	*wcaI*
	Pilus	*yraK*
	Flagellum	*fliL fliR*
	Outer membrane	*bbtuB csgF nlpE ompA* *ompG rhsB*
	Plasma membrane	*agaD cyoC cysQ damX* *dppF envZ ettA exbB* *fliL fliR glcA IptB* *malE mngA nhaB ppx* *prc putA yaiP yccF* *yejF ygdD yifK* *yojI yqfA*
	Periplasm	*malE nlpE prc*
Biosynthesis	Amino acid biosynthesis	*argE cysk serC proC* *serA serC metL* *trpB trpD*
	Nucleotide biosynthesis	*dcd pyrF*
	Amine biosynthesis	*gss*
	Carbohydrate biosynthesis	*mdh*
	Secondary metabolite biosynthesis	*fldB*
	Cofactor biosynthesis	*bioC bioF nudB lipB* *nadA nadB nadC gss* *thiS serC*
	Other	*aroC metL argE alaS*
Degradation	Amino acid degradation	*astA cysK gadA putA*
	Carbohydrate degradation	*galM yigL glcE*
	Secondary metabolite degradation	*idcA*
	Polymer degradation	*idcA*
Other pathways	Inorganic nutrient metabolism	*cysC cysD cysH cysI*
	Detoxification	*gadA sodA*
	Activation/inactivation/interconversion	*cysC cysD*
	Other	*ahpF bglB cysQ dam* *gluQ pepB ppx prc* *purU rluF trmL* *yfaU yjhG*
Energy	TCA cycle	*mdh*
	Fermentation	*mdh*
	Aerobic respiration	*cyoC putA*
	Other	*bioC bioF mdh*
Cellular process	Cell cycle/Division	*dam damX dicC*
	Cell death	*ldcA*
	Genetic transfer	*ompA ygcO*
	Biofilm formation	*csgF*
	Adhesion	*yraK*
	Locomotion	*fliL malE rzpD*
	Viral response	*ompA rzpD*
	Bacterial response	*rzpD*
	Host interaction	*ompA rzpD*
Response to stimulus	Heat	*sodA*
	DNA damage	*dam malE ompA recT* *yaiP yciT*
	pH	*sodA*
	Oxidant detoxification	*sodA*
	Other	*ahpF btuB crp cysC* *cysD cysH cysI dcd* *dppF envZ exbB fliL* *nhaB prc putA recT* *rzpD ybaM yejF* *yigL yojI*

^1^ Each individual score represents the mean of 12 trials—three biological and four technical; ^2^ Two-tailed *t*-test and significance was determined using the Benjamini-Hochberg procedure; ^3^ Gene hits can be mapped to more than one system and subsystem.

**Table 2:** Ag-sensitive hits organized according to system and subsystem mined using the Omics Dashboard (Pathway Tools), which surveys against the EcoCyc Database; genes represent resistant hits, each with a score <−0.15 and a false discovery rate of 0.1 ^1,2^.

System	Subsystem	Gene ^3^
Central Dogma	Transcription	*arcB exuR fis galR* *glnL higB hupB rapA* *rfaH sspA rhoL* *ybeY yfjR*
	Translation	*higB prfC rhaH rplI* *tufB ybeY*
	DNA Metabolism	*fis hsdS hofM* *ruvA mutL*
	RNA Metabolism	*arcB exuR fis galR* *glnL higB hupB rapA* *rfah rhoL rsmE rraB* *sspA ybeY yfjR ygfZ*
	Protein Metabolism	*arcB glnL higB hybD* *iadA mobA pflA prfC* *pqqL rfaH rplI tufB* *ybeY ygeY yicR*
Cell Exterior	Transport	*chbB clcA cusB cysA* *cysP dtpB fepA feoB* *tdcC tolC trkH* *tyrP yiaN*
	Cell wall biogenesis/organization	*amiB rfe*
	Lipopolysaccharide metabolism	*kdsD rfaD rfe waaG*
	Pilus	*yfcQ*
	Flagellum	*flgH*
	Outer membrane	*fepA flgH lpp tolC yraP*
	Plasma membrane	*arcB atpB atpE atpF* *bcsF clcA clcB cstA* *cysA dtpB feoB glnL* *glvB hokD hycB ppdB* *rfe sanA tdcC tolC* *trkH tufB tyrP ydcV* *ydjZ ygeY ygiZ yhaH* *yhjD yiaB yiaN yibN* *yjiG yqiJ*
	Periplasm	*amiB cusB cysP hmp* *lpp sanA tolC yfdX* *yjfY yraP ytfJ*
	Cell wall components	*rfe*
Biosynthesis	Amino acid biosynthesis	*hisA ilvG lysC*
	Nucleotide biosynthesis	*add*
	Fatty acid and lipid biosynthesis	*fabF wag clsB*
	Carbohydrate biosynthesis	*yggF rfaD kdsD*
	Cofactor biosynthesis	*mobA ubiE gshB*
	Other	*aroL lysC*
Degradation	Amino acid degradation	*ilvG pflB*
	Nucleotide degradation	*add*
	Amine degradation	*caiC*
	Carbohydrate degradation	*yidA ulaG*
	Secondary metabolite degradation	*lsrF*
	Aromatic degradation	*hcaD mhpC*
Other pathways	Other	*amiB higB hmp hsdS* *iadA mutL nfsB nudF* *pflA qorB rsmE ruvA*
Energy	Glycolysis	*yggF*
	Pentose phosphate pathway	*rpiA*
	Fermentation	*hycB pflB*
	ATP synthesis	*atpB atpE atpF*
Cellular processes	Cell cycle and division	*amiB minC*
	Cell death	*hokD*
	Genetic transfer	*ydcV*
	Biofilm formation	*yfjR*
	Adhesion	*yfcQ*
	Locomotion	*flgH*
	Viral Response	*fis*
Response to Stimulus	Starvation	*cstA sanA sspA*
	Heat	*Nudf ybeY yobF*
	DNA damage	*add feoB hisA mutL* *pflA ruvA ybiX* *yiaB yqiJ*
	Osmotic stress	*flgH*
	pH	*clcA*
	Detoxification	*cusB*
	Other	*arcB cstA dtpB fis* *glnL hcaD hmp hsdS* *mhpC sanA sspA tolC* *tufB yfdS yggX*

^1^ Each individual score represents the mean of 12 trials—three biological and four technical; ^2^ Two-tailed *t*-test and significance was determined using the Benjamini-Hochberg procedure; ^3^ Gene hits can be mapped to more than one system and subsystem.

## Supplementary Materials

The following are available online at http://www.mdpi.com/2073-4425/9/7/344/s1, Figure S1: Resistant and sensitive hits involved in regulation; Figure S2: Connectivity map displaying silver resistant hits; Figure S3: Connectivity map displaying silver sensitive hits; Figure S4: Silver resistant metabolic overview; Figure S5: Silver sensitive metabolic overview; Table S1: Normalized and scored gene list.

## Appendix A

**Table A1:** Systems and comprising subsystems cited in this study. The Ag resistant and sensitive hits were surveyed against the EcoCyc database permitting the clustering of the hits into systems, subsystems, component subsystems, and lastly into individual objects ^1,2^.

Systems	Subsystems
Regulation	Signaling, sigma factor regulon, transcription factor, and transcription factor regulons
Response to Stimulus	Starvation, heat, cold, DNA damage, pH, detoxification, osmotic stress, and other
Cellular processes	Cell cycle and division, cell death, genetic transfer, biofilm formation, quorum sensing, adhesion, locomotion, viral response, response to bacterium, host interactions with host, other pathogenesis proteins
Energy	Glycolysis, the pentose phosphate pathway, the TCA cycle, fermentation, and aerobic and anaerobic respiration
Other pathways	Detoxification, inorganic nutrient metabolism, macromolecule modification, activation/inactivation/interconversion, and other enzymes
Degradation	Amino acids, nucleotide, amine, carbohydrate/carboxylate, secondary metabolite, alcohol, polymer and aromatic, the cell exterior, and regulation
Biosynthesis	Amino acids, nucleotides, fatty acid/lipid amines, carbohydrate/carboxylates, cofactors, secondary metabolites, and other pathways
Cell exterior	Transport, cell wall biogenesis and organization, lipopolysaccharide metabolism, pilus, flagellar, outer and inner membrane, periplasm, and cell wall components
Central Dogma	Transcription, translation, DNA metabolism, RNA metabolism, protein metabolism and protein folding and secretion

^1^ Each individual score represents the mean of 12 trials—three biological and four technical; ^2^ Two-tailed *t*-test and significance was determined using the Benjamini-Hochberg procedure.
