# Supplementary material for: Using a Chemical Genetic Screen to Enhance Our Understanding of the Antibacterial Properties of Silver
Source: Genes (Basel). 2018 Jul 6;9(7):344. doi: 10.3390/genes9070344 (PMC6071238; doi:10.3390/genes9070344)
Supplement: Supplementary file 1 [file genes-09-00344-s001.pdf]

Supplementary information

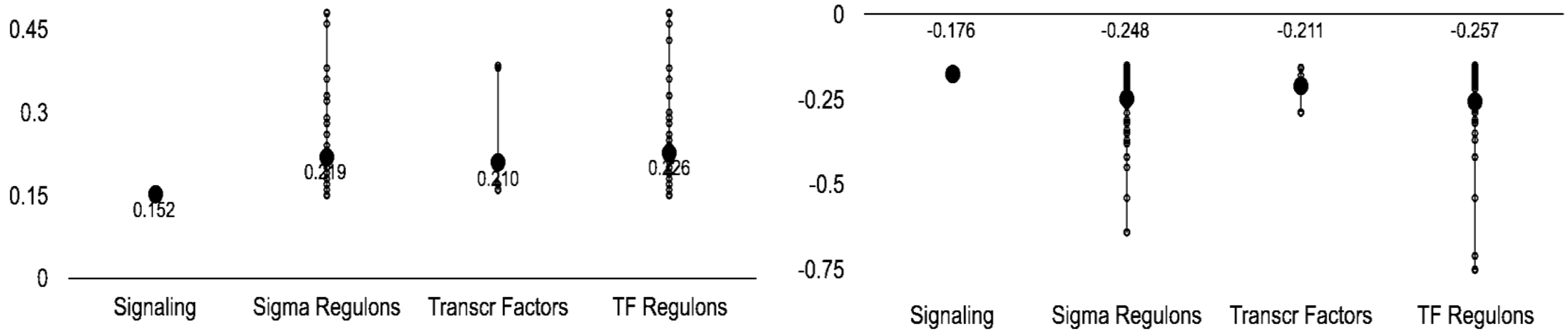

(**a**) (**b**)

Figure S1. Resistant (**a**) and sensitive (**b**) gene scores plotted against subsystems involved in cell regulation. The small circles represent the individual hits and the large circles represent the mean of each subsystem. Each individual score signifies the mean of 12 trials – three biological and four technical. The *p*-value was calculated as a two-tailed *t*-test and significance was determined using the Benjamini-Hochberg procedure; false discovery rate was selected to be 0.1. Plots constructed using Pathway Tools, Omics Dashboard.

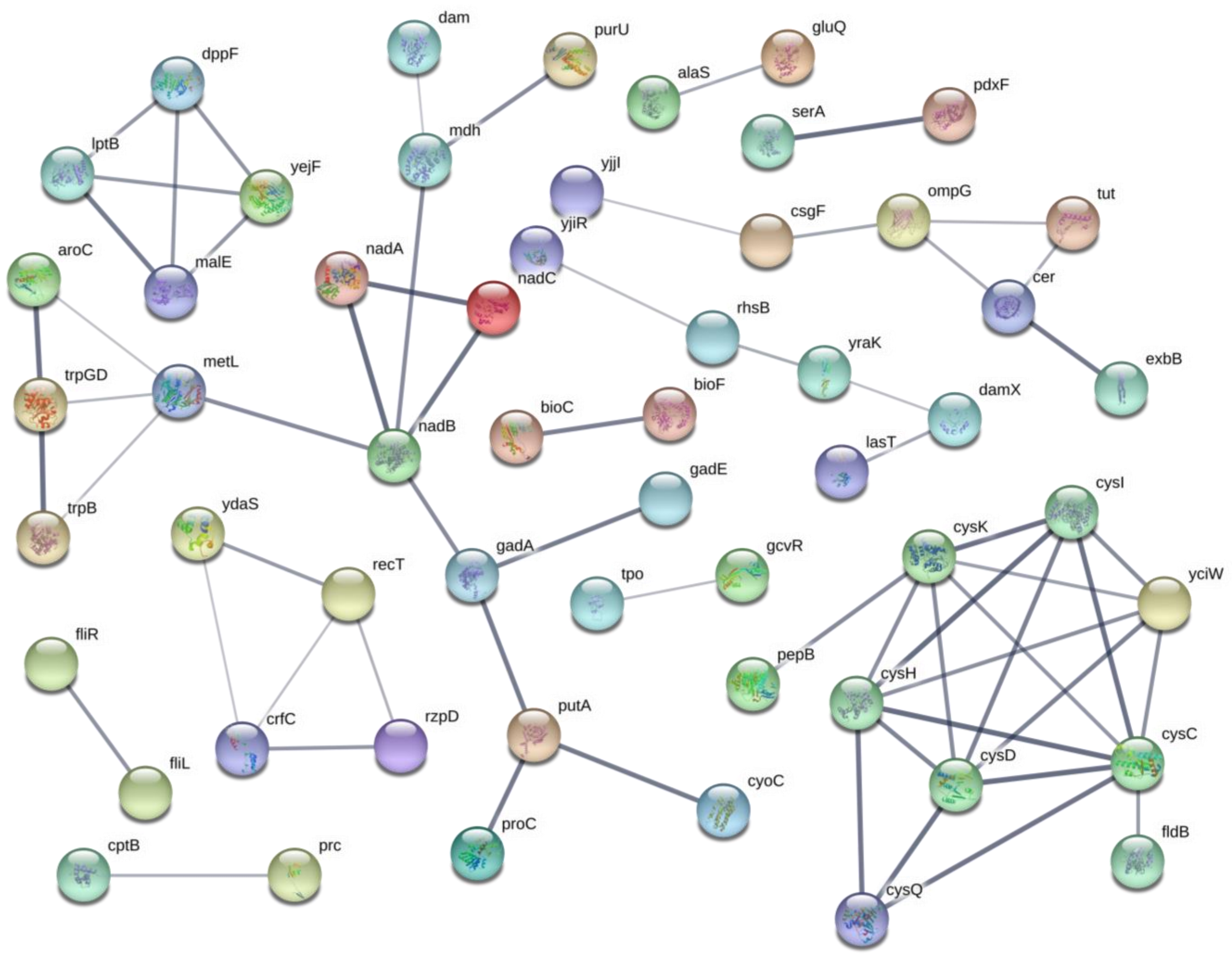

Figure S2. Connectivity map displaying the predicted functional associations between the silver-resistant gene hits; disconnected gene hits not shown. The thicknesses of the lines indicate the degree of confidence prediction for the given interaction, based on fusion, co-occurrence, experimental and co-expression data. Figure produced using STRING (version 10.5) and a medium confidence score (approximate probability) of 0.4.

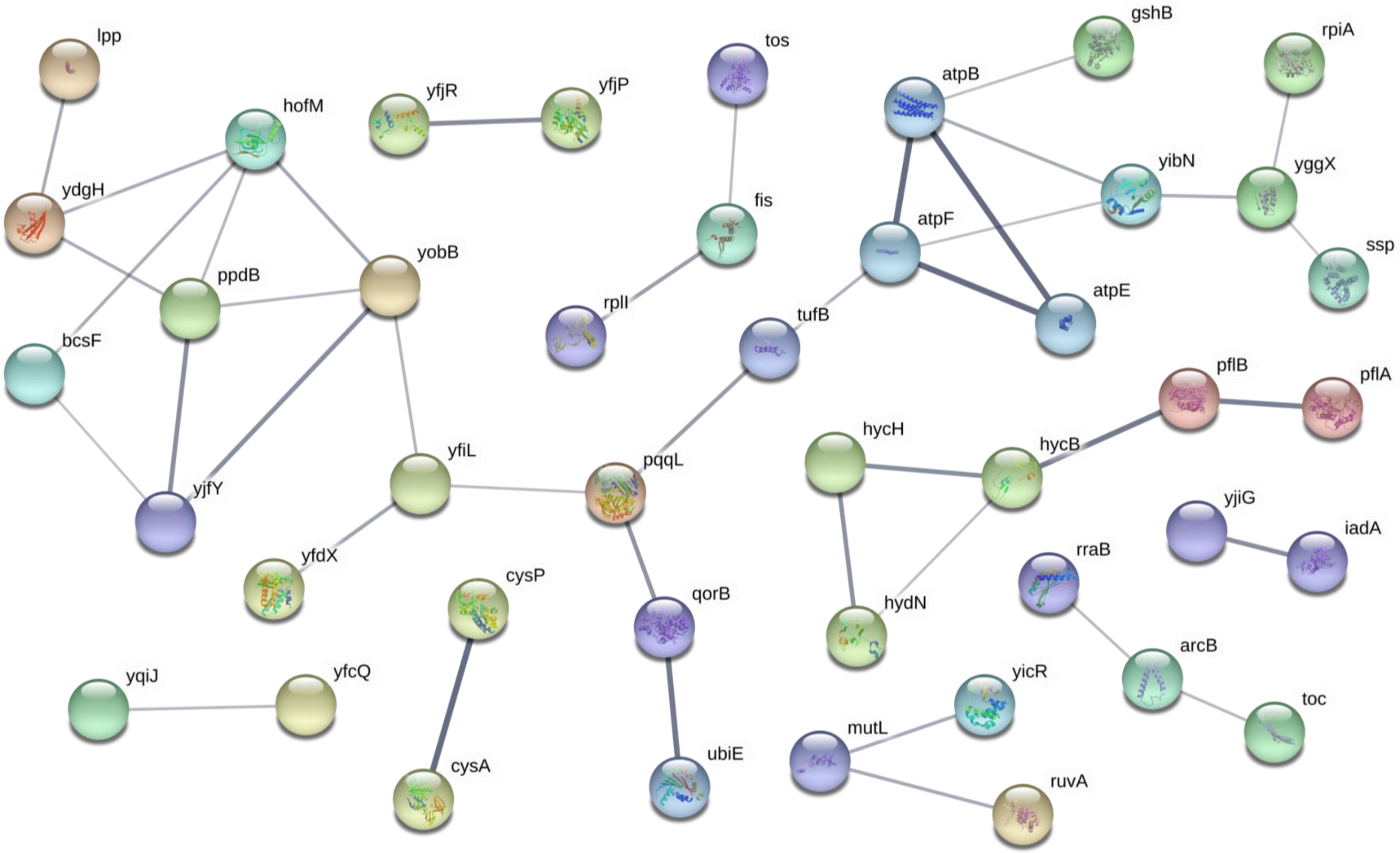

Figure S3. Connectivity map displaying the predicted functional associations between the silver-sensitive gene hits; disconnected gene hits not shown. The thicknesses of the lines indicate the degree of confidence prediction for the given interaction, based on fusion, co-occurrence, experimental and co-expression data. Figure produced using STRING (version 10.5) and a medium confidence score (approximate probability) of 0.4.

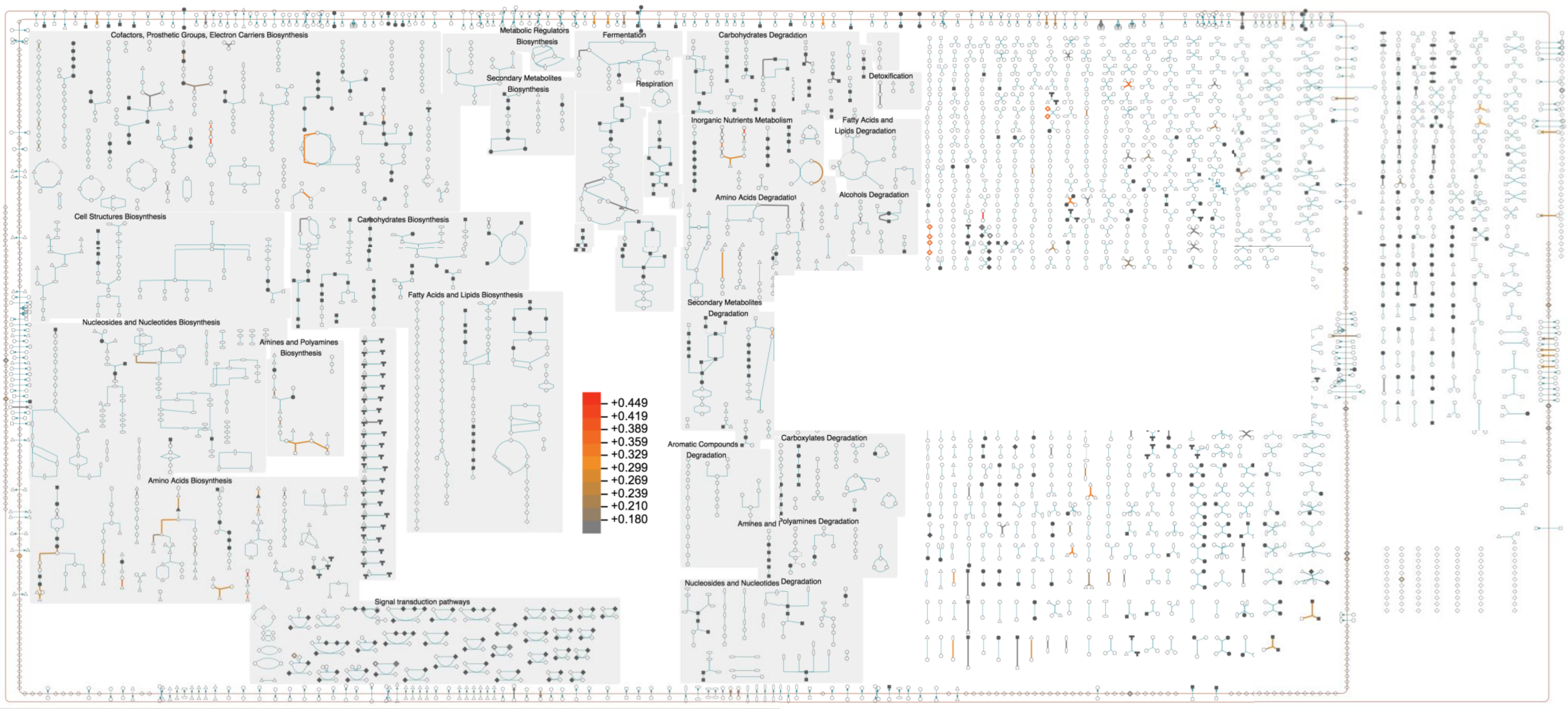

Figure S4. Metabolic overview of the pathways in *Escherichia coli*. The pathways involved in silver-resistance are coloured according to respective normalized score. Each individual score represents the mean of 12 trials – three biological and four technical. Amino acid – upward pointing triangle, carbohydrate – square, proteins – diamond, purines – vertical ellipse, cofactor – downward pointing triangle, tRNA – tee, and other – circle. Grey filled shapes represent phosphorylated metabolites.

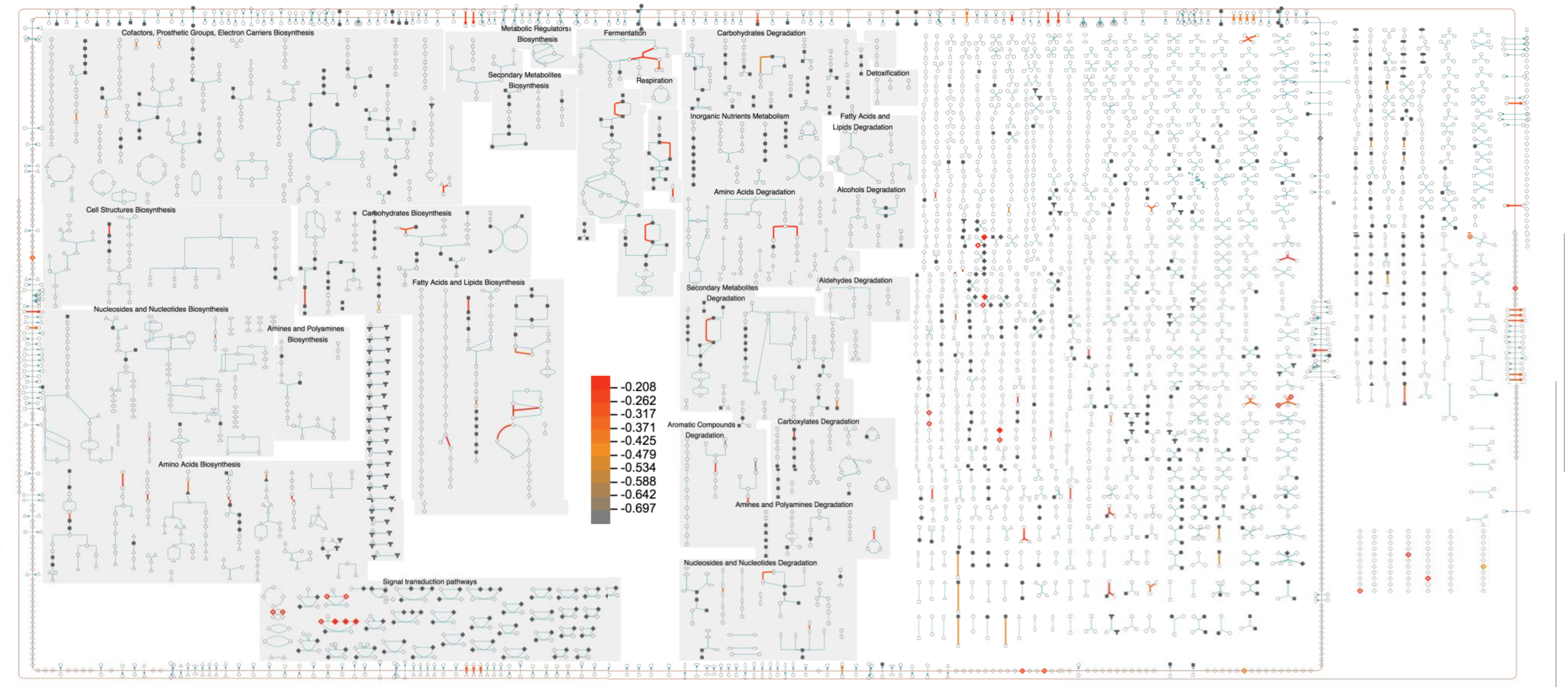

Figure S5: Metabolic overview of the pathways in *Escherichia coli*. The pathways involved in silver-sensitivity are coloured according to respective normalized score. Each individual score represents the mean of 12 trials – three biological and four technical. Amino acid – upward pointing triangle, carbohydrate – square, proteins – diamond, purines – vertical ellipse, cofactor – downward pointing triangle, tRNA – tee, and other – circle. Coloured shapes represent phosphorylated metabolites.

Table S1. Synthetic Array Tools (version 1.0) was used to normalize and score the silver-resistant and -sensitive gene hits as a means of representing the growth differences in *Escherichia coli* K12 BW25113 in the presence of 100μM silver nitrate. Genes with a normalized score <0 are implied as silver-sensitive and those with a score of >0 are silver-resistant hits.

ID	Name	Function[1]	Score[2]	P-value[3]
JW0001	thrA	bifunctional aspartokinase/homoserine dehydrogenase 1	-0.0534	0.2569
JW0002	thrB	homoserine kinase	-0.0004	0.9944
JW0002	thrB	homoserine kinase	0.2060	0.0897
JW0003	thrC	L-threonine synthase	0.1294	0.0041
JW0004	yaaX	DUF2502 family putative periplasmic protein	0.0001	0.9941
JW0005	yaaA	peroxide resistance protein, lowers intracellular iron	0.0270	0.0685
JW0006	yaaJ	putative transporter	0.0901	0.0002
JW0007	talB	transaldolase B	-0.1347	0.0009
JW0008	mog	molybdochelatase incorporating molybdenum into molybdopterin	-0.0590	0.0002
JW0009	yaaH	succinate-acetate transporter	0.0493	0.1511
JW0010	yaaW	UPF0174 family protein	0.0270	0.5107
JW0012	yaaI	UPF0412 family protein	0.0007	0.6916
JW0013	dnaK	chaperone Hsp70, with co-chaperone DnaJ	0.0990	0.0028
JW0014	dnaJ	chaperone Hsp40, DnaK co-chaperone	-0.1171	0.0037
JW0018	nhaA	sodium-proton antiporter	-0.0472	0.2963
JW0019	nhaR	transcriptional activator of nhaA	-0.0016	0.9484
JW0022	rpsT	30S ribosomal subunit protein S20	-0.0188	0.5675
JW0024	ileS	isoleucyl-tRNA synthetase	0.0301	0.4161
JW0026	fkpB	FKBP-type peptidyl-prolyl cis-trans isomerase (rotamase)	-0.0763	0.4062
JW0028	rihC	ribonucleoside hydrolase 3	0.0220	0.1171
JW0030	carA	carbamoyl phosphate synthetase small subunit, glutamine amidotransferase	-0.0749	0.0615
JW0031	carB	carbamoyl-phosphate synthase large subunit	0.0024	0.9649
JW0033	caiF	cai operon transcriptional activator	0.0552	0.2974
JW0035	caiD	carnitinyl-CoA dehydratase	0.0320	0.1290
JW0036	caiC	putative crotonobetaine/carnitine-CoA ligase	-0.2512	0.0002
JW0037	caiB	crotonobetainyl CoA:carnitine CoA transferase	-0.0480	0.1644
JW0038	caiA	crotonobetaine reductase subunit II, FAD-binding	-0.0579	0.1935
JW0039	caiT	putative transporter	0.0146	0.8119
JW0040	fixA	anaerobic carnitine reduction putative electron transfer flavoprotein subunit	0.0458	0.0029
JW0041	fixB	putative electron transfer flavoprotein, NAD/FAD-binding domain and ETFP	0.0915	0.0037

		adenine nucleotide-binding domain-like protein		
JW0042	fixC	putative oxidoreductase	-0.0490	0.0303
JW0043	fixX	putative 4Fe-4S ferredoxin-type protein	0.0065	0.8522
JW0044	yaaU	putative MFS sugar transporter; membrane protein	0.1737	0.2868
JW0045	kefF	potassium-efflux system ancillary protein for KefC, glutathione-regulated; quinone oxidoreductase, FMN-dependent	-0.0352	0.3635
JW0046	kefC	potassium:proton antiporter	-0.0163	0.5461
JW0048	apaH	diadenosine tetraphosphatase	-0.1556	0.2481
JW0049	apaG	protein associated with Co2+ and Mg2+ efflux	0.0098	0.8831
JW0050	ksgA	16S rRNA m(6)A1518, m(6)A1519 dimethyltransferase, SAM-dependent	0.0648	0.0128
JW0051	pdxA	4-hydroxy-L-threonine phosphate dehydrogenase, NAD-dependent	0.2949	0.0557
JW0052	surA	peptidyl-prolyl cis-trans isomerase (PPIase)	0.0420	0.1053
JW0054	djlA	membrane-anchored DnaK co-chaperone, DNA-binding protein	0.0149	0.3970
JW0055	yabP	pseudogene, pentapeptide repeats-containing	-0.0133	0.6481
JW0057	rluA	dual specificity 23S rRNA pseudouridine(746), tRNA pseudouridine(32) synthase, SAM-dependent	-0.0156	0.5632
JW0058	hepA	RNA polymerase remodeling/recycling factor ATPase; RNA polymerase-associated, ATP-dependent RNA translocase	-0.1693	0.0063
JW0059	polB	DNA polymerase II	0.0069	0.8212
JW0063	araC	ara regulon transcriptional activator; autorepressor	-0.1094	0.0056
JW0065	thiQ	thiamine/thiamine pyrophosphate ABC transporter ATPase	-0.0575	0.0012
JW0066	thiP	thiamine/thiamine pyrophosphate ABC transporter permease	-0.0545	0.0856
JW0067	tbpA	thiamine/thiamine pyrophosphate/thiamine monophosphate ABC transporter periplasmic binding protein	-0.0160	0.5373
JW0068	sgrR	transcriptional DNA-binding transcriptional activator of sgrS sRNA	-0.0487	0.0562
JW0069	setA	broad specificity sugar efflux system	-0.1094	0.3620
JW0070	leuD	3-isopropylmalate dehydratase small subunit	0.0129	0.8362

JW0071	leuC	3-isopropylmalate dehydratase large subunit	0.1346	0.0024
JW0073	leuA	2-isopropylmalate synthase	-0.0199	0.5889
JW0074	leuL	leu operon leader peptide	0.0472	0.0718
JW0075	leuO	global transcription factor	-0.1227	0.0001
JW0076	ilvI	acetolactate synthase 3 large subunit	-0.0103	0.6806
JW0077	ilvH	acetolactate synthase 3, small subunit, valine-sensitive	-0.0073	0.7893
JW0078	fruR	transcriptional repressor-activator for carbon metabolism	0.0608	0.0029
JW0079	mraZ	RsmH methytransferase inhibitor	-0.0571	0.0421
JW0080	mraW	16S rRNA m(4)C1402 methyltransferase, SAM-dependent	0.0118	0.5659
JW0090	ddlB	D-alanine:D-alanine ligase	0.1386	0.0002
JW0097	mutT	dGTP-preferring nucleoside triphosphate pyrophosphohydrolase	-0.1218	0.3731
JW0099	yacF	FtsZ stabilizer	0.0615	0.0567
JW0100	coaE	dephospho-CoA kinase	-0.0409	0.0817
JW0101	guaC	GMP reductase	-0.1751	0.0434
JW0102	hofC	assembly protein in type IV pilin biogenesis, transmembrane protein	-0.0395	0.1599
JW0103	hofB	T2SE secretion family protein; P-loop ATPase superfamily protein	0.0946	0.4871
JW0104	ppdD	putative prepilin peptidase-dependent pilin	0.0023	0.9355
JW0105	nadC	quinolinate phosphoribosyltransferase	0.4257	0.0000
JW0106	ampD	1,6-anhydro-N-acetylmuramyl-L-alanine amidase, Zn-dependent; murein amidase	-0.0929	0.0752
JW0107	ampE	ampicillin resistance inner membrane protein; putative signaling protein in beta-lactamase regulation	0.1436	0.0137
JW0108	aroP	aromatic amino acid transporter	-0.3680	0.0491
JW0109	pdhR	pyruvate dehydrogenase complex repressor; autorepressor	0.0247	0.5738
JW0110	aceE	pyruvate dehydrogenase, decarboxylase component E1, thiamine triphosphate-binding	0.0364	0.2135
JW0111	aceF	pyruvate dehydrogenase, dihydrolipoyltransacetylase component E2	-0.0775	0.0140
JW0112	lpd	dihydrolipoyl dehydrogenase; E3 component of pyruvate and 2-oxoglutarate dehydrogenases complexes; glycine cleavage system L protein; dihydrolipoamide dehydrogenase	0.0103	0.5847
JW0113	yacH	DUF3300 family protein	-0.0650	0.0010
JW0114	acnB	aconitate hydratase 2; aconitase B; 2-methyl-cis-aconitate hydratase	0.0361	0.0767
JW0115	yacL	UPF0231 family protein	-0.0922	0.0144

JW0116	speD	S-adenosylmethionine decarboxylase	-0.1070	0.0060
JW0117	speE	spermidine synthase (putrescine aminopropyltransferase)	0.2887	0.0469
JW0118	yacC	PulS_OutS family protein	0.0084	0.6483
JW0119	cueO	multicopper oxidase (laccase)	0.0047	0.9004
JW0120	gcd	glucose dehydrogenase	-0.0185	0.3599
JW0123	yadG	putative ABC transporter ATPase	-0.1331	0.0230
JW0124	yadH	putative ABC transporter permease	-0.1005	0.0054
JW0125	yadI	putative PTS Enzyme IIA	-0.0348	0.0541
JW0126	yadE	putative polysaccharide deacetylase lipoprotein	-0.0887	0.0027
JW0127	panD	aspartate 1-decarboxylase	-0.0101	0.6568
JW0129	panC	pantothenate synthetase	-0.0502	0.1273
JW0130	panB	3-methyl-2-oxobutanoate hydroxymethyltransferase	0.0583	0.3930
JW0131	yadC	putative fimbrial-like adhesin protein	-0.0327	0.1033
JW0132	yadK	putative fimbrial-like adhesin protein	0.0367	0.0809
JW0133	yadL	putative fimbrial-like adhesin protein	-0.0162	0.6796
JW0134	yadM	putative fimbrial-like adhesin protein	-0.0753	0.0018
JW0135	htrE	putative outer membrane usher protein	0.0501	0.3654
JW0136	ecpD	putative periplasmic pilin chaperone	-0.0507	0.0669
JW0137	yadN	putative fimbrial-like adhesin protein	-0.0434	0.0073
JW0141	dksA	transcriptional regulator of rRNA transcription; DnaK suppressor protein	0.2042	0.0000
JW0142	sfsA	sugar fermentation stimulation protein A	-0.0038	0.8051
JW0144	hrpB	putative ATP-dependent helicase	0.0979	0.0013
JW0145	mrcB	fused glycosyl transferase and transpeptidase	0.0531	0.2187
JW0146	fhuA	ferrichrome outer membrane transporter	0.0975	0.0717
JW0147	fhuC	iron(3+)-hydroxamate import ABC transporter ATPase	0.0345	0.5803
JW0148	fhuD	iron(3+)-hydroxamate import ABC transporter periplasmic binding protein	0.1716	0.0932
JW0149	fhuB	iron(3+)-hydroxamate import ABC transporter permease	-0.0543	0.0197
JW0153	yadS	UPF0126 family inner membrane protein	-0.0261	0.3485
JW0154	btuF	vitamin B12 ABC transporter periplasmic binding protein	0.2510	0.0302
JW0155	pfs	5'-methylthioadenosine/S-adenosylhomocysteine nucleosidase	-0.1135	0.0708
JW0156	dgt	deoxyguanosine triphosphate triphosphohydrolase	0.0520	0.2579
JW0157	degP	serine endoprotease (protease Do), membrane-associated	0.0178	0.6798
JW0159	yaeH	UPF0325 family protein	0.0037	0.9642
JW0162	glnD	uridylyltransferase	-0.0338	0.4051
JW0173	hlpA	periplasmic chaperone	-0.0014	0.9697
JW0178	rnhB	ribonuclease HII, degrades RNA of DNA-RNA hybrids	0.1380	0.0445

JW0181	ldcC	lysine decarboxylase 2, constitutive	0.0670	0.1069
JW0182	yaeR	putative lyase	0.0754	0.0081
JW0184	rof	modulator of Rho-dependent transcription termination	0.1987	0.0240
JW0185	yaeP	UPF0253 family protein	-0.0043	0.8365
JW0186	yaeQ	PDDEXK superfamily protein	-0.0213	0.6030
JW0187	yaeJ	alternative stalled-ribosome rescue factor B; peptidyl-tRNA hydrolase, ribosome-attached	0.0563	0.0672
JW0188	nlpE	lipoprotein involved with copper homeostasis and adhesion	0.1925	0.0001
JW0191	yaeB	tRNA-Thr(GGU) m(6)t(6)A37 methyltransferase, SAM-dependent	0.0251	0.0644
JW0192	rcsF	putative outer membrane protein	0.2090	0.0274
JW0193	metQ	DL-methionine transporter subunit	-0.0182	0.4503
JW0194	metI	DL-methionine transporter subunit	0.0794	0.0550
JW0195	metN	DL-methionine transporter subunit	-0.0252	0.1474
JW0196	gmhB	D,D-heptose 1,7-bisphosphate phosphatase	-0.0254	0.2336
JW0197	dkgB	2,5-diketo-D-gluconate reductase B	0.0255	0.3856
JW0198	yafC	LysR family putative transcriptional regulator	0.0729	0.0198
JW0200	yafE	putative S-adenosyl-L-methionine-dependent methyltransferase	0.0264	0.0283
JW0202	gloB	hydroxyacylglutathione hydrolase	0.0063	0.9167
JW0203	yafS	putative S-adenosyl-L-methionine-dependent methyltransferase	0.0942	0.0088
JW0204	rnhA	ribonuclease HI, degrades RNA of DNA-RNA hybrids	-0.0282	0.4103
JW0205	dnaQ	DNA polymerase III epsilon subunit	0.0311	0.4572
JW0206	yafT	lipoprotein	-0.0481	0.3376
JW0207	yafU	pseudogene	-0.0210	0.1397
JW0210	ivy	inhibitor of c-type lysozyme, periplasmic	-0.0022	0.0624
JW0212	lpcA	D-sedoheptulose 7-phosphate isomerase	-0.2942	0.0724
JW0213	yafJ	type 2 glutamine amidotransferase family protein	0.0620	0.0524
JW0214	yafK	L,D-transpeptidase-related protein	0.0750	0.0021
JW0215	yafQ	mRNA interferase toxin of toxin-antitoxin pair YafQ/DinJ	-0.0303	0.4339
JW0216	dinJ	antitoxin of YafQ-DinJ toxin-antitoxin system	-0.0570	0.1064
JW0217	yafL	putative lipoprotein and C40 family peptidase	-0.0215	0.4775
JW0218	yafM	RAYT REP element-mobilizing transposase; TnpA(REP)	-0.0479	0.1852
JW0221	dinB	DNA polymerase IV	-0.0101	0.6685
JW0222	yafN	antitoxin of the YafO-YafN toxin-antitoxin system	0.0263	0.2077
JW0223	yafO	mRNA interferase toxin of the YafO-YafN toxin-antitoxin system	-0.0151	0.5109
JW0224	yafP	GNAT family putative N-acetyltransferase	0.0746	0.0120

JW0225	ykfJ	pseudogene	-0.0460	0.0504
JW0227	pepD	aminoacyl-histidine dipeptidase (peptidase D)	0.0430	0.2061
JW0228	gpt	xanthine phosphoribosyltransferase; xanthine-guanine phosphoribosyltransferase	0.0168	0.3524
JW0229	frsA	fermentation-respiration switch protein; PTS Enzyme IIA(Glc)-binding protein; pNP-butyrate esterase activity	0.0029	0.1212
JW0230	crl	pseudogene, sigma factor-binding protein, RNA polymerase holoenzyme formation stimulator	-0.0159	0.4400
JW0231	phoE	outer membrane phosphoporin protein E	0.0559	0.2438
JW0232	proB	gamma-glutamate kinase	0.1457	0.0420
JW0233	proA	gamma-glutamylphosphate reductase	0.0718	0.0208
JW0234	ykfI	CP4-6 prophage; toxin of the YkfI-YafW toxin-antitoxin system	0.0149	0.2332
JW0235	yafW	CP4-6 prophage; antitoxin of the YkfI-YafW toxin-antitoxin system	0.0195	0.5969
JW0236	ykfG	CP4-6 prophage; RadC-like JAB domain protein	-0.0028	0.9444
JW0239	ykfB	CP4-6 prophage; uncharacterized protein	0.0306	0.3187
JW0240	yafY	lipoprotein, inner membrane; degP regulator; CP4-6 prophage	0.0797	0.0465
JW0242	yafZ	CP4-6 prophage; conserved protein	0.0286	0.3941
JW0243	ykfA	CP4-6 prophage; putative GTP-binding protein	0.0014	0.9760
JW0244	perR	CP4-6 prophage; putative DNA-binding transcriptional regulator	-0.1395	0.0365
JW0247	insO	2-octaprenyl-3-methyl-6-methoxy-1,4-benzoquinol hydroxylase	0.0033	0.9239
JW0253	mmuM	CP4-6 prophage; S-methylmethionine:homocysteine methyltransferase	0.0123	0.8598
JW0254	afuC	CP4-6 prophage; putative ferric transporter subunit	-0.0625	0.0038
JW0255	afuB	pseudogene, CP4-6 prophage; ferric iron ABC transporter permease gene fragment	0.0146	0.3920
JW0258	ykgN	Heat shock protein 15	0.0504	0.1257
JW0259	yagB	pseudogene, CP4-6 prophage	-0.0501	0.0551
JW0260	yagA	CP4-6 prophage; putative DNA-binding transcriptional regulator	-0.0562	0.1147
JW0261	yagE	2-keto-3-deoxy gluconate (KDG) aldolase; CP4-6 prophage	0.0803	0.0662
JW0264	yagH	CP4-6 prophage; putative xylosidase/arabinosidase	0.0205	0.3126
JW0265	yagI	CP4-6 prophage; putative DNA-binding transcriptional regulator	-0.0267	0.1848
JW0266	argF	ornithine carbamoyltransferase 2, chain F; CP4-6 prophage	0.1188	0.0003
JW0270	yagJ	CP4-6 prophage; uncharacterized protein	0.0073	0.7909

JW0271	yagK	CP4-6 prophage; conserved protein	0.0272	0.2190
JW0272	yagL	CP4-6 prophage; DNA-binding protein	-0.0266	0.2917
JW0273	yagM	CP4-6 prophage; uncharacterized protein	-0.0103	0.7524
JW0275	intF	CP4-6 prophage; putative phage integrase	-0.0276	0.6062
JW0276	yagP	pseudogene, LysR family, fragment	0.0501	0.0777
JW0277	yagQ	moco insertion factor for PaoABC aldehyde oxidoreductase	0.0413	0.0844
JW0278	yagR	PaoABC aldehyde oxidoreductase, Moco-containing subunit	0.0338	0.0001
JW0279	yagS	PaoABC aldehyde oxidoreductase, FAD-containing subunit	0.0131	0.6245
JW0280	yagT	PaoABC aldehyde oxidoreductase, 2Fe-2S subunit	0.1139	0.0061
JW0281	yagU	DUF1440 family inner membrane acid resistance protein	-0.0113	0.5426
JW0282	ykgJ	UPF0153 cysteine cluster protein	0.0209	0.6844
JW0284	yagW	polymerized tip adhesin of ECP fibers	-0.0370	0.1930
JW0285	yagX	ECP production outer membrane protein	0.0525	0.3218
JW0286	yagY	ECP production pilus chaperone	-0.0486	0.1318
JW0287	yagZ	ECP pilin	-0.0368	0.0302
JW0291	eaeH	pseudogene, attaching and effacing protein homology	-0.0137	0.4197
JW0298	ykgD	reactive chlorine species (RCS)-specific activator of the rcl genes	-0.0583	0.0082
JW0300	ykgF	ferridoxin-like LutB family protein; putative electron transport chain YkgEFG component	0.0841	0.0378
JW0302	ykgH	putative inner membrane protein	-0.0007	0.9785
JW0303	betA	choline dehydrogenase, a flavoprotein	-0.0593	0.2727
JW0305	betI	choline-inducible betIBA-betT divergent operon transcriptional repressor	0.0694	0.0720
JW0306	betT	choline transporter of high affinity	-0.0694	0.0508
JW0307	yahA	c-di-GMP-specific phosphodiesterase	-0.0858	0.0018
JW0308	yahB	putative DNA-bindng transcriptional regulator	-0.0698	0.0251
JW0309	yahC	putative inner membrane protein	0.0071	0.7466
JW0310	yahD	ankyrin repeat protein	-0.0003	0.9927
JW0311	yahE	DUF2877 family protein	-0.0451	0.1041
JW0312	yahF	putative NAD(P)-binding succinyl-CoA synthase	0.0580	0.0682
JW0313	yahG	DUF1116 family protein	0.0088	0.5659
JW0315	yahI	carbamate kinase-like protein	-0.0435	0.1041
JW0316	yahJ	putative metallo-dependent hydrolase domain deaminase	0.1698	0.0152
JW0317	yahK	broad specificity NADPH-dependent aldehyde reductase, Zn-containing	-0.0650	0.0114
JW0318	yahL	uncharacterized protein	0.0840	0.0001

JW0320	yahN	amino acid exporter for proline, lysine, glutamate, homoserine	0.0533	0.1929
JW0321	yahO	periplasmic protein, function unknown, YhcN family	-0.0505	0.0401
JW0322	prpR	propionate catabolism operon regulatory protein	-0.0447	0.0514
JW0323	prpB	2-methylisocitrate lyase	-0.0166	0.8476
JW0324	prpC	2-methylcitrate synthase	-0.0693	0.0017
JW0325	prpD	2-methylcitrate dehydratase	-0.0239	0.4568
JW0326	prpE	propionate--CoA ligase	0.0399	0.0191
JW0327	codB	cytosine transporter	-0.0054	0.9733
JW0328	codA	cytosine/isoguanine deaminase	0.0726	0.0123
JW0330	cynT	carbonic anhydrase	-0.0940	0.0368
JW0331	cynS	cyanate aminohydrolase	0.0108	0.7029
JW0332	cynX	putative cyanate transporter	-0.0478	0.1713
JW0333	lacA	thiogalactoside acetyltransferase	-0.0013	0.9685
JW0334	lacY	lactose permease	0.0006	0.1717
JW0336	lacI	lactose-inducible lac operon transcriptional repressor	-0.0393	0.0159
JW0337	mhpR	mhp operon transcriptional activator	-0.0519	0.0165
JW0338	mhpA	3-(3-hydroxyphenyl)propionate hydroxylase	-0.0050	0.7709
JW0339	mhpB	2,3-dihydroxyphenylpropionate 1,2-dioxygenase	-0.0498	0.1851
JW0340	mhpC	2-hydroxy-6-ketonona-2,4-dienedioic acid hydrolase	-0.1639	0.0030
JW0341	mhpD	2-keto-4-pentenoate hydratase	0.0628	0.5075
JW0342	mhpF	acetaldehyde-CoA dehydrogenase II, NAD-binding	-0.0336	0.3631
JW0343	mhpE	4-hyroxy-2-oxovalerate/4-hydroxy-2-oxopentanoic acid aldolase, class I	-0.0151	0.4418
JW0345	yaiL	DUF2058 family protein	0.0103	0.7899
JW0346	frmB	S-formylglutathione hydrolase	-0.0196	0.4250
JW0347	frmA	alcohol dehydrogenase class III; glutathione-dependent formaldehyde dehydrogenase	0.0005	0.9841
JW0348	frmR	regulator protein that represses frmRAB operon	-0.0098	0.5459
JW0349	yaiO	outer membrane protein	0.0472	0.0763
JW0350	yaiX	pseudogene, interrupted by IS2A; hexapeptide transferase superfamily	0.0301	0.2087
JW0355	yaiP	putative family 2 glycosyltransferase	0.2036	0.0002
JW0356	yaiS	putative PIG-L family deacetylase	-0.0172	0.5996
JW0357	tauA	taurine ABC transporter periplasmic binding protein	0.0637	0.1095
JW0358	tauB	taurine ABC transporter ATPase	-0.0476	0.2846
JW0359	tauC	taurine ABC transporter permease	0.0273	0.7155
JW0360	tauD	taurine dioxygenase, 2-oxoglutarate-dependent	0.0486	0.1110
JW0362	yaiT	pseudogene, autotransporter family	0.0344	0.3312
JW0366	yaiV	putative transcriptional regulator	0.0133	0.6567
JW0368	sbmA	peptide antibiotic transporter	-0.1435	0.0001
JW0369	yaiW	microcin Bac7 uptake protein; outer membrane surface-exposed lipoprotein	0.0203	0.2387

JW0370	yaiY	DUF2755 family inner membrane protein	0.0515	0.2744
JW0372	ddlA	D-alanine-D-alanine ligase A	0.0697	0.0792
JW0373	yaiB	anti-RssB factor, RpoS stabilzer during Pi starvation; anti-adapter protein	-0.0487	0.0062
JW0374	phoA	bacterial alkaline phosphatase	-0.0604	0.1740
JW0376	yaiC	diguanylate cyclase, cellulose regualtor	-0.0073	0.8053
JW0377	proC	pyrroline-5-carboxylate reductase, NAD(P)-binding	0.3748	0.0005
JW0378	yaiI	UPF0178 family protein	0.0491	0.0242
JW0379	aroL	shikimate kinase II	-0.2653	0.0162
JW0380	yaiA	OxyR-regulated conserved protein	0.0956	0.0418
JW0381	aroM	AroM family protein	-0.2024	0.2864
JW0382	yaiE	pyrimidine/purine nucleoside phosphorylase	-0.0294	0.2841
JW0383	ykiA	pseudogene	0.1479	0.0002
JW0385	mak	manno(fructo)kinase	-0.0084	0.6708
JW0386	araJ	L-arabinose-inducible putative transporter, MFS family	-0.0620	0.2381
JW0387	sbcC	exonuclease, dsDNA, ATP-dependent	0.1131	0.0292
JW0388	sbcD	exonuclease, dsDNA, ATP-dependent	-0.0020	0.9391
JW0389	phoB	response regulator in two-component regulatory system with PhoR	-0.0424	0.2103
JW0390	phoR	sensory histidine kinase in two-component regulatory system with PhoB	-0.0250	0.4879
JW0391	brnQ	branched-chain amino acid transport system 2 carrier protein; LIV-II transport system for Ile, Leu, and Val	-0.0001	0.9954
JW0393	malZ	maltodextrin glucosidase	-0.0378	0.1931
JW0394	yajB	acyl carrier protein (ACP) phosphodiesterase; ACP hydrolyase	0.0228	0.0025
JW0395	queA	S-adenosylmethionine:tRNA ribosyltransferase-isomerase	0.1078	0.0746
JW0396	tgt	tRNA-guanine transglycosylase	0.0113	0.8740
JW0397	yajC	SecYEG protein translocase auxillary subunit	-0.0460	0.2012
JW0400	yajD	HNH nuclease family protein	0.0422	0.2165
JW0401	tsx	nucleoside channel, receptor of phage T6 and colicin K	0.0285	0.8230
JW0403	ybaD	Nrd regulon repressor	-0.0237	0.0144
JW0406	nusB	transcription antitermination protein	-0.0005	0.9877
JW0408	pgpA	phosphatidylglycerophosphatase A	0.0033	0.9785
JW0409	yajO	2-carboxybenzaldehyde reductase	-0.0599	0.0860
JW0412	xseB	exonuclease VII small subunit	0.0922	0.0015
JW0413	thiI	tRNA s(4)U8 sulfurtransferase	0.1694	0.0286
JW0415	panE	2-dehydropantoate reductase, NADPH-specific	-0.0215	0.6088

JW0418	cyoE	protoheme IX farnesyltransferase	0.1488	0.0131
JW0419	cyoD	cytochrome o ubiquinol oxidase subunit IV	-0.0607	0.0053
JW0420	cyoC	cytochrome o ubiquinol oxidase subunit III	0.1647	0.0000
JW0421	cyoB	cytochrome o ubiquinol oxidase subunit I	-0.1840	0.1237
JW0422	cyoA	cytochrome o ubiquinol oxidase subunit II	-0.0480	0.2920
JW0423	ampG	muropeptide transporter	-0.0717	0.1260
JW0424	yajG	putative lipoprotein	0.0226	0.3448
JW0426	tig	peptidyl-prolyl cis/trans isomerase (trigger factor)	-0.0523	0.1749
JW0427	clpP	proteolytic subunit of ClpA-ClpP and ClpX-ClpP ATP-dependent serine proteases	0.0015	0.9782
JW0428	clpX	ATPase and specificity subunit of ClpX-ClpP ATP-dependent serine protease	0.0364	0.2136
JW0430	hupB	HU, DNA-binding transcriptional regulator, beta subunit	-0.1817	0.0015
JW0431	ppiD	periplasmic folding chaperone, has an inactive PPIase domain	0.0288	0.5853
JW0432	ybaV	putative competence-suppressing periplasmic helix-hairpin-helix DNA-binding protein	0.0604	0.0573
JW0433	ybaW	long-chain acyl-CoA thioesterase III	0.0417	0.1119
JW0434	ybaX	7-cyano-7-deazaguanine (preQ0) synthase; queuosine biosynthesis	-0.0802	0.0040
JW0435	ybaE	putative ABC transporter periplasmic binding protein	0.0433	0.0345
JW0436	cof	thiamine pyrimidine pyrophosphate hydrolase; HMP-PP phosphatase	-0.0300	0.1677
JW0437	ybaO	putative DNA-binding transcriptional regulator	0.0370	0.1508
JW0438	mdlA	putative multidrug ABC transporter ATPase	0.0493	0.0356
JW0440	glnK	nitrogen assimilation regulatory protein for GlnL, GlnE, and AmtB	-0.0048	0.8819
JW0441	amtB	ammonium transporter	0.0017	0.9535
JW0442	tesB	acyl-CoA thioesterase 2	-0.0544	0.4901
JW0443	ybaY	outer membrane lipoprotein	0.0357	0.0788
JW0444	ybaZ	excision repair protein, alkyltransferase-like protein ATL	-0.0945	0.0688
JW0445	ybaA	DUF1428 family protein	0.0593	0.1907
JW0448	maa	maltose O-acetyltransferase	0.1144	0.0009
JW0449	hha	modulator of gene expression, with H-NS	-0.0175	0.4444
JW0450	ybaJ	Hha toxicity attenuator; conjugation-related protein	-0.0176	0.4032
JW0451	acrB	multidrug efflux system protein	0.0360	0.3433
JW0452	acrA	multidrug efflux system	0.0837	0.0030
JW0453	acrR	transcriptional repressor	0.0517	0.0234
JW0454	kefA	mechanosensitive channel protein, intermediate conductance, K+ regulated	-0.1327	0.0406

JW0454	kefA	mechanosensitive channel protein, intermediate conductance, K+ regulated	0.0282	0.5983
JW0455	ybaM	DUF2496 family protein	0.1587	0.0001
JW0456	priC	primosomal replication protein N''	-0.0336	0.3113
JW0457	ybaN	DUF454 family inner membrane protein	0.0317	0.1013
JW0458	apt	adenine phosphoribosyltransferase	-0.0455	0.1807
JW0460	ybaB	DNA-binding protein, putative nucleoid-associated protein	-0.0570	0.0913
JW0461	recR	gap repair protein	0.1463	0.0011
JW0462	htpG	protein refolding molecular co-chaperone Hsp90, Hsp70-dependent; heat-shock protein; ATPase	0.0254	0.0743
JW0465	aes	acetyl esterase	0.1131	0.0502
JW0466	gsk	inosine/guanosine kinase	-0.0758	0.0414
JW0467	ybaL	inner membrane putative NAD(P)-binding transporter	0.0075	0.8085
JW0468	fsr	putative fosmidomycin efflux system protein	-0.1783	0.1178
JW0469	ushA	bifunctional UDP-sugar hydrolase/5'-nucleotidase	-0.0554	0.0088
JW0470	ybaK	Cys-tRNA(Pro)/Cys-tRNA(Cys) deacylase	0.0813	0.0006
JW0471	ybaP	TraB family protein	0.0369	0.1230
JW0472	ybaQ	putative DNA-binding transcriptional regulator	-0.0916	0.0043
JW0473	copA	copper transporter	0.0571	0.3965
JW0474	ybaS	glutaminase 1	-0.0180	0.3494
JW0475	ybaT	putative amino acid transporter	-0.0642	0.1950
JW0476	cueR	copper-responsive regulon transcriptional regulator	0.0563	0.0609
JW0478	ybbK	PHB domain membrane-anchored putative protease	0.1177	0.0013
JW0479	ybbL	iron export ABC transporter ATPase; peroxide resistance protein	-0.1177	0.3360
JW0482	ybbO	short-chain dehydrogenases/reductases (SDR) family protein	0.0551	0.0356
JW0483	tesA	acyl-CoA thioesterase 1 and protease I and lysophospholipase L1	-0.0862	0.0603
JW0484	ybbA	putative ABC transporter ATPase	0.0341	0.2921
JW0485	ybbP	putative ABC transporter permease	-0.0355	0.1730
JW0486	rhsD	Rhs protein with DUF4329 family putative toxin domain; putative neighboring cell growth inhibitor	-0.0010	0.9642
JW0487	ybbC	putative immunity protein	0.0929	0.0003
JW0488	ylbH	pseudogene, Rhs family C-terminal fragment with unique putative toxin domain	0.0047	0.8086

JW0489	ybbD	pseudogene	0.0673	0.0003
JW0491	ybbB	tRNA 2-selenouridine synthase, selenophosphate-dependent	0.0189	0.2198
JW0492	ybbS	allD operon transcriptional activator	-0.0515	0.0018
JW0493	allA	ureidoglycolate lyase, releasing urea	0.0619	0.0982
JW0494	allR	glyoxylate-inducible transcriptional repressor of all and gcl operons	0.0519	0.1331
JW0495	gcl	glyoxylate carboligase	0.0292	0.2641
JW0496	hyi	hydroxypyruvate isomerase	-0.0420	0.0337
JW0497	glxR	tartronate semialdehyde reductase, NADH-dependent	-0.0508	0.4112
JW0498	ybbV	Uncharacterized protein	0.0016	0.0161
JW0499	ybbW	putative allantoin transporter	0.1213	0.0170
JW0500	allB	allantoinase	-0.1142	0.0000
JW0501	ybbY	putative uracil/xanthine transporter	0.0009	0.9764
JW0502	glxK	glycerate kinase II	0.0148	0.7942
JW0503	ylbA	S-ureidoglycine aminohydrolase	-0.0486	0.0131
JW0504	allC	allantoate amidohydrolase	-0.0556	0.0126
JW0505	allD	ureidoglycolate dehydrogenase	0.0032	0.8113
JW0506	fdrA	putative NAD(P)-binding acyl-CoA synthetase	0.0276	0.0465
JW0508	ylbE	Uncharacterized protein	0.0322	0.1145
JW0509	ylbF	putative anaerobic allantoin catabolic oxamate carbamoyltransferase; DUF2877 family protein	0.0877	0.0041
JW0510	ybcF	putative carbonate kinase	0.1383	0.0028
JW0511	purK	N5-carboxyaminoimidazole ribonucleotide synthase	0.1136	0.0546
JW0514	ppiB	peptidyl-prolyl cis-trans isomerase B (rotamase B)	0.0562	0.1558
JW0516	ybcI	DUF457 family inner membrane protein	0.0484	0.1625
JW0519	sfmA	FimA homolog, function unknown	0.0161	0.8679
JW0520	sfmC	putative periplasmic pilus chaperone	0.0103	0.8083
JW0521	sfmD	putative outer membrane export usher protein	-0.0568	0.0257
JW0525	intD	DLP12 prophage; putative phage integrase	0.0021	0.8737
JW0526	ybcC	pseudogene, DLP12 prophage; phage-type exonuclease family	-0.1281	0.2438
JW0527	ybcD	pseudogene; DLP12 prophage; replication protein family	-0.0170	0.5564
JW0530	renD	pseudogene, DLP12 prophage	0.0125	0.5312
JW0531	emrE	DLP12 prophage; multidrug resistance protein	-0.0774	0.0180
JW0532	ybcK	DLP12 prophage; putative phage recombinase/integrase	-0.0136	0.7690
JW0533	ybcL	DLP12 prophage; inactive polymorphonuclear leukocyte migration suppressor; UPF0098 family secreted protein	0.0463	0.0251
JW0534	ybcM	DLP12 prophage; putative DNA-binding transcriptional regulator	0.1393	0.0054

JW0535	ybcN	DLP12 prophage; SSB and ssDNA binding protein; putative recombination protein	0.0255	0.1652
JW0536	ninE	DLP12 prophage; conserved protein	-0.0225	0.2634
JW0537	ybcO	DLP12 prophage; DUF1364 family protein	-0.0056	0.7531
JW0538	rusA	DLP12 prophage; endonuclease RUS	0.0473	0.0183
JW0539	ybcQ	DLP12 prophage; putative antitermination protein	0.0851	0.0846
JW0543	essD	DLP12 prophage; putative phage lysis protein	-0.0081	0.7490
JW0544	ybcS	DLP12 prophage; putative lysozyme	-0.0101	0.7355
JW0546	borD	DLP12 prophage; putative lipoprotein	-0.0589	0.0141
JW0548	ybcW	DLP12 prophage; uncharacterized protein	0.1560	0.0925
JW0549	nohB	DLP12 prophage; DNA packaging protein	-0.0738	0.1384
JW0551	ybcY	pseudogene, DLP12 prophage; methyltransferase homology	-0.0504	0.0207
JW0552	ylcE	pseudogene, DLP12 prophage	0.0389	0.2172
JW0553	appY	global transcriptional activator; DLP12 prophage	-0.0189	0.2124
JW0554	ompT	DLP12 prophage; outer membrane protease VII; outer membrane protein 3b	-0.0187	0.6659
JW0555	envY	porin thermoregulatory transcriptional activator	-0.0212	0.1416
JW0556	ybcH	PRK09936 family protein	0.0060	0.7827
JW0557	nfrA	bacteriophage N4 receptor, outer membrane subunit	0.0660	0.0064
JW0558	nfrB	bacteriophage N4 receptor, inner membrane subunit	0.0660	0.0618
JW0560	cusR	response regulator in two-component regulatory system with CusS	0.0009	0.9580
JW0561	cusC	copper/silver efflux system, outer membrane component	0.0121	0.5290
JW0562	cusF	periplasmic copper- and silver-binding protein	0.0166	0.4669
JW0563	cusB	copper/silver efflux system, membrane fusion protein	-0.4172	0.0001
JW0564	cusA	copper/silver efflux system, membrane component	0.0280	0.2991
JW0565	pheP	phenylalanine transporter	0.0241	0.2099
JW0566	ybdG	mechanosensitive channel protein, miniconductance	-0.0138	0.2492
JW0567	nfnB	dihydropteridine reductase, NAD(P)H-dependent, oxygen-insensitive	-0.1839	0.0058
JW0569	ybdJ	DUF1158 family putative inner membrane protein	-0.0013	0.9596
JW0570	ybdK	weak gamma-glutamyl:cysteine ligase	0.0509	0.0418
JW0576	fes	enterobactin/ferrienterobactin esterase	-0.1243	0.2965

JW0577	ybdZ	stimulator of EntF adenylation activity, MbtH-like	0.0072	0.7795
JW0578	entF	enterobactin synthase multienzyme complex component, ATP-dependent	0.0064	0.9007
JW0579	fepE	regulator of length of O-antigen component of lipopolysaccharide chains	-0.0952	0.0261
JW0580	fepC	ferrienterobactin ABC transporter ATPase	-0.0089	0.8604
JW0581	fepG	iron-enterobactin ABC transporter permease	0.1056	0.0108
JW0582	fepD	ferrienterobactin ABC transporter permease	0.0458	0.0086
JW0583	ybdA	enterobactin exporter, iron-regulated	0.1033	0.0465
JW0584	fepB	ferrienterobactin ABC transporter periplasmic binding protein	0.0275	0.6959
JW0585	entC	isochorismate synthase 1	0.0425	0.2846
JW0586	entE	2,3-dihydroxybenzoate-AMP ligase component of enterobactin synthase multienzyme complex	0.0863	0.1707
JW0587	entB	isochorismatase	-0.0660	0.0535
JW0588	entA	2,3-dihydro-2,3-dihydroxybenzoate dehydrogenase	-0.0259	0.3035
JW0589	ybdB	enterobactin synthesis proofreading thioesterase	-0.0111	0.5167
JW0590	cstA	carbon starvation protein involved in peptide utilization; APC peptide transporter family protein	-0.1690	0.0000
JW0591	ybdD	DUF466 family protein	0.0021	0.9183
JW0592	ybdH	hydroxycarboxylate dehydrogenase A	0.0244	0.4360
JW0594	ybdM	Spo0J family protein, ParB-like nuclease domain	-0.0235	0.0390
JW0595	ybdN	PAPS reductase-like domain protein	0.0076	0.6894
JW0596	ybdO	putative DNA-binding transcriptional regulator	0.0365	0.0207
JW0597	dsbG	thiol:disulfide interchange protein, periplasmic	-0.1461	0.0001
JW0598	ahpC	alkyl hydroperoxide reductase, C22 subunit	0.0160	0.5950
JW0599	ahpF	alkyl hydroperoxide reductase, F52a subunit, FAD/NAD(P)-binding	0.4632	0.0078
JW0600	uspG	universal stress protein UP12	-0.0606	0.0115
JW0601	ybdR	uncharacterized zinc-type alcohol dehydrogenase-like protein	0.0638	0.3428
JW0602	rnk	regulator of nucleoside diphosphate kinase	0.0222	0.4438
JW0603	rna	ribonuclease I	-0.0556	0.0300
JW0604	citT	citrate/succinate antiporter; citrate carrier	-0.0127	0.6205
JW0605	citG	2-(5''-triphosphoribosyl)-3'-dephosphocoenzyme-A synthase	0.0536	0.0384
JW0606	citX	apo-citrate lyase phosphoribosyl-dephospho-CoA transferase	-0.0158	0.6582

JW0608	citE	citrate lyase, cityl-ACP lyase (beta) subunit	0.0009	0.9637
JW0609	citD	citrate lyase, acyl carrier (gamma) subunit	0.0214	0.1721
JW0610	citC	citrate lyase ligase; [citrate [pro-3S]-lyase] ligase	-0.0414	0.2016
JW0611	citA	sensory histidine kinase in two-component regulatory system with CitB	-0.0912	0.2057
JW0612	citB	response regulator in two-component regulatory system with CitA	-0.0071	0.7523
JW0613	dcuC	Anaerobic C4-dicarboxylate transporter	0.0072	0.8311
JW0617	crcA	phospholipid:lipid A palmitoyltransferase	-0.0339	0.2120
JW0618	cspE	constitutive cold shock family transcription antitermination protein; negative regulator of cspA transcription; RNA melting protein; ssDNA-binding protein	-0.0278	0.2150
JW0619	crcB	fluoride efflux channel, dual topology membrane protein	0.0541	0.2072
JW0621	ybeM	Deaminated glutathione amidase	-0.1488	0.0024
JW0622	tatE	TatABCE protein translocation system subunit	0.0061	0.8393
JW0623	lipA	lipoyl synthase	0.0205	0.7960
JW0624	ybeF	LysR family putative transcriptional regulator	0.1445	0.0811
JW0626	ybeD	UPF0250 family protein	-0.2877	0.0305
JW0628	rlpA	septal ring protein, suppressor of prc, minor lipoprotein	0.0321	0.5482
JW0631	ybeA	23S rRNA m(3)Psi1915 pseudouridine methyltransferase, SAM-dependent	0.0559	0.0204
JW0633	cobC	putative alpha-ribazole-5'-P phosphatase	-0.1093	0.3467
JW0638	ybeL	DUF1451 family protein	-0.0385	0.0907
JW0640	ybeR	DUF1266 family protein	-0.1019	0.0001
JW0641	djlB	putative HscC co-chaperone, uncharacterized J domain-containing protein	0.0190	0.3005
JW0642	ybeT	Sel1 family TPR-like repeat protein	-0.0348	0.0965
JW0643	ybeU	DUF1266 family protein	-0.0378	0.1455
JW0644	djlC	J domain-containing HscC co-chaperone; Hsc56	0.0171	0.7258
JW0645	hscC	Hsp70 family chaperone Hsc62; RpoD-binding transcription inhibitor	0.0054	0.9279
JW0646	rihA	ribonucleoside hydrolase 1	-0.0649	0.1094
JW0647	gltL	glutamate/aspartate ABC transporter ATPase	-0.0257	0.4969
JW0648	gltK	glutamate/aspartate ABC transporter permease	-0.0897	0.0269
JW0649	gltJ	glutamate/aspartate ABC transporter permease	-0.0596	0.0225

JW0655	ybeX	putative ion transport	-0.1587	0.0432
JW0656	ybeY	ssRNA-specific endoribonuclease; 16S rRNA 3' end maturation and quality control co-endoribonuclease working with RNase R; rRNA transcription antitermination factor	-0.4173	0.0073
JW0657	ybeZ	heat shock protein, putative NTPase; PhoH-like protein	0.0069	0.6671
JW0658	miaB	tRNA-i(6)A37 methylthiotransferase	-0.0329	0.0725
JW0659	ubiF	2-octaprenyl-3-methyl-6-methoxy-1,4-benzoquinol oxygenase	0.0471	0.2975
JW0660	asnB	asparagine synthetase B	0.0639	0.0136
JW0661	nagD	UMP phosphatase	-0.0773	0.0687
JW0662	nagC	N-acetylglucosamine-inducible nag divergent operon transcriptional repressor	-0.0368	0.1139
JW0663	nagA	N-acetylglucosamine-6-phosphate deacetylase	-0.0545	0.1045
JW0664	nagB	glucosamine-6-phosphate deaminase	-0.0627	0.1224
JW0665	nagE	N-acetyl glucosamine specific PTS enzyme IIC, IIB, and IIA components	-0.0391	0.0869
JW0667	ybfM	chitoporin, uptake of chitosugars	0.0184	0.6284
JW0668	ybfN	chitosugar-induced verified lipoprotein	0.0272	0.4800
JW0669	fur	ferric iron uptake regulon transcriptional repressor; autorepressor	-0.0007	0.9637
JW0673	ybfF	acyl-CoA esterase	0.0172	0.2757
JW0674	seqA	negative modulator of initiation of replication	0.1028	0.0425
JW0675	pgm	phosphoglucomutase	0.0198	0.3650
JW0676	ybfP	lipoprotein	0.0422	0.0101
JW0679	potE	putrescine/proton symporter: putrescine/ornithine antiporter	-0.0681	0.0790
JW0680	speF	ornithine decarboxylase isozyme, inducible	0.0675	0.0803
JW0683	kdpD	fused sensory histidine kinase in two-component regulatory system with KdpE: signal sensing protein	0.1680	0.0401
JW0684	kdpC	potassium translocating ATPase, subunit C	-0.1064	0.2469
JW0685	kdpB	potassium translocating ATPase, subunit B	0.0046	0.8027
JW0686	kdpA	potassium translocating ATPase, subunit A	-0.0379	0.1112
JW0687	kdpF	potassium ion accessory transporter subunit	0.0340	0.2686
JW0688	ybfA	DUF2517 family protein	-0.0628	0.0845
JW0689	rhsC	Rhs protein with putative toxin domain; putative neighboring cell growth inhibitor	-0.0503	0.2442
JW0691	ybfB	putative membrane protein	-0.0356	0.1796
JW0692	ybfO	pseudogene, Rhs family	-0.0125	0.6588

JW0693	ybfC	putative secreted protein	0.0098	0.4317
JW0694	ybfQ	Putative defective transposase	0.0531	0.0856
JW0695	ybfL	pseudogene, DDE domain transposase family	0.0662	0.1256
JW0696	ybfD	H repeat-associated putative transposase	-0.0583	0.0451
JW0697	ybgA	DUF1722 family protein	0.0042	0.8710
JW0698	phr	deoxyribodipyrimidine photolyase, FAD-binding	-0.1874	0.1171
JW0699	ybgH	dipeptide and tripeptide permease D	0.0291	0.2222
JW0700	ybgI	GTP cyclohydrolase-like radiation resistance protein; metal-binding	0.0276	0.5652
JW0701	ybgJ	putative allophanate hydrolase, subunit 1	0.1015	0.0025
JW0702	ybgK	putative allophanate hydrolase, subunit 2	-0.0545	0.0416
JW0703	ybgL	UPF0271 family protein	0.0078	0.7737
JW0704	nei	endonuclease VIII and 5-formyluracil/5-hydroxymethyluracil DNA glycosylase	-0.0675	0.2148
JW0707	ybgP	putative periplasmic pilin chaperone	0.0060	0.8791
JW0709	ybgD	putative fimbrial-like adhesin protein	-0.0946	0.0010
JW0710	gltA	citrate synthase	0.1277	0.0885
JW0711	sdhC	succinate dehydrogenase, membrane subunit, binds cytochrome b556	-0.0043	0.7547
JW0712	sdhD	succinate dehydrogenase, membrane subunit, binds cytochrome b556	0.0652	0.0902
JW0713	sdhA	succinate dehydrogenase, flavoprotein subunit	0.0870	0.0198
JW0714	sdhB	succinate dehydrogenase, FeS subunit	-0.0289	0.1476
JW0715	sucA	2-oxoglutarate decarboxylase, thiamine triphosphate-binding	-0.0290	0.7699
JW0716	sucB	dihydrolipoyl succinyltransferase, subunit of 2-oxoglutarate dehydrogenase	0.1171	0.0004
JW0717	sucC	succinyl-CoA synthetase, beta subunit	0.0562	0.1679
JW0718	sucD	succinyl-CoA synthetase, NAD(P)-binding, alpha subunit	0.1277	0.0347
JW0719	mngR	transcriptional repressor for the mannosyl-D-glycerate catabolic operon	-0.1341	0.0057
JW0720	mngA	fused 2-O-a-mannosyl-D-glycerate specific PTS enzymes: IIA component/IIB component/IIC component	0.3256	0.0035
JW0721	mngB	alpha-mannosidase	-0.0134	0.4323
JW0723	cydB	cytochrome d terminal oxidase, subunit II	0.0072	0.6653
JW0724	ybgT	cytochrome d (bd-I) ubiquinol oxidase subunit X	0.0676	0.0126
JW0725	ybgE	putative inner membrane protein in cydABX-ybgE operon	-0.0312	0.1127

JW0726	ybgC	acyl-CoA thioester hydrolase	-0.1118	0.0342
JW0727	tolQ	membrane spanning protein in TolA-TolQ-TolR complex	0.1215	0.0001
JW0728	tolR	membrane spanning protein in TolA-TolQ-TolR complex	-0.1620	0.1417
JW0729	tolA	membrane anchored protein in TolA-TolQ-TolR complex	0.0992	0.0003
JW0731	pal	peptidoglycan-associated outer membrane lipoprotein	-0.0697	0.1271
JW0732	ybgF	periplasmic TolA-binding protein	-0.0865	0.0037
JW0733	nadA	quinolinate synthase, subunit A	0.2520	0.0001
JW0734	pnuC	nicotinamide riboside transporter	-0.0469	0.1126
JW0735	zitB	zinc efflux system	-0.0526	0.0053
JW0736	ybgS	putative periplasmic protein	-0.0452	0.0762
JW0737	aroG	3-deoxy-D-arabino-heptulosonate-7-phosphate synthase, phenylalanine repressible	0.0453	0.0997
JW0738	gpmA	phosphoglyceromutase 1	0.0000	0.9990
JW0739	galM	aldose 1-epimerase; type-1 mutarotase; galactose mutarotase	0.1577	0.0000
JW0740	galK	galactokinase	-0.0389	0.3534
JW0741	galT	galactose-1-phosphate uridylyltransferase	-0.0810	0.0006
JW0742	galE	UDP-galactose-4-epimerase	0.0097	0.8270
JW0743	modF	molybdate ABC transporter ATPase	-0.0722	0.1842
JW0744	modE	transcriptional repressor for the molybdenum transport operon modABC	-0.0410	0.5248
JW0747	modB	molybdate ABC transporter permease; chlorate resistance protein	0.0910	0.0021
JW0748	modC	molybdate ABC transporter ATPase; chlorate resistance protein	0.0518	0.3994
JW0749	ybhA	pyridoxal phosphate (PLP) phosphatase	0.0695	0.0107
JW0750	ybhE	6-phosphogluconolactonase	-0.0628	0.0408
JW0752	ybhH	putative PrpF family isomerase	0.0048	0.7957
JW0753	ybhI	putative DASS family tricarboxylate or dicarboxylate transporter	0.0453	0.0416
JW0755	ybhC	acyl-CoA thioesterase, lipoprotein	0.1452	0.0211
JW0756	ybhB	kinase inhibitor homolog, UPF0098 family	-0.0522	0.0225
JW0757	bioA	7,8-diaminopelargonic acid synthase, PLP-dependent	0.1248	0.0019
JW0758	bioB	biotin synthase	0.1907	0.0854
JW0759	bioF	8-amino-7-oxononanoate synthase	0.1890	0.0010
JW0760	bioC	malonyl-ACP O-methyltransferase, SAM-dependent	0.1850	0.0097
JW0761	bioD	dethiobiotin synthetase	0.0368	0.4311
JW0762	uvrB	exision nuclease of nucleotide excision repair, DNA damage recognition component	0.0596	0.1760
JW0763	ybhK	putative CofD superfamily transferase	0.0169	0.5842
JW0764	moaA	molybdopterin biosynthesis protein A	0.0595	0.0121

JW0765	moaB	inactive molybdopterin adenylyltransferase	-0.0026	0.9322
JW0766	moaC	molybdopterin biosynthesis, protein C	-0.1019	0.0006
JW0767	moaD	molybdopterin synthase, small subunit	-0.0765	0.0687
JW0768	moaE	molybdopterin synthase, large subunit	0.0059	0.8292
JW0769	ybhL	putative acetate transporter; BAX Inhibitor-1 family inner membrane protein	-0.0594	0.6739
JW0770	ybhM	BAX Inhibitor-1 family inner membrane protein	0.0027	0.8898
JW0772	ybhO	cardiolipin synthase 2	-0.2683	0.0006
JW0773	ybhP	endo/exonuclease/phosphatase family protein	0.0164	0.5391
JW0774	ybhQ	inner membrane protein	-0.0404	0.1556
JW0777	ybhS	putative ABC transporter permease	-0.0954	0.0710
JW0779	ybhG	putative membrane fusion protein (MFP) component of efflux pump, membrane anchor	0.0147	0.5810
JW0780	ybiH	DUF1956 domain-containing tetR family putative transcriptional regulator	-0.0022	0.9418
JW0781	rhlE	ATP-dependent RNA helicase	0.0377	0.4712
JW0783	ybiA	DUF1768 family protein	-0.0281	0.0817
JW0784	dinG	ATP-dependent DNA helicase	0.0020	0.9425
JW0785	ybiB	putative family 3 glycosyltransferase	0.0063	0.8132
JW0786	ybiC	hydroxycarboxylate dehydrogenase B	0.0055	0.8920
JW0787	ybiJ	DUF1471 family periplasmic protein, trinitrotoluene-inducible	-0.0337	0.1626
JW0788	ybiI	DksA-type zinc finger protein	-0.0693	0.0015
JW0790	fiu	catecholate siderophore receptor	0.0106	0.7370
JW0794	glnQ	glutamine transporter subunit	-0.0072	0.7740
JW0795	glnP	glutamine transporter subunit	-0.1106	0.1289
JW0796	glnH	glutamine transporter subunit	-0.0360	0.3705
JW0797	dps	Fe-binding and storage protein; stress-inducible DNA-binding protein	0.0000	0.9990
JW0798	rhtA	threonine and homoserine efflux system	0.0655	0.0580
JW0799	ompX	outer membrane protein X	-0.0648	0.0185
JW0800	ybiP	OPG biosynthetic transmembrane phosphoethanolamine transferase	0.0081	0.8260
JW0801	mntR	Mn(2+)-responsive manganese regulon transcriptional regulator	-0.0210	0.3015
JW0802	ybiR	putative ArsB family transporter; inner membrane protein	-0.0449	0.0479
JW0803	ybiS	L,D-transpeptidase linking Lpp to murein	0.0420	0.0596
JW0804	ybiT	ABC-F family putative regulatory ATPase	-0.2325	0.0566
JW0805	ybiU	DUF1479 family protein	-0.0065	0.7622

JW0806	ybiV	sugar phosphatase; fructose-1-P/ribose-5-P/glucose-6-P phosphatase	-0.0445	0.2509
JW0807	ybiW	putative pyruvate formate lyase	0.0872	0.0017
JW0808	ybiY	putative pyruvate formate lyase activating enzyme	-0.0579	0.0465
JW0810	moeB	molybdopterin synthase sulfurylase	0.0060	0.8416
JW0811	moeA	molybdopterin molybdenumtransferase; molybdopterin biosynthesis protein	-0.0420	0.5508
JW0812	iaaA	Isoaspartyl peptidase	0.0074	0.7565
JW0815	yliC	glutathione ABC transporter permease	0.0723	0.0253
JW0816	yliD	glutathione ABC transporter permease	-0.0668	0.5316
JW0817	yliE	putative membrane-anchored cyclic-di-GMP phosphodiesterase	-0.0710	0.0253
JW0818	yliF	putative membrane-anchored diguanylate cyclase	-0.0394	0.1955
JW0819	yliG	ribosomal protein S12 methylthiotransferase	0.1266	0.0293
JW0820	yliH	repressor of biofilm formation by indole transport regulation	-0.0322	0.3865
JW0821	yliI	soluble aldose sugar dehydrogenase	0.0278	0.3856
JW0822	yliJ	glutathione S-transferase	-0.0655	0.0218
JW0823	dacC	D-alanyl-D-alanine carboxypeptidase; penicillin-binding protein 6a	-0.0098	0.8100
JW0824	deoR	deoxyribose-5-phosphate-inducible deoxyribose operon transcriptional repressor; repressor of nupG and tsx	-0.0030	0.8056
JW0826	cmr	multidrug efflux system protein	-0.0124	0.8193
JW0827	ybjH	uncharacterized protein	0.0035	0.8147
JW0829	ybjJ	putative drug efflux MFS transporter, inner membrane protein	0.0039	0.9235
JW0831	ybjL	putative transporter	-0.0357	0.5134
JW0832	ybjM	inner membrane protein	-0.0274	0.0479
JW0833	grxA	glutaredoxin 1, redox coenzyme for ribonucleotide reductase (RNR1a)	-0.0004	0.9906
JW0834	ybjC	DUF1418 family protein	-0.0248	0.4200
JW0835	nfsA	nitroreductase A, NADPH-dependent, FMN-dependent	-0.0200	0.4996
JW0836	rimK	ribosomal protein S6 modification protein	0.0438	0.0245
JW0837	ybjN	multicopy suppressor of coaA(Ts); ionizing radiation survival protein; putative chaperone; putative negative regulator of fimbriae and motility	0.1713	0.0586
JW0838	potF	putrescine ABC transporter periplasmic binding protein	-0.0625	0.0002
JW0840	potH	putrescine ABC transporter permease	0.0772	0.1373

JW0841	potI	putrescine ABC transporter permease	0.1676	0.1732
JW0842	ybjO	DUF2593 family inner membrane protein	0.0521	0.0766
JW0844	artJ	arginine ABC transporter periplasmic binding protein	0.0545	0.4118
JW0845	artM	arginine ABC transporter permease	-0.0106	0.7253
JW0846	artQ	arginine ABC transporter permease	-0.0121	0.6826
JW0847	artI	arginine transporter subunit	0.0011	0.9632
JW0848	artP	arginine ABC transporter ATPase	0.0094	0.6916
JW0849	ybjP	lipoprotein	0.0384	0.0707
JW0850	ybjQ	UPF0145 family protein	-0.0454	0.0000
JW0851	ybjR	1,6-anhydro-N-acetylmuramyl-L-alanine amidase, Zn-dependent; OM lipoprotein	0.0701	0.1207
JW0854	ltaE	L-allo-threonine aldolase, PLP-dependent	0.0081	0.6478
JW0855	poxB	pyruvate dehydrogenase, thiamine triphosphate-binding, FAD-binding	0.0482	0.0993
JW0858	ybjE	putative transporter	0.0078	0.8427
JW0859	aqpZ	aquaporin Z	0.0919	0.0385
JW0860	ybjD	putative OLD family ATP-dependent endonuclease; DUF2813 family protein	-0.0163	0.5726
JW0861	ybjX	DUF535 family protein	0.0153	0.6982
JW0862	macA	macrolide transporter membrane fusion protein (MFP) component	0.0490	0.0569
JW0863	macB	macrolide ABC transporter peremase/ATPase	0.0423	0.3108
JW0864	cspD	inhibitor of DNA replication, cold shock protein homolog	0.1141	0.0028
JW0865	clpS	regulatory protein for ClpA substrate specificity	-0.0167	0.6505
JW0866	clpA	ATPase and specificity subunit of ClpA-ClpP ATP-dependent serine protease, chaperone activity	-0.0009	0.9761
JW0868	aat	leucyl/phenylalanyl-tRNA-protein transferase	0.0316	0.0966
JW0870	cydD	glutathione/cysteine ABC transporter export permease/ATPase	-0.1451	0.3942
JW0871	trxB	thioredoxin reductase, FAD/NAD(P)-binding	0.0528	0.1384
JW0872	lrp	leucine-responsive global transcriptional regulator	0.0704	0.0630
JW0875	ycaJ	recombination intermediate processing DNA-dependent ATPase	0.1241	0.0025
JW0878	dmsB	dimethyl sulfoxide reductase, anaerobic, subunit B	0.0883	0.0080
JW0879	dmsC	dimethyl sulfoxide reductase, anaerobic, subunit C	0.0926	0.0080
JW0880	ycaC	putative isochorismatase family hydrolase	0.0317	0.0702
JW0881	ycaD	putative MFS transporter, inner membrane protein	-0.2113	0.1024

JW0883	ycaN	LysR family putative transcriptional regulator	-0.0099	0.7248
JW0884	ycaK	putative NAD(P)H-dependent oxidoreductase	-0.0809	0.0860
JW0885	pflA	pyruvate formate-lyase 1-activating enzyme; [formate-C-acetyltransferase 1]-activating enzyme; PFL activase	-0.3423	0.0000
JW0886	pflB	formate C-acetyltransferase 1, anaerobic; pyruvate formate-lyase 1	-0.1625	0.0000
JW0887	focA	formate channel	0.0119	0.6463
JW0888	ycaO	ribosomal protein S12 methylthiotransferase accessory factor	0.0822	0.0125
JW0889	ycaP	UPF0702 family putative inner membrane protein	-0.0617	0.1927
JW0890	serC	3-phosphoserine/phosphohydroxythreonine aminotransferase	0.1942	0.0034
JW0891	aroA	5-enolpyruvylshikimate-3-phosphate synthetase	0.1067	0.0240
JW0892	ycaL	putative peptidase-related chaperone	0.0121	0.6023
JW0893	cmk	cytidylate kinase	-0.2031	0.1069
JW0895	ihfB	integration host factor (IHF), DNA-binding protein, beta subunit	0.0113	0.5668
JW0899	ycaQ	DUF1006 family protein with C-terminal wHTH domain	-0.0708	0.0019
JW0900	ycaR	peroxide and acid resistance protein, UPF0434 family	-0.0411	0.4108
JW0903	ycbC	envelope biogenesis factor; DUF218 superfamily protein	-0.0459	0.5343
JW0904	smtA	putative S-adenosyl-L-methionine-dependent methyltransferase	0.0192	0.1825
JW0908	ycbB	murein L,D-transpeptidase	0.0051	0.8814
JW0909	ycbK	M15A protease-related family periplasmic protein	-0.0508	0.0380
JW0910	ycbL	putative metal-binding enzyme	-0.1061	0.3013
JW0911	aspC	aspartate aminotransferase, PLP-dependent	-0.0803	0.2517
JW0912	ompF	outer membrane porin 1a (Ia;b;F)	0.0162	0.4283
JW0914	pncB	nicotinate phosphoribosyltransferase	0.1026	0.0130
JW0915	pepN	aminopeptidase N	-0.0113	0.6900
JW0916	ssuB	aliphatic sulfonate ABC transporter ATPase	-0.0691	0.0661
JW0918	ssuD	alkanesulfonate monooxygenase, FMNH(2)-dependent	-0.0514	0.0707
JW0919	ssuA	aliphatic sulfonate ABC transporter periplasmic binding protein	-0.0112	0.8008
JW0920	ssuE	NAD(P)H-dependent FMN reductase	0.2421	0.0685
JW0922	ycbR	putative periplasmic pilin chaperone	-0.0168	0.5521
JW0923	ycbS	putative outer membrane fimbrial subunit export usher protein	0.0179	0.6049
JW0924	ycbT	putative fimbrial-like adhesin protein	-0.0925	0.0007
JW0925	ycbU	putative fimbriae protein	-0.0563	0.0280

JW0925	ycbU	putative fimbriae protein	-0.0338	0.2286
JW0928	pyrD	dihydro-orotate oxidase, FMN-linked	-0.0380	0.4724
JW0931	ycbY	23S rRNA m(2)G2445 and m(7)G2069 methyltransferases, SAM-dependent	-0.1018	0.0008
JW0932	uup	replication regulatory ABC-F family DNA-binding ATPase	0.0514	0.1682
JW0933	pqiA	inner membrane subunit of the putative PqiABC transporter	0.1027	0.0098
JW0934	pqiB	periplasmic MCE subunit of the putative PqiABC transporter, IM-anchored, paraquat-inducible	-0.0925	0.0039
JW0936	rmf	ribosome modulation factor	-0.0528	0.0296
JW0938	ycbZ	putative peptidase	-0.0613	0.1170
JW0939	ycbG	Ter macrodomain organizer matS-binding protein	-0.0641	0.0368
JW0940	ompA	outer membrane protein A (3a;II*;G;d)	0.1689	0.0113
JW0941	sulA	SOS cell division inhibitor	-0.0278	0.1279
JW0942	yccR	CRP-S-dependent promoter expression factor	-0.0778	0.1144
JW0944	yccF	DUF307 family inner membrane protein	0.1514	0.0022
JW0945	helD	DNA helicase IV	0.0857	0.0001
JW0947	yccT	UPF0319 family protein	0.0407	0.2460
JW0952	yccK	mnm(5)-s(2)U34-tRNA 2-thiolation sulfurtransferase	-0.0446	0.2200
JW0953	yccA	Modulator of FtsH protease, inner membrane protein	0.0802	0.5564
JW0954	hyaA	hydrogenase 1, small subunit	0.0539	0.1685
JW0955	hyaB	hydrogenase 1, large subunit	-0.1019	0.0582
JW0956	hyaC	hydrogenase 1, b-type cytochrome subunit	0.1108	0.0000
JW0957	hyaD	hydrogenase 1 maturation protease	-0.0369	0.0227
JW0958	hyaE	putative HyaA chaperone	0.0074	0.7538
JW0959	hyaF	hydrogenase-1 protein nickel incorporation factor	0.0519	0.1039
JW0960	appC	cytochrome bd-II oxidase, subunit I	0.0353	0.3251
JW0961	appB	cytochrome bd-II oxidase, subunit II	0.0435	0.4133
JW0963	appA	phosphoanhydride phosphorylase	-0.0413	0.1263
JW0964	yccC	tyrosine-protein kinase, role in O-antigen capsule formation	-0.0891	0.0009
JW0966	yccZ	putative O-antigen capsule outer membrane auxillary protein export channel	0.0788	0.0059
JW0967	ymcA	putative O-antigen capsule production periplasmic protein	-0.0118	0.6588
JW0968	ymcB	putative O-antigen capsule production periplasmic protein	-0.0720	0.0462
JW0969	ymcC	O-antigen capsule production lipoprotein	0.0512	0.0687
JW0974	cspG	cold shock protein homolog, cold-inducible	-0.1133	0.4060
JW0975	ymcE	cold shock gene	0.0685	0.0835
JW0976	gnsA	putative phosphatidylethanolamine synthesis regulator	-0.0813	0.0529

JW0977	yccM	putative 4Fe-4S membrane protein	-0.0740	0.1439
JW0980	torR	response regulator in two-component regulatory system with TorS	0.0443	0.2154
JW0981	torC	trimethylamine N-oxide (TMAO) reductase I, cytochrome c-type subunit	0.0908	0.1952
JW0982	torA	trimethylamine N-oxide (TMAO) reductase I, catalytic subunit	0.0977	0.0027
JW0983	torD	TorA-maturation chaperone	-0.0238	0.3785
JW0985	cbpA	DnaK co-chaperone; curved DNA-binding protein	0.0268	0.5277
JW0986	yccE	PRK09784 family protein	0.3264	0.0000
JW0987	agp	glucose-1-phosphatase/inositol phosphatase	0.0507	0.1170
JW0988	yccJ	uncharacterized protein	0.0258	0.1525
JW0989	wrbA	NAD(P)H:quinone oxidoreductase	-0.0831	0.0023
JW0993	ycdI	putative malonic semialdehyde reductase	-0.0159	0.5506
JW0994	rarA	putative reactive intermediate detoxifying aminoacrylate hydrolase	-0.1507	0.0737
JW0995	ycdK	putative aminoacrylate deaminase, reactive intermediate detoxification; weak enamine/imine deaminase activity	-0.0161	0.7270
JW0997	ycdM	pyrimidine oxygenase, FMN-dependent	-0.0211	0.3375
JW0998	ycdC	rut operon transcriptional repressor for	-0.0075	0.8402
JW0999	putA	fused DNA-binding transcriptional regulator/proline dehydrogenase/pyrroline-5-carboxylate dehydrogenase	0.1676	0.0099
JW1001	putP	proline:sodium symporter	-0.0246	0.3811
JW1002	ycdN	Putative inactive ferrous iron permease	0.0148	0.6045
JW1003	ycdO	inactive ferrous ion transporter EfeUOB	-0.0196	0.4997
JW1004	ycdB	deferrrochelatase, periplasmic	-0.0378	0.1424
JW1005	phoH	ATP-binding protein; putative PhoH family P-loop ATPase	-0.0297	0.4078
JW1006	ycdP	biofilm PGA synthase PgaCD, regulatory subunit; c-di-GMP-stimulated activity and dimerization	0.0427	0.0715
JW1007	ycdQ	biofilm PGA synthase PgaCD, catalytic subunit; poly-beta-1,6-N-acetyl-D-glucosamine synthase	0.0588	0.0232
JW1010	ycdS	biofilm adhesin polysaccharide PGA secretin; OM porin; poly-beta-1,6-N-acetyl-D-glucosamine export protein	0.0886	0.0114
JW1015	ycdU	putative inner membrane protein	0.0259	0.3912
JW1017	ycdX	alkaline phosphatase	0.0445	0.0176

JW1018	ycdY	redox enzyme maturation protein (REMP) chaperone for YcdX	-0.0181	0.4021
JW1020	csgG	curli production assembly/transport outer membrane lipoprotein	-0.1016	0.0003
JW1021	csgF	curli nucleation outer membrane protein	0.1788	0.0006
JW1022	csgE	curlin secretion specificity factor	-0.0185	0.6024
JW1023	csgD	csgBAC operon transcriptional regulator	-0.0487	0.1433
JW1024	csgB	curlin nucleator protein, minor subunit in curli complex	-0.0026	0.9454
JW1025	csgA	curlin subunit, amyloid curli fibers, cryptic	0.0329	0.5162
JW1026	csgC	curli assembly protein	-0.0224	0.4378
JW1031	ymdA	uncharacterized protein	0.0427	0.1441
JW1032	ymdB	O-acetyl-ADP-ribose deacetylase; RNase III inhibitor during cold shock; putative cardiolipin synthase C regulatory subunit	0.0697	0.0090
JW1034	mdoC	OPG biosynthetic transmembrane succinyltransferase	0.0229	0.1295
JW1035	mdoG	OPG biosynthetic periplasmic beta-1,6 branching glycosyltransferase	-0.0512	0.1600
JW1037	mdoH	OPG biosynthetic ACP-dependent transmembrane UDP-glucose beta-1,2 glycosyltransferase; nutrient-dependent cell size regulator, FtsZ assembly antagonist	-0.0045	0.8789
JW1039	msyB	multicopy suppressor of secY and secA	-0.0289	0.3059
JW1040	mdtG	putative drug efflux system protein	-0.0557	0.2367
JW1041	lpxL	lauryl-acyl carrier protein (ACP)-dependent acyltransferase	-0.0463	0.7670
JW1042	yceA	putative rhodanese-related sulfurtransferase	-0.0728	0.2054
JW1043	yceI	periplasmic high pH-inducible lipid-binding protein	0.1365	0.0008
JW1044	yceJ	putative cytochrome b561	0.0078	0.7247
JW1045	yceO	uncharacterized protein	-0.1141	0.0003
JW1046	solA	N-methyltryptophan oxidase, FAD-binding	-0.0031	0.9384
JW1048	dinI	DNA damage-inducible protein I	-0.0670	0.7188
JW1049	pyrC	dihydro-orotase	0.0447	0.2334
JW1050	yceB	lipoprotein, DUF1439 family	0.0551	0.0063
JW1051	grxB	glutaredoxin 2 (Grx2)	-0.0037	0.9563
JW1052	mdtH	multidrug resistance efflux transporter conferring overexpression resistance to norfloxacin and enoxacin	0.0176	0.5799
JW1053	rimJ	ribosomal-protein-S5-alanine N-acetyltransferase	0.0323	0.6001
JW1054	yceH	UPF0502 family protein	-0.0139	0.2066

JW1055	mviM	putative oxidoreductase	-0.0330	0.1421
JW1057	flgN	export chaperone for FlgK and FlgL	-0.0433	0.2950
JW1059	flgA	assembly protein for flagellar basal-body periplasmic P ring	0.1145	0.0109
JW1060	flgB	flagellar component of cell-proximal portion of basal-body rod	0.0171	0.6494
JW1061	flgC	flagellar component of cell-proximal portion of basal-body rod	0.0547	0.3716
JW1062	flgD	flagellar hook assembly protein	0.1365	0.0013
JW1063	flgE	flagellar hook protein	0.0627	0.3361
JW1064	flgF	flagellar component of cell-proximal portion of basal-body rod	-0.0754	0.1717
JW1065	flgG	flagellar component of cell-distal portion of basal-body rod	0.0327	0.4728
JW1067	flgI	putative flagellar basal body protein	-0.0011	0.9817
JW1068	flgJ	bifunctional flagellar rod assembly protein (N-terminal) and beta-N-acetylglucosaminidase (C-terminal)	0.1302	0.0090
JW1069	flgK	flagellar hook-filament junction protein 1	0.1292	0.0050
JW1070	flgL	flagellar hook-filament junction protein	0.0563	0.0539
JW1072	rluC	23S rRNA pseudouridine(955,2504,2580) synthase	0.0620	0.0137
JW1074	yceD	DUF177 family protein	-0.0016	0.9148
JW1075	rpmF	50S ribosomal subunit protein L32	-0.0797	0.0174
JW1077	fabH	3-oxoacyl-[acyl-carrier-protein] synthase III	0.0887	0.0171
JW1081	fabF	3-oxoacyl-[acyl-carrier-protein] synthase II	-0.1612	0.0039
JW1082	pabC	4-amino-4-deoxychorismate lyase component of para-aminobenzoate synthase multienzyme complex	-0.0195	0.5548
JW1083	yceG	septation protein, ampicillin sensitivity	-0.0117	0.7275
JW1086	ycfH	putative DNase	0.1408	0.0000
JW1087	ptsG	fused glucose-specific PTS enzymes: IIB component/IIC component	0.0451	0.5418
JW1088	fhuE	ferric-rhodotorulic acid outer membrane transporter	-0.0222	0.6150
JW1090	ycfL	uncharacterized protein	0.0579	0.0177
JW1092	ycfN	thiamine kinase	0.0977	0.0041
JW1093	nagZ	beta N-acetyl-glucosaminidase	0.0145	0.6269
JW1095	ndh	respiratory NADH dehydrogenase 2/cupric reductase	-0.0551	0.0030
JW1096	ycfJ	uncharacterized protein	0.1401	0.1990
JW1098	ycfR	biofilm, cell surface and signaling protein	0.0435	0.0479
JW1100	mfd	transcription-repair coupling factor	-0.2756	0.2021

JW1101	ycfT	inner membrane protein	-0.0738	0.0344
JW1105	ycfX	N-acetyl-D-glucosamine kinase	0.0309	0.2325
JW1106	cobB	deacetylase of acs and cheY, chemotaxis regulator	-0.0473	0.0542
JW1109	potD	spermidine/putrescine ABC transporter periplasmic binding protein	0.0096	0.7184
JW1110	potC	spermidine/putrescine ABC transporter permease	0.0237	0.5522
JW1111	potB	spermidine/putrescine ABC transporter permease	0.0317	0.3690
JW1112	potA	spermidine/putrescine ABC transporter ATPase	-0.0219	0.4607
JW1113	pepT	peptidase T	-0.0333	0.4337
JW1114	ycfD	50S ribosomal protein L16 arginine hydroxylase; 2-oxoglutarate oxygenase	-0.0883	0.0017
JW1115	phoQ	sensory histidine kinase in two-component regulatory system with PhoP	0.0218	0.5920
JW1116	phoP	response regulator in two-component regulatory system with PhoQ	0.0137	0.4979
JW1119	trmU	tRNA(Gln,Lys,Glu) U34 2-thiouridylase	-0.2376	0.0442
JW1120	ymfB	bifunctional thiamine pyrimidine pyrophosphate hydrolase and thiamine pyrophosphate hydrolase	0.0674	0.1163
JW1121	ymfC	23S rRNA pseudouridine(2457) synthase	-0.1132	0.0287
JW1122	icd	isocitrate dehydrogenase; e14 prophage attachment site; tellurite reductase	0.1241	0.0048
JW1123	ymfD	e14 prophage; putative SAM-dependent methyltransferase	-0.0517	0.0107
JW1125	lit	T4 phage exclusion protein; cell death peptidase, e14 prophage	-0.0496	0.1473
JW1126	intE	e14 prophage; putative integrase	0.0080	0.6833
JW1127	ymfG	e14 prophage; putative excisionase	0.0261	0.2316
JW1128	ymfH	Putative uncharacterized protein b1142	-0.0531	0.0126
JW1130	ymfJ	Uncharacterized protein	0.3479	0.0352
JW1133	ymfL	e14 prophage; putative DNA-binding transcriptional regulator	0.0079	0.8310
JW1134	ymfM	e14 prophage; uncharacterized protein	0.0244	0.1151
JW1135	ymfN	pseudogene, phage terminase protein A family, e14 prophage	-0.0042	0.9175
JW1136	ymfR	e14 prophage; uncharacterized protein	0.0121	0.2652
JW1137	ymfO	pseudogene, portal protein family, e14 prophage	0.0064	0.7387
JW1139	ymfQ	prohage e14 tail protein homolog	-0.0320	0.0332
JW1140	ycfK	e14 prophage; uncharacterized protein	0.0769	0.0004
JW1142	tfaE	e14 prophage; putative tail fiber assembly protein	-0.0023	0.8978

JW1144	pin	e14 prophage; site-specific DNA recombinase	0.0549	0.2688
JW1145	mcrA	putative 5-methylcytosine/5-hydroxymethylcytosine-specific restriction nuclease; 5-methylcytosine DNA binding protein	-0.1291	0.0548
JW1147	elbA	RpoS stabilzer during Mg starvation, anti-RssB factor	-0.0424	0.0762
JW1148	ycgX	DUF1398 family protein	0.0053	0.8375
JW1149	ycgE	repressor of blue light-responsive genes	-0.0437	0.1322
JW1150	ycgF	anti-repressor for YcgE, blue light-responsive; FAD-binding; inactive c-di-GMP phosphodiesterase-like EAL domain protein	0.0392	0.1744
JW1151	ycgZ	RcsB connector protein for regulation of biofilm and acid-resistance	0.0240	0.4690
JW1152	ymgA	RcsB connector protein for regulation of biofilm	0.0086	0.6660
JW1153	ymgB	RcsB connector protein for regulation of biofilm and acid-resistance	0.0488	0.1130
JW1154	ymgC	blue light, low temperature and stress induced protein	-0.0162	0.5541
JW1156	ymgF	inner membrane division septum protein	-0.0124	0.3536
JW1162	ycgI	pseudogene	0.0183	0.0792
JW1165	minC	inhibitor of FtsZ ring polymerization	-0.3693	0.0000
JW1166	ycgJ	uncharacterized protein	-0.0656	0.0222
JW1167	ycgK	periplasmic inhibitor of g-type lysozyme	-0.1948	0.0640
JW1168	ycgL	UPF0745 family protein	0.0175	0.7061
JW1169	ycgM	putative isomerase/hydrolase	-0.0006	0.9916
JW1173	umuC	translesion error-prone DNA polymerase V subunit; DNA polymerase activity	-0.0076	0.7698
JW1175	nhaB	sodium:proton antiporter	0.1842	0.0116
JW1176	fadR	fatty acid metabolism regulon transcriptional regulator	-0.0973	0.1958
JW1177	ycgB	SpoVR family stationary phase protein	-0.0531	0.2088
JW1178	dadA	D-amino acid dehydrogenase	0.0248	0.5290
JW1179	dadX	alanine racemase, catabolic, PLP-binding	0.0125	0.7771
JW1181	ldcA	murein tetrapeptide carboxypeptidase; LD-carboxypeptidase A	0.3106	0.0000
JW1183	ycgR	flagellar velocity braking protein, c-di-GMP-regulated	0.0705	0.0918
JW1184	ymgE	UPF0410 family putative inner membrane protein	-0.0541	0.2469
JW1185	ycgY	uncharacterized protein	-0.0222	0.4117
JW1186	treA	periplasmic trehalase	0.0499	0.0069

JW1193	ycgV	putative adhesin	-0.1179	0.1123
JW1194	ychF	catalase inhibitor protein; ATPase, K+-dependent, ribosome-associated	-0.0116	0.5048
JW1196	ychH	DUF2583 family putative inner membrane protein	-0.0009	0.9578
JW1204	ychQ	SIRB family inner membrane protein	-0.0602	0.0022
JW1205	ychA	transglutaminase-like TPR-repeat protein	-0.0022	0.9529
JW1207	chaA	calcium/sodium:proton antiporter	-0.0401	0.7227
JW1208	chaB	cation transport regulator	-0.0633	0.5163
JW1209	chaC	cation transport regulator	-0.0134	0.7599
JW1211	ychP	putative invasin	0.0262	0.5182
JW1212	narL	response regulator in two-component regulatory system with NarX	0.0168	0.5189
JW1213	narX	sensory histidine kinase in two-component regulatory system with NarL	-0.0552	0.2982
JW1214	narK	nitrate/nitrite transporter	-0.0264	0.3088
JW1215	narG	nitrate reductase 1, alpha subunit	-0.0483	0.0342
JW1216	narH	nitrate reductase 1, beta (Fe-S) subunit	0.0078	0.7501
JW1217	narJ	molybdenum-cofactor-assembly chaperone delta subunit of nitrate reductase 1	0.1565	0.0287
JW1218	narI	nitrate reductase 1, gamma (cytochrome b(NR)) subunit	-0.0411	0.3333
JW1219	tpr	protamine-like protein	0.0145	0.6370
JW1220	purU	formyltetrahydrofolate hydrolase	0.2370	0.0001
JW1221	ychJ	UPF0225 family protein	0.0691	0.4765
JW1222	rssA	putative patatin-like family phospholipase	-0.0665	0.0293
JW1223	rssB	PcnB-degradosome interaction factor; response regulator	0.1137	0.0002
JW1225	hns	global DNA-binding transcriptional dual regulator H-NS	-0.1013	0.0005
JW1226	tdk	thymidine kinase/deoxyuridine kinase	0.1006	0.0050
JW1228	adhE	fused acetaldehyde-CoA dehydrogenase/iron-dependent alcohol dehydrogenase/pyruvate-formate lyase deactivase	0.0438	0.3920
JW1229	ychE	UPF0056 family inner membrane protein	-0.0909	0.0240
JW1235	oppA	oligopeptide ABC transporter periplasmic binding protein	0.0038	0.7949
JW1236	oppB	oligopeptide ABC transporter permease	0.0247	0.2818
JW1237	oppC	oligopeptide ABC transporter permease	-0.0154	0.6764
JW1238	oppD	oligopeptide ABC transporter ATPase	0.0304	0.4045
JW1239	oppF	oligopeptide ABC transporter ATPase	-0.0006	0.9836

JW1240	yciU	UPF0263 family protein	-0.0301	0.1265
JW1241	cls	cardiolipin synthase 1	-0.0674	0.0022
JW1242	kch	voltage-gated potassium channel	0.0630	0.0652
JW1243	yciI	putative DGPF domain-containing enzyme	-0.0420	0.0650
JW1245	yciA	acyl-CoA esterase	-0.0817	0.0023
JW1246	yciB	IspA family inner membrane protein	0.0603	0.0923
JW1247	yciC	UPF0259 family inner membrane protein	-0.0648	0.0006
JW1248	ompW	outer membrane protein W	0.1261	0.0034
JW1249	yciE	putative rubrerythrin/ferritin-like metal-binding protein	-0.0363	0.0419
JW1250	yciF	putative rubrerythrin/ferritin-like metal-binding protein	-0.0047	0.8375
JW1251	yciG	KGG family protein	-0.0015	0.9536
JW1252	trpA	tryptophan synthase, alpha subunit	-0.3403	0.1171
JW1253	trpB	tryptophan synthase, beta subunit	0.2808	0.0008
JW1254	trpC	indole-3-glycerolphosphate synthetase and N-(5-phosphoribosyl)anthranilate isomerase	0.0207	0.7431
JW1255	trpD	fused glutamine amidotransferase (component II) of anthranilate synthase/anthranilate phosphoribosyl transferase	0.2617	0.0029
JW1256	trpE	component I of anthranilate synthase	-0.0849	0.0521
JW1257	trpL	trp operon leader peptide	-0.0079	0.6706
JW1258	yciV	PHP domain protein	0.0243	0.3601
JW1261	rluB	23S rRNA pseudouridine(2605) synthase	0.0074	0.7791
JW1262	btuR	cob(I)yrinic acid a,c-diamide adenosyltransferase	0.0398	0.1206
JW1263	yciK	putative EmrKY-TolC system oxoacyl-(acyl carrier protein) reductase	-0.1168	0.0000
JW1264	sohB	inner membrane protein, S49 peptidase family protein	0.0473	0.1795
JW1265	yciN	DUF2498 protein YciN	-0.0153	0.3923
JW1267	cysB	N-acetylserine-responsive cysteine regulon transcriptional activator; autorepressor	0.0140	0.5768
JW1268	acnA	aconitate hydratase 1; aconitase A	-0.0318	0.1385
JW1270	pgpB	phosphatidylglycerophosphatase B	0.0435	0.0268
JW1271	yciS	DUF1049 family inner membrane protein, function unknown	-0.0508	0.0879
JW1272	yciM	LPS regulatory protein; putative modulator of LpxC proteolysis	-0.0622	0.0910
JW1273	pyrF	orotidine-5'-phosphate decarboxylase	0.1547	0.0016
JW1274	yciH	initiation factor function partial mimic, SUI1 family	0.0228	0.2904
JW1275	osmB	osmotically and stress inducible lipoprotein	0.1322	0.0017
JW1276	yciT	global regulator of transcription; DeoR family	0.2124	0.0008

JW1278	gmr	cyclic-di-GMP phosphodiesterase; csgD regulator; modulator of RNase II stability	-0.0146	0.6926
JW1279	rnb	ribonuclease II	0.0326	0.0125
JW1282	ycjD	DUF559 family endonuclease-related protein	0.0000	0.9986
JW1283	sapF	antimicrobial peptide ABC transporter ATPase	-0.0370	0.1225
JW1284	sapD	antimicrobial peptide ABC transporter ATPase	0.0355	0.4976
JW1285	sapC	antimicrobial peptide transport ABC transporter permease	0.0673	0.0416
JW1286	sapB	antimicrobial peptide transport ABC transporter permease	-0.0054	0.8001
JW1287	sapA	antimicrobial peptide transport ABC transporter periplasmic binding protein	-0.0817	0.0751
JW1288	ymjA	DUF2543 family protein	0.0173	0.1633
JW1289	puuP	putrescine importer	-0.0037	0.9482
JW1291	puuD	gamma-glutamyl-gamma-aminobutyrate hydrolase	0.0670	0.1856
JW1292	puuR	repressor for the divergent puu operons, putrescine inducible	-0.0821	0.0200
JW1293	puuC	gamma-glutamyl-gamma-aminobutyraldehyde dehydrogenase; succinate semialdehyde dehydrogenase	0.0830	0.0112
JW1294	puuB	gamma-glutamylputrescine oxidoreductase	-0.0291	0.1614
JW1295	puuE	4-aminobutyrate aminotransferase, PLP-dependent	0.0054	0.9046
JW1296	pspF	psp operon transcriptional activator	0.0399	0.1914
JW1297	pspA	regulatory protein for phage-shock-protein operon	-0.1176	0.0170
JW1298	pspB	psp operon transcription co-activator	0.0101	0.7089
JW1299	pspC	psp operon transcription co-activator	0.0287	0.3307
JW1300	pspD	peripheral inner membrane phage-shock protein	-0.0474	0.2926
JW1301	pspE	thiosulfate:cyanide sulfurtransferase (rhodanese)	0.0089	0.7322
JW1302	ycjM	alpha amylase catalytic domain family protein	-0.0319	0.3529
JW1303	ycjN	putative ABC sugar transporter periplasmic binding protein	-0.0030	0.9577
JW1304	ycjO	putative sugar ABC transporter permease	0.1201	0.2984
JW1305	ycjP	putative sugar ABC transporter permease	-0.0739	0.0604
JW1306	ycjQ	putative Zn-dependent NAD(P)-binding oxidoreductase	0.0173	0.4068
JW1308	ycjS	putative NADH-binding oxidoreductase	-0.0076	0.8406
JW1309	ycjT	putative family 65 glycosyl hydrolase	-0.0596	0.0978

JW1310	ycjU	beta-phosphoglucomutase	-0.1092	0.3932
JW1311	ycjV	pseudogene	0.0687	0.0659
JW1312	ompG	outer membrane porin G	0.2035	0.0060
JW1313	ycjW	LacI family putative transcriptional repressor	0.0383	0.2253
JW1314	ycjX	DUF463 family protein, puatative P-loop NTPase	0.0591	0.2420
JW1315	ycjF	UPF0283 family inner membrane protein	0.0093	0.7069
JW1316	tyrR	aromatic amino acid biosynthesis and transport regulon transcriptional regulator; autorepressor; ATPase; phosphatase	-0.0110	0.5756
JW1317	tpx	lipid hydroperoxide peroxidase	-0.0268	0.1560
JW1318	ycjG	L-Ala-D/L-Glu epimerase	-0.0120	0.3970
JW1319	mpaA	murein peptide amidase A	0.0673	0.0449
JW1321	ycjZ	murein peptide degradation regulator	-0.0501	0.0285
JW1322	mppA	murein tripeptide (L-ala-gamma-D-glutamyl-meso-DAP) transporter subunit	-0.0793	0.0027
JW1326	ynaJ	DUF2534 family putative inner membrane protein	-0.0858	0.0331
JW1327	uspE	stress-induced protein	-0.0155	0.5843
JW1328	fnr	oxygen-sensing anaerobic growth regulon transcriptional regulator FNR; autorepressor	-0.1486	0.0028
JW1329	ogt	O-6-alkylguanine-DNA:cysteine-protein methyltransferase	0.1437	0.0005
JW1331	abgB	p-aminobenzoyl-glutamate hydrolase, B subunit	0.0189	0.0902
JW1333	abgR	putative DNA-binding transcriptional regulator of abgABT operon	-0.0678	0.0004
JW1334	ydaL	DNA endonuclease	-0.0682	0.0159
JW1336	ydaN	putative Zn(II) transporter	0.0348	0.1822
JW1337	dbpA	ATP-dependent RNA helicase, specific for 23S rRNA	-0.1538	0.2165
JW1338	ydaO	tRNA s(2)C32 thioltransferase, iron–sulfur cluster protein	0.1680	0.0905
JW1339	intR	Rac prophage; integrase	-0.0252	0.2626
JW1341	ydaC	DUF1187 family protein, Rac prophage; putative double-strand break reduction protein	-0.0512	0.0854
JW1343	recT	Rac prophage; recombination and repair protein	0.2470	0.0221
JW1344	recE	Rac prophage; exonuclease VIII, 5' to 3' specific dsDNA exonuclease	0.0858	0.0432
JW1345	racC	Rac prophage; uncharacterized protein	-0.0252	0.4458
JW1346	ydaE	conserved protein, Rac prophage	0.0866	0.0000
JW1347	kil	killing protein, Rac prophage; FtsZ inhibitor protein	0.0543	0.0163

JW1349	ydaF	uncharacterized protein, Rac prophage	-0.0047	0.9678
JW1352	ydaS	Rac prophage; putative DNA-binding transcriptional regulator	0.2164	0.0000
JW1353	ydaT	Rac prophage; uncharacterized protein	-0.0234	0.1925
JW1354	ydaU	Rac prophage; conserved protein	0.0032	0.8309
JW1355	ydaV	Rac prophage; putative DNA replication protein	0.0615	0.3359
JW1358	trkG	Rac prophage; potassium transporter subunit	-0.0991	0.0781
JW1359	ynaK	Rac prophage; conserved protein	-0.0329	0.1100
JW1361	ynaA	Rac prophage; pseudogene, tail protein family	-0.0285	0.2635
JW1366	stfR	Rac prophage; putative tail fiber protein	0.0287	0.1034
JW1367	tfaR	Rac prophage; putative tail fiber assembly protein	0.0400	0.1029
JW1368	pinR	Rac prophage; putative site-specific recombinase	-0.0741	0.0808
JW1369	ynaE	cold shock protein, Rac prophage	-0.0020	0.8735
JW1370	uspF	stress-induced protein, ATP-binding protein	0.0471	0.0571
JW1371	ompN	outer membrane pore protein N, non-specific	-0.0075	0.8032
JW1372	ydbK	pyruvate-flavodoxin oxidoreductase	-0.0103	0.6843
JW1374	hslJ	heat-inducible lipoprotein involved in novobiocin resistance	0.0235	0.6204
JW1375	ldhA	fermentative D-lactate dehydrogenase, NAD-dependent	-0.0388	0.0406
JW1376	ydbH	putative membrane-anchored protein, function unknown	0.0326	0.2856
JW1377	ynbE	lipoprotein	-0.0576	0.0106
JW1379	feaR	transcriptional activator for tynA and feaB	0.0366	0.2357
JW1380	feaB	phenylacetaldehyde dehydrogenase	0.0463	0.1498
JW1381	tynA	tyramine oxidase, copper-requiring	0.0920	0.0010
JW1382	maoC	oxepin-CoA hydrolase and 3-oxo-5,6-dehydrosuberyl-CoA semialdehyde dehydrogenase	-0.0941	0.0033
JW1383	paaA	ring 1,2-phenylacetyl-CoA epoxidase subunit	-0.1905	0.0692
JW1384	paaB	putative ring 1,2-phenylacetyl-CoA epoxidase subunit	0.0114	0.9013
JW1385	paaC	ring 1,2-phenylacetyl-CoA epoxidase subunit	0.1105	0.0656
JW1387	paaE	ring 1,2-phenylacetyl-CoA epoxidase, NAD(P)H oxidoreductase component	0.0748	0.0313
JW1388	paaF	2,3-dehydroadipyl-CoA hydratase	0.0892	0.1442
JW1389	paaG	1,2-epoxyphenylacetyl-CoA isomerase, oxepin-CoA-forming	0.0194	0.6252
JW1390	paaH	3-hydroxyadipyl-CoA dehydrogenase, NAD+-dependent	-0.1078	0.0042

JW1392	paaJ	3-oxoadipyl-CoA/3-oxo-5,6-dehydrosuberyl-CoA thiolase	0.0827	0.0056
JW1394	paaX	transcriptional repressor of phenylacetic acid degradation paa operon, phenylacetyl-CoA inducer	0.0221	0.7524
JW1395	paaY	thioesterase required for phenylacetic acid degradation; trimeric; phenylacetate regulatory and detoxification protein; hexapeptide repeat protein	0.0709	0.0074
JW1402	ydbA	Putative exported protein	0.0373	0.2552
JW1403	ydbC	pyridoxine 4-dehydrogenase	0.0617	0.2376
JW1405	ynbA	inner membrane protein	-0.0335	0.3491
JW1406	ynbB	putative CDP-diglyceride synthase	0.0143	0.6866
JW1407	ynbC	putative esterase	0.0013	0.9648
JW1408	ynbD	putative phosphatase inner membrane protein	0.0206	0.3165
JW1409	azoR	NADH-azoreductase, FMN-dependent	0.0528	0.0390
JW1411	ydcF	DUF218 superfamily protein, SAM-binding	-0.0111	0.6581
JW1412	aldA	aldehyde dehydrogenase A, NAD-linked	0.0571	0.0036
JW1413	gapC	Uncharacterized protein	-0.0394	0.5064
JW1416	ydcA	putative periplasmic protein	0.0532	0.0196
JW1417	trg	methyl-accepting chemotaxis protein III, ribose and galactose sensor receptor	0.0385	0.3236
JW1419	ydcJ	putative metalloenzyme	0.0119	0.7184
JW1420	mdoD	OPG biosynthetic periplasmic protein	0.0202	0.6792
JW1423	rimL	ribosomal-protein-L7/L12-serine acetyltransferase	0.1999	0.0380
JW1424	ydcK	uncharacterized protein	-0.0174	0.3937
JW1425	tehA	potassium-tellurite ethidium and proflavin transporter	0.0575	0.1079
JW1426	tehB	tellurite, selenium methyltransferase, SAM-dependent; tellurite, selenium resistance protein	-0.0348	0.1905
JW1427	ydcL	lipoprotein	0.0270	0.3483
JW1430	ydcN	putative DNA-binding transcriptional regulator	-0.0028	0.9595
JW1431	ydcP	putative peptidase	0.0191	0.4411
JW1432	yncJ	uncharacterized protein	-0.2186	0.0596
JW1433	ydcQ	antitoxin for the HicAB toxin-antitoxin system	0.0052	0.8184
JW1434	ydcR	putative DNA-binding transcriptional regulator and putative aminotransferase	0.0427	0.4032
JW1435	ydcS	putative ABC transporter periplasmic binding protein	0.1165	0.0036
JW1436	ydcT	putative ABC transporter ATPase	0.0292	0.5275
JW1437	ydcU	putative ABC transporter permease	0.0065	0.8722

JW1438	ydcV	putative ABC transporter permease	-0.1655	0.0013
JW1439	ydcW	gamma-aminobutyraldehyde dehydrogenase	0.0122	0.5799
JW1441	ydcY	DUF2526 family protein	0.0059	0.7635
JW1442	ydcZ	DUF606 family inner membrane protein	0.0493	0.0242
JW1445	yncC	colanic acid and biofilm gene transcriptional regulator, MqsR-controlled	-0.0844	0.0434
JW1446	yncD	putative iron outer membrane transporter	-0.0181	0.4755
JW1447	yncE	ATP-binding protein, periplasmic, function unknown	0.0011	0.9582
JW1449	yncG	glutathione S-transferase homolog	-0.0416	0.0840
JW1451	rhsE	pseudogene, Rhs family	0.0148	0.6993
JW1452	ydcD	putative immunity protein for RhsE	-0.0027	0.8916
JW1453	yncI	pseudogene	-0.0398	0.1510
JW1455	ydcC	H repeat-associated putative transposase	0.0650	0.0002
JW1456	ydcE	4-oxalocrotonate tautomerase	-0.0020	0.9463
JW1457	yddH	flavin reductase like-protein	-0.0904	0.0676
JW1458	nhoA	N-hydroxyarylamine O-acetyltransferase	-0.0658	0.0801
JW1459	yddE	PhzC-PhzF family protein	0.0082	0.7042
JW1460	narV	nitrate reductase 2 (NRZ), gamma subunit	-0.0045	0.8730
JW1461	narW	nitrate reductase 2 (NRZ), delta subunit (assembly subunit)	0.0051	0.8597
JW1462	narY	nitrate reductase 2 (NRZ), beta subunit	0.0229	0.6023
JW1463	narZ	nitrate reductase 2 (NRZ), alpha subunit	0.0425	0.3742
JW1464	narU	nitrate/nitrite transporter	0.0569	0.2438
JW1466	yddJ	Uncharacterized protein	0.0743	0.0075
JW1467	yddK	pseudogene, leucine-rich protein	-0.0449	0.0895
JW1468	yddL	putative lipoprotein	-0.0162	0.8199
JW1469	yddG	aromatic amino acid exporter	0.0713	0.0139
JW1471	fdnH	formate dehydrogenase-N, Fe-S (beta) subunit, nitrate-inducible	-0.0210	0.2320
JW1472	fdnI	formate dehydrogenase-N, cytochrome B556 (gamma) subunit, nitrate-inducible	-0.0033	0.9069
JW1474	adhP	ethanol-active dehydrogenase/acetaldehyde-active reductase	-0.0448	0.3758
JW1477	osmC	lipoyl-dependent Cys-based peroxidase, hydroperoxide resistance; salt-shock inducible membrane protein; peroxiredoxin	0.1115	0.0020
JW1478	ddpF	D,D-dipeptide ABC transporter ATPase	0.1300	0.0004
JW1479	ddpD	D,D-dipeptide ABC transporter ATPase	0.1150	0.1572
JW1480	ddpC	D,D-dipeptide ABC transporter permease	0.1175	0.4346
JW1481	ddpB	D,D-dipeptide ABC transporter permease	-0.0468	0.2877

JW1483	ddpX	D-ala-D-ala dipeptidase, Zn-dependent	-0.0656	0.0406
JW1484	dos	oxygen sensor, c-di-GMP phosphodiesterase, heme-regulated; cold- and stationary phase-induced bioflim regulator	-0.0223	0.3260
JW1486	yddW	liprotein, glycosyl hydrolase homolog	0.0042	0.9289
JW1487	gadC	glutamate:gamma-aminobutyric acid antiporter	-0.1261	0.1392
JW1488	gadB	glutamate decarboxylase B, PLP-dependent	-0.0116	0.8282
JW1489	pqqL	putative periplasmic M16 family zinc metalloendopeptidase	-0.3706	0.0095
JW1490	yddB	putative TonB-dependent outer membrane receptor	-0.0473	0.1938
JW1492	ydeM	putative YdeN-specific sulfatase-maturating enzyme	-0.0306	0.5958
JW1494	ydeO	UV-inducible global regulator, EvgA-, GadE-dependent	0.1164	0.0001
JW1495	ydeP	putative oxidoreductase	0.0010	0.9094
JW1496	ydeQ	putative fimbrial-like adhesin protein	-0.0544	0.0432
JW1497	ydeR	putative fimbrial-like adhesin protein	0.0919	0.0006
JW1498	ydeS	putative fimbrial-like adhesin protein	0.0029	0.8755
JW1499	ydeT	pseudogene	0.0039	0.9347
JW1500	hipA	inactivating GltX kinase facilitating persister formation; toxin of HipAB TA pair; autokinase	0.0273	0.2617
JW1501	hipB	antitoxin of HipAB toxin-antitoxin system	0.1143	0.0001
JW1502	ydeU	Uncharacterized protein	-0.0654	0.0944
JW1503	ydeK	pseudogene, AidA homolog	-0.0646	0.0001
JW1504	ydeV	autoinducer-2 (AI-2) kinase	0.0960	0.0005
JW1505	ydeW	lsr operon transcriptional repressor	0.0159	0.4853
JW1506	ego	autoinducer 2 import ATP-binding protein	0.0756	0.1785
JW1507	lsrC	autoinducer 2 import system permease protein	-0.0387	0.2066
JW1508	lsrD	autoinducer 2 import system permease protein	0.0157	0.4888
JW1509	lsrB	autoinducer 2-binding protein	-0.1057	0.0268
JW1510	lsrF	putative autoinducer-2 (AI-2) aldolase	-0.3726	0.0014
JW1511	lsrG	autoinducer-2 (AI-2) degrading protein LsrG	-0.0236	0.1367
JW1512	tam	trans-aconitate methyltransferase	-0.0479	0.0287
JW1514	uxaB	altronate oxidoreductase, NAD-dependent	-0.0814	0.0080
JW1516	yneG	DUF4186 family protein	-0.0250	0.4486
JW1517	yneH	glutaminase 2	0.0020	0.9637
JW1519	yneJ	putative DNA-binding transcriptional regulator	-0.0531	0.0374
JW1520	yneK	uncharacterized protein	-0.0438	0.1145

JW1521	ydeA	arabinose efflux transporter, arabinose-inducible	0.0123	0.6403
JW1522	marC	UPF0056 family inner membrane protein	-0.0185	0.2949
JW1525	marB	periplasmic mar operon regulator	-0.0671	0.0436
JW1527	ydeE	putative transporter	-0.0533	0.0064
JW1528	ydeH	diguanylate cyclase, zinc-sensing	0.0191	0.6460
JW1529	ydeI	hydrogen peroxide resistance OB fold protein; putative periplasmic protein	0.0301	0.0110
JW1530	ydeJ	inactive PncC family protein	0.0768	0.0199
JW1531	dcp	dipeptidyl carboxypeptidase II	-0.0135	0.7332
JW1532	ydfG	NADP-dependent 3-hydroxy acid dehydrogenase; malonic semialdehyde reductase	-0.0218	0.4082
JW1533	ydfH	transcriptional repressor for rspAB	-0.0477	0.0307
JW1534	ydfZ	selenoprotein, function unknown	0.0039	0.8888
JW1535	ydfI	putative NAD-dependent D-mannonate oxidoreductase	0.0016	0.9618
JW1536	ydfJ	pseudogene, MFS transporter family; interrupted by Qin prophage	0.0514	0.0608
JW1537	ydfK	cold shock protein, function unknown, Qin prophage	0.0419	0.2412
JW1538	pinQ	Qin prophage; putative site-specific recombinase	0.0076	0.7003
JW1539	tfaQ	Qin prophage; putative tail fibre assembly protein	-0.0757	0.0290
JW1540	stfQ	Qin prophage; putative side tail fibre assembly protein	0.0502	0.0223
JW1541	nohA	pseudogene, Qin prophage; Phage DNA packaging protein Nu1 family	-0.1494	0.0010
JW1545	ydfP	Qin prophage; Rz-like protein	-0.0072	0.7374
JW1546	ydfQ	Qin prophage; putative lysozyme	0.0240	0.2452
JW1547	ydfR	Qin prophage; DUF1327 family protein	0.0082	0.6511
JW1549	cspB	Qin prophage; cold shock protein	-0.0243	0.1268
JW1550	cspF	Qin prophage; cold shock protein	-0.0714	0.0905
JW1551	ydfT	Qin prophage; putative antitermination protein Q	0.0987	0.0006
JW1553	rem	Qin prophage; uncharacterized protein	-0.0385	0.4730
JW1554	hokD	Qin prophage; small toxic polypeptide	-0.1910	0.0067
JW1555	relE	Qin prophage; toxin of the RelE-RelB toxin-antitoxin system	0.0267	0.2330
JW1556	relB	antitoxin of the RelE-RelB toxin-antitoxin syste; transcriptional repressor	0.0584	0.1383
JW1557	ydfV	Qin prophage; uncharacterized protein	0.0431	0.0819
JW1558	flxA	Qin prophage; uncharacterized protein	0.1194	0.0088
JW1559	ydfW	pseudogene, integrase fragment, Qin prophage	0.1457	0.0037
JW1560	ydfX	pseudogene, Qin prophage	0.0405	0.1474
JW1561	dicC	Qin prophage; DNA-binding transcriptional regulator for DicB	0.2209	0.0151

JW1563	ydfA	Qin prophage; DUF1391 family protein	0.0001	0.9952
JW1565	ydfC	Qin prophage; uncharacterized protein	-0.0189	0.4124
JW1566	dicB	Qin prophage; cell division inhibition protein	0.0814	0.1250
JW1567	ydfD	Qin prophage; DUF1482 family protein	-0.0131	0.5511
JW1568	ydfE	Qin prophage; pseudogene	-0.0078	0.7687
JW1571	intQ	pseudogene, Qin prophage; phage integrase family	0.0161	0.4884
JW1572	rspB	putative Zn-dependent NAD(P)-binding oxidoreductase	0.0090	0.7569
JW1573	rspA	bifunctional D-altronate/D-mannonate dehydratase	-0.0172	0.6832
JW1574	ynfA	UPF0060 family inner membrane protein	0.0011	0.9644
JW1575	ynfB	UPF0482 family putative periplasmic protein	0.0541	0.2658
JW1576	speG	spermidine N(1)-acetyltransferase	0.0399	0.7060
JW1579	ynfE	putative selenate reductase, periplasmic	-0.0173	0.3684
JW1581	ynfG	oxidoreductase, Fe-S subunit	0.0119	0.5237
JW1586	dgsA	glucosamine anaerobic growth regulon transcriptional repressor; autorepressor	0.0117	0.1452
JW1587	ynfL	LysR family putative transcriptional regulator	0.0188	0.6686
JW1588	ynfM	putative arabinose efflux transporter	0.0385	0.4674
JW1590	ydgD	putative peptidase	0.0500	0.1169
JW1591	mdtI	multidrug efflux system transporter	0.0266	0.0869
JW1592	mdtJ	multidrug efflux system transporter	-0.0227	0.2674
JW1593	ydgG	pheromone AI-2 transporter	-0.1575	0.1928
JW1594	pntB	pyridine nucleotide transhydrogenase, beta subunit	0.0343	0.3995
JW1595	pntA	pyridine nucleotide transhydrogenase, alpha subunit	-0.0614	0.0336
JW1596	ydgH	DUF1471 family periplasmic protein	-0.1508	0.0027
JW1597	ydgI	putative arginine/ornithine antiporter transporter	-0.0741	0.2397
JW1598	folM	dihydromonapterin reductase, NADPH-dependent; dihydrofolate reductase isozyme	0.0685	0.0149
JW1599	ydgC	GlpM family inner membrane protein	0.0000	0.9990
JW1600	rstA	response regulator of RstAB two-component system	0.0847	0.0017
JW1601	rstB	sensory histidine kinase of RstAB two-component system	0.0015	0.9254
JW1602	tus	inhibitor of replication at Ter, DNA-binding protein	0.0489	0.0241
JW1603	fumC	fumarate hydratase (fumarase C),aerobic Class II	-0.0088	0.8098
JW1604	fumA	fumarate hydratase (fumarase A), aerobic Class I	-0.0152	0.7502
JW1605	manA	mannose-6-phosphate isomerase	-0.0026	0.9085

JW1606	ydgA	DUF945 family protein	-0.0718	0.0300
JW1607	uidC	putative outer membrane porin for beta-glucuronides porin protein	0.0856	0.0025
JW1608	uidB	glucuronide transporter	0.0425	0.1476
JW1609	uidA	beta-D-glucuronidase	0.0392	0.1623
JW1610	uidR	transcriptional repressor	-0.0391	0.0507
JW1611	hdhA	7-alpha-hydroxysteroid dehydrogenase, NAD-dependent	0.1152	0.2301
JW1612	malI	transcriptional repressor of Mal regulon	-0.0734	0.0323
JW1613	malX	maltose and glucose-specific PTS enzyme IIB component and IIC component	0.0196	0.7693
JW1614	malY	PLP-dependent beta-cystathionase and maltose regulon regulator	0.0248	0.3275
JW1615	add	adenosine deaminase	-0.2906	0.0165
JW1617	ydgT	nucleoid-associated oriC-binding protein; H-NS and StpA stabilizing factor	-0.0413	0.1656
JW1619	rsxA	SoxR iron-sulfur cluster reduction factor component; inner membrane protein of electron transport complex	-0.0263	0.2175
JW1620	rsxB	SoxR iron-sulfur cluster reduction factor component; putative iron-sulfur protein	0.1415	0.1364
JW1621	rsxC	SoxR iron-sulfur cluster reduction factor component; putative membrane-associated NADH oxidoreductase of electron transport complex	-0.0566	0.0021
JW1622	rsxD	SoxR iron-sulfur cluster reduction factor component; putative membrane protein of electron transport complex	-0.0152	0.4641
JW1623	rsxG	SoxR iron-sulfur cluster reduction factor component; putative membrane protein of electron transport complex	-0.0066	0.9198
JW1624	rsxE	SoxR iron-sulfur cluster reduction factor component; electron transport inner membrane NADH-quinone reductase	-0.0258	0.1731
JW1625	nth	DNA glycosylase and apyrimidinic (AP) lyase (endonuclease III)	0.0013	0.9409
JW1626	ydgR	dipeptide and tripeptide permease A	-0.0031	0.9005
JW1627	gst	glutathionine S-transferase	-0.0675	0.0018
JW1628	pdxY	pyridoxamine kinase	0.0437	0.2923
JW1630	pdxH	pyridoxine 5'-phosphate oxidase	-0.1866	0.0235
JW1631	ydhA	inhibitor of c-type lysozyme, membrane-bound; putative lipoprotein	0.0513	0.1319
JW1632	ydhH	anhydro-N-acetylmuramic acid kinase	0.0302	0.3680
JW1633	slyB	outer membrane lipoprotein	0.0536	0.0161

JW1635	ydhI	DUF1656 family putative inner membrane efflux pump associated protein	0.0453	0.0248
JW1636	ydhJ	putative membrane fusion protein (MFP) of YdhJK efflux pump	0.0525	0.2429
JW1637	ydhK	putative efflux protein (PET) component of YdhJK efflux pump	-0.0122	0.6787
JW1638	sodC	superoxide dismutase, Cu, Zn, periplasmic	0.0426	0.0023
JW1639	ydhF	putative oxidoreductase	0.0237	0.2060
JW1642	nemA	chromate reductase, quinone reductase, FMN-linked; N-Ethylmaleimide reductase; old yellow enzyme	-0.0067	0.8147
JW1643	gloA	glyoxalase I, Ni-dependent	0.0145	0.4765
JW1644	rnt	RNase T; exoribonuclease T; structured DNA 3' exonuclease; RNA processing; DNA repair	-0.0475	0.3271
JW1645	lhr	putative ATP-dependent helicase	-0.0174	0.6065
JW1646	ydhD	glutaredoxin-4	-0.0861	0.1971
JW1648	sodB	superoxide dismutase, Fe	-0.0436	0.3129
JW1649	ydhP	putative MFS transporter, inner membrane protein	0.0209	0.3539
JW1650	purR	transcriptional repressor, hypoxanthine-binding	0.0622	0.0233
JW1651	ydhB	LysR family putative transcriptional regulator	-0.1015	0.0086
JW1652	ydhC	putative arabinose efflux transporter	0.0673	0.1856
JW1653	cfa	cyclopropane fatty acyl phospholipid synthase, SAM-dependent	0.0818	0.1249
JW1655	mdtK	multidrug efflux system transporter	-0.0106	0.7530
JW1656	ydhQ	autotransporter adhesin-related protein	0.0369	0.1033
JW1657	ydhR	putative monooxygenase	-0.0057	0.8775
JW1659	ydhT	FNR, Nar, NarP-regulated protein; putative subunit of YdhYVWXUT oxidoreductase complex	-0.0551	0.0051
JW1660	ydhU	putative cytochrome b subunit of YdhYVWXUT oxidoreductase complex	0.0028	0.9263
JW1662	ydhW	FNR, Nar, NarP-regulated protein; putative subunit of YdhYVWXUT oxidoreductase complex	-0.0204	0.4246
JW1664	ydhY	putative 4Fe-4S ferridoxin-type protein; FNR, Nar, NarP-regulated protein; putative subunit of YdhYVWXUT oxidoreductase complex	-0.1183	0.1790
JW1665	ydhZ	fumarase D	0.0317	0.0591
JW1666	pykF	pyruvate kinase I	0.0100	0.9171

JW1667	lpp	murein lipoprotein	-0.3235	0.0014
JW1668	ynhG	murein L,D-transpeptidase	0.0008	0.9885
JW1669	sufE	sulfur acceptor protein	-0.0246	0.3132
JW1670	sufS	cysteine desulfurase, stimulated by SufE; selenocysteine lyase, PLP-dependent	0.0005	0.9786
JW1671	sufD	component of SufBCD Fe-S cluster assembly scaffold	0.1114	0.0015
JW1672	sufC	SufBCD Fe-S cluster assembly scaffold protein, ATP-binding protein	0.1188	0.0091
JW1674	sufA	Fe-S cluster assembly protein	0.0786	0.0957
JW1675	ydiH	uncharacterized protein	0.0117	0.5723
JW1676	ydiI	1,4-dihydroxy-2-naphthoyl-CoA hydrolase	-0.0083	0.7754
JW1677	ydiJ	putative FAD-linked oxidoreductase	-0.0547	0.0388
JW1678	ydiK	UPF0118 family inner membrane protein	0.0084	0.8030
JW1682	ydiB	quinate/shikimate 5-dehydrogenase, NAD(P)-binding	-0.0163	0.6885
JW1683	aroD	3-dehydroquinate dehydratase	0.1933	0.0544
JW1684	ydiF	putative acetyl-CoA:acetoacetyl-CoA transferase: alpha subunit/beta subunit	-0.0270	0.4113
JW1686	ydiP	putative DNA-binding transcriptional regulator	-0.0038	0.9226
JW1688	ydiR	putative electron transfer flavoprotein, FAD-binding subunit	0.1448	0.0065
JW1689	ydiS	putative oxidoreductase	-0.0049	0.8493
JW1692	pps	phosphoenolpyruvate synthase	-0.0102	0.8138
JW1693	ydiA	PEP synthase kinase and PEP synthase pyrophosphorylase	-0.0029	0.8667
JW1694	aroH	3-deoxy-D-arabino-heptulosonate-7-phosphate synthase, tryptophan repressible	0.0454	0.3102
JW1695	ydiE	hemin uptake protein HemP homolog	0.0476	0.0118
JW1696	ydiU	UPF0061 family protein	-0.0232	0.2358
JW1697	ydiV	anti-FlhD4C2 factor, inactive EAL family phosphodiesterase	0.0142	0.7609
JW1698	nlpC	putative C40 clan peptidase lipoprotein	0.1185	0.0018
JW1699	btuD	vitamin B12 ABC transporter ATPase	-0.0459	0.2594
JW1700	btuE	glutathione peroxidase	-0.1241	0.0003
JW1701	btuC	vitamin B12 ABC transporter permease	-0.0407	0.1383
JW1702	ihfA	integration host factor (IHF), DNA-binding protein, alpha subunit	0.1454	0.0103
JW1705	pheM	phenylalanyl-tRNA synthetase operon leader peptide	-0.0361	0.1583
JW1711	ydiY	acid-inducible putative outer membrane protein	-0.0151	0.5889
JW1713	ydiZ	uncharacterized protein	-0.0042	0.7773

JW1714	yniA	fructosamine kinase family protein	-0.0023	0.9038
JW1715	yniB	putative inner membrane protein	-0.1099	0.0002
JW1716	yniC	hexitol phosphatase B; 2-deoxyglucose-6-P phosphatase	0.0182	0.6593
JW1718	ydjN	putative transporter	0.1126	0.0910
JW1719	ydjO	uncharacterized protein	-0.0632	0.0199
JW1720	cedA	cell division modulator	-0.0138	0.5279
JW1721	katE	catalase HPII, heme d-containing	0.0968	0.0618
JW1722	chbG	chito-oligosaccharide deacetylase	-0.0907	0.0327
JW1723	chbF	phospho-chitobiase; general 6-phospho-beta-glucosidase activity	-0.1138	0.0057
JW1724	chbR	repressor of chb operon for N,N'-diacetylchitobiose utilization	-0.0089	0.5960
JW1725	chbA	N,N'-diacetylchitobiose-specific enzyme IIA component of PTS	-0.0657	0.1125
JW1726	chbC	N,N'-diacetylchitobiose-specific enzyme IIC component of PTS	-0.0367	0.2280
JW1727	chbB	N,N'-diacetylchitobiose-specific enzyme IIB component of PTS	-0.2784	0.0013
JW1728	osmE	osmotically-inducible lipoprotein	-0.0739	0.1879
JW1730	ydjQ	endonuclease of nucleotide excision repair	0.0637	0.0152
JW1731	ydjR	cold- and stress-inducible protein	-0.0694	0.0107
JW1732	spy	periplasmic ATP-independent protein refolding chaperone, stress-induced	-0.0660	0.0557
JW1733	astE	succinylglutamate desuccinylase	0.1087	0.0125
JW1734	astB	succinylarginine dihydrolase	-0.1289	0.0004
JW1736	astA	arginine succinyltransferase	0.1502	0.0012
JW1737	astC	succinylornithine transaminase, PLP-dependent	-0.0054	0.7157
JW1738	xthA	exonuclease III	0.0632	0.0840
JW1739	ydjX	TVP38/TMEM64 family inner membrane protein	0.1014	0.0018
JW1741	ydjZ	TVP38/TMEM64 family inner membrane protein	-0.1928	0.0038
JW1742	ynjA	carboxymuconolactone decarboxylase family protein	-0.0155	0.4857
JW1747	ynjF	CDP-alcohol phosphatidyltransferase family inner membrane protein	0.0741	0.1253
JW1748	nudG	CTP pyrophosphohydrolase; also hydrolyzes 2-hydroxy-dATP, 8-hydroxy-dGTP, 5-hydroxy-CTP, dCTP and 5-methyl-dCTP	-0.0169	0.5778
JW1749	ynjH	DUF1496 family protein	-0.0713	0.0063
JW1750	gdhA	glutamate dehydrogenase, NADP-specific	0.0522	0.0326
JW1752	topB	DNA topoisomerase III	0.0229	0.0980
JW1753	selD	selenophosphate synthase	0.0104	0.5210
JW1754	ydjA	putative oxidoreductase	0.0520	0.2330

JW1755	sppA	protease IV (signal peptide peptidase)	-0.0337	0.3327
JW1756	ansA	cytoplasmic L-asparaginase 1	0.0835	0.0447
JW1757	pncA	nicotinamidase/pyrazinamidase	0.0803	0.1087
JW1758	ydjE	putative MFS sugar transporter, membrane protein	-0.1397	0.0372
JW1759	ydjF	putative DNA-binding transcriptional regulator	0.0422	0.1125
JW1760	ydjG	methylglyoxal reductase, NADH-dependent	-0.0480	0.0976
JW1762	ydjI	putative aldolase	-0.0285	0.0277
JW1763	ydjJ	putative Zn-dependent NAD(P)-binding oxidoreductase	0.1029	0.0246
JW1765	ydjL	putative Zn-dependent NAD(P)-binding oxidoreductase	0.0960	0.0096
JW1766	yeaC	DUF1315 family protein	-0.0530	0.3181
JW1767	yeaA	methionine sulfoxide reductase B	-0.0526	0.2178
JW1769	yeaD	D-hexose-6-phosphate epimerase-like protein	0.0008	0.9652
JW1770	yeaE	aldo-keto reductase, methylglyoxal to acetol, NADPH-dependent	-0.0183	0.5087
JW1771	mipA	scaffolding protein for murein synthesizing machinery	0.0176	0.5973
JW1772	yeaG	protein kinase, endogenous substrate unidentified; autokinase	0.0460	0.0328
JW1773	yeaH	UPF0229 family protein	0.0641	0.3200
JW1774	yeaI	Inactive diguanylate cyclase	0.0203	0.2738
JW1776	yeaK	aminoacyl-tRNA editing domain protein	-0.0428	0.4278
JW1778	yeaL	UPF0756 family putative inner membrane protein	-0.0322	0.0538
JW1779	yeaM	putative DNA-binding transcriptional regulator	0.0073	0.7494
JW1780	yeaN	putative MFS transporter, inner membrane protein	-0.0148	0.5386
JW1781	yeaO	DUF488 family protein	-0.0393	0.1313
JW1782	yoaF	DUF333 family outer membrane lipoprotein	0.0493	0.0951
JW1784	yeaQ	UPF0410 family protein	0.0151	0.5890
JW1785	yoaG	uncharacterized protein	0.0067	0.9126
JW1786	yeaR	DUF1971 family protein, nitrate-inducible	0.0089	0.5897
JW1787	yeaS	leucine efflux protein	-0.0759	0.1605
JW1788	yeaT	transcriptional activator of dmlA	0.0679	0.0842
JW1789	yeaU	D-malate oxidase, NAD-dependent; putative tartrate dehydrogenase	-0.1027	0.0876
JW1792	yeaX	putative YeaWX dioxygenase beta subunit, reductase component	0.0317	0.2811
JW1793	rnd	ribonuclease D	0.0776	0.0070
JW1794	fadD	acyl-CoA synthetase (long-chain-fatty-acid--CoA ligase)	0.0315	0.1625
JW1795	yeaY	Slp family lipoprotein, RpoE-regulated	0.0203	0.3564
JW1797	yoaA	putative ATP-dependent helicase, DinG family	0.0043	0.8362
JW1801	pabB	aminodeoxychorismate synthase, subunit I	0.0248	0.6040

JW1802	yeaB	putative CoA pyrophosphohydrolase, weak 3-phosphohydroxypyruvate phosphatase	-0.0042	0.8830
JW1803	sdaA	L-serine dehydratase 1	0.1051	0.2596
JW1804	yoaD	putative membrane-anchored cyclic-di-GMP phosphodiesterase, regulator of cellulose production	0.0016	0.9229
JW1805	yoaE	putative membrane protein/conserved protein	-0.0434	0.4789
JW1806	manX	fused mannose-specific PTS enzymes: IIA component/IIB component	0.0606	0.2943
JW1807	manY	mannose-specific enzyme IIC component of PTS	0.1448	0.2011
JW1808	manZ	mannose-specific enzyme IID component of PTS	0.0223	0.0705
JW1809	yobD	UPF0266 family inner membrane protein	-0.1019	0.0465
JW1811	rrmA	23S rRNA m(1)G745 methyltransferase, SAM-dependent	-0.0590	0.0130
JW1812	cspC	stress protein, member of the CspA-family	0.0021	0.0005
JW1813	yobF	DUF2527 family heat-induced protein	-0.1873	0.0067
JW1814	yebO	putative inner membrane protein	-0.0553	0.2124
JW1815	yobG	regulatory peptide for PhoPQ, feedback inhibition	-0.0366	0.2129
JW1816	kdgR	KDG regulon transcriptional repressor	-0.0169	0.4238
JW1818	htpX	putative endopeptidase	0.0540	0.6452
JW1819	prc	carboxy-terminal protease for penicillin-binding protein 3	0.2088	0.0000
JW1821	yebR	free methionine-(R)-sulfoxide reductase	-0.1318	0.0208
JW1822	yebS	inner membrane subunit of the putative YebST transporter; PqiA domain protein	-0.0740	0.0529
JW1823	yebT	periplasmic MCE subunit of the putative YebST transporter, IM-anchored	-0.0879	0.0802
JW1827	pphA	serine/threonine-specific protein phosphatase 1	-0.0262	0.4262
JW1828	yebY	DUF2511 family protein	-0.0713	0.0916
JW1829	yebZ	inner membrane protein	0.0033	0.9177
JW1830	yobA	CopC family protein	0.0784	0.0632
JW1831	holE	DNA polymerase III, theta subunit	-0.1076	0.0040
JW1832	yobB	C-N hydrolase family protein	-0.1899	0.0000
JW1833	exoX	exodeoxyribonuclease 10; DNA exonuclease X	0.0512	0.0837
JW1834	ptrB	protease II	0.0699	0.3100
JW1835	yebE	DUF533 family inner membrane protein	-0.0058	0.8846
JW1836	yebF	extracellular Colicin M immunity family protein	-0.0049	0.8973

JW1837	yebG	DNA damage-inducible protein regulated by LexA	0.0934	0.0015
JW1838	purT	phosphoribosylglycinamide formyltransferase 2	0.1154	0.0699
JW1839	eda	KHG/KDPG aldolase; 2-dehydro-3-deoxy-phosphogluconate/4-hydroxy-2-oxoglutarate aldolase	0.0090	0.5972
JW1840	edd	6-phosphogluconate dehydratase	0.0158	0.5403
JW1841	zwf	glucose-6-phosphate 1-dehydrogenase	-0.0263	0.3605
JW1842	yebK	putative DNA-binding transcriptional regulator	0.0545	0.1308
JW1843	pykA	pyruvate kinase II	-0.0369	0.5901
JW1844	lpxM	myristoyl-acyl carrier protein (ACP)-dependent acyltransferase	0.1008	0.0353
JW1847	znuC	zinc ABC transporter ATPase	0.1179	0.0057
JW1848	znuB	zinc ABC transporter permease	0.0297	0.3130
JW1849	ruvB	ATP-dependent DNA helicase, component of RuvABC resolvasome	-0.0369	0.3975
JW1850	ruvA	component of RuvABC resolvasome, regulatory subunit	-0.4221	0.0033
JW1852	ruvC	component of RuvABC resolvasome, endonuclease	0.1639	0.4090
JW1853	yebC	UPF0082 family protein	0.0101	0.7913
JW1854	nudB	dihydroneopterin triphosphate pyrophosphatase	0.2439	0.0003
JW1857	yecE	UPF0759 family protein	-0.1413	0.0432
JW1859	yecO	carboxy-SAM synthase	-0.0461	0.0003
JW1861	torZ	trimethylamine N-oxide reductase system III, catalytic subunit	-0.0390	0.3081
JW1862	torY	TMAO reductase III (TorYZ), cytochrome c-type subunit	0.0175	0.1808
JW1863	cutC	putative copper homeostasis protein	-0.0159	0.7243
JW1867	flhE	proton seal during flagellar secretion	-0.1316	0.1554
JW1868	flhA	putative flagellar export pore protein	-0.0010	0.9760
JW1869	flhB	flagellin export apparatus, substrate specificity protein	-0.1043	0.0005
JW1870	cheZ	chemotaxis regulator, protein phosphatase for CheY	0.0388	0.4204
JW1871	cheY	chemotaxis regulator transmitting signal to flagellar motor component	0.0077	0.8022
JW1872	cheB	fused chemotaxis regulator: protein-glutamate methylesterase in two-component regulatory system with CheA	0.0319	0.6198
JW1873	cheR	chemotaxis regulator, protein-glutamate methyltransferase	-0.0538	0.1744
JW1874	tap	methyl-accepting protein IV	0.0706	0.0035
JW1875	tar	methyl-accepting chemotaxis protein II	0.0487	0.0885
JW1876	cheW	purine-binding chemotaxis protein	-0.0091	0.7803

JW1877	cheA	fused chemotactic sensory histidine kinase in two-component regulatory system with CheB and CheY: sensory histidine kinase/signal sensing protein	-0.0298	0.3450
JW1878	motB	protein that enables flagellar motor rotation	0.1043	0.1034
JW1879	motA	proton conductor component of flagella motor	0.0131	0.7670
JW1880	flhC	flagellar class II regulon transcriptional activator, with FlhD	0.0198	0.2197
JW1881	flhD	flagellar class II regulon transcriptional activator, with FlhC	-0.0215	0.6406
JW1884	yecG	universal stress protein	-0.2251	0.1293
JW1886	otsB	trehalose-6-phosphate phosphatase, biosynthetic	0.1320	0.0015
JW1887	araH	L-arabinose ABC transporter permease	0.0448	0.0348
JW1888	araG	L-arabinose ABC transporter ATPase	0.0604	0.1717
JW1889	araF	L-arabinose ABC transporter periplasmic binding protein	0.0956	0.0767
JW1890	yecI	ferritin B, putative ferrous iron reservoir	0.0122	0.6589
JW1891	yecJ	DUF2766 family protein	0.0161	0.6214
JW1892	yecR	lipoprotein, function unknown	-0.0106	0.7756
JW1893	ftn	ferritin iron storage protein (cytoplasmic)	-0.2436	0.0369
JW1894	yecH	DUF2492 family protein	-0.0566	0.0608
JW1895	tyrP	tyrosine transporter	-0.3166	0.0143
JW1896	yecA	UPF0149 family protein	-0.1662	0.0000
JW1898	uvrC	excinuclease UvrABC, endonuclease subunit	-0.0138	0.5854
JW1899	uvrY	response regulator in two-component regulatory system with BarA	0.0286	0.4486
JW1900	yecF	DUF2594 family protein	-0.0133	0.4960
JW1901	sdiA	quorum-sensing transcriptional activator	0.0563	0.0019
JW1902	yecC	putative ABC transporter ATPase	-0.0052	0.8815
JW1903	yecS	ABC family putative inner membrane permease	0.0183	0.4430
JW1905	fliY	cystine transporter subunit	0.0546	0.5977
JW1906	fliZ	RpoS antagonist; putative regulator of FliA activity	0.0849	0.3257
JW1907	fliA	RNA polymerase, sigma 28 (sigma F) factor	-0.0937	0.0002
JW1908	fliC	flagellar filament structural protein (flagellin)	0.1141	0.0003
JW1909	fliD	flagellar filament capping protein	0.0041	0.8987
JW1910	fliS	flagellar protein potentiates polymerization	0.0238	0.6531
JW1911	fliT	putative flagellar synthesis and assembly chaperone	0.0720	0.1578
JW1913	yedD	lipoprotein	-0.0013	0.9469

JW1914	yedE	UPF0394 family sulphur transport domain-containing inner membrane protein	0.0997	0.0132
JW1915	yedF	putative TusA family sulfurtransferase	0.0661	0.0082
JW1916	yedK	DUF159 family protein	0.0009	0.9677
JW1917	yedL	GNAT family putative N-acetyltransferase	-0.0094	0.6087
JW1918	yedN	Uncharacterized protein	0.0231	0.1591
JW1920	yedM	Uncharacterized protein	0.0985	0.0192
JW1921	fliE	flagellar basal-body component	-0.1111	0.0337
JW1922	fliF	flagellar basal-body MS-ring and collar protein	0.0310	0.2654
JW1923	fliG	flagellar motor switching and energizing component	-0.0345	0.0551
JW1924	fliH	negative regulator of FliI ATPase activity	-0.0691	0.1148
JW1925	fliI	flagellum-specific ATP synthase	0.0464	0.0487
JW1926	fliJ	flagellar protein	0.0337	0.2982
JW1927	fliK	flagellar hook-length control protein	0.0140	0.6588
JW1928	fliL	flagellar biosynthesis protein	0.2246	0.0128
JW1929	fliM	flagellar motor switching and energizing component	0.0007	0.9724
JW1930	fliN	flagellar motor switching and energizing component	0.0425	0.2312
JW1932	fliP	flagellar biosynthesis protein	0.1292	0.0706
JW1933	fliQ	flagellar biosynthesis protein	-0.0359	0.4625
JW1934	fliR	flagellar export pore protein	0.2425	0.0010
JW1935	rcsA	transcriptional regulator of colanic acid capsular biosynthesis	0.0339	0.2451
JW1936	dsrB	uncharacterized protein	0.0256	0.2260
JW1938	yedP	putative mannosyl-3-phosphoglycerate phosphatase	0.0642	0.0374
JW1940	yodC	uncharacterized protein	-0.0235	0.1428
JW1941	yedI	DUF808 family inner membrane protein	0.0524	0.1647
JW1942	yedA	amino acid exporter for phenylalanine, threonine	0.0514	0.0744
JW1943	vsr	DNA mismatch endonuclease of very short patch repair	0.1470	0.0222
JW1944	dcm	DNA cytosine methyltransferase	0.0958	0.0066
JW1945	yedJ	putative HD superfamily phosphohydrolase	0.0237	0.5361
JW1946	yedR	inner membrane protein	0.0642	0.0869
JW1948	yedS	Putative outer membrane protein	-0.0827	0.0610
JW1950	hchA	glyoxalase III and Hsp31 molecular chaperone	0.0462	0.0815
JW1951	yedV	putative sensory kinase in two-component regulatory system with YedW	0.0340	0.1490
JW1953	yedX	hydroxyisourate hydrolase	-0.1246	0.0140
JW1954	yedY	membrane-anchored, periplasmic TMAO, DMSO reductase	0.0102	0.7891
JW1955	yedZ	inner membrane heme subunit for periplasmic YedYZ reductase	0.0223	0.4021

JW1956	yodA	zinc and cadmium binding protein, periplasmic	-0.1038	0.0399
JW1958	yeeI	anti-repressor for DgsA(Mlc)	0.0557	0.2234
JW1961	yeeL	Uncharacterized protein	0.0939	0.0033
JW1962	shiA	shikimate transporter	-0.0197	0.6715
JW1963	amn	AMP nucleosidase	-0.0945	0.0892
JW1964	yeeN	UPF0082 family protein	0.0110	0.5675
JW1965	yeeO	putative multdrug exporter, MATE family	0.0951	0.0028
JW1966	cbl	ssuEADCB/tauABCD operon transcriptional activator	0.0390	0.2634
JW1967	nac	nitrogen assimilation regulon transcriptional regulator; autorepressor	0.0519	0.0471
JW1968	erfK	L,D-transpeptidase linking Lpp to murein	0.1160	0.0217
JW1969	cobT	nicotinate-nucleotide--dimethylbenzimidazole phosphoribosyltransferase	0.0103	0.8018
JW1970	cobS	cobalamin synthase	-0.0868	0.2186
JW1971	cobU	cobinamide kinase and cobinamide phosphate guanylyltransferase	0.0181	0.5451
JW1982	flu	CP4-44 prophage; antigen 43 (Ag43) phase-variable biofilm formation autotransporter	0.0393	0.1321
JW1983	yeeR	CP4-44 prophage; putative membrane protein	-0.0131	0.5730
JW1984	yeeS	CP4-44 prophage; putative DNA repair protein	0.0415	0.0552
JW1985	yeeT	CP4-44 prophage; uncharacterized protein	0.0120	0.5373
JW1986	yeeU	CP4-44 prophage; cytoskeleton bundling-enhancing factor A; CbtA antitoxin	-0.0788	0.1142
JW1987	yeeV	CP4-44 prophage; toxin of the YeeV-YeeU toxin-antitoxin system	0.0215	0.4393
JW1988	yeeW	CP4-44 prophage; uncharacterized protein	0.0313	0.0788
JW1989	yeeX	UPF0265 family protein	0.0934	0.0002
JW1990	yeeA	putative transporter, FUSC family inner membrane protein	0.0240	0.1963
JW1991	sbmC	DNA gyrase inhibitor	0.0976	0.0290
JW1993	sbcB	exodeoxyribonuclease I; exonuclease I	0.0417	0.2372
JW1994	yeeD	putative TusA family sulfurtransferase	0.0471	0.2170
JW1995	yeeE	UPF0394 family inner membrane protein	0.0038	0.8903
JW1998	yeeZ	putative epimerase	0.3711	0.0086
JW2000	hisL	his operon leader peptide	-0.0058	0.7897
JW2001	hisG	ATP phosphoribosyltransferase	0.0949	0.1704
JW2002	hisD	bifunctional histidinal dehydrogenase/ histidinol dehydrogenase	0.0041	0.9690
JW2003	hisC	histidinol-phosphate aminotransferase	0.0006	0.9949

JW2004	hisB	histidinol-phosphatase and imidazoleglycerol-phosphate dehydratase	0.1094	0.0001
JW2005	hisH	imidazole glycerol phosphate synthase, glutamine amidotransferase subunit	-0.0285	0.6782
JW2006	hisA	N-(5'-phospho-L-ribosyl-formimino)-5-amino-1-(5'-phosphoribosyl)-4-imidazolecarboxamide isomerase	-0.1623	0.0051
JW2007	hisF	imidazole glycerol phosphate synthase, catalytic subunit with HisH	0.0068	0.9702
JW2008	hisI	phosphoribosyl-AMP cyclohydrolase and phosphoribosyl-ATP pyrophosphatase	0.0287	0.6003
JW2010	ugd	UDP-glucose 6-dehydrogenase	-0.0941	0.3858
JW2011	gnd	6-phosphogluconate dehydrogenase, decarboxylating	-0.0161	0.6454
JW2012	wbbL	Glucose-1-phosphate thymidylyltransferase 1	-0.0762	0.0000
JW2017	wbbK	lipopolysaccharide biosynthesis protein	-0.0494	0.1440
JW2018	wbbJ	putative lipopolysaccharide biosynthesis O-acetyl transferase	-0.0328	0.2755
JW2019	wbbI	d-Galf:alpha-d-Glc beta-1,6-galactofuranosyltransferase	-0.1341	0.0008
JW2020	wbbH	O-antigen polymerase	0.0331	0.2894
JW2021	glf	UDP-galactopyranose mutase, FAD/NAD(P)-binding	-0.0934	0.0219
JW2022	rfbX	putative polisoprenol-linked O-antigen transporter	0.0187	0.7470
JW2023	rfbC	dTDP-4-deoxyrhamnose-3,5-epimerase	0.0728	0.0019
JW2024	rfbA	glucose-1-phosphate thymidylyltransferase	0.1324	0.0320
JW2025	rfbD	dTDP-L-rhamnose synthase, NAD(P)-dependent dTDP-4-dehydrorhamnose reductase subunit	0.0862	0.1575
JW2026	rfbB	dTDP-glucose 4,6 dehydratase, NAD(P)-binding	-0.0116	0.5243
JW2027	galF	putative regulatory subunit for GalU	-0.0053	0.8200
JW2028	wcaM	colanic acid biosynthesis protein	0.0493	0.0946
JW2029	wcaL	putative glycosyl transferase	0.1035	0.0303
JW2030	wcaK	colanic acid biosynthesis protein	-0.1010	0.0405
JW2031	wzxC	putative colanic acid exporter	-0.0223	0.7493
JW2032	wcaJ	colanic biosynthesis UDP-glucose lipid carrier transferase	0.0414	0.0992
JW2033	cpsG	phosphomannomutase	0.0949	0.2545
JW2034	cpsB	mannose-1-phosphate guanyltransferase	-0.0170	0.0708
JW2035	wcaI	putative glycosyl transferase	0.2625	0.0051
JW2037	fcl	bifunctional GDP-fucose synthetase: GDP-4-dehydro-6-deoxy-D-mannose epimerase/ GDP-4-dehydro-6-L-deoxygalactose reductase	0.0200	0.7836

JW2038	gmd	GDP-D-mannose dehydratase, NAD(P)-binding	0.0708	0.1010
JW2039	wcaF	putative acyl transferase	0.1500	0.1180
JW2040	wcaE	putative glycosyl transferase	0.0166	0.2044
JW2041	wcaD	putative colanic acid polymerase	-0.0079	0.6024
JW2042	wcaC	putative glycosyl transferase	0.1345	0.0397
JW2043	wcaB	putative acyl transferase	-0.0353	0.6164
JW2044	wcaA	putative glycosyl transferase	-0.0196	0.3812
JW2045	wzc	colanic acid production tyrosine-protein kinase; autokinase; Ugd phosphorylase	-0.0009	0.9255
JW2046	wzb	colanic acid production protein-tyrosine-phosphatase; Wzc-P dephosphorylase	-0.0094	0.7994
JW2047	wza	colanic acid export protein; outer membrane auxillary lipoprotein	-0.0093	0.7078
JW2049	asmA	suppressor of OmpF assembly mutants; putative outer membrane protein assembly factor; inner membrane-anchored periplasmic protein	0.1249	0.0001
JW2050	dcd	deoxycytidine triphosphate deaminase; dCTP deaminase	0.2240	0.0078
JW2051	udk	uridine-cytidine kinase	-0.1351	0.0175
JW2052	yegE	putative diguanylate cyclase	0.0209	0.4004
JW2053	alkA	3-methyl-adenine DNA glycosylase II	-0.0433	0.1131
JW2054	yegD	Hsp70 chaperone family protein	-0.0569	0.0117
JW2055	yegI	protein kinase-related putative non-specific DNA-binding protein	-0.0302	0.4945
JW2056	yegJ	DUF2314 family protein	0.0407	0.1377
JW2057	yegK	ser/thr phosphatase-related protein	-0.0941	0.0011
JW2058	yegL	VMA domain protein	-0.1320	0.1403
JW2060	mdtB	multidrug efflux system, subunit B	-0.0248	0.4109
JW2061	mdtC	multidrug efflux system, subunit C	-0.0020	0.9053
JW2062	mdtD	putative citrate/iron-citrate/zinc-citrate efflux transporter	-0.0087	0.7900
JW2063	baeS	sensory histidine kinase in two-component regulatory system with BaeR	-0.0378	0.1347
JW2064	baeR	response regulator in two-component regulatory system with BaeS	0.0710	0.0304
JW2066	yegQ	putative peptidase	0.0822	0.0797
JW2067	ogrK	orphan Ogr protein, positive regulator of P2 growth	0.1923	0.0000
JW2070	yegS	phosphatidylglycerol kinase, metal-dependent	0.0128	0.4352
JW2074	gatR	Putative galactitol utilization operon repressor	-0.0111	0.6993
JW2075	gatD	galactitol-1-phosphate dehydrogenase, Zn-dependent and NAD(P)-binding	0.0538	0.0065

JW2076	gatC	pseudogene, galactitol-specific enzyme IIC component of PTS	-0.0013	0.9673
JW2077	gatB	galactitol-specific enzyme IIB component of PTS	0.0422	0.3326
JW2082	gatZ	D-tagatose 1,6-bisphosphate aldolase 2, subunit	-0.1107	0.0002
JW2085	yegT	nucleoside transporter, low affinity	-0.0529	0.1214
JW2086	yegU	ADP-ribosylglycohydrolase family protein	0.0848	0.0598
JW2087	yegV	putative kinase	0.0139	0.6674
JW2088	yegW	putative DNA-binding transcriptional regulator	-0.0080	0.7082
JW2090	thiD	hydroxy-methylpyrimidine kinase and hydroxy-phosphomethylpyrimidine kinase	0.0312	0.2577
JW2092	yohL	transcriptional repressor of rcnA	-0.0073	0.8354
JW2093	yohM	membrane protein conferring nickel and cobalt resistance	0.0054	0.7462
JW2095	yehA	putative fimbrial-like adhesin protein	-0.0144	0.3939
JW2096	yehB	putative outer membrane protein	0.0594	0.1257
JW2097	yehC	putative periplasmic pilin chaperone	-0.0109	0.6143
JW2098	yehD	putative fimbrial-like adhesin protein	-0.0261	0.3097
JW2099	yehE	DUF2574 family protein	-0.1350	0.1220
JW2100	mrp	antiporter inner membrane protein	0.1867	0.0345
JW2102	molR	Putative molybdate metabolism regulator	0.0248	0.4520
JW2106	yehK	uncharacterized protein	0.0781	0.0046
JW2108	yehM	uncharacterized protein	0.0187	0.1146
JW2110	yehQ	pseudogene	-0.0537	0.0011
JW2112	yehS	DUF1456 family protein	0.0158	0.5156
JW2115	mlrA	transcriptional activator of csgD and csgBA	-0.0270	0.5131
JW2116	yehW	putative ABC transporter permease	0.0199	0.5796
JW2117	yehX	putative ABC transporter ATPase	0.0236	0.5198
JW2118	yehY	putative ABC transporter permease	0.0762	0.0405
JW2119	yehZ	putative ABC transporter periplasmic binding protein	-0.0121	0.3581
JW2120	bglX	beta-D-glucoside glucohydrolase, periplasmic	-0.0991	0.0044
JW2121	dld	D-lactate dehydrogenase, FAD-binding, NADH independent	0.0157	0.5490
JW2124	yohD	DedA family inner membrane protein	-0.0610	0.0317
JW2125	yohF	putative oxidoreductase	-0.0060	0.7620
JW2128	dusC	tRNA-dihydrouridine synthase C	-0.0464	0.0675
JW2129	yohJ	UPF0299 family inner membrane protein	0.0454	0.0021
JW2130	yohK	LrgB family inner membrane protein	0.0226	0.1986
JW2131	cdd	cytidine/deoxycytidine deaminase	0.0469	0.3173
JW2132	sanA	DUF218 superfamily vancomycin high temperature exclusion protein	-0.3201	0.0043
JW2133	yeiT	dihydropyrimidine dehydrogenase, NADH-dependent, subunit N	-0.0713	0.0491
JW2134	yeiA	dihydropyrimidine dehydrogenase, NADH-dependent, subunit C	0.0918	0.0112
JW2135	mglC	methyl-galactoside transporter subunit	0.0521	0.2836

JW2136	mglA	methyl-galactoside ABC transporter ATPase	-0.1458	0.2555
JW2137	mglB	methyl-galactoside transporter subunit	-0.0443	0.3257
JW2138	galS	galactose- and fucose-inducible galactose regulon transcriptional isorepressor; mgl operon transcriptional repressor; autorepressor	-0.0092	0.7909
JW2139	yeiB	DUF418 family putative inner membrane protein	-0.0327	0.1471
JW2141	yeiG	S-formylglutathione hydrolase	-0.0680	0.1510
JW2142	cirA	colicin IA outer membrane receptor and translocator; ferric iron-catecholate transporter	-0.0309	0.3062
JW2143	lysP	lysine transporter	0.0397	0.1467
JW2144	yeiE	putative DNA-binding transcriptional regulator	-0.0128	0.6039
JW2145	yeiH	UPF0324 family inner membrane protein	0.0635	0.2533
JW2146	nfo	endonuclease IV with intrinsic 3'-5' exonuclease activity	0.0575	0.0435
JW2147	yeiI	putative kinase	0.0483	0.1212
JW2148	yeiJ	nucleoside permease	0.0083	0.7322
JW2149	rihB	ribonucleoside hydrolase 2	0.0298	0.1568
JW2150	yeiL	nitrogen starvation viability factor; putative ColA sensitivity effector; putative Crp-Fnr family transcriptional regulator	-0.0805	0.0139
JW2151	yeiM	putative nucleoside transporter	-0.0077	0.6628
JW2152	yeiN	pseudouridine 5'-phosphate glycosidase	0.0217	0.6015
JW2153	yeiC	pseudouridine kinase	-0.0516	0.0585
JW2154	fruA	fused fructose-specific PTS enzymes: IIBcomponent/IIC components	-0.0880	0.0236
JW2155	fruK	fructose-1-phosphate kinase	0.0778	0.0000
JW2156	fruB	fused fructose-specific PTS enzymes: IIA component/HPr component	0.0990	0.1261
JW2157	setB	lactose/glucose efflux system	0.0363	0.2333
JW2160	yeiQ	putative NAD-dependent D-mannonate oxidoreductase	0.0887	0.0215
JW2161	yeiR	Zn-stimulated GTPase involved in zinc homeostasis; mutants are cadmium and EDTA sensitive; Zn(2+) binding protein	-0.0110	0.6861
JW2162	yeiU	lipid A 1-diphosphate synthase; undecaprenyl pyrophosphate:lipid A 1-phosphate phosphotransferase	0.0252	0.3074
JW2163	spr	murein DD-endopeptidase, space-maker hydrolase, mutational suppressor of prc thermosensitivity, outer membrane lipoprotein, weak murein LD-carboxypeptidase	0.0057	0.8830

JW2164	rtn	resistance protein for phages lambda and N4, putative membrane-anchored cyclic-di-GMP phosphodiesterase	0.0908	0.0571
JW2165	yejA	microcin C ABC transporter periplasmic binding protein	0.0647	0.0006
JW2166	yejB	microcin C ABC transporter permease	-0.0162	0.3893
JW2167	yejE	microcin C ABC transporter permease	-0.0043	0.8520
JW2168	yejF	microcin C ABC transporter ATPase	0.2254	0.0176
JW2169	yejG	uncharacterized protein	-0.1795	0.1114
JW2171	rsuA	16S rRNA pseudouridine(516) synthase	-0.0010	0.9721
JW2172	yejH	putative ATP-dependent DNA or RNA helicase	0.0257	0.4763
JW2173	rplY	50S ribosomal subunit protein L25	0.0178	0.7212
JW2175	yejL	UPF0352 family protein	-0.0170	0.2502
JW2181	narP	response regulator in two-component regulatory system with NarQ	0.0062	0.7828
JW2182	ccmH	heme lyase, CcmH subunit	0.0220	0.5112
JW2183	ccmG	periplasmic thioredoxin of cytochrome c-type biogenesis	0.1365	0.0125
JW2184	ccmF	heme lyase, CcmF subunit	-0.0115	0.8212
JW2185	ccmE	periplasmic heme chaperone	0.0510	0.0304
JW2188	ccmB	heme export ABC transporter permease	-0.0274	0.2703
JW2190	napC	quinol dehydrogenase, electron source for NapAB	0.1287	0.1728
JW2192	napH	ferredoxin-type protein	0.0623	0.1763
JW2193	napG	ferredoxin-type protein	-0.0554	0.2671
JW2194	napA	nitrate reductase, periplasmic, large subunit	0.0113	0.6679
JW2195	napD	assembly protein for periplasmic nitrate reductase	0.0473	0.0371
JW2196	napF	ferredoxin-type protein, role in electron transfer to periplasmic nitrate reductase NapA	0.0365	0.0076
JW2197	eco	ecotin, a serine protease inhibitor	-0.0146	0.4233
JW2198	mqo	malate dehydrogenase, FAD/NAD(P)-binding domain	0.0561	0.2982
JW2199	yojI	microcin J25 efflux ABC transporter permease/ATPase	0.1773	0.0134
JW2200	alkB	oxidative demethylase of N1-methyladenine or N3-methylcytosine DNA lesions	-0.0665	0.0083
JW2201	ada	fused DNA-binding transcriptional dual regulator/O6-methylguanine-DNA methyltransferase	0.0272	0.3023
JW2203	ompC	outer membrane porin protein C	0.1032	0.1080

JW2204	rcsD	phosphotransfer intermediate protein in two-component regulatory system with RcsBC	-0.0767	0.0211
JW2205	rcsB	response regulator in two-component regulatory system with RcsC and YojN	0.0575	0.0227
JW2213	atoS	sensory histidine kinase in two-component regulatory system with AtoC	0.0371	0.2655
JW2214	atoC	fused response regulator of ato operon, in two-component system with AtoS: response regulator/sigma54 interaction protein	-0.1070	0.0623
JW2215	atoD	acetyl-CoA:acetoacetyl-CoA transferase, alpha subunit	-0.1170	0.0163
JW2216	atoA	acetyl-CoA:acetoacetyl-CoA transferase, beta subunit	-0.0445	0.0636
JW2217	atoE	short chain fatty acid transporter	0.0116	0.4578
JW2218	atoB	acetyl-CoA acetyltransferase	-0.0541	0.0947
JW2219	yfaP	DUF2135 family protein, putative host defense protein	-0.0077	0.0831
JW2220	yfaQ	tandem DUF2300 domain protein, putative host defense protein	-0.0093	0.7521
JW2221	yfaS	Putative UPF0192 protein	-0.2235	0.0404
JW2223	yfaT	DUF1175 family protein, putative host defense protein	0.0401	0.3723
JW2224	yfaA	DUF2138 family protein, putative host defense protein	0.1604	0.0001
JW2226	ubiG	bifunctional 3-demethylubiquinone-9 3-methyltransferase/ 2-octaprenyl-6-hydroxy phenol methylase	0.0919	0.1566
JW2227	yfaL	adhesin	0.0573	0.0159
JW2230	yfaE	ferredoxin involved with ribonucleotide reductase diferric-tyrosyl radical (Y*) cofactor maintenance	0.1047	0.0000
JW2231	inaA	acid-inducible Kdo/WaaP family putative kinase	-0.1541	0.3019
JW2232	yfaH	pseudogene	-0.2269	0.0001
JW2233	glpQ	periplasmic glycerophosphodiester phosphodiesterase	0.0388	0.5144
JW2234	glpT	sn-glycerol-3-phosphate transporter	0.0805	0.3588
JW2235	glpA	anaerobic sn-glycerol-3-phosphate dehydrogenase, large FAD/NAD(P)-binding subunit	-0.0398	0.1259
JW2236	glpB	anaerobic sn-glycerol-3-phosphate dehydrogenase membrane anchor subunit	0.1511	0.0452
JW2237	glpC	anaerobic sn-glycerol-3-phosphate dehydrogenase, C subunit, 4Fe-4S iron-sulfur cluster	-0.0069	0.6671
JW2238	yfaD	transposase_31 family protein	-0.3210	0.0423

JW2239	yfaU	2-keto-3-deoxy-L-rhamnonate aldolase	0.1692	0.0083
JW2240	yfaV	putative L-rhamnonate transporter	-0.1334	0.4586
JW2241	yfaW	L-rhamnonate dehydratase	-0.0496	0.0038
JW2242	yfaX	putative DNA-binding transcriptional regulator for the rhm operon	-0.0470	0.0515
JW2243	yfaY	inactive PncC family protein	-0.0301	0.1406
JW2245	yfaO	nucleoside triphosphatase	-0.0410	0.0286
JW2246	ais	putative LPS core heptose(II)-phosphate phosphatase	0.0356	0.1149
JW2248	yfbF	undecaprenyl phosphate-L-Ara4FN transferase	0.0701	0.1546
JW2249	yfbG	fused UDP-L-Ara4N formyltransferase/UDP-GlcA C-4'-decarboxylase	-0.0413	0.1033
JW2250	yfbH	undecaprenyl phosphate-alpha-L-ara4FN deformylase	0.0186	0.6638
JW2251	arnT	4-amino-4-deoxy-L-arabinose transferase	-0.0475	0.0588
JW2252	yfbW	undecaprenyl phosphate-alpha-L-ara4N exporter; flippase ArnEF subunit	0.0471	0.2988
JW2254	pmrD	inactive two-component system connector protein	-0.0784	0.2299
JW2255	menE	O-succinylbenzoate-CoA ligase	0.0403	0.2362
JW2256	menC	O-succinylbenzoyl-CoA synthase	0.1062	0.0117
JW2257	menB	dihydroxynaphthoic acid synthetase	0.0092	0.8365
JW2258	yfbB	2-succinyl-6-hydroxy-2,4-cyclohexadiene-1-carboxylate synthase	0.0165	0.3781
JW2260	menF	isochorismate synthase 2	0.0538	0.3448
JW2261	elaB	putative membrane-anchored DUF883 family ribosome-binding protein	-0.0788	0.0435
JW2262	elaA	GNAT family putative N-acetyltransferase	0.0472	0.2291
JW2263	elaC	RNase BN, tRNA processing enzyme	-0.0197	0.3380
JW2265	yfbK	Von Willebrand factor domain putative lipoprotein	0.0036	0.9031
JW2266	yfbL	putative M28A family peptidase	-0.0495	0.1049
JW2267	yfbM	DUF1877 family protein	0.0530	0.0153
JW2268	yfbN	uncharacterized protein	0.0709	0.4476
JW2269	yfbO	uncharacterized protein	-0.0406	0.4643
JW2270	yfbP	TPR-like repeats-containing protein	-0.0489	0.1756
JW2271	nuoN	NADH:ubiquinone oxidoreductase, membrane subunit N	-0.0269	0.2759
JW2272	nuoM	NADH:ubiquinone oxidoreductase, membrane subunit M	-0.0227	0.3711

JW2273	nuoL	NADH:ubiquinone oxidoreductase, membrane subunit L	0.0356	0.2889
JW2274	nuoK	NADH:ubiquinone oxidoreductase, membrane subunit K	-0.0101	0.8770
JW2275	nuoJ	NADH:ubiquinone oxidoreductase, membrane subunit J	0.0069	0.6830
JW2276	nuoI	NADH:ubiquinone oxidoreductase, chain I	-0.0716	0.3532
JW2277	nuoH	NADH:ubiquinone oxidoreductase, membrane subunit H	-0.0298	0.2455
JW2278	nuoG	NADH:ubiquinone oxidoreductase, chain G	-0.0202	0.3722
JW2279	nuoF	NADH:ubiquinone oxidoreductase, chain F	0.0310	0.1148
JW2280	nuoE	NADH:ubiquinone oxidoreductase, chain E	0.0388	0.1586
JW2283	nuoA	NADH:ubiquinone oxidoreductase, membrane subunit A	0.1121	0.0002
JW2284	lrhA	transcriptional repressor of flagellar, motility and chemotaxis genes	0.0466	0.2393
JW2287	yfbQ	glutamate-pyruvate aminotransferase; glutamic-pyruvic transaminase (GPT); alanine transaminase	0.1208	0.0008
JW2288	yfbR	5'-nucleotidase	-0.0770	0.0361
JW2289	yfbS	putative transporter	-0.0589	0.0570
JW2291	yfbU	UPF0304 family protein	0.1109	0.0068
JW2292	yfbV	UPF0208 family inner membrane protein	-0.0079	0.7904
JW2293	ackA	acetate kinase A and propionate kinase 2	-0.0357	0.2169
JW2294	pta	phosphate acetyltransferase	-0.0026	0.9467
JW2295	yfcC	putative inner membrane transporter; C4-dicarboxylate anaerobic carrier family protein	-0.1218	0.0002
JW2296	yfcD	putative NUDIX hydrolase	-0.1433	0.0092
JW2298	yfcF	glutathione S-transferase	0.0036	0.4696
JW2299	yfcG	GSH-dependent disulfide bond oxidoreductase	0.0847	0.0076
JW2300	folX	D-erythro-7,8-dihydroneopterin triphosphate 2'-epimerase and dihydroneopterin aldolase	0.0597	0.0783
JW2301	yfcH	putative NAD-dependent nucleotide-sugar epimerase	-0.0944	0.0731
JW2302	yfcI	transposase_31 family protein	-0.0194	0.5976
JW2303	hisP	histidine ABC transporter ATPase	-0.0664	0.0102
JW2304	hisM	histidine ABC transporter permease	-0.0262	0.1500
JW2305	hisQ	histidine ABC transporter permease	0.0298	0.1478
JW2306	hisJ	histidine ABC transporter periplasmic binding protein	0.0273	0.3429
JW2307	argT	lysine/arginine/ornithine transporter subunit	-0.0783	0.4077
JW2308	ubiX	3-octaprenyl-4-hydroxybenzoate carboxy-lyase	0.0075	0.9729

JW2309	purF	amidophosphoribosyltransferase	0.1567	0.0401
JW2310	cvpA	colicin V production protein	-0.0913	0.1617
JW2314	dedA	DedA family inner membrane protein	0.0535	0.0124
JW2315	truA	tRNA pseudouridine(38-40) synthase	0.0545	0.2214
JW2316	usg	putative semialdehyde dehydrogenase	0.0221	0.2880
JW2317	pdxB	erythronate-4-phosphate dehydrogenase	0.0618	0.1044
JW2318	flk	putative flagella assembly protein	0.0132	0.4068
JW2319	yfcJ	putative arabinose efflux transporter	-0.0570	0.0014
JW2322	yfcL	uncharacterized protein	0.2553	0.0353
JW2324	yfcA	TauE/TSUP family inner membrane protein	-0.0723	0.0037
JW2325	mepA	murein DD-endopeptidase	-0.1091	0.1631
JW2326	aroC	chorismate synthase	0.2733	0.0141
JW2328	yfcN	putative DNA endonuclease	-0.0060	0.8352
JW2329	yfcO	DUF2544 family putative outer membrane protein	-0.0736	0.2228
JW2330	yfcP	putative fimbrial-like adhesin protein	0.0411	0.3136
JW2331	yfcQ	putative fimbrial-like adhesin protein	-0.1597	0.0018
JW2332	yfcR	putative fimbrial-like adhesin protein	-0.0241	0.6114
JW2333	yfcS	putative periplasmic pilin chaperone	-0.0127	0.5204
JW2334	yfcT	pseudogene, FimD fimbrial export usher family	0.0209	0.7046
JW2335	yfcU	Putative outer membrane usher protein	-0.0988	0.0035
JW2336	yfcV	putative fimbrial-like adhesin protein	0.0836	0.0900
JW2337	sixA	phosphohistidine phosphatase	0.0755	0.1737
JW2338	yfcX	enoyl-CoA hydratase/epimerase and isomerase/3-hydroxyacyl-CoA dehydrogenase	0.0048	0.9649
JW2339	yfcY	beta-ketoacyl-CoA thiolase, anaerobic, subunit	0.0070	0.8014
JW2340	yfcZ	UPF0381 family protein	0.0762	0.0000
JW2341	fadL	long-chain fatty acid outer membrane transporter	0.0858	0.0278
JW2342	yfdF	uncharacterized protein	-0.0585	0.0511
JW2343	vacJ	ABC transporter maintaining OM lipid asymmetry, OM lipoprotein component	-0.0381	0.1163
JW2344	yfdC	inner membrane protein	-0.0905	0.0005
JW2345	intS	CPS-53 (KpLE1) prophage; putative prophage CPS-53 integrase	0.0377	0.1943
JW2346	yfdG	CPS-53 (KpLE1) prophage; bactoprenol-linked glucose translocase/flippase	0.0270	0.4380
JW2347	yfdH	CPS-53 (KpLE1) prophage; bactoprenol glucosyl transferase	-0.0140	0.3024
JW2350	yfdK	CPS-53 (KpLE1) prophage; conserved protein	0.1076	0.0000
JW2352	yfdM	CPS-53 (KpLE1) prophage; putative methyltransferase	0.1235	0.0131
JW2355	yfdO	pseudogene, CPS-53 (KpLE1) prophage; bacteriophage replication protein O family	-0.0538	0.2931
JW2356	yfdP	CPS-53 (KpLE1) prophage; uncharacterized protein	-0.0334	0.0941

JW2357	yfdQ	CPS-53 (KpLE1) prophage; uncharacterized protein	0.0274	0.5299
JW2358	yfdR	CPS-53 (KpLE1) prophage; conserved protein	-0.0067	0.8320
JW2359	yfdS	CPS-53 (KpLE1) prophage; uncharacterized protein	-0.3320	0.0105
JW2361	dsdC	dsd operon activator; autorepressor	-0.0212	0.3364
JW2362	dsdX	D-serine transporter	0.0066	0.8713
JW2363	dsdA	D-serine dehydratase	-0.0597	0.0451
JW2364	emrY	putative multidrug efflux system	-0.0890	0.3215
JW2365	emrK	multidrug resistance efflux pump membrane fusion protein	-0.0184	0.4591
JW2366	evgA	response regulator in two-component regulatory system with EvgS	-0.0552	0.0541
JW2367	evgS	hybrid sensory histidine kinase in two-component regulatory system with EvgA	-0.0248	0.5989
JW2368	yfdE	acetyl-CoA:oxalate CoA-transferase	0.0151	0.6653
JW2369	yfdV	putative transporter	0.0017	0.9066
JW2370	oxc	oxalyl CoA decarboxylase, ThDP-dependent	-0.1448	0.4462
JW2371	frc	formyl-CoA transferase, NAD(P)-binding	-0.0974	0.0000
JW2372	yfdX	uncharacterized protein	-0.1949	0.0054
JW2373	ypdI	putative lipoprotein involved in colanic acid biosynthesis	0.0371	0.1284
JW2374	yfdY	DUF2545 family putative inner membrane protein	-0.0677	0.0174
JW2375	ddg	palmitoleoyl-acyl carrier protein (ACP)-dependent acyltransferase	0.0058	0.7523
JW2376	yfdZ	glutamate-pyruvate aminotransferase; glutamic-pyruvic transaminase (GPT); alanine transaminase	-0.0367	0.1572
JW2378	ypdB	response regulator activating yhjX; pyruvate-responsive YpdAB two-component system	0.0214	0.4002
JW2379	ypdC	putative DNA-binding protein	-0.0453	0.0345
JW2380	ypdD	putative PTS enzyme: Hpr, enzyme I and IIA components	-0.0547	0.1262
JW2381	ypdE	aminopeptidase	0.0679	0.0402
JW2382	ypdF	Xaa-Pro aminopeptidase	0.0431	0.0631
JW2383	ypdG	putative enzyme IIC component of PTS	-0.0033	0.9116
JW2385	glk	glucokinase	0.0302	0.1200
JW2386	yfeO	putative ion channel protein	0.0176	0.5900
JW2387	ypeC	DUF2502 family putative periplasmic protein	0.0377	0.2592
JW2388	mntH	manganese/divalent cation transporter	0.0141	0.6219
JW2389	nupC	nucleoside (except guanosine) transporter	-0.0606	0.2614

JW2393	yfeC	DUF1323 family putative DNA-binding protein	-0.0322	0.1663
JW2394	yfeD	DUF1323 family putative DNA-binding protein	0.0265	0.1582
JW2396	xapR	transcriptional activator of xapAB	-0.0500	0.1045
JW2397	xapB	xanthosine transporter	0.0029	0.9095
JW2398	xapA	purine nucleoside phosphorylase 2; nicotinamide 1-beta-D-riboside synthase	0.0463	0.2265
JW2399	yfeN	putative outer membrane protein	-0.0817	0.0184
JW2400	yfeR	transcriptional regulator of yefH	-0.0188	0.3940
JW2406	cysZ	sulfate transporter, sulfite inhibited	-0.0242	0.6761
JW2407	cysK	cysteine synthase A, O-acetylserine sulfhydrolase A subunit	0.2926	0.0003
JW2409	ptsI	PEP-protein phosphotransferase of PTS system (enzyme I)	0.0320	0.2443
JW2410	crr	glucose-specific enzyme IIA component of PTS	-0.0080	0.7517
JW2411	pdxK	pyridoxal-pyridoxamine kinase/hydroxymethylpyrimidine kinase	0.0351	0.2046
JW2412	yfeK	uncharacterized protein	-0.0989	0.0330
JW2413	yfeS	WGR domain protein	0.0182	0.7090
JW2414	cysM	cysteine synthase B (O-acetylserine sulfhydrolase B)	-0.0437	0.1120
JW2415	cysA	sulfate/thiosulfate transporter subunit	-0.2400	0.0160
JW2416	cysW	sulfate/thiosulfate ABC transporter permease	-0.0337	0.2068
JW2417	cysU	sulfate/thiosulfate ABC transporter permease	-0.0024	0.9284
JW2418	cysP	thiosulfate-binding protein	-0.2476	0.0005
JW2420	yfeT	repressor for murPQ, MurNAc 6-P inducible	0.0196	0.3906
JW2421	yfeU	N-acetylmuramic acid 6-phosphate (MurNAc-6-P) etherase	0.0201	0.0613
JW2422	murP	N-acetylmuramic acid permease, EIIBC component, PTS system	-0.0270	0.6237
JW2424	yfeX	porphyrinogen oxidase, cytoplasmic	-0.0339	0.0365
JW2425	yfeY	RpoE-regulated lipoprotein	0.0737	0.0051
JW2426	yfeZ	inner membrane protein	0.0460	0.3391
JW2427	ypeA	GNAT family putative N-acetyltransferase	-0.0307	0.2788
JW2428	amiA	N-acetylmuramoyl-l-alanine amidase I	0.0234	0.4617
JW2430	yfeG	eut operon transcriptional activator, AraC family	0.0331	0.2017
JW2431	yffI	putative ethanol utilization carboxysome structural protein	0.0561	0.1785
JW2432	eutL	putative ethanol utilization carboxysome structural protein	0.0061	0.9466
JW2433	eutC	ethanolamine ammonia-lyase, small subunit (light chain)	0.0118	0.6930
JW2434	eutB	ethanolamine ammonia-lyase, large subunit, heavy chain	0.0778	0.1627

JW2435	eutA	reactivating factor for ethanolamine ammonia lyase	-0.0701	0.0346
JW2436	eutH	ethanolamine transporter	0.0610	0.0956
JW2437	eutG	ethanol dehydrogenase involved in ethanolamine utilization; aldehyde reductase	-0.0262	0.6688
JW2438	eutJ	ethanolamine utilization protein, HSP70/actin superfamily protein	0.0518	0.4260
JW2439	eutE	aldehyde oxidoreductase, ethanolamine utilization protein	0.0872	0.0301
JW2440	cchB	Ethanolamine catabolic microcompartment shell protein	0.0158	0.4792
JW2441	cchA	ethanolamine utilization protein, putative carboxysome structural protein	-0.0805	0.0007
JW2442	eutI	phosphate acetyltransferase	0.0050	0.8288
JW2443	eutT	cobalamin adenosyltransferase involved in ethanolamine utilization	0.0675	0.1331
JW2444	eutQ	RmlC-like cupin domain protein	0.0926	0.0889
JW2445	eutP	putative P-loop NTPase ethanolamine utilization protein	-0.0010	0.9354
JW2446	ypfE	putative ethanol utilization carboxysome structural protein	0.0548	0.0867
JW2447	maeB	malic enzyme: putative oxidoreductase/phosphotransacetylase	0.0860	0.0832
JW2448	talA	transaldolase A	-0.0679	0.0294
JW2449	tktB	transketolase 2, thiamine triphosphate-binding	-0.0497	0.4470
JW2450	ypfG	DUF1176 family protein	0.1076	0.0188
JW2451	yffH	GDP-mannose pyrophosphatase	0.0667	0.0882
JW2452	aegA	putative oxidoreductase, FeS binding subunit/NAD/FAD-binding subunit	-0.1647	0.0918
JW2453	narQ	sensory histidine kinase in two-component regulatory system with NarP	0.0198	0.5278
JW2454	acrD	aminoglycoside/multidrug efflux system	-0.0135	0.7716
JW2455	yffB	putative ArsC family reductase	-0.0513	0.2036
JW2457	ypfN	putative membrane protein, UPF0370 family	-0.1107	0.0030
JW2459	ypfI	elongator methionine tRNA (ac4C34) acetyltransferase	-0.0052	0.7151
JW2460	ypfJ	putative neutral zinc metallopeptidase	0.0014	0.9123
JW2461	purC	phosphoribosylaminoimidazole-succinocarboxamide synthetase	0.0137	0.7251
JW2462	nlpB	BamABCDE complex OM biogenesis lipoprotein	0.0176	0.5400
JW2464	gcvR	transcriptional repressor, regulatory protein accessory to GcvA	0.1989	0.0003
JW2465	bcp	peroxiredoxin; thiol peroxidase, thioredoxin-dependent	0.0800	0.0217
JW2466	hyfA	hydrogenase 4, 4Fe-4S subunit	-0.0309	0.6678
JW2467	hyfB	hydrogenase 4, membrane subunit	0.1175	0.0584

JW2468	hyfC	hydrogenase 4, membrane subunit	0.0756	0.0037
JW2469	hyfD	hydrogenase 4, membrane subunit	-0.0129	0.7066
JW2470	hyfE	hydrogenase 4, membrane subunit	0.0021	0.9157
JW2471	hyfF	hydrogenase 4, membrane subunit	-0.0258	0.4550
JW2472	hyfG	hydrogenase 4, subunit	-0.0633	0.0445
JW2473	hyfH	hydrogenase 4, Fe-S subunit	-0.0523	0.4157
JW2475	hyfJ	putative processing element hydrogenase 4	0.0269	0.4446
JW2476	hyfR	hydrogenase-4 transcriptional activator	0.0712	0.2149
JW2477	focB	putative formate transporter	-0.0035	0.8672
JW2478	yfgO	putative UPF0118 family inner membrane permease	0.0079	0.5329
JW2479	yfgC	OM protein maintenance and assembly metalloprotease and chaperone, periplasmic	0.0784	0.0089
JW2480	yfgD	putative oxidoreductase	0.0164	0.4352
JW2482	uraA	uracil permease	0.0251	0.3986
JW2483	upp	uracil phosphoribosyltransferase	-0.0773	0.2397
JW2484	purM	phosphoribosylaminoimidazole synthetase	-0.0056	0.8031
JW2485	purN	phosphoribosylglycinamide formyltransferase 1	-0.0613	0.3366
JW2486	ppk	polyphosphate kinase, component of RNA degradosome	-0.0135	0.4811
JW2487	ppx	exopolyphosphatase	0.1699	0.0146
JW2488	yfgF	cyclic-di-GMP phosphodiesterase, anaerobic	-0.0289	0.1282
JW2490	yfgI	nalidixic acid resistance protein, putative periplasmic protein	-0.0616	0.1635
JW2491	guaA	GMP synthetase (glutamine aminotransferase)	-0.0198	0.2328
JW2493	xseA	exonuclease VII, large subunit	-0.0177	0.4663
JW2496	yfgL	BamABCDE complex OM biogenesis lipoprotein	0.1093	0.0171
JW2497	yfgM	ancillary SecYEG translocon subunit; putative anti-RcsB factor	0.0094	0.5549
JW2500	yfgA	MreB assembly cytoskeletal protein	0.0045	0.9238
JW2501	yfgB	dual specificity 23S rRNA m(2)A2503, tRNA m(2)A37 methyltransferase, SAM-dependent	0.0171	0.6402
JW2502	ndk	multifunctional nucleoside diphosphate kinase and apyrimidinic endonuclease and 3'-phosphodiesterase	0.1258	0.0255
JW2503	pbpC	penicillin-insensitive murein repair transglycosylase; inactive transpeptidase domain protein	0.0444	0.1014
JW2504	yfhM	bacterial alpha2-macroglobulin colonization factor ECAM; anti-host protease defense factor; periplasmic inner membrane-anchored lipoprotein	0.0318	0.1921
JW2505	sseA	3-mercaptopyruvate sulfurtransferase	0.0612	0.0305
JW2507	pepB	aminopeptidase B	0.1900	0.0098
JW2508	yfhJ	Fe(2+) donor and activity modulator for cysteine desulfurase	-0.0333	0.1848

JW2509	fdx	[2Fe-2S] ferredoxin	-0.0479	0.2900
JW2510	hscA	DnaK-like molecular chaperone specific for IscU	0.1013	0.0193
JW2511	hscB	HscA co-chaperone, J domain-containing protein Hsc56; IscU-specific chaperone HscAB	0.0111	0.4607
JW2512	iscA	FeS cluster assembly protein	0.0818	0.1244
JW2513	iscU	iron-sulfur cluster assembly scaffold protein	0.0933	0.1165
JW2514	iscS	cysteine desulfurase (tRNA sulfurtransferase), PLP-dependent	0.0116	0.5515
JW2515	iscR	isc operon transcriptional repressor; suf operon transcriptional activator; oxidative stress- and iron starvation-inducible; autorepressor	0.0688	0.0074
JW2516	yfhQ	tRNA mC32,mU32 2'-O-methyltransferase, SAM-dependent	-0.0152	0.5430
JW2518	yfhR	putative S9 family prolyl oligopeptidase	-0.0072	0.4300
JW2520	hcaT	putative 3-phenylpropionic transporter	0.0003	0.9916
JW2521	hcaR	hca operon transcriptional regulator	-0.0614	0.0122
JW2522	hcaE	3-phenylpropionate dioxygenase, large (alpha) subunit	-0.0158	0.4730
JW2523	hcaF	3-phenylpropionate dioxygenase, small (beta) subunit	-0.1066	0.0000
JW2524	hcaC	3-phenylpropionate dioxygenase, ferredoxin subunit	0.0457	0.0023
JW2525	hcaB	2,3-dihydroxy-2,3-dihydrophenylpropionate dehydrogenase	-0.0221	0.8225
JW2526	hcaD	phenylpropionate dioxygenase, ferredoxin reductase subunit	-0.7065	0.0001
JW2527	yphA	DoxX family inner membrane protein	-0.0148	0.5573
JW2528	yphB	mutarotase superfamily protein, YphB family	0.0211	0.5862
JW2530	yphD	putative sugar ABC transporter permease	0.0986	0.0011
JW2531	yphE	putative sugar ABC transporter ATPase	-0.0061	0.8655
JW2535	glyA	serine hydroxymethyltransferase	0.2974	0.0498
JW2535	glyA	serine hydroxymethyltransferase	0.3433	0.0355
JW2536	hmp	fused nitric oxide dioxygenase/dihydropteridine reductase 2	-0.2541	0.0189
JW2537	glnB	regulatory protein P-II for glutamine synthetase	0.0259	0.7961
JW2538	yfhA	response regulator regulating glmY sRNA in two-component system with sensor protein GlrK	0.0701	0.1638

JW2539	yfhG	putative outer membrane protein modulating the QseEF response	0.0638	0.1894
JW2541	purL	phosphoribosylformyl-glycineamide synthetase	-0.0006	0.9878
JW2542	yfhD	membrane-bound lytic transglycosylase F, murein hydrolase	-0.0068	0.7600
JW2545	yfhH	putative DNA-binding transcriptional regulator	0.0888	0.0022
JW2546	yfhL	putative 4Fe-4S cluster-containing protein	0.0398	0.0317
JW2548	pdxJ	pyridoxine 5'-phosphate synthase	0.0662	0.1345
JW2549	recO	gap repair protein	0.0113	0.4539
JW2553	lepA	back-translocating elongation factor EF4, GTPase	0.0425	0.0081
JW2554	rseC	SoxR iron-sulfur cluster reduction factor component	-0.0221	0.4516
JW2555	rseB	anti-sigma E factor, binds RseA	-0.1032	0.1244
JW2558	nadB	quinolinate synthase, L-aspartate oxidase (B protein) subunit	0.3788	0.0000
JW2559	yfiC	tRNA1(Val) (adenine(37)-N6)-methyltransferase	-0.0827	0.0007
JW2560	srmB	ATP-dependent RNA helicase	-0.0439	0.2081
JW2561	yfiE	putative DNA-binding transcriptional regulator	0.0209	0.4644
JW2562	yfiK	cysteine and O-acetylserine exporter	0.0154	0.4331
JW2563	yfiD	autonomous glycyl radical cofactor	-0.0160	0.4399
JW2564	ung	uracil-DNA-glycosylase	0.0208	0.4352
JW2565	yfiF	putative methyltransferase	0.0072	0.7919
JW2566	trxC	thioredoxin 2	-0.0801	0.1058
JW2568	yfiQ	protein lysine acetyltransferase	-0.0361	0.1885
JW2570	yfiM	putative lipoprotein	-0.0132	0.6138
JW2571	kgtP	alpha-ketoglutarate transporter	-0.0205	0.4281
JW2575	yfiH	UPF0124 family protein	-0.0302	0.5315
JW2576	rluD	23S rRNA pseudouridine(1911,1915,1917) synthase	-0.0270	0.1205
JW2578	yfiA	cold shock protein associated with 30S ribosomal subunit	0.0651	0.0065
JW2579	pheL	pheA gene leader peptide	0.0317	0.0143
JW2580	pheA	chorismate mutase and prephenate dehydratase, P-protein	-0.0156	0.7117
JW2581	tyrA	fused chorismate mutase T/prephenate dehydrogenase	-0.0241	0.7685
JW2584	yfiR	putative periplasmic inhibitor of YfiN activity	-0.1375	0.0377
JW2585	yfiN	putative membrane-anchored diguanylate cyclase	0.0527	0.0149
JW2592	ypjD	cytochrome c assembly protein family inner membrane protein	-0.0889	0.0014
JW2598	smpA	lipoprotein component of BamABCDE OM biogenesis complex	0.0512	0.3749
JW2599	yfjF	UPF0125 family protein	0.0202	0.3843

JW2600	yfjG	toxic UPF0083 family protein inhibitor of 70S ribosome formation	0.0622	0.0524
JW2601	smpB	tmRNA-binding trans-translation protein	-0.0466	0.0467
JW2602	intA	CP4-57 prophage; integrase	-0.0843	0.0318
JW2603	yfjH	CP4-57 prophage; uncharacterized protein	0.0089	0.4249
JW2604	alpA	CP4-57 prophage; DNA-binding transcriptional activator	-0.0441	0.2381
JW2605	yfjI	CP4-57 prophage; uncharacterized protein	-0.0801	0.0013
JW2607	yfjJ	CP4-57 prophage; uncharacterized protein	0.0525	0.0292
JW2608	yfjK	radiation resistance protein; DEAD/H helicase-like protein; CP4-57 putative defective prophage	0.0699	0.0491
JW2609	yfjL	CP4-57 putative defective prophage, DUF4297/DUF1837 polymorphic toxin family protein	-0.0327	0.0205
JW2610	yfjM	CP4-57 prophage; uncharacterized protein	-0.0211	0.2835
JW2611	yfjN	CP4-57 prophage; RNase LS	0.0155	0.6818
JW2614	yfjQ	CP4-57 prophage; uncharacterized protein	-0.0594	0.1910
JW2615	yfjR	CP4-57 prophage; putative DNA-binding transcriptional regulator	-0.2140	0.0039
JW2618	yfjT	CP4-57 prophage; putative periplasmic protein	-0.0209	0.6527
JW2619	yfjU	CP4-57 prophage; conserved protein	0.0836	0.0004
JW2621	yfjV	Uncharacterized protein	0.2336	0.0138
JW2623	yfjW	CP4-57 prophage; putative inner membrane protein	-0.0092	0.8187
JW2624	yfjX	CP4-57 prophage; putative antirestriction protein	0.1064	0.1725
JW2625	yfjY	CP4-57 prophage; putative DNA repair protein	-0.0428	0.1744
JW2626	yfjZ	CP4-57 prophage; antitoxin of the YpjF-YfjZ toxin-antitoxin system	0.0097	0.6267
JW2627	ypjF	CP4-57 prophage; toxin of the YpjF-YfjZ toxin-antitoxin system	0.0247	0.2260
JW2629	ypjB	pseudogene	-0.0457	0.0509
JW2631	ygaR	Putative uncharacterized protein	-0.0695	0.0834
JW2633	yqaD	Putative uncharacterized protein	0.0511	0.0037
JW2635	ygaF	L-2-hydroxyglutarate oxidase	0.0212	0.4235
JW2636	gabD	succinate-semialdehyde dehydrogenase I, NADP-dependent	0.0184	0.4521
JW2637	gabT	4-aminobutyrate aminotransferase, PLP-dependent	-0.0086	0.6653
JW2638	gabP	gamma-aminobutyrate transporter	-0.0599	0.0977
JW2639	csiR	transcriptional repressor of csiD	-0.0334	0.5059
JW2640	ygaU	uncharacterized protein	0.0008	0.9769
JW2642	ygaV	tributyltin-inducible repressor of ygaVP	0.1232	0.0751

JW2643	ygaP	DUF2892 family inner membrane rhodanese	-0.0182	0.6325
JW2644	stpA	DNA binding protein, nucleoid-associated	-0.0278	0.5390
JW2645	ygaW	alanine exporter, alanine-inducible, stress-responsive	-0.0165	0.4885
JW2646	ygaC	uncharacterized protein	0.0378	0.3563
JW2647	ygaM	putative membrane-anchored DUF883 family ribosome-binding protein	0.0926	0.0004
JW2648	nrdH	hydrogen donor for NrdEF electron transport system; glutaredoxin-like protein	0.0915	0.0007
JW2649	nrdI	NrdEF cluster assembly flavodoxin	0.0024	0.8950
JW2650	nrdE	ribonucleoside-diphosphate reductase 2, alpha subunit	-0.1750	0.2653
JW2651	nrdF	ribonucleoside-diphosphate reductase 2, beta subunit, ferritin-like protein	-0.0176	0.4554
JW2652	proV	glycine betaine/proline ABC transporter periplasmic binding protein	0.0104	0.7438
JW2653	proW	glycine betaine/proline ABC transporter permease	0.1381	0.0060
JW2654	proX	glycine betaine/proline ABC transporter periplasmic binding protein; cold shock protein	0.0522	0.2245
JW2655	ygaX	pseudogene, major facilitator transporter superfamily	0.1140	0.0174
JW2657	ygaZ	putative L-valine exporter, norvaline resistance protein	0.0629	0.0714
JW2658	ygaH	putative L-valine exporter, norvaline resistance protein	0.0664	0.0040
JW2659	mprA	transcriptional repressor of microcin B17 synthesis and multidrug efflux	0.0212	0.1216
JW2660	emrA	multidrug efflux system	-0.0903	0.0243
JW2661	emrB	multidrug efflux system protein	0.0445	0.0825
JW2662	luxS	S-ribosylhomocysteine lyase	-0.0338	0.7338
JW2663	gshA	glutamate-cysteine ligase	-0.1085	0.0051
JW2664	yqaA	COG1238 family inner membrane protein	0.0361	0.2573
JW2667	alaS	alanyl-tRNA synthetase	0.1678	0.0069
JW2668	recX	regulatory protein for RecA	-0.2204	0.0374
JW2669	recA	DNA recombination and repair protein; ssDNA-dependent ATPase; synaptase; ssDNA and dsDNA binding protein; ATP-dependent homologous DNA strand exchanger; recombinase A; LexA autocleavage cofactor	-0.0881	0.2977
JW2670	ygaD	nicotinamide-nucleotide amidohydrolase; NMN amidohydrolase	-0.0479	0.0775
JW2671	mltB	membrane-bound lytic murein transglycosylase B	-0.0138	0.5624
JW2673	srlB	glucitol/sorbitol-specific enzyme IIA component of PTS	0.0589	0.2675
JW2674	srlD	sorbitol-6-phosphate dehydrogenase	0.0136	0.3603

JW2675	gutM	sorbitol=responsive srl operon transcriptional activator	-0.0080	0.7956
JW2676	srlR	sorbitol-inducible srl operon transcriptional repressor	-0.0794	0.0022
JW2680	norV	anaerobic nitric oxide reductase flavorubredoxin	-0.0220	0.5326
JW2681	norW	NADH:flavorubredoxin oxidoreductase	0.0672	0.0184
JW2683	hydN	formate dehydrogenase-H, [4Fe-4S] ferredoxin subunit	-0.1696	0.0070
JW2686	ascB	cryptic 6-phospho-beta-glucosidase	0.0829	0.0184
JW2687	hycI	protease involved in processing C-terminal end of HycE	0.0126	0.7005
JW2688	hycH	hydrogenase 3 maturation protein	-0.1712	0.0076
JW2689	hycG	hydrogenase 3 and formate hydrogenase complex, HycG subunit	0.1157	0.1801
JW2690	hycF	formate hydrogenlyase complex iron-sulfur protein	0.0798	0.0029
JW2691	hycE	hydrogenase 3, large subunit	-0.0527	0.0223
JW2692	hycD	hydrogenase 3, membrane subunit	0.0059	0.9212
JW2693	hycC	hydrogenase 3, membrane subunit	-0.0016	0.9639
JW2694	hycB	hydrogenase 3, Fe-S subunit	-0.2690	0.0110
JW2695	hycA	regulator of the transcriptional regulator FhlA	0.0462	0.1139
JW2696	hypA	protein involved in nickel insertion into hydrogenases 3	0.0275	0.3217
JW2697	hypB	GTP hydrolase involved in nickel liganding into hydrogenases	0.1281	0.0000
JW2698	hypC	hydrogenase maturation protein	-0.0991	0.0016
JW2699	hypD	hydrogenase maturation protein	0.0259	0.3594
JW2700	hypE	carbamoyl dehydratase, hydrogenases 1,2,3 maturation protein	0.0263	0.2018
JW2701	fhlA	formate hydrogenlyase transcriptional activator	-0.0160	0.5560
JW2702	ygbA	uncharacterized protein	0.0346	0.1904
JW2703	mutS	methyl-directed mismatch repair protein	0.0527	0.0996
JW2704	pphB	serine/threonine-specific protein phosphatase 2	0.0591	0.0059
JW2705	ygbI	DeoR family putative transcriptional regulator	-0.1130	0.0005
JW2706	ygbJ	putative dehydrogenase	-0.0397	0.4228
JW2707	ygbK	FliA-regulated DUF1537 family protein	-0.0206	0.5396
JW2708	ygbL	putative class II aldolase	-0.0284	0.4346
JW2709	ygbM	putative hydroxypyruvate isomerase	-0.0109	0.7338
JW2710	ygbN	putative transporter	0.0755	0.1304

JW2712	nlpD	activator of AmiC murein hydrolase activity, lipoprotein	0.0133	0.8867
JW2713	pcm	L-isoaspartate protein carboxylmethyltransferase type II	-0.0509	0.0248
JW2714	surE	broad specificity 5'(3')-nucleotidase and polyphosphatase	0.0114	0.5735
JW2715	truD	tRNA(Glu) pseudouridine(13) synthase	0.0435	0.0610
JW2719	ygbE	DUF3561 family inner membrane protein	0.0599	0.0582
JW2720	cysC	adenosine 5'-phosphosulfate kinase	0.1529	0.0003
JW2721	cysN	sulfate adenylyltransferase, subunit 1	0.0892	0.3571
JW2722	cysD	sulfate adenylyltransferase, subunit 2	0.1721	0.0091
JW2723	iap	aminopeptidase in alkaline phosphatase isozyme conversion	-0.0683	0.2455
JW2725	ygbT	multifunctional endonuclease Cas1, CRISPR adaptation protein; DNA repair enzyme	-0.0009	0.9775
JW2726	ygcH	CRISPR RNA precursor cleavage enzyme; CRISP RNA (crRNA) containing Cascade antiviral complex protein	-0.0350	0.0548
JW2728	ygcJ	CRISP RNA (crRNA) containing Cascade antiviral complex protein	0.0291	0.5752
JW2729	ygcK	CRISP RNA (crRNA) containing Cascade antiviral complex protein	0.0140	0.7262
JW2730	ygcL	CRISP RNA (crRNA) containing Cascade antiviral complex protein	-0.0132	0.6059
JW2731	ygcB	Cascade complex anti-viral R-loop helicase-annealase Cas3	0.0379	0.0853
JW2732	cysH	phosphoadenosine phosphosulfate reductase; PAPS reductase, thioredoxin dependent	0.3604	0.0000
JW2733	cysI	sulfite reductase, beta subunit, NAD(P)-binding, heme-binding	0.1774	0.0000
JW2734	cysJ	sulfite reductase, alpha subunit, flavoprotein	0.1326	0.1554
JW2735	ygcM	6-pyruvoyl tetrahydrobiopterin synthase (PTPS)	0.0350	0.0245
JW2736	ygcN	putative oxidoreductase	-0.0508	0.0092
JW2737	ygcO	putative 4Fe-4S cluster-containing protein	0.3123	0.0008
JW2738	ygcP	putative antiterminator regulatory protein	0.0247	0.4482
JW2746	yqcE	putative MFS transporter, inner membrane protein	0.0866	0.0002
JW2748	ygcF	7-carboxy-7-deazaguanine synthase; queosine biosynthesis	-0.0337	0.4936
JW2752	mazG	nucleoside triphosphate pyrophosphohydrolase	-0.0887	0.0207
JW2753	chpA	mRNA interferase toxin, antitoxin is MazE	0.2457	0.0769
JW2755	relA	(p)ppGpp synthetase I/GTP pyrophosphokinase	0.0977	0.0015
JW2756	rumA	23S rRNA m(5)U1939 methyltransferase, SAM-dependent	0.0213	0.3252
JW2757	barA	hybrid sensory histidine kinase, in two-component regulatory system with UvrY	-0.1331	0.0044
JW2758	gudD	D-glucarate dehydratase 1	0.0013	0.9546

JW2759	gudX	glucarate dehydratase-related protein, substrate unknown	-0.0140	0.5001
JW2760	gudP	putative D-glucarate transporter	0.0191	0.3957
JW2761	yqcA	short-chain flavodoxin, FMN-binding	0.0354	0.3279
JW2762	yqcB	tRNA(Ile1,Asp) pseudouridine(65) synthase	0.0023	0.9098
JW2763	yqcC	DUF446 family protein	0.0566	0.0545
JW2764	syd	SecY-interacting protein	0.0048	0.9161
JW2765	yqcD	7-cyano-7-deazaguanine reductase (NADPH-dependent)	-0.0253	0.7767
JW2766	ygdH	pyrimidine/purine nucleotide 5'-monophosphate nucleosidase	0.0349	0.0314
JW2767	sdaC	putative serine transporter	0.1022	0.0137
JW2768	sdaB	L-serine dehydratase 2	0.1047	0.0501
JW2770	fucO	L-1,2-propanediol oxidoreductase	0.0236	0.4115
JW2771	fucA	L-fuculose-1-phosphate aldolase	0.1777	0.1235
JW2772	fucP	L-fucose transporter	-0.0481	0.1106
JW2773	fucI	L-fucose isomerase	-0.1635	0.0836
JW2774	fucK	L-fuculokinase	0.0104	0.5853
JW2775	fucU	L-fucose mutarotase	0.0421	0.2240
JW2776	fucR	l-fucose operon activator	-0.0442	0.0801
JW2777	ygdE	23S rRNA C2498 2'-O-ribose methyltransferase, SAM-dependent	0.1469	0.0183
JW2778	ygdD	UPF0382 family inner membrane protein	0.1703	0.0001
JW2779	gcvA	glycine cleavage system transcriptional activator; autorepressor	-0.0229	0.6407
JW2781	csdA	cysteine sulfinate desulfinase	-0.0371	0.1088
JW2782	ygdK	CsdA-binding activator; Fe-S protein	-0.0467	0.0465
JW2784	mltA	membrane-bound lytic murein transglycosylase A	0.0013	0.9749
JW2786	argA	amino acid N-acetyltransferase and inactive acetylglutamate kinase	-0.0447	0.6990
JW2787	recD	exonuclease V (RecBCD complex), alpha chain	0.0653	0.1949
JW2788	recB	exonuclease V (RecBCD complex), beta subunit	-0.0654	0.5322
JW2790	recC	exonuclease V (RecBCD complex), gamma chain	0.0954	0.1783
JW2791	ppdC	putative prepilin peptidase-dependent protein	0.0207	0.4434
JW2794	ppdA	putative prepilin peptidase-dependent protein	0.0557	0.1047
JW2795	thyA	thymidylate synthetase	-0.0134	0.0626
JW2797	ptsP	PEP-protein phosphotransferase enzyme I; GAF domain containing protein	-0.0546	0.2101
JW2798	nudH	RNA pyrophosphohydrolase	-0.1258	0.3207
JW2799	mutH	methyl-directed mismatch repair protein	-0.0750	0.2108
JW2800	ygdQ	UPF0053 family inner membrane protein	0.0217	0.7478

JW2801	ygdR	DUF903 family verified lipoprotein	-0.1008	0.0168
JW2802	tas	putative NADP(H)-dependent aldo-keto reductase	0.0205	0.5772
JW2803	ygeD	lysophospholipid transporter	-0.0353	0.0729
JW2804	aas	fused 2-acylglycerophospho-ethanolamine acyl transferase/acyl-acyl carrier protein synthetase	0.0879	0.0067
JW2805	galR	galactose-inducible d-galactose regulon transcriptional repressor; autorepressor	-0.2876	0.0138
JW2806	lysA	diaminopimelate decarboxylase, PLP-binding	0.0516	0.0775
JW2807	lysR	transcriptional activator of lysA; autorepressor	0.1592	0.0001
JW2808	ygeA	Asp/Glu_racemase family protein	0.0073	0.6703
JW2809	araE	arabinose transporter	0.0368	0.3448
JW2810	kduD	2-dehydro-3-deoxy-D-gluconate 5-dehydrogenase; KDG oxidoreductase; 20-ketosteroid reductase	0.0038	0.9359
JW2811	kduI	hexuronate isomerase	-0.1385	0.1049
JW2813	yqeG	putative transporter	0.0567	0.3052
JW2815	yqeI	putative transcriptional regulator	0.0044	0.9160
JW2817	yqeK	uncharacterized protein	-0.3085	0.0502
JW2818	ygeF	pseudogene	-0.0541	0.1619
JW2819	ygeG	SycD-like chaperone family TPR-repeat-containing protein	0.0744	0.0898
JW2820	ygeH	putative transcriptional regulator	0.0171	0.2244
JW2824	ygeL	Putative uncharacterized protein	0.0875	0.0164
JW2831	ygeP	pseudogene, glycosyl hydrolase family 15, part of T3SS PAI ETT2 remnant	-0.0196	0.1273
JW2833	ygeR	LysM domain-containing M23 family putative peptidase; septation lipoprotein	-0.0293	0.4907
JW2835	xdhB	xanthine dehydrogenase, FAD-binding subunit	0.0090	0.8525
JW2836	xdhC	xanthine dehydrogenase, Fe-S binding subunit	0.0840	0.2549
JW2837	ygeV	putative sigma-54-interacting transcriptional activator	0.0138	0.6525
JW2839	ygeX	2,3-diaminopropionate ammonia lyase, PLP-dependent	-0.1030	0.0248
JW2840	ygeY	putative peptidase	-0.1933	0.0096
JW2841	hyuA	D-stereospecific phenylhydantoinase	0.0042	0.9204
JW2842	yqeA	putative amino acid kinase	0.0373	0.6140
JW2843	yqeB	XdhC-CoxI family protein with NAD(P)-binding Rossman fold	-0.0869	0.0114
JW2845	ygfJ	CTP:molybdopterin cytidylyltransferase	-0.0133	0.5067

JW2848	ygfM	putative oxidoreductase	-0.0558	0.1460
JW2849	xdhD	putative hypoxanthine oxidase, molybdopterin-binding/Fe-S binding	0.0406	0.0736
JW2850	ygfO	xanthine permease	0.1879	0.0630
JW2857	idi	isopentenyl diphosphate isomerase	0.0709	0.2549
JW2858	lysS	lysine tRNA synthetase, constitutive	-0.0759	0.3401
JW2860	recJ	ssDNA exonuclease, 5' --> 3'-specific	-0.0459	0.1438
JW2861	dsbC	protein disulfide isomerase II	0.0541	0.0571
JW2862	xerD	site-specific tyrosine recombinase	0.0319	0.3310
JW2863	fldB	flavodoxin 2	0.1652	0.0008
JW2865	ygfY	flavinator of succinate dehydrogenase; antitoxin of CptAB toxin-antitoxin pair	0.1886	0.0000
JW2866	ygfZ	iron-sulfur cluster repair protein, plumbagin resistance	-0.3678	0.0001
JW2867	yqfA	hemolysin III family HylIII inner membrane protein	0.1694	0.0026
JW2868	yqfB	UPF0267 family protein	-0.0912	0.2583
JW2869	bglA	6-phospho-beta-glucosidase A	0.0395	0.1475
JW2870	ygfF	putative NAD(P)-dependent oxidoreductase	-0.0053	0.8161
JW2871	gcvP	glycine decarboxylase, PLP-dependent, subunit P of glycine cleavage complex	-0.1392	0.0016
JW2872	gcvH	glycine cleavage system lipoylprotein H, methylamine group carrier	0.0172	0.8265
JW2873	gcvT	aminomethyltransferase, tetrahydrofolate-dependent, subunit (T protein) of glycine cleavage complex	-0.0314	0.2921
JW2874	visC	2-octaprenylphenol hydroxylase, FAD-dependent	-0.0584	0.2243
JW2875	ubiH	2-octaprenyl-6-methoxyphenol hydroxylase, FAD/NAD(P)-binding	-0.0838	0.1794
JW2876	pepP	proline aminopeptidase P II	0.0342	0.4681
JW2878	zapA	FtsZ stabilizer	0.0086	0.7267
JW2879	ygfA	5-formyltetrahydrofolate cyclo-ligase family protein	0.0218	0.2953
JW2880	serA	D-3-phosphoglycerate dehydrogenase	0.2727	0.0124
JW2880	serA	D-3-phosphoglycerate dehydrogenase	0.4792	0.0008
JW2882	yqfE	pseudogene, LysR family	0.0051	0.9216
JW2883	argP	transcriptional regulator for arginine transport and DNA replication genes; replication initiation inhibitor	-0.0009	0.9603
JW2884	yliK	methylmalonyl-CoA mutase	-0.0669	0.0803
JW2885	argK	membrane ATPase/protein kinase	0.1142	0.0762
JW2886	ygfG	methylmalonyl-CoA decarboxylase, biotin-independent	-0.0039	0.8566
JW2887	ygfH	propionyl-CoA:succinate CoA transferase	-0.0039	0.8280
JW2889	yggE	oxidative stress defense protein	0.2526	0.0275

JW2890	argO	arginine transporter	0.0227	0.6035
JW2891	mscS	mechanosensitive channel protein, small conductance	0.0045	0.9218
JW2894	epd	D-erythrose 4-phosphate dehydrogenase	0.0241	0.4447
JW2895	yggC	putative PanK family P-loop kinase	-0.0970	0.4822
JW2896	yggD	Fumarase E; MtlR family putative transcriptional repressor	-0.1884	0.0362
JW2897	yggF	fructose 1,6 bisphosphatase isozyme	-0.2135	0.0004
JW2900	cmtA	putative mannitol-specific PTS IIB and IIC components	0.0146	0.2888
JW2901	cmtB	putative mannitol-specific enzyme IIA component of PTS	0.0423	0.5284
JW2903	yggG	Phe-Phe periplasmic metalloprotease, OM lipoprotein; low salt-inducible; Era-binding heat shock protein	-0.0625	0.1726
JW2904	speB	agmatinase	0.0135	0.6676
JW2905	speA	biosynthetic arginine decarboxylase, PLP-binding	0.0034	0.9106
JW2906	yqgB	uncharacterized protein	-0.0526	0.0517
JW2907	yqgC	uncharacterized protein	0.0352	0.2748
JW2910	galP	D-galactose transporter	-0.0024	0.9644
JW2911	sprT	Zn-dependent metalloprotease-related protein	-0.2664	0.0138
JW2912	endA	DNA-specific endonuclease I	-0.0587	0.1127
JW2913	yggJ	16S rRNA m(3)U1498 methyltransferase, SAM-dependent	-0.2497	0.0000
JW2914	gshB	glutathione synthetase	-0.2188	0.0002
JW2915	yqgE	uncharacterized protein	0.0361	0.2199
JW2917	yggR	putative PilT family AAA+ ATPase	0.0343	0.2793
JW2918	yggS	UPF0001 family protein, PLP-binding	-0.0894	0.0330
JW2919	yggT	putative inner membrane protein; compensates for loss in K+ uptake	-0.0363	0.1986
JW2921	yggV	dITP/XTP pyrophosphatase	0.1566	0.1090
JW2922	yggW	HemN family putative oxidoreductase	-0.0710	0.4749
JW2923	yggM	DUF1202 family putative secreted protein	0.0084	0.7385
JW2924	ansB	periplasmic L-asparaginase 2	-0.0767	0.0284
JW2925	yggN	DUF2884 family putative periplasmic protein	-0.0251	0.2821
JW2926	yggL	DUF469 family protein	0.1013	0.3454
JW2927	yggH	tRNA m(7)G46 methyltransferase, SAM-dependent	0.1198	0.0221
JW2928	mutY	adenine DNA glycosylase	0.0685	0.0148
JW2929	yggX	oxidative damage protective factor for iron-sulfur proteins	-0.4504	0.0025
JW2932	nupG	nucleoside transporter	-0.0462	0.5005
JW2934	yqgA	DUF554 family putative inner membrane protein	-0.1322	0.0299
JW2935	yghD	putative membrane-anchored secretion pathway M-type protein	-0.1546	0.0010
JW2938	yghG	secretin (GspDbeta) OM localization lipoprotein pilotin	-0.0569	0.0097

JW2939	pppA	bifunctional prepilin leader peptidase/ methylase	0.0784	0.0186
JW2942	yghK	glycolate transporter	0.2929	0.0001
JW2943	glcB	malate synthase G	-0.1080	0.0346
JW2944	glcG	DUF336 family protein	0.0637	0.0142
JW2946	glcD	glycolate oxidase subunit, FAD-linked	-0.0912	0.0612
JW2952	yghR	putative ATP-binding protein	0.2218	0.0687
JW2954	yghT	putative ATP-binding protein	-0.1636	0.0003
JW2955	pitB	phosphate transporter	0.0033	0.9200
JW2956	gss	glutathionylspermidine amidase and glutathionylspermidine synthetase	0.1518	0.0112
JW2958	hybG	hydrogenase 2 accessory protein	-0.2732	0.0424
JW2960	hybE	hydrogenase 2-specific chaperone	0.0983	0.0065
JW2961	hybD	maturation protease for hydrogenase 2	-0.2096	0.0135
JW2962	hybC	hydrogenase 2, large subunit	0.0134	0.5824
JW2964	hybA	hydrogenase 2 4Fe-4S ferredoxin-type component	0.0037	0.8841
JW2965	hybO	hydrogenase 2, small subunit	-0.1260	0.0113
JW2966	yghW	DUF2623 family protein	0.0641	0.2697
JW2970	yghZ	L-glyceraldehyde 3-phosphate reductase	-0.0278	0.6064
JW2971	yqhA	UPF0114 family putative inner membrane protein	-0.0358	0.7613
JW2972	yghA	putative oxidoreductase	0.0486	0.1670
JW2973	exbD	membrane spanning protein in TonB-ExbB-ExbD complex	0.1366	0.0006
JW2974	exbB	membrane spanning protein in TonB-ExbB-ExbD complex	0.2407	0.0000
JW2975	metC	cystathionine beta-lyase, PLP-dependent	-0.0643	0.5763
JW2976	yghB	general envelope maintenance protein; DedA family inner membrane protein	-0.0512	0.0937
JW2978	yqhD	aldehyde reductase, NADPH-dependent	-0.0017	0.9485
JW2982	yqhH	outer membrane lipoprotein, Lpp paralog	0.0346	0.0859
JW2987	parC	DNA topoisomerase IV, subunit A	-0.0009	0.9853
JW2988	ygiS	putative ABC transporter permease	0.0017	0.9438
JW2990	ygiU	GCU-specific mRNA interferase toxin of the MqsR-MqsA toxin-antitoxin system; biofilm/motility regulator; anti-repressor	0.0564	0.1646
JW2992	ygiW	hydrogen peroxide and cadmium resistance periplasmic protein; stress-induced OB-fold protein	0.0225	0.5452
JW2993	qseB	quorum sensing DNA-binding response regulator in two-component regulatory system with QseC	-0.1000	0.0023
JW2994	qseC	quorum sensing sensory histidine kinase in two-component regulatory system with QseB	-0.0205	0.1861
JW2995	ygiZ	inner membrane protein	-0.7512	0.0000
JW2996	mdaB	NADPH quinone reductase	-0.0093	0.8086
JW2997	ygiN	quinol monooxygenase	-0.1015	0.0165

JW2999	yqiA	acyl CoA esterase	-0.1920	0.0983
JW3000	cpdA	3',5' cAMP phosphodiesterase	0.0032	0.9216
JW3001	yqiB	DUF1249 protein YqiB	0.0738	0.3012
JW3002	nudF	ADP-ribose pyrophosphatase	-0.1753	0.0089
JW3006	ygiC	ATP-Grasp family ATPase	0.0094	0.7871
JW3007	zupT	4,5- DOPA-extradiol-dioxygenase	-0.0697	0.0438
JW3008	ygiE	zinc transporter	0.0088	0.2995
JW3011	ygiL	putative fimbrial-like adhesin protein	-0.0040	0.8794
JW3021	glgS	motility and biofilm regulator	-0.0171	0.5699
JW3022	yqiJ	DUF1449 family inner membrane protein	-0.1502	0.0118
JW3023	yqiK	PHB family membrane protein, function unknown	0.1009	0.0776
JW3024	rfaE	heptose 7-phosphate kinase and heptose 1-phosphate adenyltransferase	-0.0509	0.0119
JW3025	glnE	fused deadenylyltransferase/adenylyltransferase for glutamine synthetase	-0.0506	0.2991
JW3026	ygiF	inorganic triphosphatase	-0.1030	0.0841
JW3027	htrG	SH3 domain protein	0.1496	0.0082
JW3029	bacA	undecaprenyl pyrophosphate phosphatase	-0.0247	0.4045
JW3030	folB	dihydroneopterin aldolase and dihydroneopterin triphosphate 2'-epimerase	0.0144	0.7101
JW3031	ygiH	putative glycerol-3-phosphate acyltransferase	-0.0359	0.0881
JW3032	ygiP	transcriptional activator of ttdABT	0.0182	0.6034
JW3033	ttdA	L-tartrate dehydratase, alpha subunit	-0.1129	0.0739
JW3034	ttdB	L-tartrate dehydratase, beta subunit	-0.0795	0.0017
JW3035	ygjE	L-tartrate/succinate antiporte	0.0400	0.4045
JW3037	rpsU	30S ribosomal subunit protein S21	0.0289	0.3314
JW3038	dnaG	DNA primase	0.0939	0.0001
JW3039	rpoD	RNA polymerase, sigma 70 (sigma D) factor	0.1155	0.0002
JW3040	ygjF	G/U mismatch-specific DNA glycosylase; xanthine DNA glycosylase	-0.1928	0.0861
JW3041	yqjH	putative siderophore interacting protein	0.0017	0.9618
JW3042	yqjI	PadR family putative transcriptional regulator	-0.0829	0.2529
JW3043	aer	fused signal transducer for aerotaxis sensory component/methyl accepting chemotaxis component	-0.0251	0.2264
JW3045	ygjH	putative tRNA binding protein; putative tRNA corner chaperone	-0.0140	0.7679
JW3048	ebgC	evolved beta-D-galactosidase, beta subunit; cupin superfamily	0.0045	0.7778
JW3050	ygjJ	putative periplasmic protein	-0.0342	0.4058

JW3051	ygjK	alpha-glucosidase	0.0339	0.5604
JW3052	fadH	2,4-dienoyl-CoA reductase, NADH and FMN-linked	0.0176	0.6414
JW3053	ygjM	antitoxinof the HigB-HigA toxin-antitoxin system	-0.0529	0.1355
JW3054	ygjN	mRNA interferase toxin of the HigB-HigA toxin-antitoxin system	-0.2247	0.0179
JW3057	ygjQ	DUF218 superfamily protein	-0.0711	0.4994
JW3058	ygjR	putative NAD(P)-dependent dehydrogenase	0.0048	0.7455
JW3061	ygjV	Imp-YgjV family inner membrane protein	0.0093	0.8132
JW3062	uxaA	altronate hydrolase	-0.0900	0.0009
JW3063	uxaC	uronate isomerase	-0.0051	0.8832
JW3064	exuT	hexuronate transporter	-0.0811	0.0506
JW3065	exuR	hexuronate regulon transcriptional repressor; autorepressor	-0.2045	0.0002
JW3066	yqjA	general envelope maintenance protein; DedA family inner membrane protein	-0.2655	0.0245
JW3069	yqjD	membrane-anchored ribosome-binding protein	-0.0256	0.3730
JW3070	yqjE	DUF1469 family inner membrane protein	-0.0307	0.3392
JW3071	yqjK	uncharacterized protein	-0.1003	0.0005
JW3073	yqjG	putative S-transferase	-0.0344	0.4732
JW3074	yhaH	DUF805 family inner membrane protein,	-0.3772	0.0006
JW3075	yhaI	DUF805 family inner membrane protein	-0.1364	0.0000
JW3076	yhaJ	transcription regulator of quinol-like compound degradation	0.0433	0.1514
JW3077	yhaK	dinitrotoluene degradation protein, redox-sensitive bicupin	-0.0353	0.1058
JW3087	tdcC	L-threonine/L-serine transporter	-0.2734	0.0111
JW3088	tdcB	L-threonine dehydratase, catabolic	-0.0811	0.0117
JW3089	tdcA	tdc operon transcriptional activator	0.0262	0.2407
JW3091	yhaB	uncharacterized protein	0.1017	0.0014
JW3092	yhaC	pentapetide repeats-related protein	-0.1019	0.0129
JW3093	garK	glycerate kinase I	-0.0739	0.0021
JW3095	garL	alpha-dehydro-beta-deoxy-D-glucarate aldolase	-0.0744	0.1067
JW3096	garP	putative (D)-galactarate transporter	0.0392	0.1571
JW3097	garD	D-galactarate dehydrogenase	-0.2433	0.0303
JW3098	sohA	antitoxin of the SohA(PrlF)-YhaV toxin-antitoxin system	0.0964	0.1212
JW3099	yhaV	toxin of the SohB(PrlF)-YhaV toxin-antitoxin system	-0.0282	0.5692
JW3100	agaR	transcriptional repressor of the aga regulon	-0.0492	0.4235
JW3101	kbaZ	tagatose 6-phosphate aldolase 1, kbaZ subunit	-0.0315	0.3916
JW3102	agaV	N-acetylgalactosamine-specific enzyme IIB component of PTS	-0.0002	0.9948

JW3103	agaW	pseudogene, N-acetylgalactosamine-specific enzyme IIC component of PTS	-0.0763	0.0651
JW3105	agaS	tagatose-6-phosphate ketose/aldose isomerase	-0.1153	0.0079
JW3106	kbaY	tagatose 6-phosphate aldolase 1, kbaY subunit	0.0947	0.0405
JW3107	agaB	N-acetylgalactosamine-specific enzyme IIB component of PTS	0.0592	0.3768
JW3108	agaC	N-acetylgalactosamine-specific enzyme IIC component of PTS	0.0513	0.2955
JW3109	agaD	N-acetylgalactosamine-specific enzyme IID component of PTS	0.2256	0.0036
JW3110	agaI	galactosamine-6-phosphate isomerase	0.0064	0.8384
JW3111	yraH	putative fimbrial-like adhesin protein	-0.1487	0.3013
JW3112	yraI	putative periplasmic pilin chaperone	0.0645	0.0652
JW3113	yraJ	putative outer membrane protein	-0.0285	0.2948
JW3114	yraK	putative fimbrial-like adhesin protein	0.1548	0.0086
JW3116	yraM	OM lipoprotein stimulator of MrcA transpeptidase	0.0005	0.9875
JW3117	yraN	UPF0102 family protein	-0.0174	0.6872
JW3118	yraO	DnaA initiator-associating factor for replication initiation	-0.0504	0.2301
JW3119	yraP	outer membrane lipoprotein	-0.1796	0.0049
JW3120	yraQ	putative inner membrane permease	0.0858	0.2823
JW3123	yhbP	UPF0306 family protein	-0.0127	0.8312
JW3124	yhbQ	GIY-YIG nuclease superfamily protein	-0.0437	0.3429
JW3125	yhbS	GNAT family putative N-acetyltransferase	0.1106	0.0169
JW3127	yhbU	U32 peptidase family protein	-0.0550	0.1460
JW3129	yhbW	putative luciferase-like monooxygenase	0.1583	0.0401
JW3130	mtr	tryptophan transporter of high affinity	0.0148	0.6290
JW3132	nlpI	lipoprotein involved in osmotic sensitivity and filamentation	-0.0275	0.2595
JW3134	rpsO	30S ribosomal subunit protein S15	-0.0178	0.3755
JW3135	truB	tRNA pseudouridine synthase B: tRNA pseudouridine(55) synthase and putative tmRNA pseudouridine(342) synthase	-0.0902	0.0016
JW3136	rbfA	30s ribosome binding factor	-0.2261	0.0885
JW3140	argG	argininosuccinate synthetase	0.1090	0.0064
JW3142	secG	preprotein translocase membrane subunit	0.0540	0.2520
JW3143	glmM	phosphoglucosamine mutase	0.0383	0.4195
JW3144	folP	7,8-dihydropteroate synthase	0.1940	0.0594
JW3146	rrmJ	23S rRNA U2552 2'-O-ribose methyltransferase, SAM-dependent	0.0963	0.0398
JW3147	yhbY	RNA binding protein associated with pre-50S ribosomal subunits	0.0124	0.7636

JW3148	greA	transcript cleavage factor	0.0719	0.0993
JW3149	dacB	D-alanyl-D-alanine carboxypeptidase	-0.0921	0.0647
JW3151	yhbE	EamA family inner membrane putative transporter	-0.0500	0.0250
JW3155	sfsB	malPQ operon transcriptional activator	0.0580	0.0117
JW3157	yrbA	acid stress protein; putative BolA family transcriptional regulator	-0.0527	0.2900
JW3159	yrbC	ABC transporter maintaining OM lipid asymmetry, periplasmic binding protein	-0.0842	0.0450
JW3160	yrbD	OM lipid asymmetry maintenance protein; membrane-anchored ABC family periplasmic binding protein	-0.0210	0.5288
JW3161	yrbE	ABC transporter maintaining OM lipid asymmetry, inner membrane permease protein	-0.0271	0.2238
JW3162	yrbF	ABC transporter maintaining OM lipid asymmetry, ATP-binding protein	-0.0070	0.8133
JW3163	yrbG	putative calcium/sodium:proton antiporter	0.0409	0.2933
JW3164	kdsD	D-arabinose 5-phosphate isomerase	-0.1797	0.0087
JW3165	kdsC	3-deoxy-D-manno-octulosonate 8-phosphate phosphatase	-0.0222	0.6081
JW3168	yhbG	lipopolysaccharide export ABC transporter ATPase	0.1559	0.0002
JW3169	rpoN	RNA polymerase, sigma 54 (sigma N) factor	0.0355	0.0017
JW3170	yhbH	ribosome hibernation promoting factor HPF; stabilizes 100S dimers	0.0567	0.2232
JW3171	ptsN	sugar-specific enzyme IIA component of PTS	0.0675	0.1369
JW3172	yhbJ	adaptor protein for GlmZ/GlmY sRNA decay, glucosamine-6-phosphate-regulated; NTPase	0.0287	0.5445
JW3173	npr	phosphohistidinoprotein-hexose phosphotransferase component of N-regulated PTS system (Npr)	-0.0304	0.6682
JW3174	yrbL	Mg(2+)-starvation-stimulated protein	0.0629	0.0300
JW3175	mtgA	biosynthetic peptidoglycan transglycosylase	0.0688	0.2245
JW3176	elbB	isoprenoid biosynthesis protein with amidotransferase-like domain	0.0183	0.2726
JW3178	yhcC	putative Fe-S oxidoreductase, Radical SAM superfamily protein	-0.0584	0.0386
JW3179	gltB	glutamate synthase, large subunit	-0.0269	0.1665
JW3180	gltD	glutamate synthase, 4Fe-4S protein, small subunit	0.0072	0.7297
JW3181	gltF	periplasmic protein	-0.0106	0.6636
JW3182	yhcA	putative periplasmic chaperone protein	0.0242	0.2657

JW3183	yhcD	putative outer membrane fimbrial subunit usher protein	-0.0242	0.5375
JW3184	yhcE	UPF0056 membrane protein	0.0323	0.0715
JW3188	yhcF	putative transcriptional regulator	-0.0339	0.0262
JW3189	yhcG	DUF1016 family protein in the PD-(D/E)XK nuclease superfamily	-0.0319	0.2749
JW3190	yhcH	DUF386 family protein, cupin superfamily	0.0222	0.5333
JW3192	nanE	putative N-acetylmannosamine-6-P epimerase	0.0155	0.4514
JW3193	nanT	sialic acid transporter	0.1297	0.0005
JW3194	nanA	N-acetylneuraminate lyase	0.0121	0.8509
JW3195	nanR	sialic acid-inducible nan operon repressor	0.0134	0.6477
JW3196	dcuD	putative transporter	-0.0173	0.6919
JW3197	sspB	ClpXP protease specificity enhancing factor	0.0261	0.3716
JW3198	sspA	stringent starvation protein A, phage P1 late gene activator, RNAP-associated acid-resistance protein, inactive glutathione S-transferase homolog	-0.2712	0.0004
JW3201	yhcM	divisome ATPase	0.0580	0.1727
JW3203	degQ	serine endoprotease, periplasmic	-0.0569	0.2403
JW3205	mdh	malate dehydrogenase, NAD(P)-binding	0.1533	0.0167
JW3206	argR	l-arginine-responsive arginine metabolism regulon transcriptional regulator	-0.0462	0.0131
JW3208	yhcO	putative barnase inhibitor	0.1650	0.0751
JW3209	aaeB	p-hydroxybenzoic acid efflux system component	0.0697	0.0007
JW3210	aaeA	p-hydroxybenzoic acid efflux system component	-0.0498	0.1569
JW3212	aaeR	transcriptional regulator for aaeXAB operon	0.0476	0.1161
JW3213	tldD	putative peptidase	0.1036	0.1773
JW3216	rng	ribonuclease G	0.0571	0.0118
JW3217	yhdE	dTTP/UTP pyrophosphatase; m(5)UTP/m(5)CTP/pseudo-UTP pyrophosphatase	-0.0658	0.0034
JW3221	yhdA	targeting factor for csrBC sRNA degradation	0.1065	0.0000
JW3222	yhdH	putative acryloyl-CoA reductase	-0.0315	0.2297
JW3225	yhdT	DUF997 family putative inner membrane protein	0.0803	0.0001
JW3226	panF	pantothenate:sodium symporter	-0.0220	0.7277
JW3227	prmA	methyltransferase for 50S ribosomal subunit protein L11	-0.0167	0.5922
JW3228	dusB	tRNA-dihydrouridine synthase B	-0.0153	0.7669
JW3229	fis	global DNA-binding transcriptional dual regulator	-0.1567	0.0050

JW3231	yhdU	putative membrane protein	0.0718	0.1066
JW3232	envR	acrAB operon transcriptional repressor	-0.0445	0.0762
JW3233	acrE	cytoplasmic membrane lipoprotein	-0.0265	0.0605
JW3234	acrF	multidrug efflux system protein	-0.1021	0.0002
JW3235	yhdV	putative outer membrane protein	0.1159	0.0005
JW3236	yhdW	pseudogene, ABC transporter periplasmic binding protein family	-0.0552	0.0215
JW3239	yhdZ	putative amino acid ABC transporter ATPase	-0.0091	0.7570
JW3241	yrdB	DUF1488 family protein	-0.0395	0.0560
JW3242	aroE	dehydroshikimate reductase, NAD(P)-binding	0.0049	0.9181
JW3245	smg	DUF494 family putative periplasmic protein	-0.0636	0.0184
JW3250	rsmB	16S rRNA m(5)C967 methyltransferase, SAM-dependent	0.0541	0.0635
JW3251	trkA	NAD-binding component of TrK potassium transporter	-0.1316	0.0151
JW3252	mscL	mechanosensitive channel protein, high conductance	0.0105	0.6998
JW3253	yhdL	alternate ribosome-rescue factor A	-0.2504	0.0234
JW3254	zntR	zntA gene transcriptional activator	-0.0120	0.5544
JW3255	yhdN	DUF1992 family protein	-0.0141	0.7519
JW3261	rpmJ	50S ribosomal subunit protein L36	0.0288	0.2604
JW3284	pioO	part of gsp divergon involved in type II protein secretion	-0.1055	0.0175
JW3285	gspA	general secretory pathway component, cryptic	-0.0073	0.7745
JW3286	gspC	general secretory pathway component, cryptic	-0.0613	0.0907
JW3288	gspE	general secretory pathway component, cryptic	0.0918	0.1256
JW3289	gspF	general secretory pathway component, cryptic	0.0543	0.0210
JW3290	gspG	pseudopilin, cryptic, general secretion pathway	-0.0499	0.4155
JW3291	gspH	putative general secretory pathway component, cryptic	-0.0660	0.0754
JW3293	gspJ	putative general secretory pathway component, cryptic	0.1456	0.0177
JW3294	gspK	general secretory pathway component, cryptic	-0.0354	0.1375
JW3297	gspO	bifunctional prepilin leader peptidase/ methylase	0.2365	0.0283
JW3298	bfr	bacterioferritin, iron storage and detoxification protein	-0.0737	0.0918
JW3299	bfd	bacterioferritin-associated ferredoxin	-0.3630	0.0134
JW3300	chiA	periplasmic endochitinase	-0.0320	0.4756
JW3301	tufA	translation elongation factor EF-Tu 1	0.0820	0.0091
JW3305	yheL	mnm(5)-s(2)U34-tRNA synthesis 2-thiolation protein	-0.0161	0.7259

JW3306	yheM	mnm(5)-s(2)U34-tRNA synthesis 2-thiolation protein	-0.0011	0.9733
JW3307	yheN	sulfurtransferase for 2-thiolation step of mnm(5)-s(2)U34-tRNA synthesis	-0.1557	0.2285
JW3309	fkpA	FKBP-type peptidyl-prolyl cis-trans isomerase (rotamase)	-0.0040	0.9298
JW3310	slyX	phi X174 lysis protein	-0.0751	0.2198
JW3311	slyD	FKBP-type peptidyl prolyl cis-trans isomerase (rotamase)	-0.0323	0.4411
JW3312	yheV	DUF2387 family putative metal-binding protein	-0.1311	0.0196
JW3313	kefB	potassium:proton antiporter	-0.0473	0.2567
JW3314	kefG	potassium-efflux system ancillary protein for KefB, glutathione-regulated	-0.0964	0.0008
JW3315	yheS	ABC-F family protein predicted regulatory ATPase	0.0309	0.2323
JW3316	yheT	UPF0017 family putative hydrolase	-0.0329	0.5264
JW3317	yheU	UPF0270 family protein	0.0010	0.9710
JW3318	prkB	putative phosphoribulokinase	-0.0390	0.1254
JW3319	yhfA	OsmC family protein	0.0472	0.0160
JW3322	argD	bifunctional acetylornithine aminotransferase and succinyldiaminopimelate aminotransferase	-0.0207	0.5237
JW3323	pabA	aminodeoxychorismate synthase, subunit II	-0.0818	0.0001
JW3324	fic	stationary phase-induced protein, putative toxin	0.0441	0.0948
JW3325	yhfG	putative antitoxin for Fic	-0.0303	0.3804
JW3326	ppiA	peptidyl-prolyl cis-trans isomerase A (rotamase A)	-0.0597	0.0251
JW3327	tsgA	putative transporter	0.0756	0.1295
JW3328	nirB	nitrite reductase, large subunit, NAD(P)H-binding	-0.0875	0.0858
JW3329	nirD	nitrite reductase (NADH) small subunit	0.0926	0.0393
JW3330	nirC	nitrite transporter	0.0864	0.0378
JW3331	cysG	fused siroheme synthase 1,3-dimethyluroporphyriongen III dehydrogenase and siroheme ferrochelatase/uroporphyrinogen methyltransferase	-0.0195	0.4187
JW3332	yhfL	small lipoprotein	-0.0170	0.7325
JW3333	frlA	putative fructoselysine transporter	0.0902	0.0003
JW3337	frlD	fructoselysine 6-kinase	-0.0119	0.6213
JW3339	yhfS	FNR-regulated pyridoxal phosphate-dependent aminotransferase family protein	0.0634	0.0556
JW3340	yhfT	inner membrane protein	-0.0572	0.4518
JW3342	php	phosphotriesterase homology protein	-0.0078	0.8993

JW3343	yhfW	phosphopentomutase-related metalloenzyme superfamily protein	0.0431	0.0795
JW3344	yhfX	putative pyridoxal 5'-phosphate binding protein	0.0027	0.8759
JW3348	gph	phosphoglycolate phosphatase	0.0178	0.4983
JW3349	rpe	D-ribulose-5-phosphate 3-epimerase	-0.0382	0.5901
JW3350	dam	DNA adenine methyltransferase	0.2377	0.0000
JW3351	damX	cell division protein that binds to the septal ring	0.1784	0.0000
JW3352	aroB	3-dehydroquinate synthase	-0.0080	0.8193
JW3354	hofQ	DNA catabolic putative fimbrial transporter	-0.0297	0.1910
JW3356	yrfB	DNA catabolic protein	0.0066	0.8395
JW3357	yrfC	DNA catabolic putative fimbrial assembly protein	0.0243	0.2980
JW3359	mrcA	penicillin-binding protein 1a, murein transglycosylase and transpeptidase	-0.0539	0.1493
JW3360	nudE	adenosine nucleotide hydrolase; Ap3A/Ap2A/ADP-ribose/NADH hydrolase	-0.0253	0.3060
JW3363	hslR	ribosome-associated heat shock protein Hsp15	-0.0547	0.0068
JW3365	yhgE	DUF4153 family putative inner membrane protein	0.2028	0.0739
JW3366	pck	phosphoenolpyruvate carboxykinase [ATP]	-0.0516	0.0631
JW3367	envZ	sensory histidine kinase in two-component regulatory system with OmpR	0.1520	0.0004
JW3368	ompR	response regulator in two-component regulatory system with EnvZ	-0.0032	0.8792
JW3369	greB	transcript cleavage factor	-0.1410	0.0000
JW3370	yhgF	putative transcriptional accessory factor; ionizing radiation survival protein; putative nucleic acid-binding protein	-0.0367	0.3271
JW3371	feoA	ferrous iron transporter, protein A	0.0050	0.8936
JW3372	feoB	ferrous iron transporter protein B and GTP-binding protein; membrane protein	-0.1543	0.0000
JW3373	yhgG	putative DNA-binding transcriptional regulator	-0.0378	0.0343
JW3374	yhgA	transposase_31 family protein	0.0051	0.9243
JW3375	bioH	pimeloyl-ACP methyl ester carboxylesterase	0.0964	0.0026
JW3377	gntY	Fe/S biogenesis protein, scaffold/chaperone protein	0.0584	0.0158
JW3379	malQ	4-alpha-glucanotransferase (amylomaltase)	-0.0138	0.2841
JW3381	malT	mal regulon transcriptional activator	0.0064	0.6555
JW3384	rtcB	RNA-splicing ligase	0.0205	0.6653
JW3385	rtcR	sigma 54-dependent transcriptional regulator of rtcBA expression	0.0228	0.1435
JW3386	glpR	pseudogene, DNA-binding transcriptional repressor	-0.0421	0.0411

JW3388	glpE	thiosulfate:cyanide sulfurtransferase (rhodanese)	0.0238	0.1450
JW3389	glpD	sn-glycerol-3-phosphate dehydrogenase, aerobic, FAD/NAD(P)-binding	0.0258	0.3468
JW3390	yzgL	pseudogene, periplasmic solute binding protein homology	0.3086	0.0165
JW3391	glgP	glycogen phosphorylase	-0.0362	0.1943
JW3392	glgA	glycogen synthase	0.0919	0.0057
JW3393	glgC	glucose-1-phosphate adenylyltransferase	0.0194	0.3506
JW3394	glgX	glycogen debranching enzyme	0.0081	0.4916
JW3395	glgB	1,4-alpha-glucan branching enzyme	-0.0730	0.2782
JW3397	yhgN	UPF0056 family inner membrane protein	-0.0496	0.0294
JW3400	gntK	gluconate kinase 2	0.0187	0.2827
JW3402	yhhW	quercetinase activity in vitro	-0.0294	0.0165
JW3403	yhhX	putative oxidoreductase	-0.0208	0.0886
JW3405	yhhY	L-amino acid N-acetyltransferase; aminoacyl nucleotide detoxifying acetyltransferase	0.0081	0.7196
JW3406	yhhZ	putative Hcp1 family polymorphic toxin protein; putative colicin-like DNase/tRNase activity	0.0032	0.9526
JW3411	yrhB	stable heat shock chaperone	0.1659	0.0088
JW3412	ggt	gamma-glutamyltranspeptidase	0.1500	0.0072
JW3413	yhhA	DUF2756 family protein	-0.3402	0.0568
JW3414	ugpQ	glycerophosphodiester phosphodiesterase, cytosolic	-0.0085	0.7310
JW3415	ugpC	sn-glycerol-3-phosphate ABC transporter ATPase	0.1518	0.2018
JW3416	ugpE	sn-glycerol-3-phosphate ABC transporter permease	-0.0238	0.4020
JW3417	ugpA	sn-glycerol-3-phosphate ABC transporter permease	-0.0792	0.0007
JW3419	livF	branched-chain amino acid ABC transporter ATPase	-0.1603	0.1260
JW3420	livG	branched-chain amino acid ABC transporter ATPase	-0.0278	0.3042
JW3421	livM	branched-chain amino acid ABC transporter permease	0.0462	0.1024
JW3422	livH	branched-chain amino acid ABC transporter permease	0.0002	0.9949
JW3423	livK	leucine transporter subunit	0.0056	0.9496
JW3424	yhhK	PanD autocleavage accelerator, panothenate synthesis	0.0169	0.7644
JW3425	livJ	branched-chain amino acid ABC transporter periplasmic binding protein	-0.0347	0.1344
JW3432	yhhM	DUF2500 family protein	-0.0193	0.5477
JW3433	yhhN	TMEM86 family putative inner membrane protein	-0.0048	0.7415
JW3434	zntA	zinc, cobalt and lead efflux system	0.0294	0.5033
JW3435	yhhP	mnm(5)-s(2)U34-tRNA 2-thiolation sulfurtransferase	-0.0760	0.0412

JW3440	acpT	4'-phosphopantetheinyl transferase	0.0648	0.0180
JW3441	nikA	nickel/heme ABC transporter periplasmic binding protein	0.0440	0.4743
JW3442	nikB	nickel ABC transporter permease	0.0324	0.3716
JW3443	nikC	nickel ABC transporter permease	0.1073	0.0049
JW3444	nikD	nickel ABC transporter ATPase	-0.0776	0.0000
JW3445	nikE	nickel ABC transporter ATPase	0.1268	0.0056
JW3446	nikR	transcriptional repressor, Ni-binding	0.0466	0.0355
JW3449	yhhH	putative NTF2 fold immunity protein for polymorphic toxin RhsB	-0.0594	0.0076
JW3451	yhhI	putative transposase	-0.0401	0.1377
JW3454	yhiI	putative membrane fusion protein (MFP) of efflux pump	0.0341	0.1155
JW3455	yhiJ	DUF4049 family protein	-0.0126	0.7193
JW3457	yhiL	Putative uncharacterized protein	0.0631	0.2953
JW3459	yhiN	putative oxidoreductase	-0.0106	0.7872
JW3460	pitA	phosphate transporter, low-affinity; tellurite importer	0.0908	0.0064
JW3461	yhiO	universal stress (ethanol tolerance) protein B	-0.1400	0.0677
JW3462	uspA	universal stress global response regulator	-0.0417	0.3129
JW3463	yhiP	dipeptide and tripeptide permease B	-0.3839	0.0137
JW3465	prlC	oligopeptidase A	-0.0361	0.5068
JW3466	yhiR	23S rRNA m(6)A2030 methyltransferase, SAM-dependent	0.0226	0.3623
JW3467	gor	glutathione oxidoreductase	0.0501	0.0013
JW3468	arsR	arsenical resistance operon transcriptional repressor; autorepressor	-0.0105	0.8502
JW3469	arsB	arsenite/antimonite transporter	0.0840	0.0052
JW3470	arsC	arsenate reductase	-0.2181	0.1456
JW3471	yhiS	pseudogene	0.0279	0.3511
JW3474	slp	outer membrane lipoprotein	-0.0127	0.5162
JW3475	yhiF	Putative LuxR family repressor for dicarboxylate transport	-0.0522	0.3517
JW3478	hdeA	stress response protein acid-resistance protein	0.1407	0.0004
JW3479	hdeD	acid-resistance membrane protein	0.0160	0.3589
JW3480	gadE	gad regulon transcriptional activator	0.1689	0.0039
JW3481	mdtE	anaerobic multidrug efflux transporter, ArcA-regulated	0.0789	0.0175
JW3482	mdtF	anaerobic multidrug efflux transporter, ArcA-regulated	0.0809	0.0002
JW3483	gadW	transcriptional activator of gadA and gadBC; repressor of gadX	-0.1016	0.3064
JW3484	gadX	acid resistance regulon transcriptional activator; autoactivator	0.0035	0.8822
JW3485	gadA	glutamate decarboxylase A, PLP-dependent	0.1795	0.0080
JW3486	yhjA	cytochrome c peroxidase	-0.0780	0.2853
JW3487	treF	cytoplasmic trehalase	0.0299	0.1809

JW3488	yhjB	putative DNA-binding transcriptional response regulator	0.4025	0.0073
JW3489	yhjC	LysR family putative transcriptional regulator	0.1498	0.0001
JW3490	yhjD	inner membrane putative BrbK family alternate lipid exporter	-0.2518	0.0001
JW3491	yhjE	putative MFS transporter; membrane protein	0.0034	0.8883
JW3492	yhjG	putative inner membrane-anchored periplasmic AsmA family protein	0.0391	0.0993
JW3493	yhjH	cyclic-di-GMP phosphodiesterase, FlhDC-regulated	-0.0607	0.0466
JW3495	yhjJ	putative periplasmic M16 family chaperone	-0.0326	0.5492
JW3496	dctA	C4-dicarboxylic acid, orotate and citrate transporter	0.0950	0.0008
JW3499	bcsZ	endo-1,4-D-glucanase	-0.0977	0.0172
JW3503	yhjR	DUF2629 family protein	0.0027	0.9300
JW3504	bcsE	cellulose production protein	-0.0368	0.1930
JW3506	bcsG	DUF3260 family cellulose production inner membrane protein	0.0306	0.1059
JW3508	yhjV	putative transporter	0.0643	0.1556
JW3509	dppF	dipeptide/heme ABC transporter ATPas	0.1689	0.0040
JW3510	dppD	dipeptide/heme ABC transporter ATPas	0.0785	0.0656
JW3512	dppB	dipeptide/heme ABC transporter permease	-0.0199	0.5028
JW3513	dppA	dipeptide/heme ABC transporter periplasmic binding protein; dipeptide chemotaxis receptor	-0.0309	0.3577
JW3516	yhjX	putative MFS antiporter, pyruvate-inducible	-0.0094	0.7583
JW3518	tag	3-methyl-adenine DNA glycosylase I, constitutive	0.1103	0.0097
JW3519	yiaC	GNAT family putative N-acetyltransferase	-0.0378	0.2641
JW3524	yiaG	HTH_CROC1 family putative transcriptional regulator	-0.0484	0.1550
JW3525	cspA	RNA chaperone and antiterminator, cold-inducible	-0.0459	0.0946
JW3526	hokA	toxic polypeptide, small	-0.0917	0.0150
JW3530	glyS	glycine tRNA synthetase, beta subunit	-0.0617	0.0052
JW3532	ysaB	uncharacterized protein	0.0747	0.0306
JW3533	yiaH	O-acetyltransferase for enterobacterial common antigen (ECA)	0.0371	0.0852
JW3534	yiaA	YiaAB family inner membrane protein, tandem domains	0.0027	0.9126
JW3536	xylB	xylulokinase	-0.0219	0.0667
JW3537	xylA	D-xylose isomerase	0.0122	0.7112
JW3538	xylF	D-xylose transporter subunit	0.0711	0.0991
JW3539	xylG	D-xylose ABC transporter dual domain ATPase	-0.0512	0.0800

JW3540	xylH	D-xylose ABC transporter permease	-0.0647	0.0081
JW3541	xylR	xylose divergent operon transcriptional activator	0.0050	0.7927
JW3543	malS	alpha-amylase	-0.0835	0.0062
JW3545	yiaI	putative hydrogenase, 4Fe-4S ferredoxin-type component	-0.0385	0.1536
JW3546	yiaJ	transcriptional repressor for the yiaKLMNO-lyxK-sgbHUE operon	0.0445	0.0057
JW3547	yiaK	2,3-diketo-L-gulonate reductase, NADH-dependent	0.0199	0.2643
JW3548	yiaL	DUF386 family protein	0.0838	0.0363
JW3549	yiaM	2,3-diketo-L-gulonate TRAP transporter small permease protein	-0.0371	0.2331
JW3551	yiaO	2,3-diketo-L-gulonate-binding periplasmic protein	0.1448	0.0871
JW3552	lyxK	L-xylulose kinase	0.0035	0.8963
JW3553	sgbH	3-keto-L-gulonate 6-phosphate decarboxylase	-0.0041	0.8960
JW3555	sgbE	L-ribulose-5-phosphate 4-epimerase	-0.1223	0.0001
JW3556	yiaT	putative outer membrane protein	0.0108	0.6698
JW3557	yiaU	putative DNA-binding transcriptional regulator	0.0020	0.9102
JW3558	yiaV	signal-anchored membrane fusion protein (MFP) component of efflux pump	-0.0097	0.8345
JW3559	yiaW	DUF3302 family inner membrane protein	0.0360	0.2693
JW3561	aldB	aldehyde dehydrogenase B	0.0664	0.0842
JW3563	selB	selenocysteinyl-tRNA-specific translation factor	0.0149	0.6598
JW3564	selA	selenocysteine synthase	0.0521	0.0859
JW3565	yibF	glutathione S-transferase homolog	-0.0079	0.8425
JW3566	rhsA	Rhs protein with putative toxin 55 domain; putative polysaccharide synthesis/export protein; putative neighboring cell growth inhibitor	-0.0919	0.4179
JW3568	yibA	putative immunity protein for polymorphic toxin RhsA; HEAT-domain protein; lethality reduction protein	-0.0701	0.0930
JW3570	yibG	TPR-like repeat protein	0.0384	0.0228
JW3571	yibH	putative membrane fusion protein (MFP) component of efflux pump	-0.0605	0.0054
JW3572	yibI	DUF3302 family inner membrane protein	-0.0422	0.3337
JW3573	mtlA	mannitol-specific PTS enzyme: IIA, IIB and IIC components	0.0600	0.1415
JW3574	mtlD	mannitol-1-phosphate dehydrogenase, NAD-dependent	-0.0268	0.1240
JW3575	mtlR	mannitol operon repressor	-0.0398	0.6727
JW3576	yibT	uncharacterized protein	0.0649	0.0649

JW3577	yibL	ribosome-associated DUF2810 family protein	-0.0286	0.3015
JW3578	lldP	L-lactate permease	0.0119	0.9014
JW3579	lldR	dual role activator/repressor for lldPRD operon	0.0056	0.7601
JW3580	lldD	L-lactate dehydrogenase, FMN-linked	0.0314	0.0519
JW3581	yibK	tRNA Leu mC34,mU34 2'-O-methyltransferase, SAM-dependent	0.1725	0.0000
JW3582	cysE	serine acetyltransferase	0.0153	0.5430
JW3584	secB	protein export chaperone	0.0452	0.1677
JW3585	grxC	glutaredoxin 3	-0.0506	0.0232
JW3586	yibN	putative rhodanese-related sulfurtransferase	-0.3068	0.0002
JW3587	gpmI	phosphoglycero mutase III, cofactor-independent	0.1176	0.0061
JW3590	yibD	LPS(HepIII)-glucuronic acid glycosyltransferase	0.0635	0.0097
JW3591	tdh	L-threonine 3-dehydrogenase, NAD(P)-binding	-0.0262	0.7017
JW3592	kbl	glycine C-acetyltransferase	0.0625	0.1630
JW3594	rfaD	ADP-L-glycero-D-mannoheptose-6-epimerase, NAD(P)-binding	-0.4487	0.0001
JW3596	rfaC	ADP-heptose:LPS heptosyl transferase I	-0.1844	0.1457
JW3597	rfaL	O-antigen ligase	-0.1161	0.0028
JW3599	rfaZ	lipopolysaccharide KdoIII transferase; lipopolysaccharide core biosynthesis protein	-0.0179	0.5321
JW3600	rfaY	lipopolysaccharide core biosynthesis protein	0.1275	0.0006
JW3601	rfaJ	lipopolysaccharide 1,2-glucosyltransferase; UDP-glucose:(glucosyl)LPS alpha-1,2-glucosyltransferase	0.0888	0.0527
JW3602	rfaI	UDP-D-galactose:(glucosyl)lipopolysaccharide-alpha-1,3-D-galactosyltransferase	-0.1378	0.0005
JW3603	rfaB	lipopolysaccharide 1,6-galactosyltransferase; UDP-D-galactose:(glucosyl)lipopolysaccharide-1,6-D-galactosyltransferase	0.0153	0.6389
JW3604	rfaS	lipopolysaccharide rhamnose:KdoIII transferase; lipopolysaccharide core biosynthesis protein	0.0409	0.1275
JW3605	rfaP	kinase that phosphorylates core heptose of lipopolysaccharide	0.0326	0.6467
JW3606	rfaG	UDP-glucose:(heptosyl)lipopolysaccharide alpha-1,3-glucosyltransferase; lipopolysaccharide core biosynthesis protein; lipopolysaccharide glucosyltransferase I	-0.6413	0.0000
JW3610	mutM	formamidopyrimidine/5-formyluracil/ 5-hydroxymethyluracil DNA glycosylase	0.0080	0.6714
JW3611	rpmG	50S ribosomal subunit protein L33	-0.0690	0.1171
JW3617	pyrE	orotate phosphoribosyltransferase	0.0657	0.4236

JW3618	rph	ribonuclease PH (defective)	-0.0286	0.1049
JW3619	yicC	UPF0701 family protein	-0.1729	0.0002
JW3620	dinD	DNA damage-inducible protein	-0.0389	0.1716
JW3621	yicG	UPF0126 family inner membrane protein	-0.0761	0.1274
JW3622	ligB	DNA ligase, NAD(+)-dependent	-0.0191	0.3064
JW3624	rpoZ	RNA polymerase, omega subunit	-0.0726	0.0036
JW3626	trmH	tRNA mG18-2'-O-methyltransferase, SAM-dependent	-0.0670	0.0821
JW3627	recG	ATP-dependent DNA helicase	-0.1451	0.0094
JW3628	gltS	glutamate transporter	-0.0085	0.6682
JW3629	yicE	xanthine permease	-0.0228	0.5683
JW3630	yicH	putative inner membrane-anchored periplasmic AsmA family protein	-0.0080	0.6994
JW3631	yicI	putative alpha-glucosidase	0.0638	0.0490
JW3633	setC	putative arabinose efflux transporter	0.0564	0.1218
JW3634	yicL	EamA family inner membrane putative transporter	-0.0114	0.5995
JW3635	nlpA	cytoplasmic membrane lipoprotein-28	-0.1043	0.0000
JW3640	ade	cryptic adenine deaminase	-0.0367	0.4539
JW3641	uhpT	hexose phosphate transporter	-0.0079	0.8709
JW3642	uhpC	membrane protein regulates uhpT expression	-0.0555	0.1683
JW3643	uhpB	sensory histidine kinase in two-component regulatory sytem with UhpA	0.0009	0.9504
JW3644	uhpA	response regulator in two-component regulatory system wtih UhpB	0.0492	0.0623
JW3645	ilvN	acetolactate synthase 1 small subunit	-0.0173	0.7538
JW3646	ilvB	acetolactate synthase 2 large subunit	-0.1984	0.1469
JW3646	ilvB	acetolactate synthase 2 large subunit	0.0852	0.2961
JW3647	ivbL	ilvB operon leader peptide	-0.0447	0.0566
JW3650	yidF	putative Cys-type oxidative YidJ-maturating enzyme	0.1074	0.0043
JW3651	yidG	inner membrane protein	-0.0956	0.0034
JW3652	yidH	DUF202 family inner membrane protein	0.0042	0.7241
JW3653	yidI	inner membrane protein	-0.1679	0.0856
JW3654	yidJ	sulfatase/phosphatase superfamily protein	-0.0675	0.0369
JW3655	yidK	putative SSF family symporter	0.0510	0.0316
JW3656	yidL	AraC family putative transcriptional regulator	0.0283	0.7603
JW3658	glvG	pseudogene	0.0650	0.1535
JW3659	glvB	pseudogene, arbutin specific enzyme IIC component of PTS	-0.1737	0.0007
JW3660	glvC	Putative permease IIC component	-0.0451	0.0362

JW3661	yidP	UTRA domain-containing GntR family putative transcriptional regulator	-0.0443	0.3691
JW3662	yidE	putative transporter	-0.0107	0.6227
JW3663	ibpB	heat shock chaperone	-0.0679	0.0364
JW3664	ibpA	heat shock chaperone	-0.0615	0.0298
JW3670	dgoK	2-oxo-3-deoxygalactonate kinase	0.0209	0.4954
JW3674	yidA	sugar phosphate phosphatase	-0.4830	0.0016
JW3675	yidB	DUF937 family protein	-0.0562	0.2145
JW3677	recF	gap repair protein	-0.0133	0.7870
JW3682	yidD	membrane protein insertion efficiency factor, UPF0161 family inner membrane protein	-0.0527	0.0391
JW3684	trmE	tRNA U34 5-methylaminomethyl-2-thiouridine modification GTPase	0.0783	0.0486
JW3685	tnaC	tryptophanase leader peptide	-0.0717	0.1092
JW3686	tnaA	tryptophanase/L-cysteine desulfhydrase, PLP-dependent	-0.0098	0.6594
JW3688	mdtL	multidrug efflux system protein	-0.0702	0.2128
JW3689	yidZ	putative DNA-binding transcriptional regulator	-0.0384	0.2042
JW3690	yieE	phosphopantetheinyl transferase superfamily protein	-0.0112	0.6139
JW3691	yieF	chromate reductase, Class I, flavoprotein	0.0231	0.4132
JW3692	yieG	adenine permease, high affinity; adenine:H+ symporter	0.0087	0.8698
JW3693	yieH	phosphoenolpyruvate and 6-phosphogluconate phosphatase	-0.0418	0.1886
JW3694	yieI	PRK09823 family inner membrane protein, creBC regulon	-0.0578	0.0589
JW3698	bglH	carbohydrate-specific outer membrane porin, cryptic	-0.0035	0.8681
JW3699	bglB	cryptic phospho-beta-glucosidase B	0.2995	0.0030
JW3700	bglF	fused beta-glucoside-specific PTS enzymes: IIA component/IIB component/IIC component	-0.0492	0.0751
JW3701	bglG	transcriptional antiterminator of the bgl operon	0.0073	0.7075
JW3702	phoU	negative regulator of PhoR/PhoB two-component regulator	-0.0093	0.6000
JW3703	pstB	phosphate ABC transporter ATPase	0.0074	0.8225
JW3704	pstA	phosphate ABC transporter permease	-0.0128	0.7848
JW3705	pstC	phosphate ABC transporter permease	0.0703	0.3591
JW3706	pstS	phosphate ABC transporter periplasmic binding protein	-0.1685	0.0632
JW3709	atpC	F1 sector of membrane-bound ATP synthase, epsilon subunit	0.0576	0.3321
JW3710	atpD	F1 sector of membrane-bound ATP synthase, beta subunit	-0.1362	0.0264
JW3711	atpG	F1 sector of membrane-bound ATP synthase, gamma subunit	-0.0575	0.0844
JW3712	atpA	F1 sector of membrane-bound ATP synthase, alpha subunit	0.0267	0.2405

JW3713	atpH	F1 sector of membrane-bound ATP synthase, delta subunit	-0.0411	0.6908
JW3714	atpF	F0 sector of membrane-bound ATP synthase, subunit b	-0.2296	0.0131
JW3715	atpE	F0 sector of membrane-bound ATP synthase, subunit c	-0.2295	0.0122
JW3716	atpB	F0 sector of membrane-bound ATP synthase, subunit a	-0.1718	0.0027
JW3718	gidB	16S rRNA m(7)G527 methyltransferase, SAM-dependent; glucose-inhibited cell-division protein	0.0661	0.0012
JW3719	gidA	5-methylaminomethyl-2-thiouridine modification at tRNA U34	0.0487	0.0866
JW3720	mioC	FMN-binding protein MioC	-0.0132	0.4293
JW3721	asnC	transcriptional activator of asnA; autorepressor	-0.0155	0.5516
JW3722	asnA	asparagine synthetase A	-0.0073	0.7750
JW3725	yieN	hexameric AAA+ MoxR family ATPase, putative molecular chaperone	0.0266	0.0100
JW3728	rbsA	D-ribose ABC transporter ATPase	-0.0400	0.0743
JW3729	rbsC	D-ribose ABC transporter permease	0.0344	0.4020
JW3730	rbsB	D-ribose ABC transporter periplasmic binding protein; ribose chemotaxis receptor	-0.0082	0.6730
JW3731	rbsK	ribokinase	-0.0601	0.0013
JW3732	rbsR	transcriptional repressor of ribose metabolism	-0.0899	0.0011
JW3733	hsrA	putative multidrug or homocysteine efflux system	-0.1365	0.0942
JW3737	yifE	UPF0438 family protein	-0.0573	0.4672
JW3738	yifB	magnesium chelatase family protein and putative transcriptional regulator	-0.0059	0.8526
JW3739	ilvL	ilvG operon leader peptide	0.0295	0.2400
JW3741	ilvG	Acetolactate synthase isozyme 2 large subunit	-0.1615	0.0000
JW3742	ilvM	acetolactate synthase 2 small subunit	-0.0099	0.7133
JW3745	ilvA	l-threonine dehydratase, biosynthetic; also known as threonine deaminase	0.1629	0.1567
JW3746	ilvY	transcriptional activator of ilvC; autorepressor	0.0646	0.4231
JW3747	ilvC	ketol-acid reductoisomerase, NAD(P)-binding	0.0026	0.9170
JW3748	ppiC	peptidyl-prolyl cis-trans isomerase C (rotamase C)	-0.0083	0.7812
JW3749	yifO	PemK toxin family pseudogene	-0.0945	0.0003
JW3750	yifN	Putative uncharacterized protein	-0.0456	0.3952
JW3753	rhlB	ATP-dependent RNA helicase	-0.0271	0.5588
JW3755	rhoL	Putative rho operon leader peptide	-0.2994	0.0006
JW3756	rho	transcription termination factor	0.0571	0.3580

JW3758	rfe	UDP-GlcNAc:undecaprenylphosphate GlcNAc-1-phosphate transferase	-0.2724	0.0104
JW3763	rffH	glucose-1-phosphate thymidylyltransferase	0.0389	0.4653
JW3766	wzxE	O-antigen translocase	0.0750	0.0283
JW3770	rffM	UDP-N-acetyl-D-mannosaminuronic acid transferase	-0.0021	0.9569
JW3773	aslA	putative Ser-type periplasmic non-aryl sulfatase	-0.0558	0.0951
JW3774	hemY	putative protoheme IX synthesis protein	0.0237	0.2910
JW3775	hemX	putative uroporphyrinogen III methyltransferase	-0.0430	0.4150
JW3778	cyaA	adenylate cyclase	0.0304	0.1221
JW3779	cyaY	iron-dependent inhibitor of iron-sulfur cluster formation; frataxin; iron-binding and oxidizing protein	-0.2253	0.1077
JW3780	yzcX	Putative uncharacterized protein b3808	-0.1280	0.0204
JW3781	yifL	putative lipoprotein	-0.0296	0.2550
JW3783	yigA	DUF484 family protein	0.0257	0.3327
JW3784	xerC	site-specific tyrosine recombinase	0.0084	0.8077
JW3786	uvrD	DNA-dependent ATPase I and helicase II	0.0137	0.6364
JW3789	corA	magnesium/nickel/cobalt transporter	-0.0383	0.4289
JW3794	pldA	outer membrane phospholipase A	-0.0265	0.1580
JW3803	yigM	putative inner membrane EamA-like transporter	0.0580	0.3286
JW3804	metR	methionine biosynthesis regulon transcriptional regulator	-0.0007	0.9801
JW3805	metE	5-methyltetrahydropteroyltriglutamate-homocysteine S-methyltransferase	-0.1560	0.0438
JW3808	udp	uridine phosphorylase	-0.0070	0.8751
JW3809	rmuC	DNA recombination protein	-0.1448	0.1469
JW3813	tatA	TatABCE protein translocation system subunit	0.1347	0.0839
JW3815	tatC	TatABCE protein translocation system subunit	-0.0347	0.4081
JW3818	rfaH	transcription antitermination protein	-0.2129	0.0072
JW3820	fre	NAD(P)H-flavin reductase	0.1218	0.0076
JW3822	fadB	fused 3-hydroxybutyryl-CoA epimerase/delta(3)-cis-delta(2)-trans-enoyl-CoA isomerase/enoyl-CoA hydratase/3-hydroxyacyl-CoA dehydrogenase	0.0083	0.7800
JW3823	pepQ	proline dipeptidase	-0.1424	0.0007
JW3829	mobA	molybdopterin-guanine dinucleotide synthase	-0.3214	0.0108
JW3830	yihD	DUF1040 protein YihD	0.1298	0.0005
JW3831	yihE	Cpx stress response Thr/Ser protein kinase; MazF antagonist protein	0.0040	0.8546
JW3834	yihG	inner membrane protein, inner membrane acyltransferase	-0.0361	0.1908

JW3835	polA	5' to 3' DNA polymerase and 3' to 5'/5' to 3' exonuclease	-0.1283	0.1608
JW3837	yihI	activator of Der GTPase	0.0349	0.0923
JW3838	hemN	coproporphyrinogen III oxidase, SAM and NAD(P)H dependent, oxygen-independent	-0.0987	0.0481
JW3839	glnG	fused DNA-binding response regulator in two-component regulatory system with GlnL: response regulator/sigma54 interaction protein	0.0138	0.3878
JW3840	glnL	sensory histidine kinase in two-component regulatory system with GlnG	-0.1649	0.0076
JW3841	glnA	glutamine synthetase	0.1034	0.0343
JW3843	yihL	putative DNA-binding transcriptional regulator	-0.2495	0.0572
JW3844	yihM	putative sugar phosphate isomerase	-0.0214	0.5425
JW3845	yihN	MFS transporter family protein	0.0100	0.6825
JW3846	ompL	outer membrane porin L; putative sulpholipid porin	0.0768	0.0121
JW3848	yihP	putative 2,3-dihydroxypropane-1-sulphonate exporter, membrane protein	0.0202	0.3694
JW3849	yihQ	putative sulpholipid alpha-glucosidase; alpha-glucosyl fluoride glucosidase	0.0026	0.9160
JW3850	yihR	putative sulphoquinovose mutarotase	-0.0726	0.0021
JW3852	yihT	6-deoxy-6-sulphofructose-1-phosphate aldolase	-0.0214	0.4496
JW3853	yihU	3-sulpholactaldehyde (SLA) reductase, NADH-dependent; gamma-hydroxybutyrate dehydrogenase, NADH-dependent	-0.1102	0.3913
JW3857	rbn	BrkB family putative transporter, inner membrane protein	0.0367	0.1902
JW3858	dtd	D-tyr-tRNA(Tyr) deacylase	0.0217	0.4251
JW3859	yiiD	GNAT family putative N-acetyltransferase	0.0352	0.0196
JW3862	fdhE	formate dehydrogenase formation protein	-0.0253	0.2875
JW3863	fdoI	formate dehydrogenase-O, cytochrome b556 subunit	-0.0510	0.0177
JW3864	fdoH	formate dehydrogenase-O, Fe-S subunit	-0.0641	0.3615
JW3865	fdoG	formate dehydrogenase-O, large subunit	0.0189	0.4264
JW3866	fdhD	formate dehydrogenase formation protein	-0.0211	0.6401
JW3867	yiiG	DUF3829 family lipoprotein	-0.0299	0.2531

JW3868	frvR	putative frv operon regulator; contains a PTS EIIA domain	0.1079	0.0009
JW3869	frvX	putative peptidase	0.0920	0.0138
JW3872	yiiL	L-rhamnose mutarotase	-0.1059	0.4247
JW3876	rhaS	transcriptional activator of rhaBAD and rhaT	-0.1041	0.0084
JW3877	rhaR	transcriptional activator of rhaSR	0.0068	0.8273
JW3878	rhaT	L-rhamnose:proton symporter	0.0126	0.7506
JW3879	sodA	superoxide dismutase, Mn	0.1590	0.0000
JW3882	cpxA	sensory histidine kinase in two-component regulatory system with CpxR	-0.0050	0.7748
JW3883	cpxR	response regulator in two-component regulatory system with CpxA	-0.0319	0.0668
JW3886	fieF	ferrous iron and zinc transporter	0.0356	0.2591
JW3887	pfkA	6-phosphofructokinase I	-0.0066	0.9259
JW3888	sbp	sulfate transporter subunit	0.0603	0.0623
JW3889	cdh	CDP-diacylglycerol phosphotidylhydrolase	0.0464	0.2100
JW3890	tpiA	triosephosphate isomerase	-0.0105	0.5825
JW3891	yiiQ	DUF1454 family putative periplasmic protein	-0.0511	0.2446
JW3892	yiiR	DUF805 family putative inner membrane protein	0.0264	0.1518
JW3893	yiiS	UPF0381 family protein	0.0134	0.7156
JW3894	yiiT	stress-induced protein	-0.0500	0.3747
JW3895	fpr	ferredoxin-NADP reductase; flavodoxin reductase	-0.0132	0.5741
JW3896	glpX	fructose 1,6-bisphosphatase II	0.1074	0.0005
JW3897	glpK	glycerol kinase	0.0217	0.3347
JW3898	glpF	glycerol facilitator	0.0330	0.6058
JW3899	yiiU	FtsZ stabilizer, septal ring assembly factor, cell division stimulator	-0.0295	0.3164
JW3900	rraA	ribonuclease E (RNase E) inhibitor protein	0.0557	0.1763
JW3901	menA	1,4-dihydroxy-2-naphthoate octaprenyltransferase	0.1463	0.0383
JW3902	hslU	molecular chaperone and ATPase component of HslUV protease	-0.1315	0.1124
JW3903	hslV	peptidase component of the HslUV protease	0.1346	0.0965
JW3905	cytR	Anti-activator for CytR-CRP nucleoside utilization regulon	-0.0330	0.3792
JW3906	priA	Primosome factor n' (replication factor Y)	-0.1203	0.2563
JW3907	rpmE	50S ribosomal subunit protein L31	-0.0300	0.3927
JW3908	yiiX	putative lipid binding hydrolase, DUF830 family protein	0.0431	0.3067
JW3909	metJ	transcriptional repressor, S-adenosylmethionine-binding	0.0199	0.3648
JW3910	metB	cystathionine gamma-synthase, PLP-dependent	0.1454	0.0335
JW3911	metL	Bifunctional aspartokinase/homoserine dehydrogenase 2	0.2835	0.0001

JW3913	metF	5,10-methylenetetrahydrofolate reductase	0.1244	0.0989
JW3914	katG	catalase-peroxidase HPI, heme b-containing	0.1740	0.0441
JW3916	yijF	DUF1287 family protein	0.1008	0.0605
JW3918	fsaB	fructose-6-phosphate aldolase 2	-0.0462	0.0892
JW3921	frwC	putative enzyme IIC component of PTS	0.0681	0.3218
JW3922	frwB	putative enzyme IIB component of PTS	0.0417	0.2506
JW3923	pflD	putative glycine radical domain-containing pyruvate formate-lyase	-0.0316	0.7403
JW3924	pflC	putative [formate-C-acetyltransferase 2]-activating enzyme; pyruvate formate-lyase 1-activating enzyme	0.1449	0.0414
JW3925	frwD	putative enzyme IIB component of PTS	0.0683	0.0169
JW3926	yijO	AraC family putative transcriptional activator	0.0747	0.0074
JW3927	yijP	LPS heptose I phosphoethanolamine transferase	-0.0492	0.0841
JW3928	ppc	phosphoenolpyruvate carboxylase	0.0714	0.2908
JW3929	argE	acetylornithine deacetylase	0.1514	0.0182
JW3929	argE	acetylornithine deacetylase	0.2009	0.0979
JW3930	argC	N-acetyl-gamma-glutamylphosphate reductase, NAD(P)-binding	-0.0712	0.0300
JW3932	argH	argininosuccinate lyase	0.2251	0.0738
JW3933	oxyR	oxidative and nitrosative stress transcriptional regulator	-0.0213	0.2811
JW3935	fabR	transcriptional repressor of fabA and fabB	-0.0230	0.2840
JW3936	yijD	DUF1422 family inner membrane protein	0.0623	0.0303
JW3937	trmA	tRNA m(5)U54 methyltransferase, SAM-dependent; tmRNA m(5)U341 methyltransferase	-0.0141	0.7504
JW3938	btuB	vitamin B12/cobalamin outer membrane transporter	0.1766	0.0032
JW3942	coaA	pantothenate kinase	-0.0751	0.0953
JW3943	tufB	translation elongation factor EF-Tu 2	-0.1795	0.0167
JW3946	rplK	50S ribosomal subunit protein L11	-0.1410	0.0005
JW3947	rplA	50S ribosomal subunit protein L1	0.0430	0.0967
JW3952	htrC	stationary phase growth adaptation protein	0.0052	0.8347
JW3953	thiH	tyrosine lyase, involved in thiamine-thiazole moiety synthesis	-0.0043	0.9173
JW3955	thiS	immediate sulfur donor in thiazole formation	0.2002	0.0002
JW3956	thiF	adenylyltransferase, modifies ThiS C-terminus	0.1205	0.0159
JW3957	thiE	thiamine phosphate synthase (thiamine phosphate pyrophosphorylase)	0.0970	0.0539
JW3958	thiC	phosphomethylpyrimidine synthase	0.0992	0.0718

JW3959	rsd	stationary phase protein, binds sigma 70 RNA polymerase subunit	0.0364	0.1967
JW3961	hemE	uroporphyrinogen decarboxylase	0.0146	0.4571
JW3963	yjaG	DUF416 domain protein	0.0676	0.0522
JW3964	hupA	HU, DNA-binding transcriptional regulator, alpha subunit	-0.0523	0.0143
JW3965	yjaH	DUF1481 family putative lipoprotein	-0.1815	0.1789
JW3967	zraS	sensory histidine kinase in two-component regulatory system with ZraR	0.0384	0.4628
JW3968	zraR	fused DNA-binding response regulator in two-component regulatory system with ZraS: response regulator/sigma54 interaction protein	0.0470	0.0732
JW3969	purD	phosphoribosylglycinamide synthetase phosphoribosylamine-glycine ligase	0.1248	0.0216
JW3970	purH	IMP cyclohydrolase and phosphoribosylaminoimidazolecarboxamide formyltransferase	0.0317	0.4788
JW3971	yjaA	stress-induced protein	0.0208	0.5092
JW3972	yjaB	GNAT-family putative N-acetyltransferase; acetyl coenzyme A-binding protein	0.1350	0.0221
JW3973	metA	homoserine O-transsuccinylase	0.0088	0.8970
JW3974	aceB	malate synthase A	-0.0034	0.9377
JW3975	aceA	isocitrate lyase	0.1521	0.0457
JW3976	aceK	isocitrate dehydrogenase kinase/phosphatase	0.0409	0.0221
JW3977	arpA	ankyrin repeat protein	0.0005	0.9898
JW3978	iclR	transcriptional repressor	-0.0184	0.3766
JW3979	metH	homocysteine-N5-methyltetrahydrofolate transmethylase, B12-dependent	-0.0291	0.5668
JW3980	yjbB	putative Na+/Pi-cotransporter	0.1183	0.0011
JW3981	pepE	peptidase E, alpha-aspartyl dipeptidase	-0.0381	0.3159
JW3982	yjbC	23S rRNA pseudouridine(2604) synthase	0.2157	0.0058
JW3983	yjbD	DUF3811 family protein	0.1185	0.0006
JW3984	lysC	lysine-sensitive aspartokinase 3	-0.3475	0.0006
JW3985	pgi	glucosephosphate isomerase	0.0459	0.0930
JW3986	yjbE	extracellular polysaccharide production threonine-rich protein	0.0439	0.1666
JW3988	yjbG	extracellular polysaccharide export OMA protein	0.0997	0.0680
JW3989	yjbH	DUF940 family extracellular polysaccharide protein	-0.0079	0.5611
JW3990	yjbA	phosphate starvation inducible protein	0.1146	0.0017

JW3991	xylE	D-xylose transporter	0.0330	0.4698
JW3992	malG	maltose transporter subunit	-0.0001	0.9990
JW3993	malF	maltose transporter subunit	0.0241	0.4976
JW3994	malE	maltose transporter subunit	0.2064	0.0017
JW3995	malK	maltose ABC transportor ATPase	0.0159	0.5248
JW3996	lamB	maltose outer membrane porin (maltoporin)	0.0324	0.3355
JW3997	malM	maltose regulon periplasmic protein	-0.0124	0.1705
JW3998	yjbI	pseudogene, SopA-related, pentapeptide repeats-containing	-0.1504	0.0009
JW4002	dgkA	diacylglycerol kinase	-0.0105	0.7889
JW4004	dinF	oxidative stress resistance protein; putative MATE family efflux pump; UV and mitomycin C inducible protein	0.1563	0.0707
JW4005	yjbJ	stress-induced protein, UPF0337 family	0.1386	0.0070
JW4007	yjbL	uncharacterized protein	-0.0817	0.0186
JW4008	yjbM	uncharacterized protein	0.0663	0.0454
JW4011	qor	quinone oxidoreductase, NADPH-dependent	-0.0081	0.5312
JW4013	alr	alanine racemase, biosynthetic, PLP-binding	-0.1504	0.2819
JW4014	tyrB	tyrosine aminotransferase, tyrosine-repressible, PLP-dependent	0.0744	0.0710
JW4015	aphA	acid phosphatase/phosphotransferase, class B, non-specific	-0.0096	0.7330
JW4017	yjbQ	thiamine phosphate synthase	-0.0361	0.1725
JW4018	yjbR	DUF419 family protein	0.0424	0.1049
JW4019	uvrA	ATPase and DNA damage recognition protein of nucleotide excision repair excinuclease UvrABC	0.0591	0.1648
JW4022	yjcC	putative membrane-anchored cyclic-di-GMP phosphodiesterase	-0.1103	0.0000
JW4023	soxS	superoxide response regulon transcriptional activator; autoregulator	0.1293	0.0000
JW4024	soxR	redox-sensitive transcriptional activator of soxS; autorepressor	0.0624	0.0130
JW4025	yjcD	guanine/hypoxanthine permease, high affinity; guanine/hypoxanthine:H+ symporter	0.0543	0.1601
JW4026	yjcE	putative cation/proton antiporter	-0.0061	0.8326
JW4028	actP	acetate transporter	-0.0061	0.6418
JW4029	yjcH	DUF485 family inner membrane protein	-0.0764	0.0216
JW4030	acs	acetyl-CoA synthetase	-0.0423	0.5786
JW4031	nrfA	nitrite reductase, formate-dependent, cytochrome	0.0311	0.3126
JW4032	nrfB	nitrite reductase, formate-dependent, penta-heme cytochrome c	0.1010	0.0378
JW4033	nrfC	formate-dependent nitrite reductase, 4Fe4S subunit	-0.0241	0.6271

JW4034	nrfD	formate-dependent nitrite reductase, membrane subunit	-0.0174	0.5623
JW4035	nrfE	heme lyase (NrfEFG) for insertion of heme into c552, subunit NrfE	-0.0107	0.8215
JW4036	nrfF	heme lyase (NrfEFG) for insertion of heme into c552, subunit NrfF	0.2976	0.0693
JW4037	nrfG	heme lyase (NrfEFG) for insertion of heme into c552, subunit NrfG	0.0685	0.1597
JW4038	gltP	glutamate/aspartate:proton symporter	-0.0731	0.0405
JW4039	yjcO	Sel1 family TPR-like repeat protein	-0.0687	0.0072
JW4040	fdhF	formate dehydrogenase-H, selenopolypeptide subunit	0.0523	0.0780
JW4041	yjcP	outer membrane factor of efflux pump	-0.0859	0.0026
JW4042	yjcQ	membrane translocase (MDR) of MdtNOP efflux pump, PET family	-0.0754	0.0007
JW4043	yjcR	membrane fusion protein of efflux pump	-0.0108	0.6887
JW4046	alsE	allulose-6-phosphate 3-epimerase	-0.0257	0.3193
JW4047	alsC	D-allose ABC transporter permease	-0.0934	0.0054
JW4048	alsA	D-allose ABC transporter ATPase	-0.0297	0.1739
JW4049	alsB	D-allose ABC transporter periplasmic binding protein	0.0198	0.4884
JW4050	rpiR	d-allose-inducible als operon transcriptional repressor; autorepressor; repressor of rpiR	-0.0240	0.6215
JW4051	rpiB	ribose 5-phosphate isomerase B/allose 6-phosphate isomerase	-0.1189	0.0032
JW4053	phnP	5-phospho-alpha-D-ribosyl 1,2-cyclic phosphate phosphodiesterase	-0.0207	0.4011
JW4054	phnO	aminoalkylphosphonate N-acetyltransferase	-0.0207	0.4535
JW4055	phnN	ribose 1,5-bisphosphokinase	0.0786	0.0198
JW4056	phnM	ribophosphonate triphosphate hydrolase	-0.0012	0.9414
JW4057	phnL	ribophosphonate triphosphate synthase subunit; putative ABC transporter-related ATPase	0.0485	0.1280
JW4059	phnJ	carbon-phosphorus lyase, SAM-dependent	-0.1329	0.0066
JW4060	phnI	ribophosphonate triphosphate synthase complex putative catalytic subunit	-0.0220	0.1169
JW4061	phnH	ribophosphonate triphosphate synthase subunit	-0.0096	0.7488
JW4062	phnG	ribophosphonate triphosphate synthase subunit	-0.1133	0.0005
JW4063	phnF	putative DNA-binding transcriptional regulator of phosphonate uptake and biodegradation	0.0349	0.1366
JW4064	phnE	defective phosphonate ABC transporter permease	-0.0266	0.0833
JW4065	phnE	Putative cryptic phosphonate transport system permease protein PhnE2	0.0100	0.6197
JW4066	phnD	phosphonate ABC transporter periplasmic binding protein	0.0655	0.0198
JW4067	phnC	phosphonate ABC transporter ATPase	-0.1184	0.0185

JW4068	phnB	metalloprotein superfamily protein	-0.0488	0.0156
JW4069	phnA	zinc-ribbon family protein	0.0453	0.0025
JW4070	yjdA	clamp-binding sister replication fork colocalization protein, dynamin-related	0.2562	0.0017
JW4072	proP	proline/glycine betaine transporter	0.0067	0.7755
JW4073	basS	sensory histidine kinase in two-component regulatory system with BasR	0.1020	0.0000
JW4074	basR	response regulator in two-component regulatory system with BasS	0.0324	0.1339
JW4076	adiC	arginine:agmatine antiporter	0.0286	0.3353
JW4077	adiY	adi system transcriptional activator	0.0107	0.6858
JW4079	melR	melibiose operon transcriptional regulator; autoregulator	-0.0051	0.7454
JW4080	melA	alpha-galactosidase, NAD(P)-binding	0.0615	0.1831
JW4081	melB	melibiose:sodium symporter	0.1118	0.1120
JW4082	yjdF	DUF2238 family inner membrane protein	-0.0721	0.0142
JW4083	fumB	anaerobic class I fumarate hydratase (fumarase B)	-0.0827	0.0001
JW4084	dcuB	C4-dicarboxylate transporter, anaerobic; DcuS co-sensor	-0.0881	0.0167
JW4085	dcuR	response regulator in two-component regulatory system with DcuS	-0.0110	0.6627
JW4086	dcuS	sensory histidine kinase in two-component regulatory system with DcuR, regulator of anaerobic fumarate respiration	-0.0184	0.3325
JW4087	yjdI	putative 4Fe-4S mono-cluster protein	-0.0204	0.4668
JW4088	yjdJ	GNAT family putative N-acetyltransferase	0.0560	0.0305
JW4089	yjdK	antitoxin of GhoTS toxin-antitoxin pair; endonuclease for ghoT mRNA	-0.0227	0.4430
JW4090	lysU	lysine tRNA synthetase, inducible	-0.0620	0.0700
JW4091	yjdL	dipeptide and tripeptide permease	0.0351	0.3245
JW4092	cadA	lysine decarboxylase, acid-inducible	-0.2249	0.1350
JW4093	cadB	putative lysine/cadaverine transporter	0.0206	0.5872
JW4094	cadC	cadBA operon transcriptional activator	-0.0315	0.3259
JW4097	cutA	divalent-cation tolerance protein, copper sensitivity	0.1432	0.1481
JW4099	aspA	aspartate ammonia-lyase	0.0219	0.2787
JW4100	fxsA	suppressor of F exclusion of phage T7	0.0443	0.3570
JW4101	yjeH	L-methionine and branched chain amino acid exporter	0.0143	0.3997
JW4103	groL	Cpn60 chaperonin GroEL, large subunit of GroESL	-0.0812	0.0491
JW4106	yjeK	EF-P-Lys34 lysylation protein; weak lysine 2,3-aminomutase	-0.0072	0.8103
JW4107	efp	polyproline-specific translation elongation factor EF-P	-0.0543	0.3546

JW4108	ecnB	entericidin B membrane lipoprotein	0.1443	0.0005
JW4110	blc	outer membrane lipoprotein cell division and growth lipocalin	-0.0983	0.0618
JW4111	ampC	penicillin-binding protein; beta-lactamase, intrinsically weak	0.0545	0.0218
JW4112	frdD	fumarate reductase (anaerobic), membrane anchor subunit	0.0239	0.2464
JW4113	frdC	fumarate reductase (anaerobic), membrane anchor subunit	0.1076	0.0983
JW4114	frdB	fumarate reductase (anaerobic), Fe-S subunit	0.0218	0.6479
JW4115	frdA	anaerobic fumarate reductase catalytic and NAD/flavoprotein subunit	-0.0389	0.3884
JW4116	poxA	Elongation Factor P Lys34 lysyltransferase	0.1730	0.1141
JW4118	yjeN	uncharacterized protein	0.0754	0.0472
JW4119	yjeO	inner membrane protein	-0.0182	0.6790
JW4120	yjeP	mechanosensitive channel protein, miniconductance	-0.0538	0.0068
JW4122	rsgA	ribosome small subunit-dependent GTPase A	0.0601	0.1688
JW4124	yjeS	epoxyqueuosine reductase, cobalamine-stimulated; queosine biosynthesis	-0.0341	0.1559
JW4125	yjeF	bifunctional NAD(P)H-hydrate repair enzyme; C-terminal domain ADP-dependent (S)-NAD(P)H-hydrate dehydratase and N-terminal domain NAD(P)H-hydrate epimerase	-0.0619	0.0227
JW4127	amiB	N-acetylmuramoyl-l-alanine amidase II	-0.1819	0.0010
JW4128	mutL	methyl-directed mismatch repair protein	-0.1658	0.0026
JW4129	miaA	delta(2)-isopentenylpyrophosphate tRNA-adenosine transferase	0.0214	0.4846
JW4130	hfq	global sRNA chaperone; HF-I, host factor for RNA phage Q beta replication	0.0734	0.0001
JW4131	hflX	GTPase, stimulated by 50S subunit binding; Mn(2+) homeostasis regulator	-0.0430	0.2341
JW4132	hflK	modulator for HflB protease specific for phage lambda cII repressor	0.0518	0.2704
JW4133	hflC	HflB protease modulator specific for phage lambda cII repressor	0.0505	0.3469
JW4134	yjeT	DUF2065 family protein	0.0937	0.0126
JW4135	purA	adenylosuccinate synthetase	0.0376	0.1433
JW4136	yjeB	nitric oxide-sensitive repressor for NO regulon	-0.0382	0.2774
JW4139	yjfI	DUF2170 family protein	0.0039	0.8655
JW4140	yjfJ	PspA/IM30 family protein	0.0347	0.2007

JW4141	yjfK	DUF2491 family protein	0.0022	0.9768
JW4142	yjfL	UPF0719 family inner membrane protein	-0.0275	0.3226
JW4143	yjfM	DUF1190 family protein	-0.0607	0.0003
JW4144	yjfC	ATP-Grasp family ATPase	0.1050	0.0129
JW4148	yjfP	acyl CoA esterase	-0.1375	0.0270
JW4149	ulaR	transcriptional repressor for the L-ascorbate utilization divergent operon	-0.2324	0.2721
JW4152	ulaB	L-ascorbate-specific enzyme IIB component of PTS	0.0130	0.8562
JW4153	ulaC	L-ascorbate-specific enzyme IIA component of PTS	-0.0219	0.4040
JW4154	ulaD	3-keto-L-gulonate 6-phosphate decarboxylase	0.1350	0.0014
JW4155	ulaE	L-xylulose 5-phosphate 3-epimerase	0.0819	0.1145
JW4156	ulaF	L-ribulose 5-phosphate 4-epimerase	0.0980	0.0026
JW4157	yjfY	YhcN family protein, periplasmic	-0.4733	0.0015
JW4158	rpsF	30S ribosomal subunit protein S6	-0.1205	0.1096
JW4159	priB	primosomal protein N	0.0243	0.2086
JW4161	rplI	50S ribosomal subunit protein L9	-0.2102	0.0002
JW4162	yjfZ	uncharacterized protein	0.1611	0.0407
JW4163	ytfA	pseudogene, related to transcriptional regulators	-0.0197	0.2677
JW4166	cycA	D-alanine/D-serine/glycine transporter	-0.0520	0.1373
JW4167	ytfE	iron-sulfur cluster repair protein RIC	0.0676	0.1233
JW4168	ytfF	DMT transporter family inner membrane protein	-0.0012	0.9629
JW4169	ytfG	NAD(P)H:quinone oxidoreductase	-0.4926	0.0006
JW4171	cpdB	2':3'-cyclic-nucleotide 2'-phosphodiesterase	0.0088	0.7678
JW4172	cysQ	3'(2'),5'-bisphosphate nucleotidase	0.1905	0.0079
JW4175	ytfJ	putative transcriptional regulator	-0.2525	0.0000
JW4177	ytfL	UPF0053 family inner membrane protein	-0.0305	0.4735
JW4178	msrA	methionine sulfoxide reductase A	-0.0462	0.2025
JW4179	ytfM	translocation and assembly module for autotransporter export, outer membrane subunit	-0.1531	0.1718
JW4180	ytfN	translocation and assembly module for autotransporter export, inner membrane subunit	-0.0401	0.8273
JW4181	ytfP	GGCT-like protein	0.1627	0.3377
JW4182	yzfA	Putative uncharacterized protein b4223	0.0920	0.1508
JW4184	chpB	toxin of the ChpB-ChpS toxin-antitoxin system	0.0931	0.0269
JW4186	ytfQ	galactofuranose ABC transporter periplasmic binding protein	0.1802	0.1997
JW4191	fbp	fructose-1,6-bisphosphatase I	-0.0208	0.2967
JW4192	mpl	UDP-N-acetylmuramate:L-alanyl-gamma-D-glutamyl-meso-diaminopimelate ligase	0.0201	0.5922
JW4193	yjgA	ribosome-associated UPF0307 family protein	-0.2700	0.0359

JW4194	pmbA	putative antibiotic peptide MccB17 maturation peptidase	0.1150	0.0054
JW4195	cybC	pseudogene, truncated cytochrome b562	-0.0094	0.6538
JW4196	nrdG	anaerobic ribonucleotide reductase activating protein	-0.0067	0.7174
JW4197	nrdD	anaerobic ribonucleoside-triphosphate reductase	-0.0578	0.0812
JW4198	treC	trehalose-6-P hydrolase	0.0370	0.4780
JW4200	treR	trehalose 6-phosphate-inducible trehalose regulon transcriptional repressor	0.0273	0.4323
JW4201	mgtA	magnesium transporter	-0.0972	0.0011
JW4203	pyrI	aspartate carbamoyltransferase, regulatory subunit	0.0490	0.0029
JW4204	pyrB	aspartate carbamoyltransferase, catalytic subunit	0.0275	0.4760
JW4205	pyrL	pyrBI operon leader peptide	-0.2200	0.0453
JW4206	yjgH	UPF0131 family protein	0.0603	0.0498
JW4207	yjgI	c-di-GMP-binding biofilm dispersal mediator protein	-0.1707	0.1406
JW4208	yjgJ	transcriptional repressor for divergent bdcA	-0.0107	0.8495
JW4211	argI	ornithine carbamoyltransferase 1	0.0471	0.0001
JW4212	yjgD	protein inhibitor of RNase E	-0.4522	0.0024
JW4216	holC	DNA polymerase III, chi subunit	0.0484	0.1066
JW4217	pepA	multifunctional aminopeptidase A: a cyteinylglycinase, transcription regulator and site-specific recombination factor	0.0728	0.1350
JW4220	yjgR	DUF853 family protein with NTPase fold	-0.0242	0.8872
JW4221	idnR	transcriptional repressor, 5-gluconate-binding	-0.0078	0.4805
JW4222	idnT	L-idonate and D-gluconate transporter	0.0330	0.3099
JW4223	idnO	5-keto-D-gluconate-5-reductase	0.0687	0.0213
JW4224	idnD	L-idonate 5-dehydrogenase, NAD-binding	0.0500	0.2353
JW4225	idnK	D-gluconate kinase, thermosensitive	0.0519	0.2180
JW4227	intB	pseudogene, integrase homology	-0.0405	0.0967
JW4233	yjgW	Putative uncharacterized protein	-0.0341	0.2565
JW4234	yjgX	Putative uncharacterized protein	0.0069	0.7928
JW4236	yjgZ	uncharacterized protein	-0.0212	0.3792
JW4242	yjhE	pseudogene, KpLE2 phage-like element	-0.0398	0.2503
JW4246	yjhV	pseudogene, KpLE2 phage-like element	-0.0538	0.6143
JW4247	fecE	ferric citrate ABC transporter ATPase	-0.0734	0.0344
JW4248	fecD	ferric citrate ABC transporter permease	0.0285	0.4260
JW4249	fecC	ferric citrate ABC transporter permease	-0.0457	0.3512
JW4250	fecB	ferric citrate ABC transporter periplasmic binding protein	0.0804	0.0031

JW4251	fecA	TonB-dependent outer membrane ferric citrate transporter and signal transducer; ferric citrate extracelluar receptor; FecR-interacting protein	-0.1459	0.0156
JW4252	fecR	anti-sigma transmembrane signal transducer for ferric citrate transport; periplasmic FecA-bound ferric citrate sensor and cytoplasmic FecI ECF sigma factor activator	0.0238	0.3985
JW4253	fecI	RNA polymerase sigma-19 factor, fec operon-specific; ECF sigma factor	0.0188	0.6023
JW4258	yjhF	putative transporter	0.1445	0.1466
JW4259	yjhG	putative dehydratase	0.3226	0.0054
JW4261	yjhI	putative DNA-binding transcriptional regulator	0.0289	0.3476
JW4262	sgcR	putative DNA-binding transcriptional regulator	-0.0242	0.2080
JW4263	sgcE	putative epimerase	-0.0477	0.0132
JW4264	sgcA	putative phosphotransferase enzyme IIA component	0.1048	0.0038
JW4265	sgcQ	putative nucleoside triphosphatase	0.0656	0.1304
JW4266	sgcC	putative PTS system EIIC permease component	0.1094	0.0001
JW4268	yjhP	putative methyltransferase	-0.1243	0.0075
JW4269	yjhQ	GNAT family putative N-acetyltransferase	-0.0518	0.0517
JW4271	yjhR	pseudogene, helicase family	0.2269	0.1958
JW4272	yjhS	9-O-acetyl N-acetylneuraminic acid esterase	-0.0243	0.1039
JW4275	fimB	tyrosine recombinase/inversion of on/off regulator of fimA	0.0652	0.0612
JW4277	fimA	major type 1 subunit fimbrin (pilin)	-0.0139	0.6349
JW4279	fimC	periplasmic chaperone	0.0005	0.9940
JW4281	fimF	minor component of type 1 fimbriae	-0.0370	0.1984
JW4282	fimG	minor component of type 1 fimbriae	-0.0414	0.0220
JW4283	fimH	minor component of type 1 fimbriae	-0.0215	0.8044
JW4284	gntP	fructuronate transporter	0.0440	0.0552
JW4285	uxuA	mannonate hydrolase	0.0645	0.1524
JW4286	uxuB	D-mannonate oxidoreductase, NAD-dependent	-0.0113	0.4224
JW4287	uxuR	fructuronate-inducible hexuronate regulon transcriptional repressor; autorepressor	-0.0225	0.0727
JW4288	yjiC	uncharacterized protein	-0.1219	0.3702
JW4290	yjiE	hypochlorite-responsive transcription factor	-0.0043	0.9059
JW4291	iadA	isoaspartyl dipeptidase	-0.1755	0.0020
JW4292	yjiG	SpmB family inner membrane protein	-0.4030	0.0028
JW4295	yjiJ	DUF1228 family putative inner membrane MFS superfamily transporter	0.0628	0.0018

JW4299	yjiN	zinc-type alcohol dehydrogenase-like protein	0.1362	0.0001
JW4300	yjiO	multidrug efflux system protein	-0.0906	0.0008
JW4302	yjiQ	Putative inactive recombination-promoting nuclease-like protein	0.0117	0.6508
JW4303	yjiR	putative DNA-binding transcriptional regulator/putative aminotransferase	0.1605	0.0009
JW4304	yjiS	DUF1127 family protein	-0.2022	0.3157
JW4310	yjiW	toxic peptide regulated by antisense sRNA symR	0.0228	0.5049
JW4311	hsdS	specificity determinant for hsdM and hsdR	-0.2372	0.0121
JW4312	hsdM	DNA methyltransferase M	-0.0950	0.0897
JW4313	hsdR	endonuclease R Type I restriction enzyme	-0.0018	0.9258
JW4314	mrr	methylated adenine and cytosine restriction protein	0.2785	0.0634
JW4316	yjiX	DUF466 family protein	-0.3353	0.0251
JW4318	tsr	methyl-accepting chemotaxis protein I, serine sensor receptor	0.0344	0.1160
JW4319	yjiZ	putative L-galactonate:H+ symporter	-0.0039	0.8544
JW4326	dnaT	DNA biosynthesis protein (primosomal protein I)	0.1234	0.0026
JW4327	yjjB	DUF3815 family inner membrane protein	0.0881	0.0071
JW4329	yjjQ	putative transcriptional regulator	-0.1857	0.2493
JW4331	fhuF	ferric iron reductase involved in ferric hydroximate transport	0.0903	0.0868
JW4333	rsmC	16S rRNA m(2)G1207 methyltransferase, SAM-dependent	-0.1423	0.0019
JW4334	holD	DNA polymerase III, psi subunit	0.0973	0.2152
JW4335	rimI	ribosomal-protein-S18-alanine N-acetyltransferase	-0.0744	0.0278
JW4336	yjjG	dUMP phosphatase	-0.2930	0.0726
JW4338	osmY	salt-inducible putative ABC transporter periplasmic binding protein	-0.0984	0.0172
JW4340	yjjU	putative patatin-like family phospholipase	0.0650	0.0209
JW4341	yjjV	putative DNase	-0.0077	0.7773
JW4342	yjjW	putative pyruvate formate lyase activating enzyme	0.1328	0.1701
JW4343	yjjI	DUF3029 family protein, putative glycine radical enzyme	0.2695	0.0000
JW4344	deoC	2-deoxyribose-5-phosphate aldolase, NAD(P)-linked	-0.0257	0.6535
JW4345	deoA	thymidine phosphorylase	0.0765	0.0348
JW4346	deoB	phosphopentomutase	-0.0665	0.0494
JW4347	deoD	purine nucleoside phosphorylase 1; nicotinamide 1-beta-D-riboside phosphorylase	-0.0232	0.5024

JW4348	yjjJ	putative protein kinase	0.0720	0.0844
JW4349	lplA	lipoate-protein ligase A	-0.0466	0.3636
JW4350	ytjB	SMP_2 family putative membrane-anchored periplasmic protein	-0.0090	0.7781
JW4351	serB	3-phosphoserine phosphatase	0.1341	0.0081
JW4352	radA	DNA repair protein	0.0042	0.9628
JW4354	yjjK	energy-dependent translational throttle A	0.4212	0.0103
JW4355	slt	lytic murein transglycosylase, soluble	0.0712	0.0456
JW4356	trpR	transcriptional repressor, tryptophan-binding	-0.1192	0.0019
JW4358	ytjC	phosphatase	0.0074	0.8471
JW4359	rob	right oriC-binding transcriptional activator, AraC family	0.0060	0.9081
JW4360	creA	putative periplasmic protein	0.1056	0.0000
JW4361	creB	response regulator in two-component regulatory system with CreC	-0.0780	0.0001
JW4362	creC	sensory histidine kinase in two-component regulatory system with CreB or PhoB	-0.0137	0.1019
JW4364	arcA	response regulator in two-component regulatory system with ArcB or CpxA	0.1183	0.1979
JW4365	yjjY	uncharacterized protein	-0.3921	0.0070
JW4366	yjtD	putative methyltransferase	0.2488	0.0193
JW4367	thrL	thr operon leader peptide	-0.0924	0.0001
JW5001	htgA	Uncharacterized protein	0.0405	0.1823
JW5002	hokC	Toxic protein HokC	-0.0968	0.0023
JW5003	yaaY	uncharacterized protein	-0.0995	0.0090
JW5004	caiE	stimulator of CaiD and CaiB enzyme activities	-0.0022	0.8914
JW5005	yabI	ionizing radiation survival protein; DedA family inner membrane protein	-0.0128	0.6782
JW5008	yacG	DNA gyrase inhibitor	0.0558	0.0148
JW5009	hpt	hypoxanthine phosphoribosyltransferase	-0.0239	0.0237
JW5011	ligT	2'-5' RNA ligase	0.0688	0.0254
JW5012	clcA	H(+)/Cl(-) exchange transporter	-0.3346	0.0226
JW5013	cdaR	carbohydrate diacid regulon transcriptional regulator; autoregulator	-0.2759	0.0803
JW5014	yaeI	phosphodiesterase with model substrate bis-pNPP	-0.0734	0.3659
JW5016	yaeF	putative lipoprotein	0.0292	0.2046
JW5017	yafD	endo/exonuclease/phosphatase family protein	0.0365	0.3139
JW5018	mltD	putative membrane-bound lytic murein transglycosylase D	0.0780	0.0056
JW5019	yafV	putative NAD(P)-binding C-N hydrolase family amidase	0.0139	0.5396
JW5020	fadE	acyl coenzyme A dehydrogenase	0.0516	0.0902

JW5022	yafX	CP4-6 prophage; uncharacterized protein	0.0223	0.5818
JW5023	ykfF	CP4-6 prophage; uncharacterized protein	0.0122	0.6933
JW5027	mmuP	CP4-6 prophage; putative S-methylmethionine transporter	0.0352	0.0156
JW5030	yagV	ECP production pilus chaperone	0.0492	0.1152
JW5031	ykgK	putative transcriptional regulator for the ecp operon	0.0173	0.3899
JW5033	ykgL	uncharacterized protein	0.0100	0.3456
JW5034	ykgM	RpmJ-like protein	-0.0394	0.0572
JW5035	ykgM	50S ribosomal protein L31 type B; alternative zinc-limitation L31 protein	-0.0549	0.0027
JW5037	ykgA	pseudogene, AraC family	0.0550	0.0642
JW5038	ykgB	reactive chlorine species (RCS) stress resistance inner membrane protein	0.0380	0.0660
JW5039	ykgI	reactive chlorine species (RCS) stress resistance periplasmic protein	0.0094	0.4234
JW5040	ykgC	reactive chlorine stress species (RCS) resistance protein; pyridine nucleotide-dependent disulfide oxidoreductase family	0.0639	0.0187
JW5041	ykgE	cysteine-rich LutA family protein; putative electron transport chain YkgEFG component	-0.0679	0.0001
JW5042	ykgG	LutC family protein; putative electron transport chain YkgEFG component	0.0412	0.0083
JW5044	yahM	uncharacterized protein	0.0328	0.1562
JW5046	mhpT	3-hydroxyphenylpropionic transporter	0.0022	0.9107
JW5049	ykiB	Uncharacterized protein	0.0147	0.5835
JW5051	yaiU	Uncharacterized protein	0.0870	0.0002
JW5052	ampH	D-alanyl-D-alanine-carboxypeptidase/endopeptidase; penicillin-binding protein; weak beta-lactamase	0.0244	0.0508
JW5053	yaiZ	DUF2754 family putative inner membrane protein	0.0660	0.0365
JW5054	psiF	PsiF family protein	0.0196	0.5100
JW5055	proY	proline-specific permease	-0.0106	0.5444
JW5056	yajI	putative lipoprotein	0.0687	0.0138
JW5057	yajL	oxidative-stress-resistance chaperone	-0.1063	0.0149
JW5058	yajQ	phage Phi6 host factor, ATP/GTP binding protein	0.0658	0.0936
JW5059	yajR	putative transporter	0.0415	0.0238
JW5060	bolA	stationary-phase morphogene, transcriptional repressor for mreB; also regulator for dacA, dacC, and ampC	-0.1315	0.0054
JW5061	mdlB	putative multidrug ABC transporter ATPase	-0.0161	0.5244
JW5062	ylaB	putative membrane-anchored cyclic-di-GMP phosphodiesterase	-0.0150	0.5285
JW5063	ylaC	DUF1449 family inner membrane protein	-0.0571	0.0235

JW5065	ybbJ	inner membrane protein; stimulator of the QmcA suppressor of ftsH-htpX	0.1017	0.0135
JW5066	ybbM	iron export ABC transporter permease; peroxide resistance protein	-0.1216	0.0086
JW5067	ybbN	DnaK co-chaperone, thioredoxin-like protein	-0.0353	0.3053
JW5070	ybcJ	ribosome-associated protein; putative RNA-binding protein	0.0436	0.1855
JW5071	sfmH	FimA homolog, function unknown	0.0396	0.3859
JW5072	sfmF	FimA homolog, function unknown	0.0210	0.3100
JW5073	fimZ	response regulator family protein	0.1139	0.0110
JW5076	ylcG	uncharacterized protein, DLP12 prophage	0.0954	0.0269
JW5079	rzpD	DLP12 prophage; putative murein endopeptidase	0.1978	0.0014
JW5080	rzoD	DLP12 prophage; putative lipoprotein	0.0987	0.0135
JW5081	ybcV	DLP12 prophage; DUF1398 family protein	-0.0640	0.0182
JW5082	cusS	copper-sensing histidine kinase in two-component regulatory system with CusR	-0.0241	0.0635
JW5083	ybdF	DUF419 family protein	0.0191	0.2516
JW5084	hokE	toxic polypeptide, small	-0.1768	0.1328
JW5086	fepA	ferrienterobactin outer membrane transporter	-0.2240	0.0002
JW5087	citF	citrate lyase, citrate-ACP transferase (alpha) subunit	-0.0347	0.1675
JW5089	lipB	octanoyltransferase; octanoyl-[ACP]:protein N-octanoyltransferase	0.1625	0.0007
JW5090	ybeB	ribosomal silencing factor	0.0315	0.0979
JW5091	ybeQ	Sel1 family TPR-like repeat protein	-0.0358	0.1228
JW5092	gltI	glutamate/aspartate periplasmic binding protein	0.0044	0.8863
JW5094	ybfG	pseudogene	0.0546	0.1660
JW5095	ybfH	Uncharacterized protein	-0.3785	0.0063
JW5096	kdpE	response regulator in two-component regulatory system with KdpD	-0.0904	0.0027
JW5097	abrB	regulator of aidB expression; inner membrane protein	0.0081	0.8288
JW5098	ybgO	putative fimbrial protein	-0.0285	0.3442
JW5099	ybgQ	putative outer membrane protein	0.0468	0.6002
JW5100	tolB	periplasmic protein	-0.2806	0.0478
JW5102	ybhT	AcrAB-TolC efflux pump accessory protein, membrane-associated	-0.1645	0.0424
JW5103	ybhJ	aconitase family protein	-0.0104	0.5161
JW5104	ybhF	putative ABC transporter ATPase	0.0185	0.5656
JW5105	ybiX	Fe(II)-dependent oxygenase superfamily protein	-0.3293	0.0158

JW5106	ybiM	colanic acid mucoidy stimulation protein	0.0088	0.7685
JW5107	ybiN	23S rRNA m(6)A1618 methyltransferase, SAM-dependent	-0.0068	0.7665
JW5108	ybiO	mechanosensitive channel protein, intermediate conductance	0.0384	0.4697
JW5109	fsaA	fructose-6-phosphate aldolase 1	-0.1360	0.2302
JW5112	ybjG	undecaprenyl pyrophosphate phosphatase	0.0515	0.0024
JW5113	ybjI	5-amino-6-(5-phospho-D-ribitylamino)uracil phosphatase; pyrimidine phosphatase; riboflavin synthesis	0.0717	0.3597
JW5114	ybjK	transcriptional regulator of csgD and ybiJI; autoregulator	-0.1110	0.0000
JW5116	ybjT	putative NAD-dependent oxidoreductase	0.0126	0.5495
JW5117	hcr	HCP oxidoreductase, NADH-dependent	-0.0178	0.5868
JW5118	dmsA	dimethyl sulfoxide reductase, anaerobic, subunit A	-0.0116	0.5052
JW5119	ycaM	putative transporter	0.1367	0.0248
JW5120	ycaI	ComEC family inner membrane protein	0.0077	0.7868
JW5121	ssuC	aliphatic sulfonate ABC transporter permease	0.0200	0.2970
JW5122	ycbQ	laminin-binding fimbrin subunit	-0.0033	0.7894
JW5123	ycbV	putative fimbrial-like adhesin protein	-0.0358	0.5316
JW5124	ycbF	putative periplasmic pilin chaperone	-0.0173	0.4748
JW5125	ycbW	FtsZ stabilizer	-0.0241	0.3688
JW5126	ycbX	6-N-hydroxylaminopurine detoxification oxidoreductase	-0.0609	0.1246
JW5127	ymbA	OM-anchored periplasmic lipoprotein component of the putative PqiABC transporter, paraquat-inducible	0.0180	0.2497
JW5128	yccS	putative transporter, FUSC superfamily inner membrane protein	0.0083	0.6826
JW5129	mgsA	methylglyoxal synthase	0.0686	0.0952
JW5130	yccU	putative CoA-binding protein	0.0406	0.0660
JW5131	yccX	weak acylphosphatase	-0.0157	0.7192
JW5132	etp	O-antigen capsule forming protein-tyrosine-phosphatase; Etk-P dephosphorylase	-0.0428	0.6003
JW5133	ymcD	O-antigen capsule production threonine-rich inner membrane protein	-0.0523	0.1710
JW5135	torS	hybrid sensory histidine kinase in two-component regulatory system with TorR	-0.0011	0.9773
JW5136	ymdF	KGG family protein	-0.0659	0.1704
JW5137	ycdG	pyrimidine permease	0.0280	0.1029
JW5138	ycdH	flavin:NADH reductase	0.0775	0.0499
JW5139	ycdL	ureidoacrylate amidohydrolase	-0.0451	0.0216
JW5141	ycdN	Putative inactive ferrous iron permease	0.0359	0.2927

JW5142	ycdR	poly-beta-1,6-N-acetyl-D-glucosamine (PGA) N-deacetylase outer membrane export lipoprotein	-0.0976	0.0119
JW5143	ycdT	diguanylate cyclase, membrane-anchored	0.3162	0.0602
JW5145	ymdE	pseudogene	-0.0646	0.0312
JW5146	ycdW	glyoxylate/hydroxypyruvate reductase A	0.0433	0.1985
JW5147	ycdZ	DUF1097 family inner membrane protein	-0.0183	0.7129
JW5150	ymdC	stationary phase cardiolipin synthase 3	0.0141	0.2153
JW5151	yceK	outer membrane integrity lipoprotein	-0.0057	0.8435
JW5152	yceP	biofilm regulator	-0.2638	0.0307
JW5153	flgH	flagellar protein of basal-body outer-membrane L ring	-0.1673	0.0002
JW5155	yceF	m(7)GTP pyrophosphatase	-0.0098	0.6946
JW5156	plsX	putative phosphate acyltransferase	0.0035	0.6825
JW5157	ycfM	OM lipoprotein stimulator of MrcB transpeptidase	0.0054	0.8209
JW5158	ycfP	putative UPF0227 family esterase	0.0093	0.6749
JW5159	ycfQ	repressor for bhsA(ycfR)	-0.1303	0.1310
JW5164	ymfA	DUF3592 family inner membrane protein	-0.0307	0.0596
JW5165	hflD	putative lysogenization regulator	0.0325	0.4357
JW5166	ymfE	e14 prophage; putative inner membrane protein	0.0866	0.0010
JW5168	ymfI	e14 prophage; uncharacterized protein	-0.0857	0.0961
JW5169	ymfT	e14 prophage; putative DNA-binding transcriptional regulator	-0.1488	0.0000
JW5170	ymfP	pseudogene, e14 prophage	0.0024	0.9029
JW5171	ymfS	e14 prophage; uncharacterized protein	0.0117	0.6126
JW5172	stfE	pseudogene, e14 prophage; side tail fiber protein fragment family	-0.0954	0.0195
JW5173	icdC	pseudogene, isocitrate dehydrogenase C-terminal gene fragment	0.0823	0.0000
JW5174	ycgG	putative membrane-anchored cyclic-di-GMP phosphodiesterase	0.0812	0.0002
JW5176	ycgH	Uncharacterized protein	-0.0133	0.6258
JW5177	ymgD	periplasmic protein, HdeA structural homolog	-0.0102	0.8377
JW5179	ymgH	Putative uncharacterized protein	-0.0689	0.0580
JW5180	ycgN	UPF0153 family cysteine cluster protein	-0.0569	0.1076
JW5181	hlyE	hemolysin E	0.0753	0.0023
JW5182	dsbB	oxidoreductase that catalyzes reoxidation of DsbA protein disulfide isomerase I	0.3677	0.0797
JW5184	cvrA	putative cation/proton antiporter	-0.0049	0.9047
JW5186	dhaL	dihydroxyacetone kinase, C-terminal domain	-0.0825	0.0774
JW5187	dhaK	dihydroxyacetone kinase, PTS-dependent, dihydroxyacetone-binding subunit	0.0782	0.0133
JW5189	ychM	C4-dicarboxylic acid transporter	0.0627	0.0083

JW5195	tonB	membrane spanning protein in TonB-ExbB-ExbD transport complex	-0.0642	0.1000
JW5196	yciO	putative RNA binding protein	0.0372	0.3180
JW5197	yciQ	enhancer of membrane protein expression; putative inner membrane protein	-0.0541	0.0453
JW5199	yciX	Uncharacterized protein	-0.0050	0.8659
JW5200	yciW	putative oxidoreductase	0.1572	0.0001
JW5201	puuA	glutamate--putrescine ligase	0.0242	0.3685
JW5202	ycjR	putative TIM alpha/beta barrel enzyme	0.0961	0.0390
JW5203	ymjB	Putative uncharacterized ABC transporter ATP-binding protein	0.0406	0.0970
JW5206	ydaM	diguanylate cyclase, csgD regulator	-0.0378	0.1018
JW5207	ydaQ	Rac prophage; conserved protein	-0.0322	0.4710
JW5208	lar	Rac prophage; restriction alleviation protein	-0.0413	0.1248
JW5209	sieB	phage superinfection exclusion protein, Rac prophage	0.0559	0.0031
JW5211	ydaW	Rac prophage; pseudogene, DNA-binding protein family	-0.0378	0.0865
JW5212	rzpR	pseudogene, Rac prophage; Bacteriophage Rz lysis protein family	-0.0078	0.8898
JW5213	rzoR	Rac prophage; putative lipoprotein	0.0875	0.0114
JW5215	ydbJ	DUF333 family putative lipoprotein	0.0873	0.0107
JW5216	ydbL	DUF1318 family protein	0.0039	0.9301
JW5217	paaD	ring 1,2-phenylacetyl-CoA epoxidase subunit	0.0440	0.1344
JW5221	ydbD	DUF2773 family methylglyoxal resistance protein	-0.1216	0.1558
JW5224	cybB	cytochrome b561	0.0706	0.0117
JW5225	hokB	toxic polypeptide, small	0.0367	0.2885
JW5226	ydcI	putative DNA-binding transcriptional regulator	-0.0611	0.0182
JW5227	yncK	Putative transposase	0.0159	0.1271
JW5228	ydcM	IS609 transposase B	0.0126	0.7603
JW5229	ydcO	BenE family inner membrane putative transporter	-0.0464	0.3655
JW5230	yncN	mRNA interferase toxin of the HicAB toxin-antitoxin system	0.0400	0.1542
JW5232	ydcX	DUF2566 family protein	-0.0029	0.8815
JW5233	yncA	methionine N-acyltransferase; L-amino acid N-acyltransferase	0.1134	0.0131
JW5234	ansP	L-asparagine transporter	-0.0023	0.8599
JW5235	yncH	IPR020099 family protein	0.0186	0.3000
JW5237	yncM	Putative transposase	0.0247	0.4219
JW5238	sfcA	malate dehydrogenase, decarboxylating, NAD-requiring; malic enzyme	-0.0520	0.2387
JW5239	bdm	biofilm-dependent modulation protein	-0.0469	0.0228
JW5240	ddpA	D,D-dipeptide ABC transporter periplasmic binding protein	-0.0654	0.1858

JW5241	yddV	diguanylate cyclase, cold- and stationary phase-induced oxygen-dependent biofilm regulator	0.0638	0.0788
JW5242	yddA	putative multidrug ABC transporter permease/ATPase	0.0067	0.8469
JW5243	ydeN	putative Ser-type periplasmic non-aryl sulfatase	-0.0264	0.3733
JW5244	yneL	pseudogene, AraC family	-0.0261	0.4031
JW5245	yneE	bestrophin family putative inner membrane protein	-0.0869	0.0091
JW5247	yneI	succinate semialdehyde dehydrogenase, NAD(P)+-dependent	-0.0189	0.6386
JW5248	marR	transcriptional repressor of multiple antibiotic resistance	-0.1747	0.1078
JW5249	marA	multiple antibiotic resistance transcriptional regulator	-0.0505	0.1239
JW5250	eamA	cysteine and O-acetyl-L-serine efflux system	-0.0355	0.4769
JW5251	ynfO	uncharacterized protein, Qin prophage	0.0049	0.8099
JW5252	ydfO	Qin prophage; DUF1398 family protein	0.0589	0.1680
JW5253	gnsB	Qin prophage; multicopy suppressor of secG(Cs) and fabA6(Ts)	-0.0035	0.9136
JW5254	ynfN	Qin prophage; cold shock-induced protein	-0.0305	0.1762
JW5255	essQ	Qin prophage; putative S lysis protein	-0.0149	0.6161
JW5257	ynfP	Uncharacterized protein	0.0240	0.1444
JW5258	ynfC	UPF0257 family lipoprotein	0.0390	0.1212
JW5259	ynfD	DUF1161 family periplasmic protein	-0.0760	0.0082
JW5260	ynfF	S- and N-oxide reductase, A subunit, periplasmic	-0.0328	0.2491
JW5261	ynfH	oxidoreductase, membrane subunit	-0.0466	0.1272
JW5262	dmsD	twin-argninine leader-binding protein for DmsA and TorA	0.0199	0.3363
JW5263	clcB	H(+)/Cl(-) exchange transporter	0.1045	0.0317
JW5264	ynfK	putative dethiobiotin synthetase	-0.0056	0.8722
JW5265	ydgJ	putative oxidoreductase	0.0097	0.7088
JW5267	slyA	global transcriptional regulator	0.0182	0.5344
JW5270	ydhO	murein DD-endopeptidase, space-maker hydrolase	0.0659	0.0713
JW5271	ydhX	putative 4Fe-4S ferridoxin-type protein; FNR, Nar, NarP-regulated protein; putative subunit of YdhYVWXUT oxidoreductase complex	-0.0746	0.0018
JW5272	ydhV	putative oxidoreductase subunit	0.0012	0.9583
JW5273	sufB	component of SufBCD Fe-S cluster assembly scaffold	0.0138	0.6961
JW5274	ydiN	putative MFS transporter, membrane protein	0.0490	0.1924
JW5275	ydiO	putative acyl-CoA dehydrogenase	0.0127	0.6945
JW5276	ydiQ	putative electron transfer flavoprotein subunit	0.0147	0.5750

JW5278	arpB	Putative ankyrin repeat protein B	-0.0432	0.0939
JW5280	pfkB	6-phosphofructokinase II	0.1007	0.0331
JW5281	ydjM	inner membrane protein regulated by LexA	-0.0646	0.0337
JW5282	astD	succinylglutamic semialdehyde dehydrogenase	-0.0432	0.1093
JW5283	ydjY	putative ferrodoxin-like lipoprotein	0.0100	0.5072
JW5284	ynjB	putative ABC transporter periplasmic binding protein	0.1042	0.0009
JW5285	ynjC	putative ABC transporter permease	0.0031	0.9408
JW5286	ynjD	putative ABC transporter ATPase	-0.0209	0.1053
JW5287	ynjE	molybdopterin synthase sulfurtransferase	0.0312	0.2507
JW5288	ynjI	inner membrane protein	-0.0171	0.5898
JW5289	ydjH	putative kinase	0.0231	0.1769
JW5290	ydjK	putative MFS sugar transporter, membrane protein	-0.0204	0.4307
JW5291	yeaJ	putative diguanylate cyclase	0.0304	0.1910
JW5292	yeaP	diguanylate cyclase	-0.1216	0.2061
JW5293	yeaV	putative transporter	-0.0025	0.9125
JW5294	yeaW	putative YeaWX dioxygenase alpha subunit; 2Fe-2S cluster	0.0504	0.0573
JW5295	yoaB	putative reactive intermediate deaminase	0.0762	0.0233
JW5296	yoaC	DUF1889 family protein	0.0735	0.0996
JW5298	yobH	uncharacterized protein	-0.0192	0.5233
JW5299	yebQ	putative transporter	0.0478	0.0136
JW5301	yebU	16S rRNA m(5)C1407 methyltransferase, SAM-dependent	-0.0547	0.3241
JW5302	yebV	uncharacterized protein	0.1027	0.0177
JW5303	yebW	uncharacterized protein	0.0397	0.1855
JW5304	yebA	murein DD-endopeptidase, space-maker hydrolase, septation protein	0.0945	0.0106
JW5306	yebB	DUF830 family protein	-0.0274	0.2396
JW5307	yecD	isochorismatase family protein	0.0101	0.7617
JW5308	yecN	MAPEG family inner membrane protein	0.0958	0.1384
JW5309	yecM	putative metal-binding enzyme	-0.0880	0.0519
JW5310	yecT	uncharacterized protein	0.0493	0.2194
JW5312	otsA	trehalose-6-phosphate synthase	-0.0658	0.0057
JW5313	yedO	D-cysteine desulfhydrase, PLP-dependent	-0.0373	0.1651
JW5316	fliO	flagellar biosynthesis protein	0.0061	0.8593
JW5317	yodD	uncharacterized protein	-0.0121	0.6134
JW5319	yedS	Putative outer membrane protein	0.0554	0.0915
JW5322	yedW	response regulator family protein	0.0032	0.8316
JW5323	yodB	cytochrome b561 homolog	-0.0202	0.6119
JW5325	yeeL	Uncharacterized protein	-0.0375	0.1061
JW5326	yoeA	CP4-44 prophage; putative disrupted hemin or colicin receptor	0.0997	0.0074

JW5327	yeeP	pseudogene, CP4-44 prophage; 50S ribosome-binding GTPase family	-0.0339	0.5977
JW5328	yoeF	pseudogene, CP4-44 putative prophage remnant	-0.0411	0.1085
JW5329	dacD	D-alanyl-D-alanine carboxypeptidase; penicillin-binding protein 6b	-0.0007	0.3284
JW5330	yeeF	putrescine importer, low affinity	0.0365	0.0789
JW5331	yoeB	toxin of the YoeB-YefM toxin-antitoxin system	0.0161	0.5484
JW5335	nudD	GDP-mannose mannosyl hydrolase	0.1329	0.0066
JW5336	yegH	inner membrane protein	0.0930	0.0009
JW5338	mdtA	multidrug efflux system, subunit A	0.0724	0.0128
JW5339	yegP	UPF0339 family protein	0.0432	0.1519
JW5340	gatR	Putative galactitol utilization operon repressor	-0.0022	0.9404
JW5343	gatY	D-tagatose 1,6-bisphosphate aldolase 2, catalytic subunit	0.0369	0.2834
JW5344	fbaB	fructose-bisphosphate aldolase class I	0.0115	0.6414
JW5345	yegX	putative family 25 glycosyl hydrolase	0.1111	0.0685
JW5346	yohN	periplasmic modulator of Ni and Co efflux	0.0185	0.5114
JW5349	yehL	putative hexameric AAA+ MoxR family ATPase	0.0026	0.8971
JW5350	yehP	VMA domain putative YehL ATPase stimulator	-0.0092	0.7783
JW5351	yehR	lipoprotein, DUF1307 family	-0.0473	0.0595
JW5352	yehT	response regulator inducing btsT; two-component system BtsSR	-0.0511	0.0409
JW5353	yehU	sensory kinase regulating btsT; two-component system BtsSR	0.0335	0.3169
JW5354	yohO	putative membrane protein	-0.0086	0.8038
JW5355	pbpG	D-alanyl-D-alanine endopeptidase	0.0349	0.2605
JW5356	yohC	Yip1 family inner membrane protein	-0.0943	0.0095
JW5358	yohH	Putative multidrug resistance outer membrane protein	0.1486	0.0060
JW5359	yeiS	DUF2542 family protein	0.0170	0.4653
JW5361	yeiW	UPF0153 cysteine cluster protein	0.0316	0.3930
JW5362	yeiP	elongation factor P-like protein	0.0215	0.4822
JW5363	bcr	bicyclomycin/cysteine/sulfonamide efflux transporter	0.0865	0.0069
JW5366	ccmA	heme export ABC transporter ATPase	0.0017	0.9197
JW5367	napB	nitrate reductase, small, cytochrome C550 subunit, periplasmic	-0.0351	0.2542
JW5368	yojL	putative thiamine-synthetic flavin transferase lipoprotein	0.1042	0.0003
JW5371	yfaZ	outer membrane protein, putative porin	-0.0292	0.3094
JW5372	yfbE	uridine 5'-(beta-1-threo-pentapyranosyl-4-ulose diphosphate) aminotransferase, PLP-dependent	0.0404	0.0840
JW5373	yfbJ	undecaprenyl phosphate-alpha-L-ara4N exporter; flippase ArnEF subunit	-0.2217	0.0271
JW5374	menD	2-succinyl-5-enolpyruvyl-6-hydroxy-3-cyclohexene-1-carboxylate synthase; SEPHCHC synthase	0.0567	0.0002

JW5375	nuoC	NADH:ubiquinone oxidoreductase, fused CD subunit	0.0684	0.0839
JW5376	yfbT	hexitol phosphatase A	0.0760	0.1512
JW5377	yfcE	phosphodiesterase activity on bis-pNPP	0.0639	0.1053
JW5378	dedD	membrane-anchored periplasmic protein involved in septation	-0.0098	0.7069
JW5380	trmC	fused 5-methylaminomethyl-2-thiouridine-forming enzyme methyltransferase and FAD-dependent demodification enzyme	-0.0206	0.2516
JW5381	yfcM	Elongation Factor P Lys34 hydroxylase	0.0656	0.0002
JW5382	yfdI	serotype-specific glucosyl transferase, CPS-53 (KpLE1) prophage	0.0122	0.6715
JW5383	tfaS	pseudogene, CPS-53 (KpLE1) prophage; tail fiber assembly protein fragment	-0.0743	0.0054
JW5384	yfdL	pseudogene, CPS-53 (KpLE1) prophage	-0.0241	0.3398
JW5385	yfdN	CPS-53 (KpLE1) prophage; uncharacterized protein	0.0461	0.0711
JW5386	ypdJ	exisonase remnant	0.0464	0.2894
JW5387	torI	response regulator inhibitor for tor operon	0.0014	0.9634
JW5388	ypdA	sensor kinase regulating yhjX; pyruvate-responsive YpdAB two-component system	0.0002	0.9953
JW5389	ypdH	putative enzyme IIB component of PTS	0.0604	0.0466
JW5391	yfeA	putative diguanylate cyclase	-0.0253	0.6536
JW5394	ucpA	furfural resistance protein, putative short-chain oxidoreductase	-0.0703	0.0167
JW5395	yfeW	penicillin binding protein PBP4B; weak DD-carboxypeptidase activity	-0.0289	0.3660
JW5396	ypfH	palmitoyl-CoA esterase activity, uncertain physiological substrate	-0.0643	0.0511
JW5397	hda	ATPase regulatory factor involved in DnaA inactivation	0.0431	0.0575
JW5399	yfgG	uncharacterized protein	-0.0209	0.3589
JW5400	yfgH	outer membrane integrity lipoprotein	-0.0688	0.0009
JW5401	guaB	IMP dehydrogenase	0.0470	0.0221
JW5402	yfgJ	DUF1407 family protein	0.0276	0.4687
JW5404	sseB	rhodanase-like enzyme, sulfur transfer from thiosulfate	0.0854	0.0080
JW5405	yphG	DUF4380 domain-containing TPR repeat protein	-0.0260	0.2867
JW5406	yphH	putative DNA-binding transcriptional regulator	0.0524	0.0576
JW5407	yfhK	sensor protein kinase regulating glmY sRNA in two-component system with response regulator GlrR	-0.0064	0.8736

JW5408	yfhB	phosphatidylglycerophosphatase C, membrane bound	-0.1043	0.0558
JW5409	yfiP	DTW domain protein	-0.0187	0.5942
JW5412	yfiL	lipoprotein	-0.1560	0.0062
JW5413	rimM	ribosome maturation factor	-0.1516	0.1170
JW5415	yfjD	UPF0053 family inner membrane protein	0.0299	0.3190
JW5416	recN	recombination and repair protein	-0.1163	0.2096
JW5418	yfjO	CP4-57 prophage; uncharacterized protein	-0.0117	0.5092
JW5419	yfjP	CP4-57 prophage; 50S ribosome-binding GTPase family protein	-0.3229	0.0029
JW5420	ypjM	Uncharacterized protein	-0.3206	0.0018
JW5421	ypjJ	uncharacterized protein	0.0239	0.4463
JW5422	ypjA	adhesin-like autotransporter	-0.0321	0.1231
JW5423	pinH	pseudogene, invertase resolvase family	-0.0605	0.6168
JW5424	ypjC	Putative uncharacterized protein	0.0186	0.1310
JW5425	ygaQ	uncharacterized protein	-0.0591	0.0383
JW5426	yqaC	Putative uncharacterized protein	-0.0023	0.9307
JW5427	ygaT	carbon starvation protein	0.0751	0.0852
JW5428	ygaY	Putative uncharacterized transporter	0.0325	0.1144
JW5429	srlA	glucitol/sorbitol-specific enzyme IIC component of PTS	0.0515	0.1193
JW5430	srlE	glucitol/sorbitol-specific enzyme IIB component of PTS	-0.0240	0.2883
JW5431	gutQ	D-arabinose 5-phosphate isomerase	0.0010	0.9796
JW5433	hypF	carbamoyl phosphate phosphatase and [NiFe] hydrogenase maturation protein	0.2252	0.0304
JW5434	ascG	asc operon transcriptional repressor; prpBC operon repressor	-0.0725	0.0970
JW5435	ascF	cellobiose/arbutin/salicin-specific PTS enzymes, IIB and IC components	0.0718	0.0157
JW5437	rpoS	RNA polymerase, sigma S (sigma 38) factor	0.1366	0.1004
JW5438	ygbF	CRISPR adaptation ssRNA endonuclease	0.0467	0.2823
JW5440	ygcQ	putative flavoprotein	-0.0619	0.0075
JW5441	ygcR	putative flavoprotein	0.0163	0.5764
JW5442	ygcU	putative FAD-linked oxidoreductase	0.0189	0.3152
JW5443	ygcW	putative SDR family oxidoreductase	0.0531	0.0942
JW5444	ygcE	putative kinase	-0.0612	0.0872
JW5445	ygcG	TPM domain protein, putative phosphatase	0.0227	0.5970
JW5446	exo	Ssb-binding protein, misidentified as ExoIX	-0.0082	0.7353
JW5448	ygdI	DUF903 family verified lipoprotein	0.0203	0.1536
JW5449	amiC	N-acetylmuramoyl-L-alanine amidase	-0.0571	0.0783
JW5450	ygdB	DUF2509 family protein	0.0972	0.1469
JW5451	ppdB	putative prepilin peptidase-dependent protein	-0.2422	0.0008
JW5453	yqeF	short chain acyltransferase	-0.0338	0.1312

JW5454	yqeH	putative LuxR family transcriptional regulator	-0.0453	0.5483
JW5455	yqeJ	uncharacterized protein	0.1201	0.2291
JW5456	ygeI	uncharacterized protein	-0.0081	0.7619
JW5457	pbl	pseudogene, peptidoglycan-binding enzyme family	0.0851	0.0239
JW5458	ygeK	pseudogene, response regulator family, part of T3SS PAI ETT2 remnant	-0.0288	0.3507
JW5459	ygeM	pseudogene, orgB family, part of T3SS PAI ETT2 remnant	-0.0032	0.9006
JW5460	ygeN	Putative uncharacterized protein	0.1041	0.0081
JW5461	ygeQ	Putative uncharacterized protein	-0.1064	0.2156
JW5462	xdhA	xanthine dehydrogenase, molybdenum binding subunit	0.0042	0.9034
JW5463	ygeW	putative carbamoyltransferase	0.0201	0.3243
JW5464	yqeC	putative selenium-dependent hydroxylase accessory protein	-0.0137	0.6744
JW5466	guaD	guanine deaminase	-0.0225	0.7195
JW5467	ygfQ	guanine/hypoxanthine permease, high affinity; guanine/hypoxanthine:H+ symporter	0.0285	0.3104
JW5468	ygfS	putative 4Fe-4S ferredoxin-type oxidoreductase subunit	-0.3040	0.0409
JW5469	ygfT	putative oxidoreductase, Fe-S subunit/nucleotide-binding subunit	0.0303	0.4949
JW5470	ygfU	uric acid permease	0.0604	0.1304
JW5473	ygfB	UPF0149 family protein	0.0483	0.3214
JW5475	rpiA	ribose 5-phosphate isomerase, constitutive	-0.2374	0.0004
JW5476	ygfI	putative DNA-binding transcriptional regulator	0.0717	0.1962
JW5477	yggP	putative Zn-binding dehydrogenase	-0.0497	0.0022
JW5478	tktA	transketolase 1, thiamine triphosphate-binding	0.0287	0.3457
JW5479	yggU	UPF0235 family protein	-0.0038	0.8720
JW5481	mltC	membrane-bound lytic murein transglycosylase C	-0.0641	0.3801
JW5482	speC	ornithine decarboxylase, constitutive	-0.0469	0.0861
JW5484	yghF	pseudogene, secretion pathway protein, C-type protein homology	-0.0213	0.5749
JW5486	glcF	glycolate oxidase 4Fe-4S iron-sulfur cluster subunit	0.0314	0.4073
JW5487	glcE	glycolate oxidase FAD binding subunit	0.1546	0.0006
JW5490	yghQ	putative inner membrane polysaccharide flippase	-0.0544	0.1414
JW5491	yghS	putative ATP-binding protein	-0.0754	0.0007
JW5492	yghU	putative S-transferase	-0.0720	0.0281
JW5493	hybF	protein involved with the maturation of hydrogenases 1 and 2	0.0535	0.0613
JW5494	hybB	putative hydrogenase 2 cytochrome b type component	0.0172	0.6778
JW5496	yghY	Putative hydrolase	-0.0798	0.0059

JW5499	dkgA	2,5-diketo-D-gluconate reductase A	-0.0267	0.3680
JW5500	yqhG	DUF3828 family putative periplasmic protein	-0.0715	0.0006
JW5501	ygiQ	Radical SAM superfamily protein	-0.1169	0.0525
JW5503	tolC	transport channel	-0.3114	0.0001
JW5505	yqiC	accessory factor for ubiquinone biosynthesis; BMFP family putative fusogenic protein	-0.0691	0.1752
JW5507	yqiG	pseudogene; fimbrial export usher family	0.0955	0.0498
JW5508	yqiH	putative periplasmic pilin chaperone	-0.0004	0.9881
JW5509	yqiI	fimbrial protein	0.0932	0.0119
JW5510	ygjG	putrescine:2-oxoglutaric acid aminotransferase, PLP-dependent	-0.0168	0.5399
JW5511	ebgA	evolved beta-D-galactosidase, alpha subunit	-0.0112	0.7531
JW5512	ygjI	putative transporter	-0.0787	0.0883
JW5513	ygjO	23S rRNA m(2)G1835 methyltransferase, SAM-dependent	0.0095	0.6173
JW5514	ygjP	UTP pyrophosphatase	0.0196	0.5034
JW5515	alx	putative membrane-bound redox modulator	0.0012	0.9553
JW5516	yqjC	DUF1090 family putative periplasmic protein	0.0113	0.5496
JW5517	yhaL	uncharacterized protein	-0.3011	0.0332
JW5518	yhaM	putative L-serine dehydratase alpha chain	0.0363	0.3805
JW5519	yhaO	putative transporter	0.0762	0.0114
JW5520	tdcG	L-serine dehydratase 3, anaerobic	-0.0583	0.6687
JW5522	tdcE	pyruvate formate-lyase 4/2-ketobutyrate formate-lyase	0.0722	0.0372
JW5525	tdcR	L-threonine dehydratase operon activator protein	0.0457	0.0153
JW5526	garR	tartronate semialdehyde reductase	-0.0253	0.3779
JW5527	agaA	pseudogene, N-acetylgalactosamine-6-phosphate deacetylase fragment	0.1078	0.0375
JW5528	yraR	putative nucleoside-diphosphate-sugar epimerase	0.0084	0.7850
JW5529	yhbO	stress-resistance protein	0.0347	0.5038
JW5531	deaD	ATP-dependent RNA helicase	-0.1240	0.0463
JW5533	yhbC	ribosome maturation factor for 30S subunits	-0.0190	0.3594
JW5534	yhbX	putative EptAB family phosphoethanolamine transferase, inner membrane protein	-0.0948	0.0164
JW5536	arcB	aerobic respiration control sensor histidine protein kinase, cognate to two-component response regulators ArcA and RssB	-0.1866	0.0022
JW5538	nanK	N-acetylmannosamine kinase	0.0182	0.5321
JW5539	yhcB	DUF1043 family inner membrane-anchored protein	0.0352	0.4570
JW5540	yhcN	cadmium and peroxide resistance protein, stress-induced	-0.0099	0.7509
JW5541	aaeX	DUF1656 family putative inner membrane efflux pump associated protein	-0.0175	0.5399
JW5542	yhdP	DUF3971-AsmA2 domains protein	0.1117	0.0781

JW5543	yhdJ	DNA adenine methyltransferase, SAM-dependent	0.0532	0.0067
JW5544	yhdX	putative amino acid ABC transporter permease	0.0625	0.0279
JW5545	yhdY	putative amino acid ABC transporter permease	0.0419	0.2707
JW5546	zraP	Zn-dependent periplasmic chaperone	0.0443	0.0220
JW5547	nfi	endonuclease V; deoxyinosine 3' endonuclease	0.0098	0.6998
JW5548	nudC	NADH pyrophosphatase	-0.0297	0.3372
JW5549	thiG	thiamine biosynthesis ThiGH complex subunit	0.0839	0.0811
JW5551	sthA	pyridine nucleotide transhydrogenase, soluble	-0.0684	0.0048
JW5553	argB	acetylglutamate kinase	-0.0047	0.9025
JW5555	ptsA	putative PTS enzyme: Hpr, enzyme I and II components	-0.0160	0.5630
JW5556	gldA	glycerol dehydrogenase, NAD+ dependent; 1,2-propanediol:NAD+ oxidoreductase	0.0145	0.6244
JW5557	yijE	EamA-like transporter family protein	0.0229	0.4675
JW5558	cpxP	inhibitor of the cpx response; periplasmic adaptor protein	-0.0946	0.0101
JW5559	yiiM	6-N-hydroxylaminopurine resistance protein	-0.0227	0.3341
JW5560	kdgT	2-keto-3-deoxy-D-gluconate transporter	0.0537	0.0622
JW5562	frvB	putative PTS enzyme, IIB component/IIC component	0.0270	0.4563
JW5563	yiiF	putative thymol sensitivity protein, CopG family putative transcriptional regulator	0.3471	0.0154
JW5566	yihX	alpha-D-glucose-1-phosphate phosphatase, anomer-specific	0.0175	0.6971
JW5567	yihW	putative transcriptional regulator for sulphoquinovose utilization	-0.0312	0.1580
JW5568	yihV	6-deoxy-6-sulphofructose kinase	0.0168	0.4207
JW5569	yihS	sulphoquinovose isomerase	0.0244	0.5116
JW5571	bipA	GTP-binding protein	0.0306	0.4736
JW5574	yihF	DUF945 family protein	0.0637	0.1105
JW5575	mobB	molybdopterin-guanine dinucleotide biosynthesis protein B	0.1380	0.0206
JW5576	trkH	potassium transporter	-0.1565	0.0000
JW5577	yigZ	UPF0029 family protein	-0.2134	0.1372
JW5578	fadA	3-ketoacyl-CoA thiolase (thiolase I)	-0.0824	0.2975
JW5580	tatB	TatABCE protein translocation system subunit	-0.1520	0.0789

JW5581	ubiE	bifunctional 2-octaprenyl-6-methoxy-1,4-benzoquinone methylase/ S-adenosylmethionine:2-DMK methyltransferase	-0.4016	0.0033
JW5584	pldB	lysophospholipase L2	0.0005	0.9907
JW5585	rhtB	homoserine, homoserine lactone and S-methyl-methionine efflux pump	0.0022	0.8995
JW5586	rhtC	threonine efflux pump	0.0084	0.7208
JW5588	yigI	4HBT thioesterase family protein	0.0312	0.3436
JW5589	rarD	putative chloramphenical resistance permease	0.0233	0.5546
JW5590	yigG	PRK11371 family inner membrane protein	0.1327	0.0025
JW5591	yigE	DUF2233 family protein	0.0768	0.0996
JW5592	dapF	diaminopimelate epimerase	0.0801	0.6660
JW5594	aslB	putative AslA-specific sulfatase-maturating enzyme	0.0821	0.0235
JW5595	yifK	putative APC family amino acid transporter	0.1584	0.0003
JW5596	rffT	TDP-Fuc4NAc:lipidIIFuc4NAc transferase	0.0326	0.4719
JW5597	rffC	TDP-fucosamine acetyltransferase	0.1092	0.0488
JW5598	rffG	dTDP-glucose 4,6-dehydratase	-0.0876	0.0471
JW5599	rffD	UDP-N-acetyl-D-mannosaminuronic acid dehydrogenase	0.1000	0.0073
JW5600	rffE	UDP-N-acetyl glucosamine-2-epimerase	0.1464	0.0016
JW5601	wzzE	Entobacterial Common Antigen (ECA) polysaccharide chain length modulation protein	0.0329	0.5230
JW5603	gpp	guanosine pentaphosphatase/exopolyphosphatase	0.0085	0.6410
JW5604	rep	DNA helicase and single-stranded DNA-dependent ATPase	-0.1013	0.0135
JW5605	ilvD	dihydroxyacid dehydratase	-0.0506	0.1600
JW5606	ilvE	branched-chain amino acid aminotransferase	0.0396	0.5013
JW5607	hdfR	flhDC operon transcriptional repressor	0.0184	0.6055
JW5608	yieP	putative transcriptional regulator	-0.0018	0.9432
JW5609	trkD	potassium transporter	0.0584	0.2979
JW5610	yieM	stimulator of RavA ATPase activity; von Willebrand factor domain protein	0.0122	0.3900
JW5611	atpI	ATP synthase, membrane-bound accessory factor	0.0337	0.3883
JW5612	yieL	putative xylanase	-0.0323	0.2880
JW5613	yieK	putative 6-phosphogluconolactonase	0.0110	0.8021
JW5619	tnaB	Low affinity tryptophan permease	-0.0344	0.0710
JW5627	dgoR	D-galactonate catabolism operon transcriptional repressor	-0.0145	0.5661
JW5628	dgoA	2-oxo-3-deoxygalactonate 6-phosphate aldolase	0.0031	0.8922

JW5629	dgoD	D-galactonate dehydratase	-0.0484	0.2120
JW5631	cbrA	colicin M resistance protein; FAD-binding protein, putative oxidoreductase	0.0321	0.4539
JW5633	yidQ	DUF1375 family outer membrane protein	0.0315	0.4684
JW5634	emrD	multidrug efflux system protein	-0.0110	0.6725
JW5636	yicO	adenine permease, high affinity; adenine:H+ symporter	0.0094	0.7385
JW5637	yicN	DUF1198 family protein	-0.0912	0.0342
JW5641	ttk	nucleoid occlusion factor, anti-FtsZ division inhibitor	0.1079	0.1461
JW5643	yicR	UPF0758 family protein	-0.3016	0.0207
JW5644	htrL	YibB family protein, function unknown	0.0862	0.2592
JW5645	yibQ	putative polysaccharide deacetylase	0.1587	0.0070
JW5646	envC	activator of AmiB,C murein hydrolases, septal ring factor	0.0800	0.0047
JW5648	yiaY	L-threonine dehydrogenase	0.0145	0.7127
JW5650	sgbU	putative L-xylulose 5-phosphate 3-epimerase	-0.0578	0.0941
JW5651	yiaN	2,3-diketo-L-gulonate TRAP transporter large permease protein	-0.5407	0.0007
JW5652	avtA	valine-pyruvate aminotransferase; transaminase C; alanine-valine transaminase	-0.3646	0.0463
JW5653	bax	putative glucosaminidase	-0.0457	0.5934
JW5654	yiaB	YiaAB family inner membrane protein	-0.5678	0.0004
JW5655	yiaF	barrier effect co-colonization resistance factor; DUF3053 family lipoprotein	-0.0349	0.1831
JW5656	tiaE	glyoxylate/hydroxypyruvate reductase B	-0.0654	0.0001
JW5657	yiaD	multicopy suppressor of bamB; outer membrane lipoprotein	0.1067	0.0606
JW5659	yhjY	autotransporter beta-domain protein	-0.0456	0.0667
JW5660	eptB	KDO phosphoethanolamine transferase, Ca(2+)-inducible	-0.0247	0.3512
JW5663	bcsF	DUF2636 family cellulose production small membrane protein	-0.3208	0.0052
JW5665	bcsA	cellulose synthase, catalytic subunit	-0.0449	0.0971
JW5668	kdgK	2-dehydro-3-deoxygluconokinase	0.0630	0.0344
JW5669	hdeB	acid-resistance protein	-0.0341	0.4263
JW5670	yhiD	putative Mg(2+) transport ATPase, inner membrane protein	0.1055	0.1865
JW5672	yhiQ	16S rRNA m(2)G1516 methyltransferase, SAM-dependent	0.0764	0.0129
JW5674	yhiK	pseudogene, DUF4049 family protein	0.0246	0.1983
JW5676	rbbA	ribosome-associated ATPase: ATP-binding protein/ATP-binding membrane protein	-0.0314	0.2496
JW5677	yhhJ	putative ABC transporter permease	0.0948	0.0755
JW5678	yrhC	pseudogene fragment	0.0428	0.2978

JW5679	rhsB	Rhs protein with DUF4329 family putative toxin domain; putative neighboring cell growth inhibitor	0.1704	0.0000
JW5680	yhhT	UPF0118 family putative transporter	-0.0032	0.2945
JW5682	dcrB	putative lipoprotein	-0.0928	0.0126
JW5683	yhhL	DUF1145 family protein	-0.0244	0.1366
JW5686	gntU	gluconate transporter, low affinity GNT 1 system	0.0434	0.0550
JW5687	glpG	rhomboid intramembrane serine protease	-0.0437	0.0197
JW5688	rtcA	RNA 3'-terminal phosphate cyclase	-0.1279	0.0067
JW5689	malP	maltodextrin phosphorylase	-0.1404	0.3027
JW5690	gntT	gluconate transporter, high-affinity GNT I system	0.1140	0.0160
JW5691	gntX	DNA catabolic protein	-0.1110	0.0006
JW5692	hslO	heat shock protein Hsp33	0.0839	0.0304
JW5693	yrfD	DNA catabolic putative pilus assembly protein	-0.1613	0.0106
JW5694	yrfA	DNA catabolic protein	-0.1083	0.0236
JW5696	yhfY	PRD domain protein	0.0233	0.3289
JW5697	yhfU	DUF2620 family protein	0.0951	0.0068
JW5698	frlR	putative DNA-binding transcriptional regulator	-0.0381	0.1158
JW5699	frlC	fructoselysine 3-epimerase	-0.1066	0.0540
JW5700	frlB	fructoselysine-6-P-deglycase	0.0363	0.0415
JW5701	yhfK	putative transporter, FUSC superfamily inner membrane protein	0.0320	0.5297
JW5702	crp	cAMP-activated global transcription factor, mediator of catabolite repression	0.3845	0.0000
JW5703	yheO	putative PAS domain-containing DNA-binding transcriptional regulator	0.0657	0.0551
JW5704	gspM	general secretory pathway component, cryptic	-0.0894	0.0476
JW5705	gspL	general secretory pathway component, cryptic	-0.0252	0.6453
JW5706	gspI	general secretory pathway component, cryptic	-0.0495	0.1081
JW5707	gspD	general secretory pathway component, cryptic	0.0181	0.7329
JW5708	smf	DNA recombination-mediator A family protein	0.0825	0.0224
JW5710	yrdA	bacterial transferase hexapeptide domain protein	-0.1904	0.0076
JW5711	yjbF	extracellular polysaccharide production lipoprotein	0.0015	0.9594
JW5713	ubiC	chorismate--pyruvate lyase	-0.0412	0.1819
JW5714	zur	transcriptional repressor, Zn(II)-binding	-0.0701	0.1865
JW5716	yjbO	phage shock protein G	0.0117	0.7080
JW5718	yjcB	putative inner membrane protein	-0.0071	0.7672

JW5721	yjcS	putative alkylsulfatase; SDS catabolic enzyme	-0.0144	0.4810
JW5727	phnK	carbon-phosphorus lyase complex subunit, putative ATP transporter ATP-binding protein	0.0135	0.7384
JW5729	yjcZ	YjcZ family protein; yhjH motility defect suppressor	-0.0549	0.1108
JW5731	adiA	arginine decarboxylase	0.0027	0.9316
JW5733	yjdC	putative transcriptional regulator	0.2375	0.1931
JW5734	dipZ	thiol:disulfide interchange protein and activator of DsbC	0.0001	0.9975
JW5735	dcuA	C4-dicarboxylate antiporter	-0.0154	0.7296
JW5736	yjeI	DUF4156 family lipoprotein	0.0430	0.2790
JW5737	ecnA	entericidin A membrane lipoprotein, antidote entericidin B	-0.0983	0.1309
JW5738	sugE	multidrug efflux system protein	0.1011	0.0169
JW5739	yjeM	putative transporter	0.0184	0.5007
JW5741	rnr	exoribonuclease R, RNase R	0.0093	0.7329
JW5742	yjfN	DUF1471 family periplasmic protein	0.0414	0.1117
JW5743	yjfO	bioflm peroxide resistance protein	-0.0180	0.5782
JW5744	ulaA	L-ascorbate-specific enzyme IIC permease component of PTS	-0.3299	0.0373
JW5745	ytfB	OapA family protein	0.1105	0.0187
JW5746	fklB	FKBP-type peptidyl-prolyl cis-trans isomerase (rotamase)	-0.0519	0.0377
JW5747	ytfH	DUF24 family HxlR-type putative transcriptional regulator	-0.0181	0.3724
JW5748	ytfI	uncharacterized protein	-0.0254	0.0504
JW5749	ytfK	DUF1107 family protein	0.0084	0.6646
JW5752	ytfR	putative sugar ABC transporter ATPase	0.0217	0.2434
JW5753	ytfT	putative sugar ABC transporter permease	0.0622	0.0594
JW5754	yjfF	putative sugar ABC transporter permease	0.0314	0.1452
JW5755	yjgF	enamine/imine deaminase, reaction intermediate detoxification	0.0782	0.0248
JW5756	yjgK	biofilm modulator regulated by toxins; DUF386 family protein, cupin superfamily protein	-0.1295	0.2563
JW5757	yjgL	SopA-central-domain-like hexapeptide repeat protein	-0.0593	0.0000
JW5758	yjgM	GNAT family putative N-acetyltransferase	-0.0354	0.1181
JW5759	yjgN	DUF898 family inner membrane protein	-0.0780	0.0244
JW5761	yjgB	broad specificity NADPH-dependent aldehyde reductase, Zn-containing	-0.0643	0.0975
JW5763	yjgX	Putative uncharacterized protein	0.0251	0.0248
JW5768	yjhB	putative MFS transporter, membrane protein	-0.0012	0.9650
JW5769	yjhC	GFO/IDH/MOCA family putative oxidoreductase. NAD(P)-dependent	-0.0147	0.6820
JW5770	yjhD	pseudogene, KpLE2 phage-like element	-0.0464	0.1011

JW5775	yjhH	putative lyase/synthase	0.0254	0.2832
JW5776	sgcX	putative endoglucanase with Zn-dependent exopeptidase domain	-0.0912	0.3162
JW5778	yjhA	N-acetylnuraminic acid outer membrane channel protein	-0.0141	0.5272
JW5780	fimD	fimbrial usher outer membrane porin protein; FimCD chaperone-usher	0.0390	0.2233
JW5782	yjiD	RpoS stabilzer after DNA damage, anti-RssB factor	-0.0268	0.1607
JW5783	yjiH	nucleoside recognition pore and gate family putative inner membrane transporter	-0.0151	0.5530
JW5784	kptA	RNA 2'-phosphotransferase	0.0814	0.0806
JW5785	yjiL	putative ATPase, activator of (R)-hydroxyglutaryl-CoA dehydratase	0.0114	0.4175
JW5786	yjiM	putative 2-hydroxyglutaryl-CoA dehydratase	-0.0083	0.7120
JW5787	yjiT	pseudogene	-0.0801	0.0770
JW5789	mcrC	5-methylcytosine-specific restriction enzyme McrBC, subunit McrC	-0.0312	0.0617
JW5790	yjiA	metal-binding GTPase	-0.1145	0.0893
JW5791	yjiY	pyruvate/H+ symporter	0.0455	0.2774
JW5792	yjjM	putative transcriptional activator for L-galactonate catabolism	-0.0490	0.0867
JW5793	yjjN	L-galactonate oxidoreductase; L-gulonate oxidoreductase	-0.0336	0.2662
JW5794	mdoB	OPG periplasmic biosynthetic phosphoglycerol transferases I (membrane-bound) and II (soluble);	-0.0202	0.2769
JW5795	yjjA	putative DUF2501 family periplasmic protein	0.0013	0.9553
JW5796	yjjP	DUF1212 family inner membrane protein	-0.0569	0.0040
JW5797	yjjZ	uncharacterized protein	-0.0191	0.3313
JW5800	nadR	trifunctional protein: nicotinamide mononucleotide adenylyltransferase, ribosylnicotinamide kinase, transcriptional repressor	-0.0171	0.3059
JW5801	yjjX	non-canonical purine NTP phosphatase, ITPase/XTPase	-0.0646	0.0185
JW5802	ydbA	Putative exported protein	-0.0054	0.7344
JW5803	ybhR	putative ABC transporter permease	-0.0007	0.9765
JW5804	ycjY	S9 homolog non-peptidase family protein	-0.0825	0.0045
JW5805	hyfI	hydrogenase 4, Fe-S subunit	0.0249	0.1958
JW5806	tdcD	propionate kinase/acetate kinase C, anaerobic	-0.0520	0.1258
JW5807	leuB	3-isopropylmalate dehydrogenase, NAD(+)-dependent	0.2662	0.1347
JW5808	pcnB	poly(A) polymerase	-0.0322	0.2740

JW5811	fhiA	pseudogene, flagellar system protein, promoterless fragment	-0.0169	0.4112
JW5812	mbhA	pseudogene, lateral flagellar motor protein fragment	0.0215	0.4055
JW5813	ykfC	CP4-6 prophage; conserved protein	0.0529	0.0449
JW5814	yaiF	Putative uncharacterized acetyltransferase	0.0752	0.0790
JW5815	tfaD	pseudogene, DLP12 prophage; tail fiber assembly protein family	-0.0596	0.0027
JW5816	ybfE	LexA-regulated protein, CopB family	0.0136	0.6181
JW5818	potG	putrescine ABC transporter ATPase	0.0155	0.7125
JW5819	ybjS	putative NAD(P)H-dependent oxidoreductase	0.0298	0.3020
JW5820	ycfS	L,D-transpeptidase linking Lpp to murein	-0.0250	0.2730
JW5821	emtA	lytic murein endotransglycosylase E	0.1286	0.2482
JW5822	abgT	p-aminobenzoyl-glutamate transporter; membrane protein	0.1082	0.0030
JW5823	ydcH	DUF465 family protein	0.0647	0.0035
JW5825	yneF	putative membrane-bound diguanylate cyclase	-0.1791	0.1795
JW5826	asr	acid shock-inducible periplasmic protein	0.0172	0.5746
JW5827	ydhL	DUF1289 family protein	0.0364	0.2496
JW5830	yebN	putative Mn(2+) efflux pump, mntR-regulated	-0.0894	0.0192
JW5831	znuA	zinc ABC transporter periplasmic binding protein	0.0334	0.1746
JW5832	yedQ	putative membrane-anchored diguanylate cyclase	0.0155	0.4204
JW5833	yeeJ	putative adhesin	0.0350	0.4685
JW5834	yeeY	LysR family putative transcriptional regulator	-0.0408	0.0803
JW5836	cld	regulator of length of O-antigen component of lipopolysaccharide chains	0.0541	0.0005
JW5837	yegR	uncharacterized protein	0.1053	0.0004
JW5838	yohG	pseudogene	0.0583	0.0889
JW5839	yejO	pseudogene, autotransporter outer membrane homology	0.1468	0.0870
JW5840	elaD	protease, capable of cleaving an AMC-ubiquitin model substrate	-0.0031	0.9001
JW5841	prmB	N5-glutamine methyltransferase	0.0774	0.0078
JW5842	yphC	putative Zn-dependent NAD(P)-binding oxidoreductase	0.0340	0.1862
JW5843	norR	anaerobic nitric oxide reductase DNA-binding transcriptional activator	0.0250	0.1759
JW5844	ygcI	CRISP RNA (crRNA) containing Cascade antiviral complex protein	-0.0456	0.0264
JW5845	ygcS	putative MFS sugar transporter; membrane protein	0.0122	0.6614
JW5846	ygeO	pseudogene, orgA family, part of T3SS PAI ETT2 remnant	0.2286	0.0326
JW5847	prfB	peptide chain release factor RF-2	0.0000	0.9987
JW5848	yghO	pseudogene, IS-interrupted	-0.0172	0.5254

JW5849	yqhC	transcriptional activator of yqhD	0.0964	0.0052
JW5850	yqjF	putative quinol oxidase subunit, trinitrotoluene-inducible	0.0147	0.6593
JW5851	pnp	polynucleotide phosphorylase/polyadenylase	0.1194	0.1587
JW5852	yihO	putative sulphoquinovose importer	0.0228	0.3611
JW5853	ysgA	putative carboxymethylenebutenolidase	-0.1316	0.0000
JW5854	yigL	pyridoxal phosphate phosphatase	0.1698	0.0200
JW5855	recQ	ATP-dependent DNA helicase	0.0307	0.3250
JW5856	trxA	thioredoxin 1	-0.0225	0.6552
JW5857	rbsD	D-ribose pyranase	0.0452	0.0111
JW5858	yidX	putative lipoprotein	0.0493	0.0065
JW5859	dgoT	D-galactonate transporter	0.0385	0.1873
JW5860	yidR	DUF3748 family protein	0.0491	0.0251
JW5864	yrhA	pseudogene, interrupted by IS1E	0.1836	0.0493
JW5865	yrfG	GMP/IMP nucleotidase	-0.1370	0.0190
JW5867	aidB	DNA alkylation damage repair protein; flavin-containing DNA binding protein, weak isovaleryl CoA dehydrogenase	-0.0345	0.3218
JW5868	ulaG	L-ascorbate 6-phosphate lactonase	-0.1606	0.0009
JW5869	yjiK	SdiA-regulated family putative membrane-anchored protein; putative phytase-like esterase	-0.0780	0.0020
JW5871	mcrB	5-methylcytosine-specific restriction enzyme McrBC, subunit McrB	0.0107	0.7455
JW5873	prfC	peptide chain release factor RF-3	-0.2528	0.0001
JW5874	ydhM	transcriptional repressor for the nemRA-gloA operon, quinone-, glyoxal-, and HOCl-activated	-0.0379	0.0659
JW5875	nuoB	NADH:ubiquinone oxidoreductase, chain B	0.0376	0.6168
JW5876	yfeH	putative inorganic ion transporter	-0.0013	0.9565
JW5877	ypeB	DUF3820 family protein	-0.0205	0.5002
JW5878	csiE	stationary phase inducible protein	-0.0258	0.5026
JW5892	yadB	glutamyl-Q tRNA(Asp) synthetase	0.1507	0.0001
JW5893	yahH	Putative uncharacterized protein	0.0136	0.4643
JW5894	cynR	transcriptional activator of cyn operon; autorepressor	-0.0142	0.5430
JW5896	ybhD	putative DNA-binding transcriptional regulator	-0.0469	0.2426
JW5897	yliA	glutathione ABC transporter ATPase	-0.0619	0.0836
JW5898	yccW	23S rRNA m(5)C1962 methyltransferase, SAM-dependent	-0.0067	0.6558
JW5901	ycgH	Putative uncharacterized protein	0.1068	0.0001
JW5904	lomR	Putative protein	-0.0382	0.1484
JW5905	hrpA	putative ATP-dependent helicase	0.0003	0.9898
JW5906	gapC	Putative uncharacterized protein	0.0606	0.0173
JW5907	yncB	curcumin/dihydrocurcumin reductase, NADPH-dependent	0.0449	0.4444
JW5908	yddM	putative DNA-binding transcriptional regulator	0.0268	0.1366
JW5909	ydfU	Qin prophage; DUF968 family protein	0.0563	0.0040
JW5911	yniD	uncharacterized protein	-0.0337	0.1759
JW5912	yedN	Putative uncharacterized protein	-0.0869	0.2602

JW5913	intG	pseudogene, phage integrase family	0.0192	0.4039
JW5916	molR	Putative molybdate metabolism regulator	-0.0664	0.0866
JW5917	rcsC	Sensor histidine kinase	-0.0013	0.9515
JW5921	yfjS	CP4-57 prophage; uncharacterized protein	-0.0656	0.0013
JW5922	ypjL	pseudogene, CP4-57 prophage	-0.0968	0.0005
JW5923	ygfK	putative Fe-S subunit oxidoreductase subunit	-0.0002	0.9922
JW5924	yghE	pseudogene, secretion pathway protein, L-type protein homology	-0.0162	0.5097
JW5925	yghJ	putative secreted and surface-associated lipoprotein mucinase	-0.0672	0.0705
JW5926	yghX	pseudogene, dienlactone hydrolase family	-0.0205	0.5517
JW5927	ygiB	DUF1190 family protein	0.0562	0.1170
JW5929	yiiE	CopG family putative transcriptional regulator	0.0281	0.2486
JW5931	tatD	quality control of Tat-exported FeS proteins; Mg-dependent cytoplasmic DNase	-0.0110	0.7802
JW5937	ysdC	Aminopeptidase	0.0347	0.2289
JW5938	yicM	putative transporter	-0.0213	0.3948
JW5939	yicJ	putative transporter	-0.0413	0.0793
JW5940	bisC	biotin sulfoxide reductase	0.0272	0.1274
JW5941	yhjQ	involved in cellulose production, minD superfamily (pseudogene)	-0.0600	0.0152
JW5942	bcsC	cellulose synthase subunit	0.0006	0.9802
JW5943	yhjK	cyclic-di-GMP phosphodiesterase	0.0162	0.5665
JW5944	yhiM	acid resistance protein, inner membrane	-0.0245	0.3521
JW5945	yhhS	putative arabinose efflux transporter	0.0882	0.1100
JW5946	gntR	d-gluconate inducible gluconate regulon transcriptional repressor	0.0293	0.3912
JW5948	yhfZ	putative DNA-binding transcriptional regulator	-0.0628	0.1027
JW5949	yrdD	ssDNA-binding protein, function unknown	-0.0136	0.6731
JW5950	yjbN	tRNA-dihydrouridine synthase A	0.1172	0.0094
JW5952	yjhU	putative DNA-binding transcriptional regulator; KpLE2 phage-like element	-0.0382	0.1061
JW5953	yjiP	pseudogene, transposase_31 family protein	0.0301	0.4481
JW5954	yjiV	Putative uncharacterized protein	0.0616	0.0472
JW5955	bglJ	bgl operon transcriptional activator	-0.0649	0.0015
JW5956	ykfH	uncharacterized protein	-0.0173	0.4882
JW5960	ymjC	pseudogene	-0.0299	0.0821
JW5961	yncK	Putative uncharacterized protein	0.0010	0.0198
JW5962	sra	stationary-phase-induced ribosome-associated protein	-0.0319	0.1366
JW5963	blr	beta-lactam resistance membrane protein; divisome-associated protein	-0.0269	0.2911
JW5964	ypaA	pseudogene, C-terminal fragment, Transposase_31 family	0.0222	0.7147
JW5965	yicS	putative periplasmic protein	0.0421	0.0744
JW5967	sgcB	putative enzyme IIB component of PTS	0.0481	0.0098
JW5968	yjhX	UPF0386 family protein	-0.1472	0.1475

[1] Function determined from UniportKB

[2] Each individual score represents the mean of 12 trials – three biological and four technical.

[3] The *p*-value was a two-tailed *t*-test and significance was determined using the Benjamini-Hochberg procedure
